# Transition Metal Catalyst‐Free, Base‐Promoted 1,2‐Additions of Polyfluorophenylboronates to Aldehydes and Ketones

**DOI:** 10.1002/anie.202103686

**Published:** 2021-06-17

**Authors:** Zhiqiang Liu, Goutam Kumar Kole, Yudha P. Budiman, Ya‐Ming Tian, Alexandra Friedrich, Xiaoling Luo, Stephen A. Westcott, Udo Radius, Todd B. Marder

**Affiliations:** ^1^ Institute of Inorganic Chemistry and Institute for Sustainable Chemistry & Catalysis with Boron Julius-Maximilians-Universität Würzburg Am Hubland 97074 Würzburg Germany; ^2^ Department of Chemistry College of Engineering and Technology SRM Institute of Science and Technology SRM Nagar Kattankulathur Tamil Nadu 603203 India; ^3^ Department of Chemistry Faculty of Mathematics and Natural Sciences Universitas Padjadjaran 45363 Jatinangor Indonesia; ^4^ Chongqing Key Laboratory of Inorganic Functional Materials College of Chemistry Chongqing Normal University Chongqing 401331 China; ^5^ Department of Chemistry and Biochemistry Mount Allison University Sackville NB E4L 1G8 Canada

**Keywords:** alcohol, 1,2-addition reaction, boronate esters, fluoroarene, transition metal-free

## Abstract

A novel protocol for the transition metal‐free 1,2‐addition of polyfluoroaryl boronate esters to aldehydes and ketones is reported, which provides secondary alcohols, tertiary alcohols, and ketones. Control experiments and DFT calculations indicate that both the ortho‐F substituents on the polyfluorophenyl boronates and the counterion K^+^ in the carbonate base are critical. The distinguishing features of this procedure include the employment of commercially available starting materials and the broad scope of the reaction with a wide variety of carbonyl compounds giving moderate to excellent yields. Intriguing structural features involving O−H⋅⋅⋅O and O−H⋅⋅⋅N hydrogen bonding, as well as arene‐perfluoroarene interactions, in this series of racemic polyfluoroaryl carbinols have also been addressed.

## Graphical abstract

Herein we report a transition metal‐free procedure for the base‐promoted 1,2‐addition of polyfluorophenylboronates to aldehydes and ketones to generate organofluorine‐containing alcohols in yields up to 93 %.

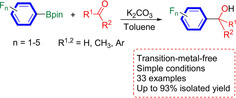

## Introduction

Over the past few decades, the transition‐metal‐catalyzed 1,2‐addition of organometallic reagents to the C=O functionality of aldehydes and ketones has developed as a useful method for the synthesis of substituted secondary and tertiary alcohols.^[1]^ Numerous reagents have been used for these reactions, including organomagnesium,^[2]^ organozinc,[^1^, ^3^] organolithium,^[4]^ organosilane,^[5]^ organostannane,^[6]^ organocerium,^[7]^ and organoboron compounds.^[8]^ In particular, organoboronate reagents offer significant advantages such as air and moisture stability, low toxicity, good functional group tolerance, and availability.^[8]^ In 1998, Miyaura and co‐workers^[9]^ first reported the addition of arylboronic acids to aldehydes using a Rh catalyst. In subsequent studies, other rhodium,^[10]^ palladium,^[11]^ platinum,^[12]^ nickel,^[13]^ copper,^[14]^ iron,^[15]^ cobalt,^[16]^ and ruthenium^[17]^ complexes have been developed as precatalysts for such reactions. However, transition metals can be expensive, toxic, and difficult to remove completely from the corresponding product. A transition metal‐free strategy would be highly desirable for these useful transformations. The reaction products for the addition of arylboronic acids to ketones, after hydrolysis, are tertiary alcohols, which are important building blocks for the synthesis of pharmaceuticals, agrochemical compounds, and natural products.^[18]^ However, the nucleophilic addition of organometallic reagents to ketones can be challenging due to the inherent steric congestion around the carbonyl group, frequently resulting in the generation of products arising from side reactions such as reduction and aldol condensation.^[19]^ Therefore, the development of an efficient, general, and convenient protocol for the synthesis of tertiary alcohols is of considerable interest.

Moreover, an ideal strategy to synthesize ketones, important and ubiquitous structural motifs,^[20]^ lies in the transition metal‐catalyzed replacement of an aldehyde's C(O)‐*H* group with a carbon electrophile.^[21]^ Recently, Zheng and co‐workers demonstrated the direct functionalization of aldehyde C−*H* bonds with aryl halides, using a precious metal palladium catalyst, which has proven to be a viable method to generate the corresponding ketone products.^[22]^

**Scheme 1:**
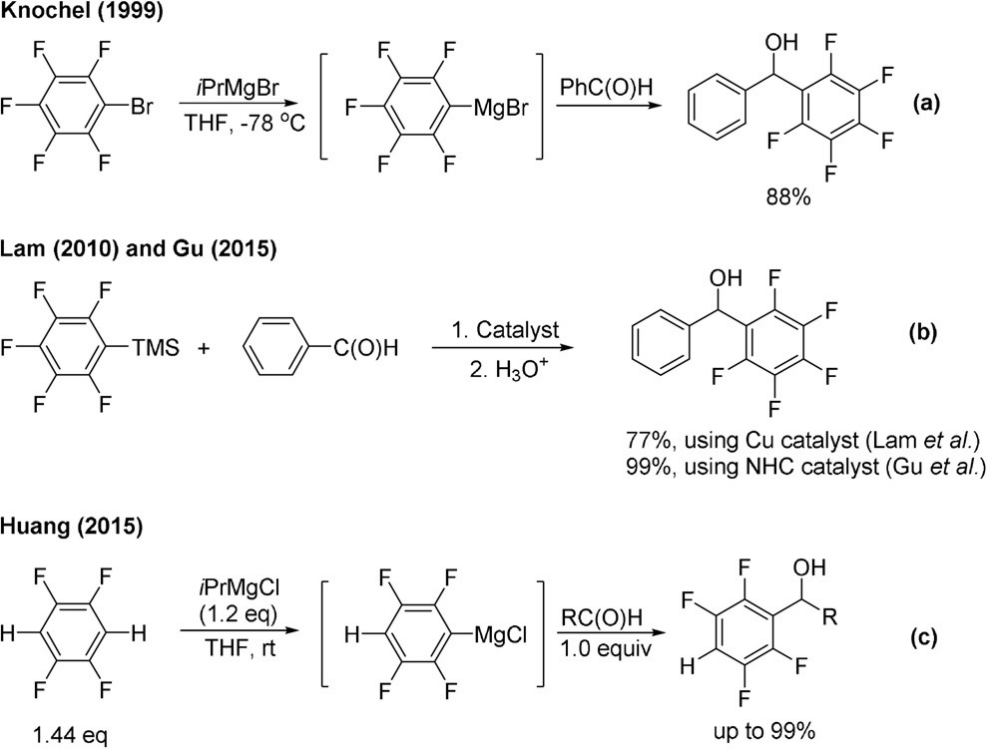
Approaches to access polyfluoroaryl carbinols via the addition to aldehydes.

Polyfluoroarenes have gained extensive attention due to their important role in pharmaceutical, agrochemical, and advanced materials.^[23]^ Thus, identifying practical and efficient concepts for the introduction of fluorine or fluorinated building blocks is highly desirable. Several studies have been reported regarding the polyfluorophenylation of aldehydes. For example, in 1999, Knochel and co‐workers^[24]^ used fluorinated aryl bromides to perform pentafluorophenylation of aldehydes (Scheme 1 a). More recently, Lam and co‐workers^[25]^ used a copper catalyst (Scheme 1 b) and Gu and co‐workers^[26]^ (Scheme 1 b) used an *N*‐heterocyclic carbene (NHC) organocatalyst to obtain fluorinated aryl carbinols using polyfluorophenyl trimethylsilane as a nucleophile for the addition to aldehydes. In 2015, Huang and co‐workers^[27]^ (Scheme 1 c) reported a Mg‐mediated polyfluoroaryl addition to aldehydes. Although some advancements in this field have been reported, these methods suffer from the requirement for highly flammable Grignard reagents, transition metals or NHC catalysts. Moreover, methods reported by Lam and co‐workers and Gu and co‐workers are limited to pentafluorophenyl trimethylsilane or 1,4‐bis (trimethylsilyl) tetrafluorobenzene as substrates.

Recently, we reported efficient methods to generate fluorinated arylboronic acid pinacol esters (Ar_F_‐Bpin) via C‐F borylation of fluoroarenes using NHC‐ligated Ni complex[^28a^, ^28b^] and C‐Cl borylation of Ar_F_‐Cl using Pd catalyst under base free condition.^[28c]^ Likewise, we reported optimized conditions for the Suzuki–Miyaura cross‐coupling reaction of Ar_F_‐Bpin compounds with ArX (X=Br, I) using a combination of CuI and 1,10‐phenanthroline as a catalyst precursor.^[28d]^ Furthermore, we reported the palladium‐catalyzed homocoupling of fluorinated arylboronates,^[28e]^ and the copper‐catalyzed oxidative cross‐coupling of electron‐deficient polyfluorophenyl boronate esters with terminal alkynes.^[28f]^ We report herein the transition metal‐free polyfluorophenylation of ketones and aldehydes with fluorinated aryl boronates, which provides a convenient and novel strategy for the synthesis of alcohols and ketones.

## Results and Discussion

Addition of arylboronic acids to aldehydes using transition metal catalysts has been well developed. We expected that the use of more Lewis acidic pentafluorophenyl‐Bpin with a base would generate a nucleophilic intermediate in the absence of a transition metal. To verify our hypothesis, we initially examined the reaction of pentafluorophenyl‐Bpin (**1 a**) and benzaldehyde (**2 a**) as a model reaction. As shown in Table 1, secondary alcohol **3 a** was observed as the addition product after hydrolysis when the mixture of **1 a** and **2 a** was heated in the presence of KOMe as the base (Table 1, entry 1). Encouraged by this first result, we screened the reaction parameters, including the base and the solvent, to improve the performance of the reaction. The employment of K_2_CO_3_ as the base dramatically increased the yield to 92 % (Table 1, entry 6). The experimental results revealed that heating is required as the room temperature reaction only afforded **3 a** in trace amounts (Table 1, entry 7). Lower conversions were observed when reactions were conducted in coordinating solvents such as DMF, THF, and 1,4‐dioxane (Table 1, entries 8, 10, 11), and the lowest yield was obtained when CH_3_CN was used as the solvent (Table 1, entry 9). In addition, the reaction exhibited very poor performance under aerobic conditions (Table 1, entry 12). Interestingly, increasing the amount of K_2_CO_3_ to 3 equiv was not helpful (Table 1, entry 13). Decreasing the amount of K_2_CO_3_ (0.8 equiv) did not impact the performance of the reaction (Table 1, entry 14). No reaction took place when K_2_CO_3_ was absent (Table 1, entry 15), indicating that K_2_CO_3_ as the base is important for this reaction. Not surprisingly, adventitious water quenched the reaction (Table 1, entries 16, 17). However, under anhydrous conditions, the transition metal‐free polyfluorophenylation of benzaldehyde with pentafluorophenyl‐Bpin is feasible and leads to high yields of the desired product.

**Table 1:** Optimization of the reaction conditions.^[a]^ 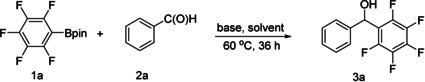

Entry	Base	Solvent	Yield [%]^[b]^
1	KOMe	toluene	20
2	KF	toluene	25
3	^*t*^BuOLi	toluene	52
4	Cs_2_CO_3_	toluene	60
5	Na_2_CO_3_	toluene	78
6	K_2_CO_3_	toluene	92
7^[c]^	K_2_CO_3_	toluene	trace
8	K_2_CO_3_	DMF	50
9	K_2_CO_3_	CH_3_CN	15
10	K_2_CO_3_	THF	88
11	K_2_CO_3_	1,4‐dioxane	79
12^[d]^	K_2_CO_3_	toluene	35
13^[e]^	K_2_CO_3_	toluene	83
**14^[f]^**	**K_2_CO_3_**	**toluene**	**92**
15	–	toluene	0
16^[g]^	K_2_CO_3_	toluene	66
17^[h]^	K_2_CO_3_	toluene	25

[a] Conditions: **1 a** (0.44 mmol), **2 a** (0.4 mmol), base (1.0 equiv), degassed and dried solvent (3 mL), 60 °C, 36 h, under argon. [b] Yields were determined by GC‐MS analysis vs. a calibrated internal standard and are averages of two runs. [c] Room temperature. [d] Under air. [e] K_2_CO_3_ (3 equiv). [f] K_2_CO_3_ (0.8 equiv). [g] K_2_CO_3_ (0.8 equiv), degassed wet toluene. [h] K_2_CO_3_ (0.8 equiv), wet toluene. Moisture and air are detrimental to the yield due to the instability of the fluorinated aryl boronate.^[23g]^

Using these optimized conditions, we evaluated the scope and the limitations of this reaction. As shown in Table 2, a series of aldehydes bearing electron‐withdrawing or ‐donating substituents at the *para*‐, *meta*‐, or *ortho*‐position all worked well with pentafluorophenyl‐Bpin to give the desired products (**3 b**–**3 k**). Notably, for reactions employing aldehydes bearing electron‐donating groups, increasing the reaction temperature to 80 °C for 48 hours was required to generate the corresponding products in acceptable yields. It should be noted that reactions using 4‐(diethoxymethyl)benzaldehyde resulted in cleavage of the diethoxymethyl group yielding **3 l**. Furthermore, this methodology could be successfully extended to more complex aldehydes, such as those incorporating naphthyl and pyridyl groups (**3 m** and **3 n**). The structures of compounds **3 f**, **3 l**, **3 m** and **3 n** were unambiguously confirmed via single crystal X‐ray analysis (vide infra). After a broad range of aromatic aldehydes were examined, reactions with aliphatic aldehydes were investigated using the optimized conditions. Gratifyingly, all reactions proceeded smoothly to afford the corresponding products (**3 o**–**3 q**). Importantly, aldehydes containing ester groups, which are well‐known to be sensitive towards Grignard reagents, also afforded the desired alcohols in excellent yield (**3 r**).

**Table 2:** Scope of the reaction with respect to the different aldehyde substrates **2**.^[a,b]^ 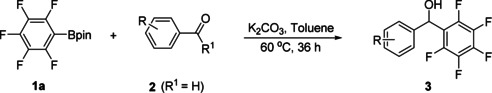

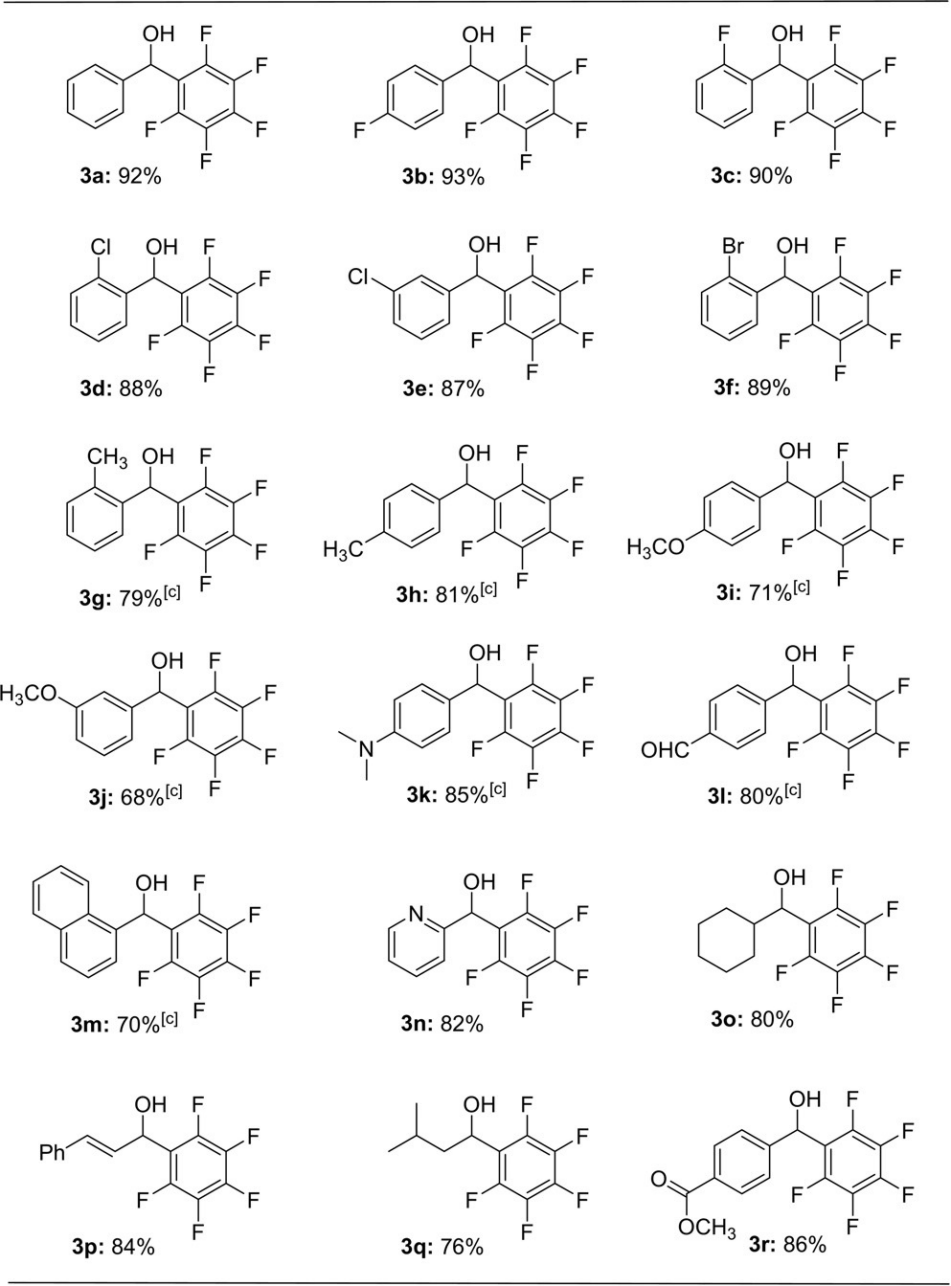

[a] Conditions: **1 a** (0.44 mmol), **2** (0.4 mmol), K_2_CO_3_ (0.32 mmol), toluene (3 mL), 60 °C, 36 h, Ar. [b] Isolated yields are reported. [c] 80 °C, 48 h.

We then briefly investigated the scope using simple ketones (Table 3). When reactions were performed at 120 °C and for prolonged reaction times, the corresponding products were provided in moderate yields (**3 s**–**3 u**). Modest reaction yields were obtained when sterically hindered benzophenone and (2‐fluorophenyl)(phenyl)methanone were used (**3 v**–**3 w**). Importantly, cyclohexanone proceeded to give the desired products in good yield (**3 x**).

**Table 3:** Scope of the reaction with respect to the different ketone substrates **2**.^[a,b]^ 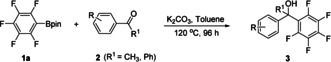

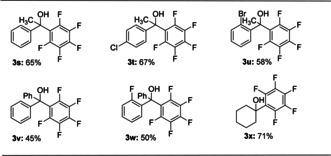

[a] Conditions: **1 a** (0.44 mmol), **2** (0.4 mmol), K_2_CO_3_ (0.32 mmol), toluene (3 mL), 120 °C, 96 h, Ar. [b] Isolated yields are reported.

To explore further the utility of this reaction, we then examined the scope using other less substituted polyfluorophenyl boronate esters with benzaldehyde (Table 4). The compounds 2,3,5,6‐tetrafluorophenyl‐Bpin, 2,3,4,6‐tetrafluorophenyl‐Bpin, and 2,4,6‐trifluorophenyl‐Bpin also proved to be effective in these reactions and afforded the products in excellent yields (**4 a**–**4 c**). Furthermore, the reaction with 2,6‐difluorophenyl‐Bpin proceeded to give the desired product in 80 % yield (**4 d**). However, these reaction conditions were not suitable for the reaction of 2,5‐difluorophenyl‐Bpin and 2‐fluorophenyl‐Bpin with benzaldehyde. Surprisingly, reactions with these substrates resulted in the formation of ketones (**4 e** and **4 f**) when a strong base was used. Tetrafluorophenyl‐Bpin reacted readily with acetophenone to yield product **4 g**. Unfortunately, no reaction occurred when the aryl‐Bpin compound did not have an *ortho*‐fluorine substituent (**4 h** and **4 i**), as 3‐fluorophenyl‐Bpin, phenyl‐Bpin, 4‐CH_3_‐phenyl‐Bpin and 4‐CN‐phenyl‐Bpin all failed to provide any product. These results demonstrate that the *ortho*‐fluorine group plays a key role in related conversions.

**Table 4:** Scope of the reaction with respect to different polyfluorophenyl boronate substrates **1**.^[a,b]^ 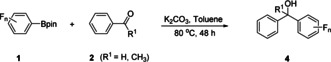

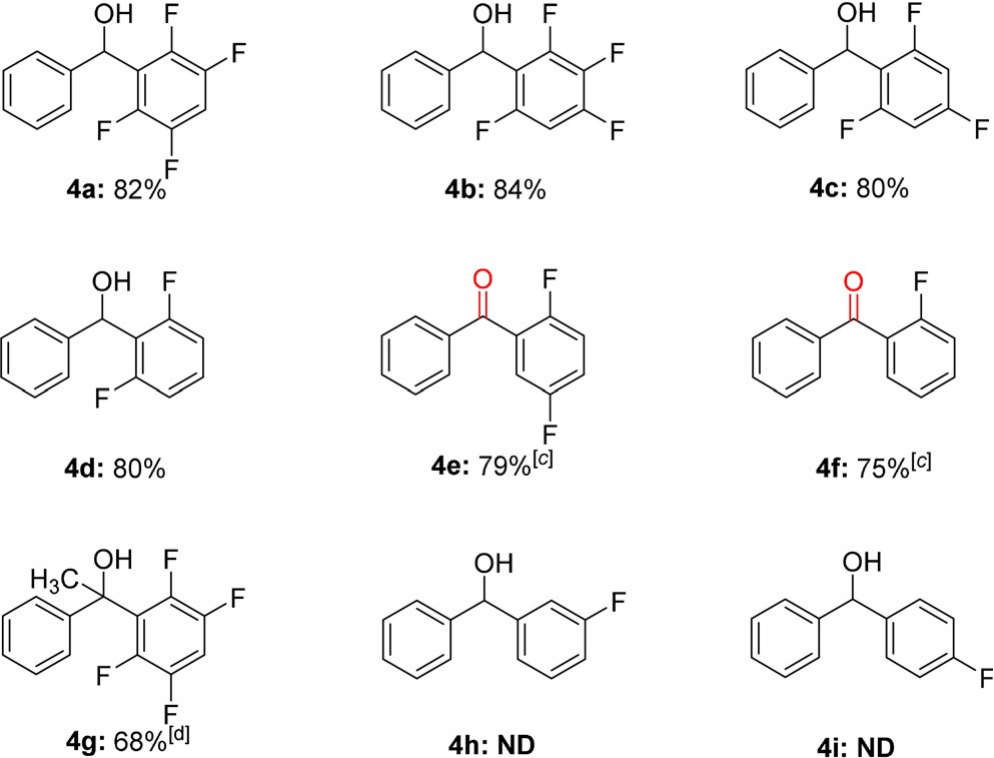

[a] Reaction conditions: **1 a** (0.44 mmol), **2** (0.4 mmol), K_2_CO_3_ (0.32 mmol), toluene (3 mL), 80 °C, 48 h, Ar. [b] Isolated yields are reported. [c] *t*‐BuOLi (0.32 mmol). [d] 120 °C, 96 h.

**Scheme 2:**
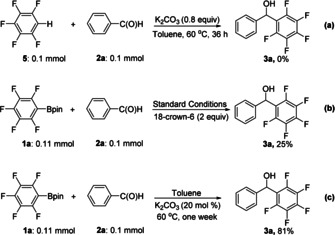
Preliminary mechanistic studies.

To gain further insight into the aforementioned reactions, several mechanistic studies were conducted. The reaction of **2 a** with pentafluorobenzene **5** under standard conditions was examined, yet **3 a** was not formed in any detectable amounts (Scheme 2 a), indicating that the C‐Bpin moiety is essential and deprotonation of the fluoroarene or nucleophilic attack at the fluoroarene by the base is not a plausible pathway. Interestingly, for the standard reaction between **1 a** and **2 a**, the yield dropped dramatically if 18‐crown‐6 ether and K_2_CO_3_ were added (Scheme 2 b). This experimental result indicates that the presence of the potassium ion plays a crucial role for the outcome of the reaction. Furthermore, if the reaction of **1 a** and **2 a** was performed in the presence of only a catalytic amount of K_2_CO_3_ (20 mol %) (Scheme 2 c), reaction rates were reduced, and a week was required to produce **3 a** in good, isolated yield. This finding again indicates that the potassium ion (or the base) plays an important role in the reaction. Substituting *ortho*‐fluorines by *ortho*‐chlorines, using either C_6_Cl_5_Bpin or 2,6‐dichlorophenyl‐1‐Bpin as substrates, did not yield any product as shown by in situ GCMS studies. Likewise, 2,3,4‐trifluorophenylBpin and 3,4,5‐trifluorophenylBpin substrates with only one or no *ortho*‐fluorine substituent also led to no detectable product formation. The presence of an *ortho*‐methoxy group on the aldehyde, however, did not inhibit the reaction.

**Scheme 3:**
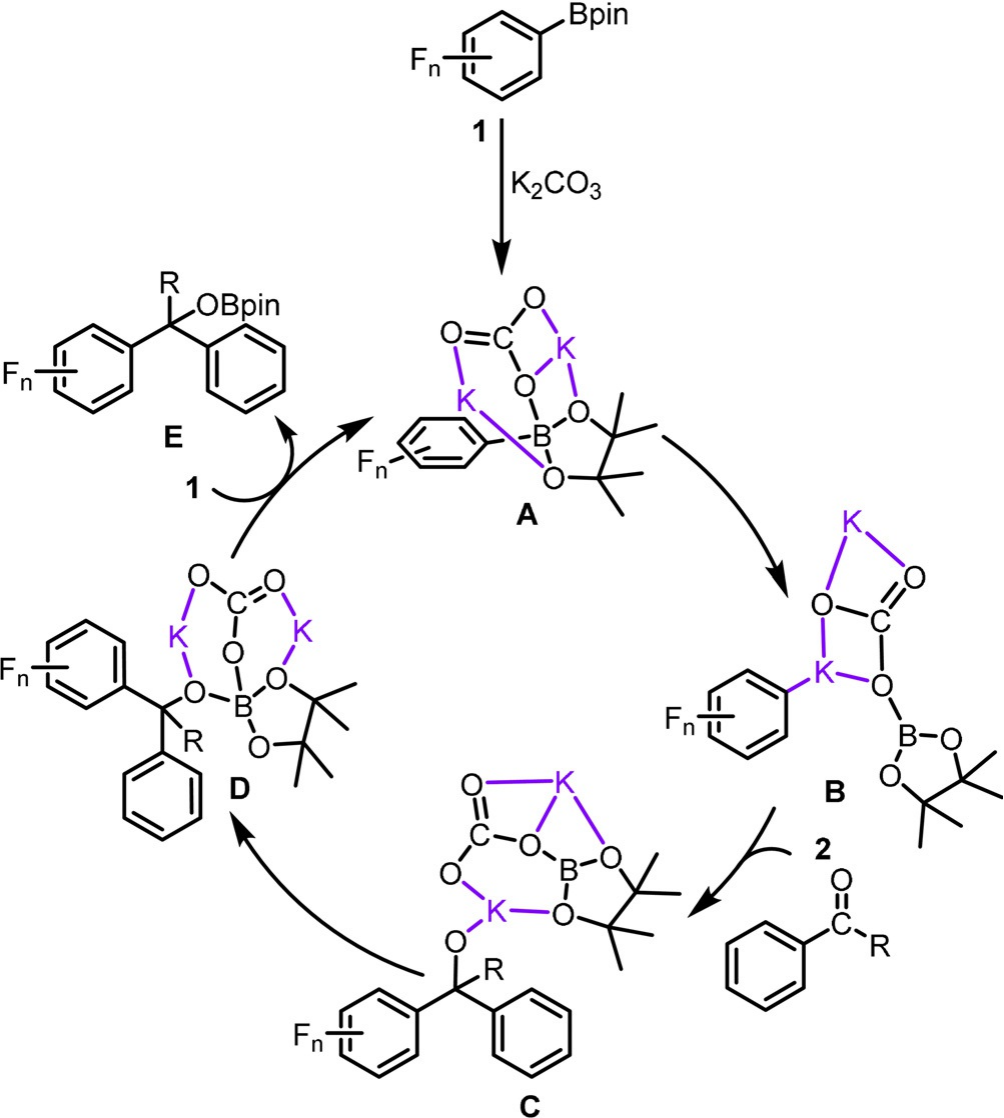
Proposed mechanism of the 1,2‐addition of polyfluorophenylboronates to aldehyde derivatives in the presence of K_2_CO_3_ as base.

Based on previous studies[^28^, ^29^] and experimental observations, a mechanism for the 1,2‐addition of polyfluorophenylboronates to aryl aldehydes in the presence of K_2_CO_3_ as base is proposed, as shown in Scheme 3. K_2_CO_3_ interacts with the Lewis‐acidic Bpin moiety of substrate **1** to generate base adduct **A**, which weakens the carbon‐boron bond and ultimately cleaves the B−C bond along with attachment of a potassium cation to the aryl group. The resulting Ar_F_^−^ anion adduct **B** undergoes nucleophilic attack at the aldehyde carbon atom of substrate **2** to generate methanolate **C**. The methanolate oxygen atom then attacks the electrophilic Bpin group to obtain compound **D**. Transfer of K_2_CO_3_ from intermediate **D** to the boron atom of the more Lewis‐acidic polyfluorophenyl‐Bpin **1** finally closes the cycle and regenerates complex **A**. Thus, the primary reaction product is the O‐borylated addition product **E**, which was detected by HRMS and NMR spectroscopy for the perfluorinated derivative (Supporting Information, section VIII).

**Figure 1:**
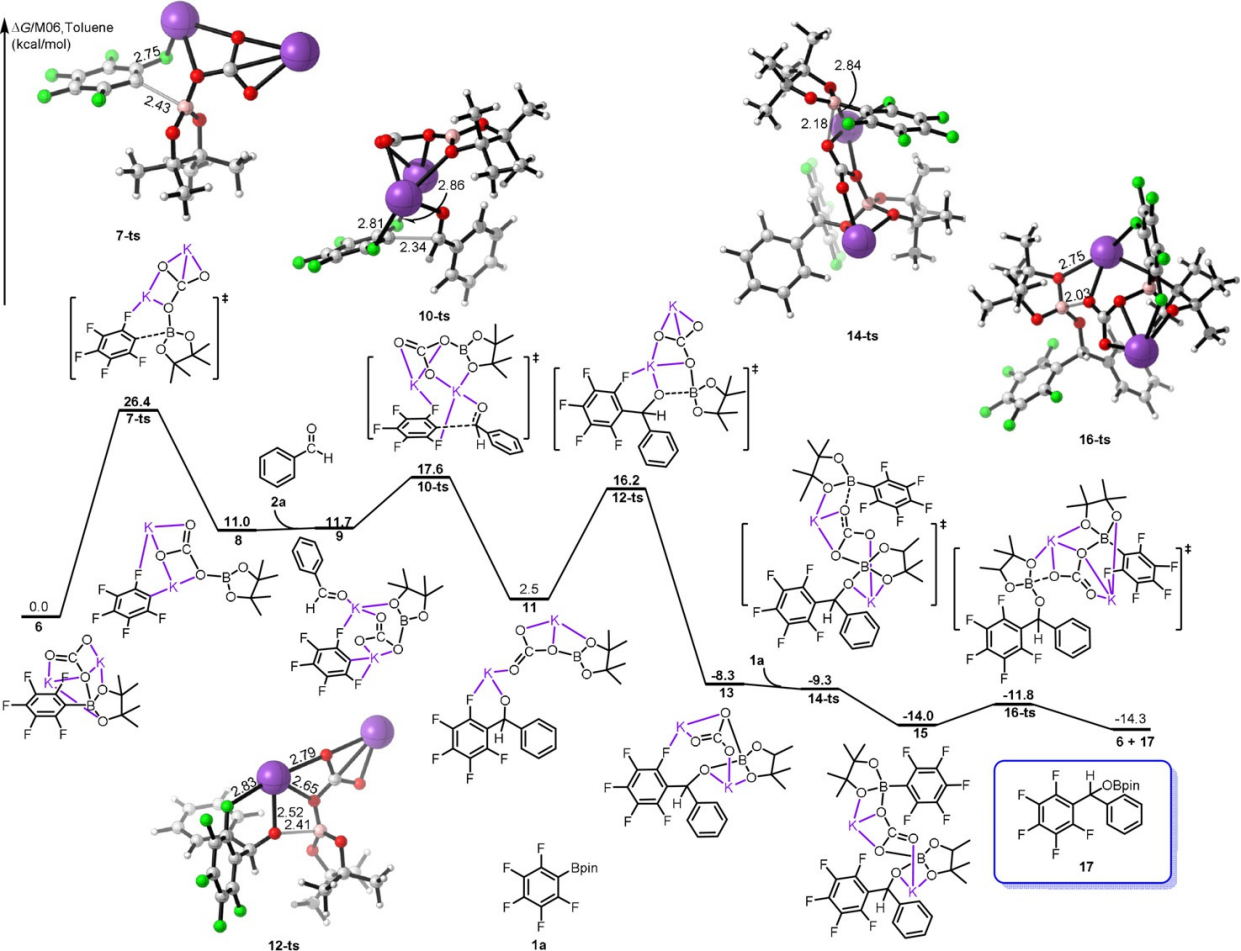
Free energy profile for the 1,2‐addition of pentafluorophenyl‐Bpin (**1 a**) and benzaldehyde (**2 a**) in the presence of K_2_CO_3_ as the base, calculated at the M06/(6–311++G(d, p), SMD)//B3LYP/(6–31+G(d)) level of theory. Relative free energies (Δ*G*) are given in kcal mol^−1^, and bond lengths are given in Å.

To corroborate this mechanism, a detailed DFT study was performed on the model 1,2‐addition of **1 a** to **2 a**, the results of which are shown in Figure 1. In the initial step, K_2_CO_3_ coordinates to the Bpin moiety of **1 a** and gives rise to the pentafluorophenyl‐Bpin‐base complex **6** with free energy decreasing by 27.2 kcal mol^−1^. The energy of compound **6** is set as the zero point of the energy profile. The pentafluorobenzene anion (Ar_F_^−^) adduct **8** is formed endothermically by cleavage of the B‐C(Ar_F_) bond via transition state **7‐ts** with an energy barrier of 26.4 kcal mol^−1^. In the optimized structures of **7‐ts**, K^+^ cations coordinate to C, O and F atoms, whereas there is only K‐O coordination in compound **6**. Subsequent cleavage of the B‐C(Ar_F_) bond can be facilitated by this pathway. The separated carbonate adduct and Ar_F_^−^ group in adduct **8** are connected and stabilized by K^+^ cations. Nucleophilic attack of Ar_F_^−^ at the aldehyde carbon atom via transition state **10‐ts** occurs to achieve the coupling intermediate **11** with an energy of 17.6 kcal mol^−1^. This low activation energy barrier can be attributed to the coordination of K^+^ to the oxygen atom of the aldehyde, thus enhancing the electrophilicity of the aldehyde carbon atom. Subsequently, the methanolate oxygen atom attacks the Lewis‐acidic boron atom to give the corresponding compound **13** irreversibly via transition state **12‐ts**. The overall energy barrier for this step is 16.2 kcal mol^−1^. Finally, K_2_CO_3_ in compound **13** coordinates to the boron of substrate **1 a** via transition state **14‐ts**, followed by cleavage of a B−O bond to give **16‐ts** and eventually **17**, regenerating the active species **6**. As shown in Figure 1, the energy barriers for these two steps are very low, indicating that intermediate **13** transforms to product **17** swiftly. The step from pentafluorophenyl‐Bpin‐base compound **6** to product **17** is calculated to be exergonic by 14.3 kcal mol^−1^. The base‐assisted cleavage of Bpin and pentafluorophenyl (Ar_F_) is calculated to be the rate determining step (RDS) with a free energy of activation of 26.4 kcal mol^−1^.

**Figure 2:**
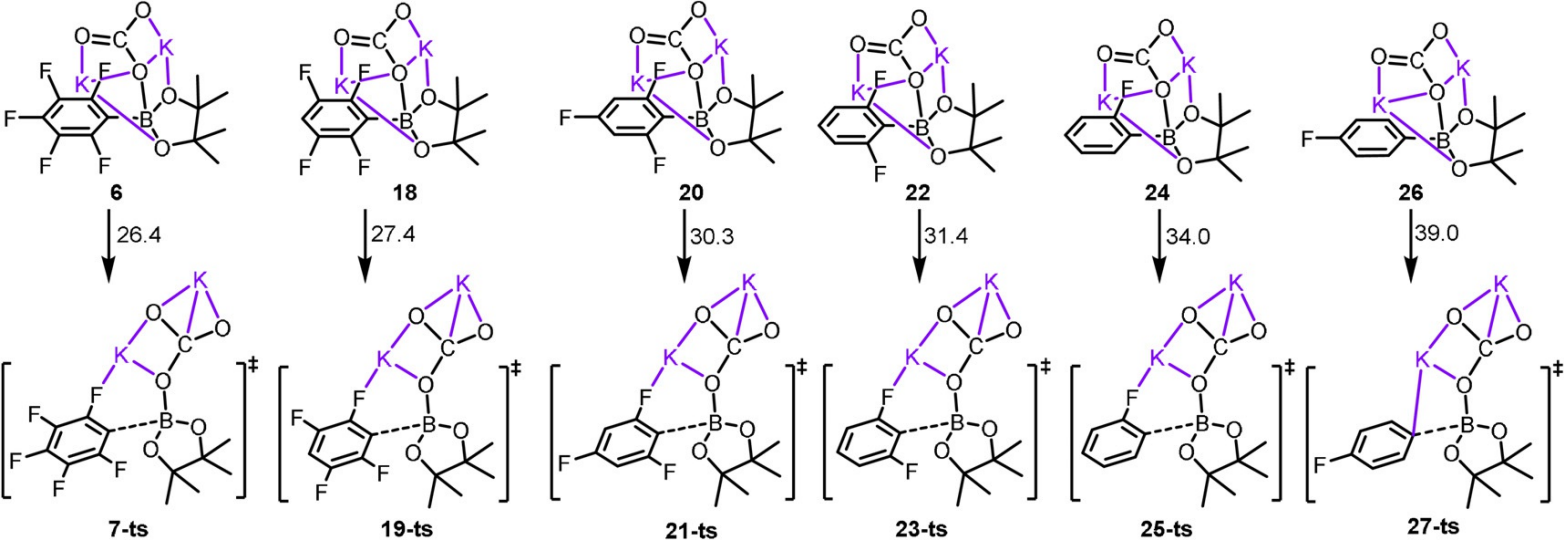
Free energies of activation of the cleavage of Bpin and Ar_F_ step calculated at the M06/(6–311++G(d, p), SMD)//B3LYP/(6–31+G(d)) level of theory. Relative free energies (Δ*G*) are given in kcal mol^−1^.

As shown in Figure 1, the cation K^+^ bonds with one or two F atoms in these intermediates and transition states, suggesting that the fluoride substituents possibly play an important role in the 1,2‐addition of polyfluorophenylboronates to aryl aldehydes. Therefore, we calculated the activation free energies of the RDS using polyfluorophenylboronates with different numbers and positions of fluorine substituents as the substrate. The results given in Figure 2 clearly show that the energy barrier rises with a reduction in the number of F substituents. The position of the fluorine atoms also affects the energy barrier, and *ortho* fluorine has a stronger effect on the barrier than F substituents at other positions. The barrier for **24**, with an *ortho*‐F substituent, is higher than that of **22** by 2.6 kcal mol^−1^, whereas that of **26** with a *para*‐F substituent rises to 39.0 kcal mol^−1^. In fact, no reaction was observed under these conditions when **26** was used as the substrate, which is consistent with our calculated results. We conclude that the *ortho*‐F substituent is vital in this reaction for interaction with K^+^ along the reaction pathway, and that other F substituents also influence the reactivity for the 1,2‐addition of polyfluorophenylboronates to aryl aldehydes via their electron‐withdrawing effect. Thus, stronger electron‐withdrawing groups located at the *para* or *meta* carbons of polyfluorophenylboronates may promote this reaction.

**Figure 3:**
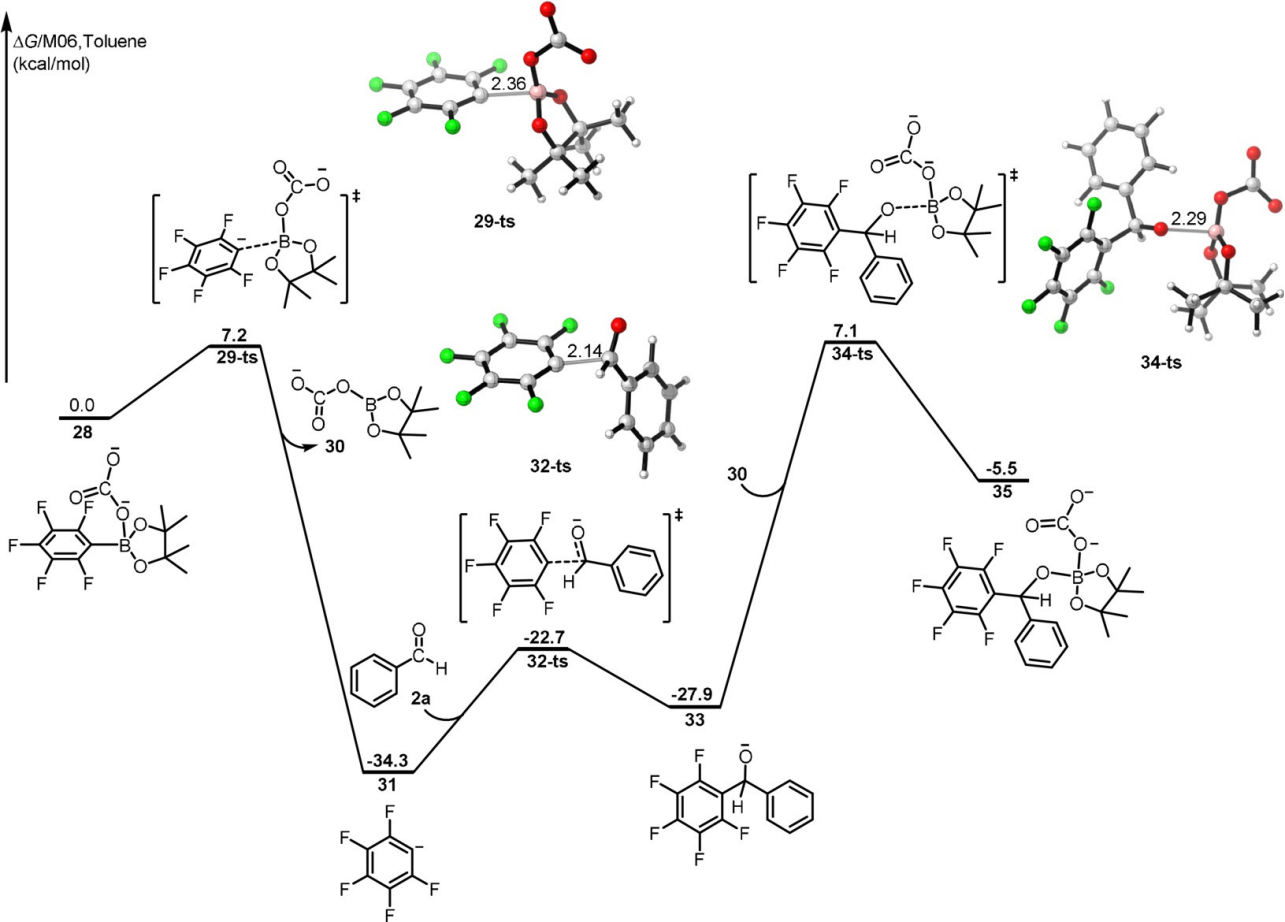
Free energy profile of 1,2‐addition of polyfluorophenylboronates with aryl aldehydes in the absence of K^+^ calculated by the M06/(6–311++G(d, p), SMD)//B3LYP/(6–31+G(d)) level of theory. Relative free energies (Δ*G*) are given in kcal mol^−1^, bond lengths are given in Å.

To ascertain the role of the K^+^ cation in these reactions, part of the free energy profile without the cation was also calculated at the same level of theory, and the results are given in Figure 3. Compared with the energy profile in Figure 1, in the absence of K^+^, the process of the methanolate oxygen anion **33** attack at the Lewis‐acidic boron in **30** becomes improbable, with an activation barrier of 41.4 kcal mol^−1^, although the initial cleavage of Bpin and pentafluorophenyl (Ar_F_) step has a lower free energy of activation. Upon addition of 18‐crown‐6 to the reaction, the yields drop dramatically. As a counterion, K^+^ clearly regulates the nucleophilicity of CO_3_^2−^, and promotes the reactivity by interaction with oxygen or fluorine atoms. Our DFT calculations indicate that both the *ortho*‐F substituents on the polyfluorophenylboronates and the counterion K^+^ are essential for the 1,2‐addition of polyfluorophenylboronates to aryl aldehydes.

**Figure 4:**
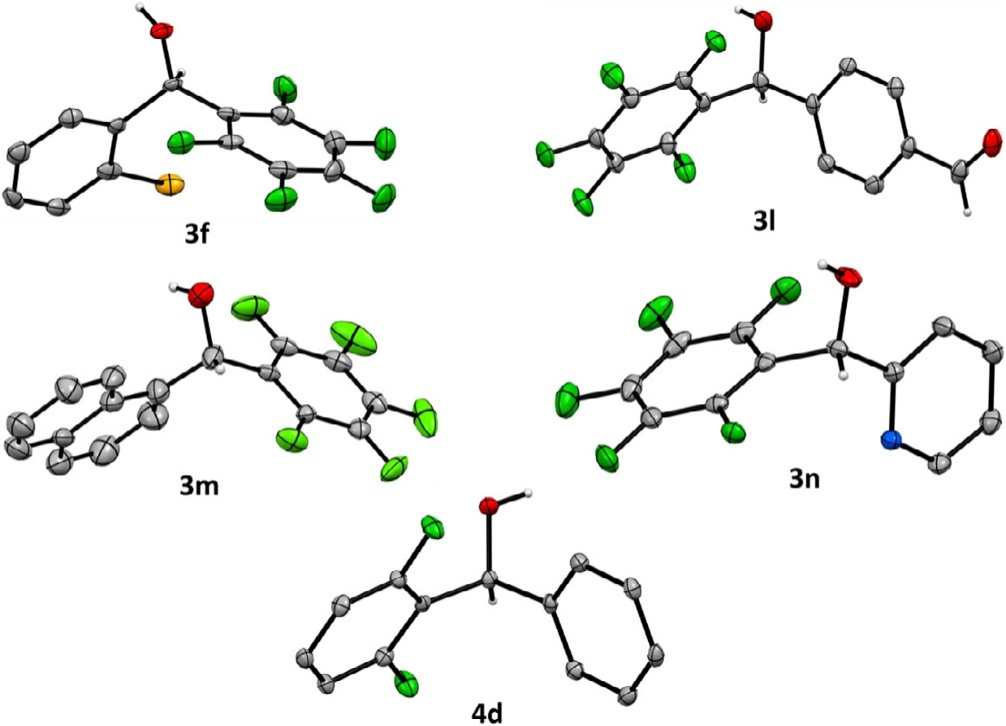
Molecular structures of compounds **3 f**, **3 l**, **3 m**, **3 n** and **4 d** in the solid state at 100 K. Atomic displacement ellipsoids are drawn with 50 % probability. Only selected hydrogen atoms are shown for clarity. C gray, O red, N blue, Br orange, F green, H white.^[36]^

**Figure 5:**
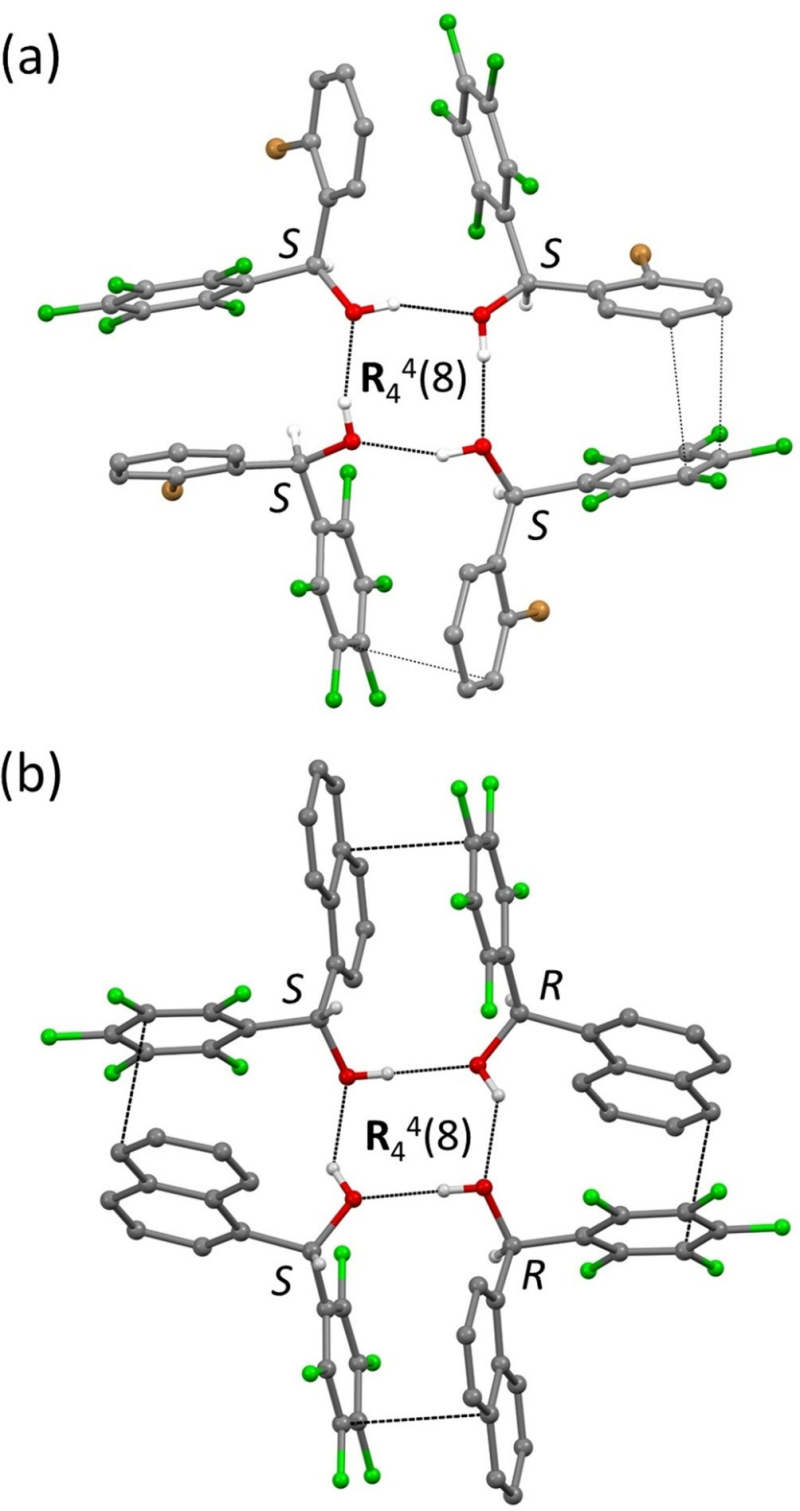
Compounds a) **3 f** and b) **3 m** self‐assemble to form tetramers via O−H⋅⋅⋅O hydrogen bonding and the corresponding graph set notation is R_4_^4^(8).^[31]^ π⋅⋅⋅π Stacking interactions between the bromophenyl or naphthyl and pentafluorophenyl groups, respectively, within the tetrameric unit are indicated by close C⋅⋅⋅C contacts (dashed lines). a) Each of the four symmetry‐independent tetramers of **3 f** consists of molecules of the same chirality (*RRRR* or *SSSS*). Only one tetramer (*SSSS*) is shown here. b) In **3 m**, the tetramer is centrosymmetric with (*RRSS*) chirality of the molecules.

The structures of **3 f**, **3 l**, **3 m**, **3 n**, and **4 d** were unambiguously confirmed by single crystal X‐ray diffraction. While the molecular structures are chiral (Figure 4), all the compounds represent racemic mixtures. Due to the presence of OH groups, the arrangement of the molecules in the crystal structures of all compounds is primarily determined by O−H⋅⋅⋅O or O−H⋅⋅⋅N hydrogen bonding (Supporting Information, Table S2). The presence of π⋅⋅⋅π stacking interactions between pentafluorophenyl and bromophenyl or naphthyl moieties (**3 f** and **3 m**), respectively, is also observed in these examples (Figure 5, Table S3). Such an attractive interaction between arenes and perfluorinated arenes results from the different electronegativities of the hydrogen and fluorine atoms with respect to the carbon atoms of the aromatic rings and, hence, from opposite multipole moments of the aromatic groups. It is called the arene‐perfluoroarene interaction and can be applied as a supramolecular synthon in crystal engineering.^[30]^ This was previously confirmed by Marder and co‐workers, who have shown that this type of interaction leads to the formation of highly ordered π‐stacks of alternating arene and perfluoroarene molecules in co‐crystals of arenes and perfluoroarenes.[^30d^, ^31^]

In the crystal structures of compounds **3 f** and **3 m**, the combination of both O−H⋅⋅⋅O hydrogen bonding and arene‐perfluoroarene interaction leads to the intriguing formation of [O−H⋅⋅⋅]_4_ hydrogen‐bonded cyclic tetramers with graph set **R**_4_^4^(8) (Figure 5, Table S2).^[32]^ The molecules of the tetramer interact via arene‐perfluoroarene π⋅⋅⋅π stacking between the bromophenyl or naphthyl and pentafluorophenyl moieties on the outside of the cyclic [O−H⋅⋅⋅]_4_ ring. The interplanar separations (3.281(7)–3.687(14) Å) are typical for π⋅⋅⋅π stacking interactions[^30^, ^31^] and the angles between the interacting planes are 4.96(19)–16.8(3)° (Table S3). In the higher symmetry compound **3 m** (space group *P*2_1_/*c* with *Z′*=2, where *Z′* denotes the number of molecules in the asymmetric unit), arene‐perfluoroarene interactions are also present between the tetramers, in addition to C−H⋅⋅⋅π, C−H⋅⋅⋅F, and F⋅⋅⋅F interactions (Figure S6). Each tetramer of **3 m** is centrosymmetric and, hence, contains molecules of opposite chirality (*RRSS*), leading to a racemic mixture (Figure 5 b). Tetramers are arranged in sheets parallel to the $\vec{b},\vec{c}$ ‐plane (Figure S6). In contrast, compound **3 f** crystallizes in the non‐centrosymmetric space group *P*1. There are 16 symmetry‐independent molecules in the asymmetric unit (*Z′*=16) of **3 f**, which build up four symmetry‐independent hydrogen‐bonded cyclic tetramers (Figure S1). Each tetramer is constituted by molecules of the same chirality (*RRRR* or *SSSS*) (Figure 5 a). Thus, the chirality of the four tetramers in the asymmetric unit, i.e., (*RRRR*)(*SSSS*)(*RRRR*)(*SSSS*), leads to a racemic mixture, as shown in Figures 5 a, S1 and S3. Tetramers of mixed chirality are arranged in sheets parallel to the $\vec{b},\vec{c}$ ‐plane with bromine atoms all pointing up or down within the sheet (Figures S2 and S3). Parallel sheets face each other either with the bromine atoms or without. In fact, crystals of **3 f** represent one of the rare class of crystals for which *Z′*>1.[^33^, ^34^] While searching for a structure of higher symmetry, the cell parameters of **3 f** were also determined at 200 K. As this resulted in a similar triclinic unit‐cell metric as was observed at 100 K, the occurrence of a phase transition at temperatures between 100 K and 200 K is unlikely.

**Figure 6:**
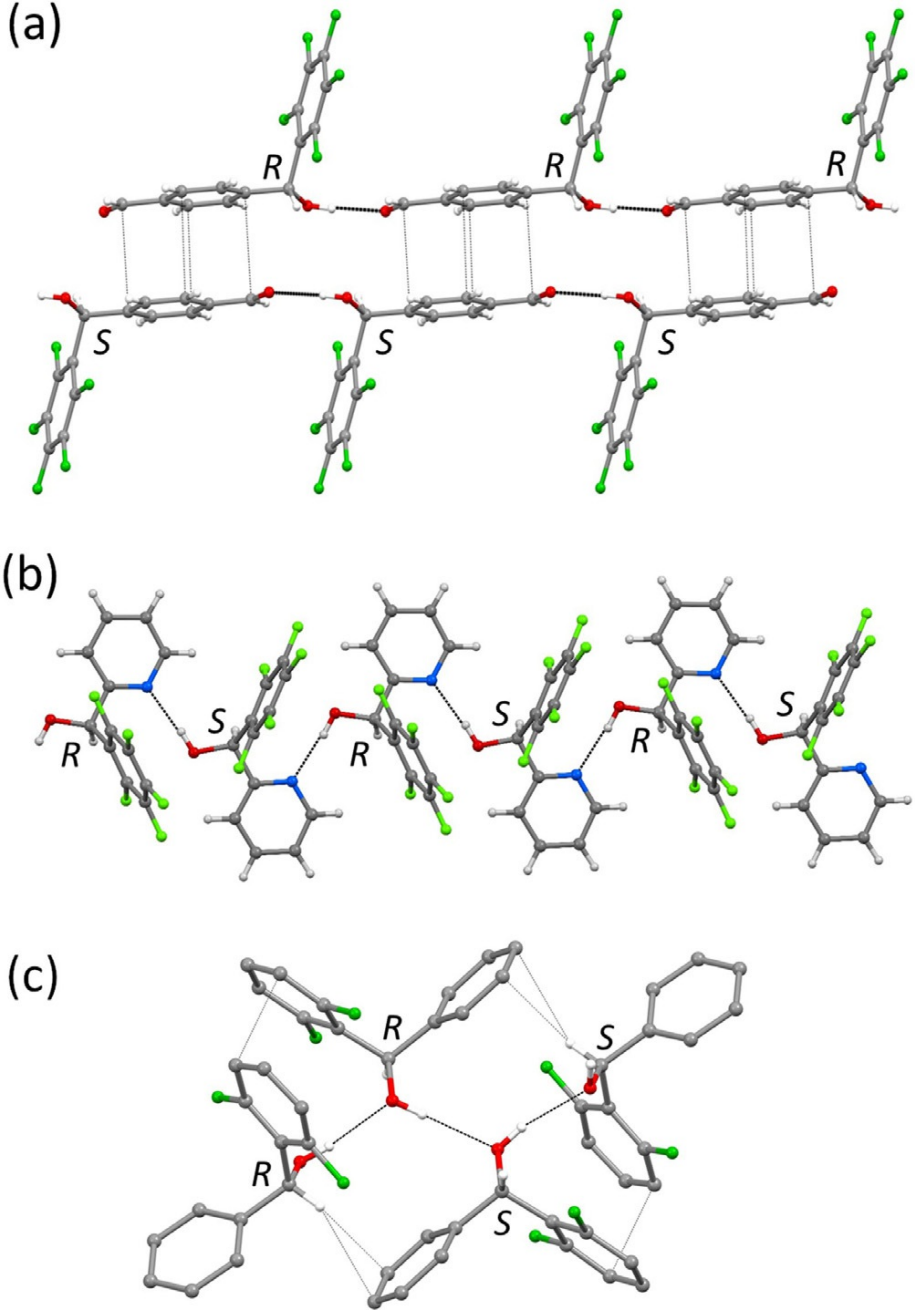
One‐dimensional hydrogen‐bonded chains are present in a) **3 l** (O−H⋅⋅⋅O), b) **3 n** (O−H⋅⋅⋅N), and c) **4 d** (O−H⋅⋅⋅O). a) In **3 l**, chains containing molecules of opposite chirality stack parallel via π⋅⋅⋅π interaction between the phenylcarboxaldehyde groups. b) A zig‐zag chain constituted by molecules of alternative chirality (*RSRS*…) is shown for compound **3 n**. c) Compound **4 d** exhibits corrugated chains with (*RRSS*…) chirality of the molecules. Additional weak interactions (C−H⋅⋅⋅π and partial π⋅⋅⋅π stacking) are shown.

Contrary to **3 f** and **3 m**, the dominance of hydrogen bonding and absence of arene‐perfluoroarene interactions in compounds **3 l** (space group $P\bar{1}$ ), **3 n** and **4 d** (both space group *C*2/*c*) resulted in the formation of one‐dimensional hydrogen‐bonded chains (Figure 6). In **3 l** and **3 n**, the intermolecular O−H⋅⋅⋅O and O−H⋅⋅⋅N hydrogen bonding interaction takes place between the alcohol (O−H, donor) and the carboxaldehyde (O, acceptor) and pyridyl (N, acceptor) groups, respectively, the latter having a stronger hydrogen bond acceptor ability compared to the alcohol group (Table S2). Depending on the position of the acceptor atom in the molecule, hydrogen‐bonded chains are straight (**3 l**, Figure 6 a) or zig‐zag‐like (**3 n**, Figure 6 b). In **3 l**, each one‐dimensional chain contains molecules of one particular chirality (either *R* or *S*), and chains of opposite chirality exhibit extensive π‐stacking interaction between the phenyl groups. In this way, double‐stranded linear chains projecting the C_6_F_5_ groups on both sides are formed, as shown in Figure 6 a. The C_6_F_5_ groups from neighboring strands undergo interdigitation and exhibit partial offset π⋅⋅⋅π interactions between fluorinated moieties and C−F⋅⋅⋅π interactions between phenyl and pentafluorophenyl groups (Figures S4 and S5, Table S3). In **3 n**, one‐dimensional zig‐zag chains are formed by molecules of alternating chirality (*RSRS*…) (Figure 6 b). The pyridyl rings lie coplanar and the pentafluorophenyl groups interdigitate via partial offset π⋅⋅⋅π interactions to form a parallel ribbon‐like arrangement (Figure S7, Table S3). This structure exhibits a bilayer architecture as there are alternating hydrophobic and hydrophilic regions (Figures S7 and S8).^[35]^ In **4 d**, corrugated one‐dimensional chains are observed by the intermolecular O−H⋅⋅⋅O−H⋅⋅⋅hydrogen bonding interactions between the alcohol groups (Table S2), and molecules constituted of alternating pairs of same chirality (*RRSSRRSS*… as shown in Figure 6 c and Figure S9). Other intermolecular interactions observed in **4 d** include C−H⋅⋅⋅F, C−H⋅⋅⋅π, and very weak, strongly offset π⋅⋅⋅π interactions (Table S3).

## Conclusion

We have demonstrated here the simple conditions for the 1,2‐addition of aldehydes and ketones with polyfluorophenylboronate compounds. This strategy has the following advantages: 1) transition metal‐free catalyst system; 2) a variety of aromatic and aliphatic aldehydes were found to be suitable substrates for this reaction using pentafluorophenyl‐Bpin in moderate to excellent yields; and 3) sterically hindered ketones also worked well to furnish the corresponding products. This method also introduces the use of polyfluorophenyl‐Bpin compounds instead of Grignard reagents for polyfluorophenylation of arylaldehyde and ketone substrates. Further studies of the synthesis and applications of polyfluorophenyl boronates are underway in our laboratory and will be reported in due course.

## Conflict of interest

The authors declare no conflict of interest.

## Supporting information

As a service to our authors and readers, this journal provides supporting information supplied by the authors. Such materials are peer reviewed and may be re‐organized for online delivery, but are not copy‐edited or typeset. Technical support issues arising from supporting information (other than missing files) should be addressed to the authors.

Supplementary
