# Supplementary material for: Transition Metal Catalyst‐Free, Base‐Promoted 1,2‐Additions of Polyfluorophenylboronates to Aldehydes and Ketones
Source: Angew Chem Int Ed Engl. 2021 Jun 17;60(30):16529–38. doi: 10.1002/anie.202103686 (PMC8362073; doi:10.1002/anie.202103686)
Supplement: Supplementary file 1 — Supplementary [file ANIE-60-16529-s001.pdf]

Angewandte Chemie
Eine Zeitschrift der Gesellschaft Deutscher Chemiker

Supporting Information

Transition Metal Catalyst-Free, Base-Promoted 1,2-Additions of Polyfluorophenylboronates to Aldehydes and Ketones

Zhiqiang Liu, Goutam Kumar Kole, Yudha P. Budiman, Ya-Ming Tian, Alexandra Friedrich, Xiaoling Luo, Stephen A. Westcott, Udo Radius,* and Todd B. Marder**

anie_202103686_sm_miscellaneous_information.pdf

Table of Contents

I. General Information S3

II. Borylation of Polyfluoroarenes S3

III. General Procedures S4

IV. Characterization S4-S15

V. ^{1}H NMR, ^{19}F NMR and ^{13}C NMR Spectra S16

VI. Single Crystal X-ray Diffraction S63-S74

VII. Computational Methods S75-S107

VIII. Investigation of the Reaction Mechanism...S108-S111

IX. References.. S112-113

I. General Information

All NMR spectra were recorded on a Bruker AC–500 spectrometer (500 MHz for ^{1}H NMR, 125 MHz for $^{13}C\{^{1}H\}$ NMR, and 470 MHz for ^{19}F NMR) with $CDCl_3$ as the solvent. Chemical shifts (δ) are given in ppm and ^{1}H NMR spectra were referenced via residual proton resonances of $CDCl_3$ (7.26 ppm), $^{13}C\{^{1}H\}$ spectra were referenced to $CDCl_3$ (77.16 ppm) and ^{19}F spectra are referenced to external $CFCl_3$. The following abbreviations were used to indicate multiplicities: s = singlet; d = doublet; t = triplet; q = quartet; m = multiplet. GCMS analyses were performed on an Agilent Technologies GCMS system (GC 7890A, EI-MS 5975C). HRMS were measured on a Thermo Scientific Exactive Plus equipped with an Orbitrap. ESI measurements were conducted using a HESI source with an aux-gas temperature of 50 °C. Measurements were conducted using an APCI source with a corona needle; aux-gas temperature was 400 °C. Chemical yields referred to pure isolated product. Automated flash chromatography was performed on silica gel (Biotage SNAP cartridge KP-Sil), obtained from Biotage, using a Biotage® Isolera Four Flash system. Unless otherwise stated, all reagents were commercially purchased and used without further purification. The degassed and dry solvents were used. B_2pin_2 was kindly provided by AllyChem Co. Ltd. (Dalian, China).

II. Borylation of Polyfluoroarenes

Pentafluorophenyl-Bpin, 2,3,5,6-tetrafluorophenyl-Bpin, 2,3,4,6-tetrafluorophenyl-Bpin and 2,4,6-trifluorophenyl-Bpin used were prepared according to the literature procedures.[1] In an argon filled glovebox, a solution of $[(COD)Ir(OMe)]_2$ (0.5 mol%), 4,4'-di-tert-butyl-2,2'-bipyridine (2 mol%), bispinacolatodiboron (B_2pin_2) (0.5 equiv.) and pentafluoroarene (1 equiv.) in hexane (dry and degassed) was stirred at room temperature in a sealed reaction vessel for 48h. The volatile materials were removed *in vacuo* to give the crude product, together with unreacted starting arene. The residue was then purified by flash chromatography on silica gel to provide the corresponding product (~90 %).

III. General Procedures

In an argon filled glovebox, a sealable reaction tube with a cap equipped with a magnetic stir bar was charged with polyfluorophenyl boronate esters **1** (0.45 mmol), aldehydes **2** (0.4 mmol) and K_2CO_3 (62.1 mg, 0.36 mmol) in toluene (3.0 mL, SPS and degassed) at room temperature. The sealed reaction vessel was placed in an oil bath at 60 °C for 36 h. After the reaction was completed, it was cooled to room temperature. The solvent was removed *in vacuo*. The residue was purified by flash chromatography on silica gel (eluant: *n*-pentane and EtOAc) to give the desired product.

IV. Characterization

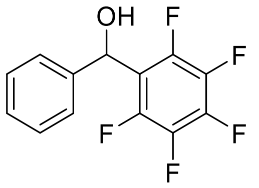

(**3a**):[2] 101 mg, 92% yield, white solid. ^{1}H NMR (500 MHz, $CDCl_3$): δ (ppm) = 7.40-7.36 (m, 4H), 7.34-7.31 (m, 1H), 6.23 (s, 1H), 2.92 (s, 1H). ^{13}C{^{1}H} NMR (125 MHz, $CDCl_3$): δ (ppm) = 144.6 (dm, $^1J_{F\text{-}C}$ = 250 Hz), 140.8 (dm, $^1J_{F\text{-}C}$ = 250 Hz), 140.6, 137.6 (dm, $^1J_{F\text{-}C}$ = 250 Hz), 128.7, 128.3, 125.4 (t, $J_{F\text{-}C}$ = 1 Hz), 117.0 (tm, $J_{F\text{-}C}$ = 17 Hz), 67.6. ^{19}F NMR (470 MHz, $CDCl_3$): δ (ppm) = -143 – -143.1 (m, 2F), -154.6 – -154.7 (m, 1F), -161.4 – -161.6 (m, 2F). HRMS (ASAP): Calcd. for $C_{13}H_7F_5O$ 274.0417, Found: 274.0403.

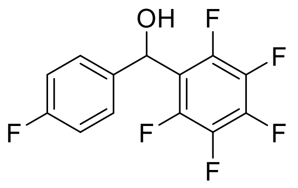

(**3b**):[2] 109 mg, 93% yield, colorless oil. ^{1}H NMR (500 MHz, $CDCl_3$): δ (ppm) = 7.38-7.34 (m, 2H), 7.07-7.02 (m, 2H), 6.21 (s, 1H), 2.73 (s, 1H). ^{13}C{^{1}H} NMR (125 MHz, $CDCl_3$): δ (ppm) = 162.5 (d, $^1J_{F\text{-}C}$ = 250 Hz), 144.6 (dm, $^1J_{F\text{-}C}$ = 250 Hz), 140.9 (dm, $^1J_{F\text{-}C}$ = 250 Hz), 137.7 (dm, $^1J_{F\text{-}C}$ = 250 Hz), 136.3 (d, $J_{F\text{-}C}$ = 3 Hz), 127.2 (m), 116.7 (tm, $J_{F\text{-}C}$ = 18 Hz), 115.6 (d, $J_{F\text{-}C}$ = 22 Hz), 66.7. ^{19}F NMR (470 MHz, $CDCl_3$): δ (ppm) = -113.7 – -113.8 (m, 1F),

-143.1 – -143.2 (m, 2F), -154.2 – -154.3 (m, 1F), -161.2 – -161.3 (m, 2F). HRMS (ASAP): Calcd. for $C_{13}H_6F_6O$ 292.0323, Found: 292.0312.

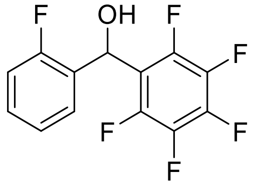

(**3c**): 105 mg, 90% yield, colorless oil. ^{1}H NMR (500 MHz, $CDCl_3$): δ (ppm) = 7.69-7.65 (m, 1H), 7.33-7.29 (m, 1H), 7.22-7.19 (m, 1H), 7.02-6.98 (m, 1H), 6.42 (s, 1H), 3.04 (s, 1H). ^{13}C{^{1}H} NMR (125 MHz, $CDCl_3$): δ (ppm) = 159.4 (d, $^1J_{F\text{-}C}$ = 250 Hz), 144.8 (dm, $^1J_{F\text{-}C}$ = 250 Hz), 141.0 (dm, $^1J_{F\text{-}C}$ = 250 Hz), 137.6 (dm, $^1J_{F\text{-}C}$ = 250 Hz), 129.9 (d, $J_{F\text{-}C}$ = 8 Hz), 127.4 (d, $J_{F\text{-}C}$ = 13 Hz), 126.9 (m), 124.2 (d, $J_{F\text{-}C}$ = 4 Hz), 115.7 (tm, $J_{F\text{-}C}$ = 15 Hz), 115.3 (d, $J_{F\text{-}C}$ = 21 Hz), 62.0. ^{19}F NMR (470 MHz, $CDCl_3$): δ (ppm) = -117.8 – -117.9 (m, 1F), -142.8 – -142.9 (m, 2F), -154.4 – -154.5 (m, 1F), -161.7 – -161.9 (m, 2F). HRMS (ASAP): Calcd. for $C_{13}H_6F_6O$ 292.0323, Found: 292.0311.

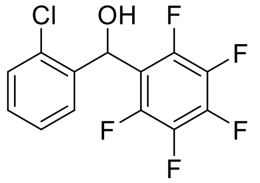

(**3d**):[2] 108 mg, 88% yield, white solid. ^{1}H NMR (500 MHz, $CDCl_3$): δ (ppm) = 7.81-7.79 (m, 1H), 7.36-7.27 (m, 3H), 6.36 (s, 1H), 3.32 (s, 1H). ^{13}C{^{1}H} NMR (125 MHz, $CDCl_3$): δ (ppm) = 145.1 (dm, $^1J_{F\text{-}C}$ = 250 Hz), 141.1 (dm, $^1J_{F\text{-}C}$ = 250 Hz), 137.6 (dm, $^1J_{F\text{-}C}$ = 250 Hz), 137.4, 131.7, 129.5, 129.4, 127.5 (t, $J_{F\text{-}C}$ = 3 Hz), 126.8, 115.0 (tm, $J_{F\text{-}C}$ = 17 Hz), 64.4. ^{19}F NMR (470 MHz, $CDCl_3$): δ (ppm) = -141.6 – -141.7 (m, 2F), -153.9 (t, J_F = 21 Hz, 1F), -161.7 – -161.9 (m, 2F). HRMS (ASAP): Calcd. for $C_{13}H_6F_5ClO$ 308.0027, Found: 308.0014.

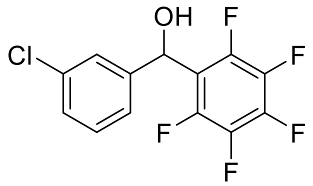

(**3e**): 108 mg, 87% yield, colorless oil. ^{1}H NMR (500 MHz, $CDCl_3$): δ (ppm) = 7.39 (s, 1H), 7.32-7.27 (m, 2H), 7.25-7.23 (m, 1H), 6.19 (s, 1H), 2.92 (s, 1H). ^{13}C{^{1}H} NMR (125 MHz, $CDCl_3$): δ (ppm) = 144.7 (dm, $^1J_{F\text{-}C}$ = 250 Hz), 142.5, 141.1 (dm, $^1J_{F\text{-}C}$ = 250 Hz), 137.7 (dm,

$^{1}J_{F\text{-}C}$ = 250 Hz), 134.7, 130.0, 128.4, 125.6, 123.5, 116.3 (tm, $J_{F\text{-}C}$ = 15 Hz), 66.7. ^{19}F NMR (470 MHz, $CDCl_3$): δ (ppm) = -142.8 – -142.9 (m, 2F), -153.7 – -153.8 (m, 1F), -160.9 – -161.1 (m, 2F). HRMS (ASAP): Calcd. for $C_{13}H_6F_5ClO$ 308.0027, Found: 308.0017.

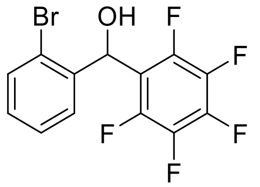

(**3f**): 125 mg, 89% yield, white solid. ^{1}H NMR (500 MHz, $CDCl_3$): δ (ppm) = 7.80-7.78 (m, 1H), 7.51 (dd, J = 8 Hz, 1 Hz, 1H), 7.39 (tm, J = 8 Hz, 1H), 7.20 (tm, J = 8 Hz, 1H), 6.29 (s, 1H), 3.24 (s, 1H). ^{13}C{^{1}H} NMR (125 MHz, $CDCl_3$): δ (ppm) = 145.1 (dm, $^{1}J_{F\text{-}C}$ = 250 Hz), 143.2 (dm, $^{1}J_{F\text{-}C}$ = 250 Hz), 138.9, 137.6 (dm, $^{1}J_{F\text{-}C}$ = 250 Hz), 132.8, 129.8, 128.1 (t, $J_{F\text{-}C}$ = 3 Hz), 127.4, 121.5, 114.9 (tm, $J_{F\text{-}C}$ = 15 Hz), 66.5. ^{19}F NMR (470 MHz, $CDCl_3$): δ (ppm) = -141.0 (m, 2F), -153.7 (t, J_F = 20 Hz, 1F), -161.6 – -161.8 (m, 2F). HRMS (ASAP): Calcd. for $C_{13}H_6F_5BrO$ 351.9522, Found: 351.9511.

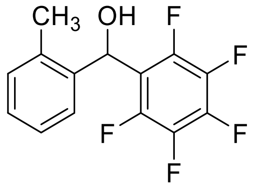

(**3g**):[2] 91 mg, 79% yield, white solid. ^{1}H NMR (500 MHz, $CDCl_3$): δ (ppm) = 7.54-7.52 (m, 1H), 7.27-7.24 (m, 2H), 7.19-7.17 (m, 1H), 6.30 (s, 1H), 2.63 (s, 1H), 2.31 (s, 3H). ^{13}C{^{1}H} NMR (125 MHz, $CDCl_3$): δ (ppm) = 144.8 (dm, $^{1}J_{F\text{-}C}$ = 250 Hz), 140.8 (dm, $^{1}J_{F\text{-}C}$ = 250 Hz), 137.9, 137.6 (dm, $^{1}J_{F\text{-}C}$ = 250 Hz), 135.4, 130.8, 128.4, 126.2, 125.7 (t, $J_{F\text{-}C}$ = 3 Hz), 115.8 (tm, $J_{F\text{-}C}$ = 15 Hz), 65.3, 18.9. ^{19}F NMR (470 MHz, $CDCl_3$): δ (ppm) = -142.0 – -142.1 (m), -154.4 – -154.5 (m), -161.5 – -161.7 (m, 2F). HRMS (ASAP): Calcd. for $C_{14}H_9F_5O$ 288.0574, Found: 288.0563.

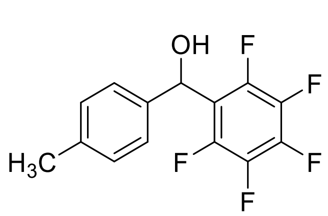

(**3h**):[2] 93 mg, 81% yield, white solid. ^{1}H NMR (500 MHz, $CDCl_3$): δ (ppm) = 7.28-7.27 (m, 2H), 7.20-7.18 (m, 2H), 6.17 (s, 1H), 3.20 (s, 1H), 2.37 (s, 3H). ^{13}C{^{1}H} NMR (125 MHz,

CDCl$_3$): δ (ppm) = 144.6 (dm, $^1J_{F\text{-}C}$ = 250 Hz), 140.7 (dm, $^1J_{F\text{-}C}$ = 250 Hz), 138.2, 137.7 (dm, $^1J_{F\text{-}C}$ = 251 Hz), 137.6, 129.4, 125.3, 117.1 (tm, $J_{F\text{-}C}$ = 15 Hz), 67.5, 21.0. ^{19}F NMR (470 MHz, CDCl$_3$): δ (ppm) = -143.1 – -143.2 (m, 2F), -155.1 (t, J_F = 21 Hz, 1F), -161.7 – -161.8 (m, 2F). HRMS (ASAP): Calcd. for $C_{14}H_9F_5O$ 288.0574, Found: 288.0563.

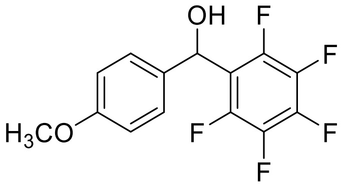

(**3i**):[2] 86 mg, 71% yield, colorless oil. ^{1}H NMR (500 MHz, CDCl$_3$): δ (ppm) = 7.30-7.28 (m, 2H), 6.88-6.85 (m, 2H), 6.14 (s, 1H), 3.79 (s, 3H), 3.24 (s, 1H). ^{13}C{^{1}H} NMR (125 MHz, CDCl$_3$): δ (ppm) = 159.5, 144.6 (dm, $^1J_{F\text{-}C}$ = 250 Hz), 140.7 (dm, $^1J_{F\text{-}C}$ = 250 Hz), 137.6 (dm, $^1J_{F\text{-}C}$ = 250 Hz), 132.7, 126.8, 117.2 (tm, $J_{F\text{-}C}$ = 15 Hz), 114.0, 67.3, 55.2. ^{19}F NMR (470 MHz, CDCl$_3$): δ (ppm) = -143.2 – -143.3 (m, 2F), -155.2 (t, J_F = 21 Hz, 1F), -161.7 – -161.8 (m, 2F). HRMS (ASAP): Calcd. for $C_{14}H_9F_5O_2$ 304.0523, Found: 304.0512.

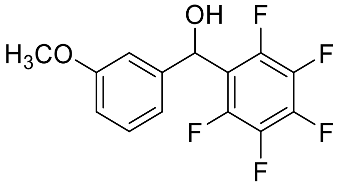

(**3j**):[2] 86 mg, 71% yield, colorless oil. ^{1}H NMR (500 MHz, CDCl$_3$): δ (ppm) = 7.28-7.25 (m, 1H), 6.97 (s, 1H), 6.92-6.91 (m, 1H), 6.85-6.83 (m, 1H), 6.17 (s, 1H), 3.80 (s, 3H), 3.01 (s, 1H). ^{13}C{^{1}H} NMR (125 MHz, CDCl$_3$): δ (ppm) = 159.9, 144.6 (dm, $^1J_{F\text{-}C}$ = 250 Hz), 142.3, 140.8 (dm, $^1J_{F\text{-}C}$ = 250 Hz), 137.6 (dm, $^1J_{F\text{-}C}$ = 250 Hz), 129.8, 117.6, 116.9 (tm, $J_{F\text{-}C}$ = 15 Hz), 113.3, 111.3, 67.3, 55.3. ^{19}F NMR (470 MHz, CDCl$_3$): δ (ppm) = -143.0 – -143.1 (m, 2F), -154.7 (t, J_F = 21 Hz, 1F), -161.5 – -161.6 (m, 2F). HRMS (ASAP): Calcd. for $C_{14}H_9F_5O_2$ 304.0523, Found: 304.0509.

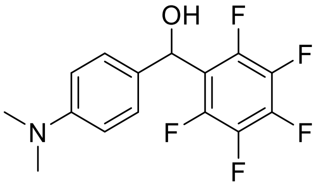

(**3k**): 108 mg, 85% yield, white solid. ^{1}H NMR (500 MHz, CDCl$_3$): δ (ppm) = 7.26-7.24 (m, 2H), 6.74-6.73 (m, 2H), 6.13 (s, 1H), 2.96 (s, 6H), 2.77 (s, 1H). ^{13}C{^{1}H} NMR (125 MHz,

$CDCl_3$): δ (ppm) = 150.2, 144.5 (dm, $^1J_{F\text{-}C}$ = 250 Hz), 140.5 (dm, $^1J_{F\text{-}C}$ = 250 Hz), 137.6 (dm, $^1J_{F\text{-}C}$ = 250 Hz), 128.7, 126.7, 117.4 (tm, $J_{F\text{-}C}$ = 15 Hz), 112.7, 67.9, 40.7. ^{19}F NMR (470 MHz, $CDCl_3$): δ (ppm) = -142.9 – -143.0 (m, 2F), -155.6 (s, 1F), -161.5 – -161.6 (m, 2F). HRMS (ASAP): Calcd. for $C_{15}H_{12}F_5NO$ $[M+H]^+$ 318.0912, Found: 318.0903.

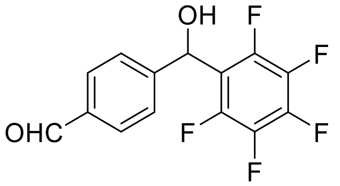

(**3l**): 97 mg, 80% yield, white solid. ^{1}H NMR (500 MHz, $CDCl_3$): δ (ppm) = 10.00 (s, 1H), 7.90-7.88 (m, 2H), 7.59-7.58 (m, 2H), 6.33 (s, 1H), 2.91 (s, 1H). $^{13}C\{^1H\}$ NMR (125 MHz, $CDCl_3$): δ (ppm) = 191.9, 147.2, 144.7 (dm, $^1J_{F\text{-}C}$ = 250 Hz), 141.2 (dm, $^1J_{F\text{-}C}$ = 250 Hz), 137.7 (dm, $^1J_{F\text{-}C}$ = 250 Hz), 136.0, 130.1, 126.0, 116.4 (tm, $J_{F\text{-}C}$ = 15 Hz), 66.7. ^{19}F NMR (470 MHz, $CDCl_3$): δ (ppm) = -142.6 – -142.7 (m, 2F), -153.3 (m, 1F), -160.7 – -160.8 (m, 2F). HRMS (ASAP): Calcd. for $C_{14}H_7F_5O_2$ $[M+H]^+$ 303.0439, Found: 303.0430.

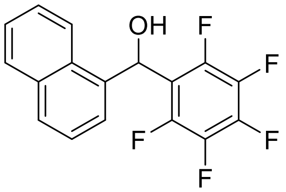

(**3m**):[2] 91 mg, 70% yield, white solid. ^{1}H NMR (500 MHz, $CDCl_3$): δ (ppm) = 8.04 (d, J = 9 Hz, 1H), 7.91-7.85 (m, 1H), 7.85 (d, J = 10 Hz, 1H), 7.61 (d, J = 8 Hz, 1H), 7.58-7.51 (m, 2H), 7.49-7.46 (m, 1H), 6.85 (s, 1H), 2.94 (s, 1H). $^{13}C\{^1H\}$ NMR (125 MHz, $CDCl_3$): δ (ppm) = 145.0 (dm, $^1J_{F\text{-}C}$ = 250 Hz), 141.9 (dm, $^1J_{F\text{-}C}$ = 250 Hz), 137.7 (dm, $^1J_{F\text{-}C}$ = 250 Hz), 134.9, 133.9, 130.3, 129.4, 129.0, 126.8, 125.9, 125.1, 123.9 (t, $J_{F\text{-}C}$ = 2 Hz), 122.7, 116.1 (tm, $J_{F\text{-}C}$ = 15 Hz), 65.2. ^{19}F NMR (470 MHz, $CDCl_3$): δ (ppm) = -142.0 – -142.1 (m, 2F), -154.2 (t, J_F = 21 Hz, 1F), -161.3 – -161.4 (m, 2F). HRMS (ASAP): Calcd. for $C_{17}H_9F_5O$ 324.0574, Found: 324.0561.

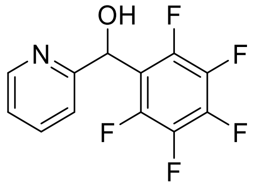

(**3n**): 90 mg, 82% yield, white solid. ^{1}H NMR (500 MHz, $CDCl_3$): δ (ppm) = 8.59 (d, J = 4 Hz, 1H), 7.72-7.69 (m, 1H), 7.30-7.28 (m, 1H), 7.16 (d, J = 8 Hz, 1H), 6.17 (s, 1H), 5.52 (s, 1H). $^{13}C\{^{1}H\}$ NMR (125 MHz, $CDCl_3$): δ (ppm) = 157.3, 147.9, 145.3 (dm, $^{1}J_{F\text{-}C}$ = 250 Hz), 141.1 (dm, $^{1}J_{F\text{-}C}$ = 250 Hz), 137.6 (dm, $^{1}J_{F\text{-}C}$ = 250 Hz), 137.3, 123.2, 120.4, 116.7 (tm, $J_{F\text{-}C}$ = 18 Hz), 65.5. ^{19}F NMR (470 MHz, $CDCl_3$): δ (ppm) = -143.3 – -143.4 (m, 2F), -154.3 – -154.4 (m, 1F), -161.7 – -161.9 (m, 2F). HRMS (ASAP): Calcd. For $C_{12}H_6F_5NO$ $[M+H]^+$ 276.0442, Found: 276.0435.

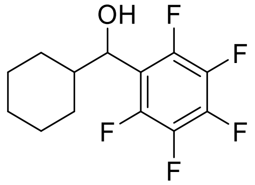

(**3o**): 90 mg, 80% yield, white solid. ^{1}H NMR (500 MHz, $CDCl_3$): δ (ppm) = 4.66 (d, J = 9 Hz, 1H), 2.45 (s, 1H), 2.16-2.13 (m, 1H), 1.83-1.78 (m, 2H), 1.68-1.66 (m, 2H), 1.29-1.13 (m, 4H), 1.05-1.03 (m, 1H), 0.96-0.88 (m, 1H). $^{13}C\{^{1}H\}$ NMR (125 MHz, $CDCl_3$): δ (ppm) = 144.7 (dm, $^{1}J_{F\text{-}C}$ = 250 Hz), 140.3 (dm, $^{1}J_{F\text{-}C}$ = 250 Hz), 137.4 (dm, $^{1}J_{F\text{-}C}$ = 250 Hz), 116.5 (tm, $J_{F\text{-}C}$ = 16 Hz), 71.4, 43.3, 29.7, 28.9, 26.1, 25.6, 25.5. ^{19}F NMR (470 MHz, $CDCl_3$): δ (ppm) = -142.9 – -143.0 (m, 2F), -155.7 (t, J_F = 19 Hz, 1F), -162.2 – -162.3 (m, 2F). HRMS (ASAP): Calcd. for $C_{13}H_{13}F_5O$ $[M-H]^+$ 279.0803, Found: 279.0800.

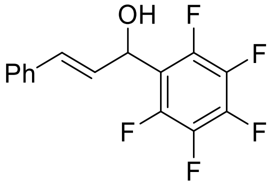

(**3p**):[2] 101 mg, 84% yield, white solid. ^{1}H NMR (500 MHz, $CDCl_3$): δ (ppm) = 7.40-7.38 (m, 2H), 7.36-7.33 (m, 2H), 7.30-7.27 (m, 1H), 6.71-6.68 (m, 1H), 6.55-6.50 (m, 1H), 5.75 (d, J = 6 Hz, 1H), 2.57 (s, 1H). $^{13}C\{^{1}H\}$ NMR (125 MHz, $CDCl_3$): δ (ppm) = 144.8 (dm, $^{1}J_{F\text{-}C}$ = 250 Hz), 140.8 (dm, $^{1}J_{F\text{-}C}$ = 250 Hz), 137.7 (dm, $^{1}J_{F\text{-}C}$ = 250 Hz), 135.7, 132.8, 128.6, 128.4, 127.5, 126.7, 116.1 (tm, $J_{F\text{-}C}$ = 15 Hz), 66.9. ^{19}F NMR (470 MHz, $CDCl_3$): δ (ppm) = -143.4

– -143.5 (m, 2F), -154.9 (t, J_F = 20 Hz, 1F), -161.7 – -161.8 (m, 2F). HRMS (ASAP): Calcd. for $C_{15}H_9F_5O$ 300.0574, Found: 300.0564.

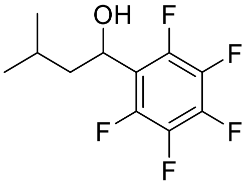

(**3q**): 77 mg, 76% yield, white solid. ^{1}H NMR (500 MHz, $CDCl_3$): δ (ppm) = 5.13-5.10 (m, 1H), 2.34 (s, 1H), 1.94-1.88 (m, 1H), 1.68-1.59 (m, 2H), 0.96-0.93 (m, 6H). $^{13}C\{^1H\}$ NMR (125 MHz, $CDCl_3$): δ (ppm) = 144.7 (dm, $^1J_{F\text{-}C}$ = 250 Hz), 140.4 (dm, $^1J_{F\text{-}C}$ = 250 Hz), 137.5 (dm, $^1J_{F\text{-}C}$ = 250 Hz), 117.4 (tm, $J_{F\text{-}C}$ = 15 Hz), 64.7 (m), 45.8, 25.1, 22,5, 22.1. ^{19}F NMR (470 MHz, $CDCl_3$): δ (ppm) = -144.2 – -144.3 (m, 2F), -155.6 – -155.7 (m, 1F), -161.9 – -162.1 (m, 2F). HRMS (ASAP): Calcd. for $C_{11}H_{11}F_5O$ $[M\text{-}H]^+$ 253.0646 , Found: 253.0643.

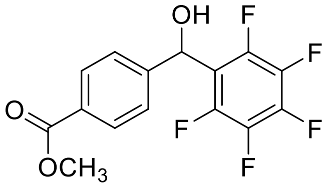

(**3r**): 114 mg, 86% yield, colorless oil. ^{1}H NMR (500 MHz, $CDCl_3$): δ (ppm) = 7.95-7.93 (m, 2H), 7.45-7.43 (m, 2H), 6.26 (s, 1H), 3.87 (s, 3H), 3.80 (s, 1H). $^{13}C\{^1H\}$ NMR (125 MHz, $CDCl_3$): δ (ppm) = 167.1, 145.9, 144.7 (dm, $^1J_{F\text{-}C}$ = 250 Hz), 141.0 (dm, $^1J_{F\text{-}C}$ = 250 Hz), 137.6 (dm, $^1J_{F\text{-}C}$ = 250 Hz), 129.8, 129.4, 125.4, 116.7 (tm, $J_{F\text{-}C}$ = 17 Hz), 66.5, 52.3. ^{19}F NMR (470 MHz, $CDCl_3$): δ (ppm) = -142.7 – -142.8 (m, 2F), -154.3 (t, J_F = 21 Hz, 1F), -161.4 – -161.5 (m, 2F). HRMS (ASAP): Calcd. for $C_{15}H_9F_5O_3$ $[M+H]^+$ 333.0545, Found: 333.0535.

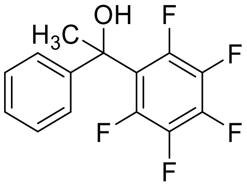

(**3s**): 81 mg, 70% yield, colorless oil. ^{1}H NMR (500 MHz, $CDCl_3$): δ (ppm) = 7.36-7.27 (m, 5H), 2.82 (s, 1H), 2.01 (t, J = 4 Hz, 3H). $^{13}C\{^1H\}$ NMR (125 MHz, $CDCl_3$): δ (ppm) = 146.8, 145.2 (dm, $^1J_{F\text{-}C}$ = 250 Hz), 140.5 (dm, $^1J_{F\text{-}C}$ = 250 Hz), 137.9 (dm, $^1J_{F\text{-}C}$ = 250 Hz), 128.6, 127.7, 124.1, 120.7 (tm, $J_{F\text{-}C}$ = 14 Hz), 76.9, 31.7 (t, $J_{F\text{-}C}$ = 6 Hz). ^{19}F NMR (470 MHz,

$CDCl_3$): δ (ppm) = -139.0 – -139.1 (m, 2F), -155.0 – -155.1 (m, 1F), -161.6 – -161.8 (m, 2F). HRMS (ASAP): Calcd. for $C_{14}H_9F_5O$ $[M-OH]^+$ 271.0541, Found: 271.0534.

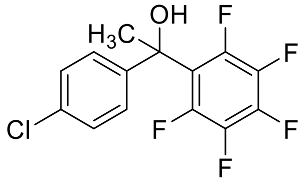

(**3t**): 92 mg, 71% yield, colorless oil. ^{1}H NMR (500 MHz, $CDCl_3$): δ (ppm) = 7.32-7.26 (m, 4H), 3.02 (s, 1H), 1.99 (t, J = 4 Hz, 3H). ^{13}C{^{1}H} NMR (125 MHz, $CDCl_3$): δ (ppm) = 145.4, 145.2 (dm, $^1J_{F\text{-}C}$ = 250 Hz), 140.6 (dm, $^1J_{F\text{-}C}$ = 250 Hz), 138.9 (dm, $^1J_{F\text{-}C}$ = 250 Hz), 133.6, 128.7, 125.7, 120.1 (tm, $J_{F\text{-}C}$ = 15 Hz), 76.5, 31.7 (t, $J_{F\text{-}C}$ = 6 Hz). ^{19}F NMR (470 MHz, $CDCl_3$): δ (ppm) = -138.9 – -139.1 (m, 2F), -154.4 – -154.5 (m, 1F), -161.2 – -161.4 (m, 2F). HRMS (ASAP): Calcd. for $C_{14}H_8F_5ClO$ 322.0184, Found: 322.0172.

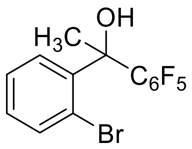

(**3u**): 89 mg, 61% yield, colorless oil. ^{1}H NMR (500 MHz, $CDCl_3$): δ (ppm) = 7.88-7.86 (m, 1H), 7.53-7.51 (m, 1H), 7.42-7.39 (m, 1H), 7.19-7.16 (m, 1H), 3.27 (s, 1H), 2.11 (t, J = 3 Hz, 3H). ^{13}C{^{1}H} NMR (125 MHz, $CDCl_3$): δ (ppm) = 145.2 (dm, $^1J_{F\text{-}C}$ = 250 Hz), 144.4, 140.6 (dm, $^1J_{F\text{-}C}$ = 250 Hz), 137.7 (dm, $^1J_{F\text{-}C}$ = 250 Hz), 134.6, 129.5, 127.7, 126.7 (t, $J_{F\text{-}C}$ = 2 Hz), 120.1, 119.7 (tm, $J_{F\text{-}C}$ = 15 Hz), 76.2, 28.9 (t, $J_{F\text{-}C}$ = 4 Hz). ^{19}F NMR (470 MHz, $CDCl_3$): δ (ppm) = -139.1 – -139.2 (m, 2F), -155.4 – -155.5 (m, 1F), -162.3 – -162.5 (m, 2F). HRMS (ASAP): Calcd. for $C_{14}H_8F_5BrO$ 365.9679, Found: 365.9668.

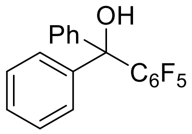

(**3v**): 84 mg, 60% yield, colorless oil. ^{1}H NMR (500 MHz, $CDCl_3$): δ (ppm) = 7.38-7.36 (m, 6H), 7.31-7.29 (m, 4H), 3.56 (s, 1H). ^{13}C{^{1}H} NMR (125 MHz, $CDCl_3$): δ (ppm) = 145.1 (dm, $^1J_{F\text{-}C}$ = 250 Hz), 144.3, 140.7 (dm, $^1J_{F\text{-}C}$ = 250 Hz), 138.1 (dm, $^1J_{F\text{-}C}$ = 250 Hz), 128.4, 128.3, 126.9, 121.1 (tm, $J_{F\text{-}C}$ = 12 Hz), 80.9. ^{19}F NMR (470 MHz, $CDCl_3$): δ (ppm) = -136.4

– -136.5 (m, 2F), -154.5 – -154.6 (m, 1F), -161.5 – -161.7 (m, 2F). HRMS (ASAP): Calcd. for $C_{19}H_{11}F_5O$ 350.0730, Found: 350.0719.

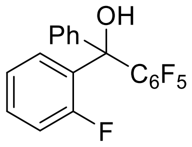

(**3w**): 93 mg, 63% yield, colorless oil. ^{1}H NMR (500 MHz, $CDCl_3$): δ (ppm) = 7.41-7.33 (m, 6H), 7.11-7.05 (m, 2H), 6.94-6.90 (m, 1H), 3.69 (s, 1H). ^{13}C{^{1}H} NMR (125 MHz, $CDCl_3$): δ (ppm) = 160.9 (d, $^1J_{F\text{-}C}$ = 250 Hz), 145.1 (dm, $^1J_{F\text{-}C}$ = 250 Hz), 142.5, 140.5 (dm, $^1J_{F\text{-}C}$ = 250 Hz), 137.7 (dm, $^1J_{F\text{-}C}$ = 250 Hz), 131.9 (d, $J_{F\text{-}C}$ = 11 Hz), 130.4 (d, $J_{F\text{-}C}$ = 9 Hz), 128.6, 128.5, 126.7, 124.0 (d, $J_{F\text{-}C}$ = 3 Hz), 119.9 (tm, $J_{F\text{-}C}$ = 15 Hz), 116.0 (d, $J_{F\text{-}C}$ = 22 Hz), 78.5. ^{19}F NMR (470 MHz, $CDCl_3$): δ (ppm) = -111.5 – -111.6 (m, 1F), -137.5 – -137.6 (m, 2F), -154.6 – -154.7 (m, 1F), -161.8 – -161.9 (m, 2F). HRMS (ASAP): Calcd. for $C_{19}H_{10}F_6O$ 368.0636, Found: 368.0625.

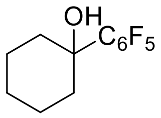

(**3x**): 76 mg, 71% yield, white solid. ^{1}H NMR (500 MHz, $CDCl_3$): δ (ppm) = 2.38 (s, 1H), 2.08-2.00 (m, 4H), 1.86-1.69 (m, 3H), 1.63-1.59 (m, 2H), 1.35-1.27 (m, 1H). ^{13}C{^{1}H} NMR (125 MHz, $CDCl_3$): δ (ppm) = 145.2 (dm, $^1J_{F\text{-}C}$ = 250 Hz), 139.7 (dm, $^1J_{F\text{-}C}$ = 250 Hz), 137.8 (dm, $^1J_{F\text{-}C}$ = 250 Hz), 121.6 (tm, $J_{F\text{-}C}$ = 12 Hz), 75.4, 37.3 (t, $J_{F\text{-}C}$ = 4 Hz), 25.1, 21.4. ^{19}F NMR (470 MHz, $CDCl_3$): δ (ppm) = -140.1 – -140.2 (m, 2F), -156.5 – -156.6 (m, 1F), -162.1 – -162.2 (m, 2F). HRMS (ASAP): Calcd. for $C_{12}H_{11}F_5O$ 266.0730, Found: 266.0721.

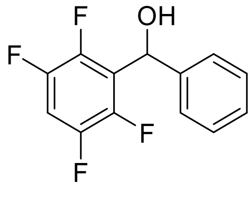

(**4a**): 84 mg, 82% yield, colorless oil. ^{1}H NMR (500 MHz, $CDCl_3$): δ (ppm) = 7.42-7.36 (m, 4H), 7.34-7.30 (m, 1H), 7.05-6.98 (m, 1H), 6.26 (s, 1H), 2.97 (s, 1H). ^{13}C{^{1}H} NMR (125 MHz, $CDCl_3$): δ (ppm) = 146.1 (dm, $^1J_{F\text{-}C}$ = 250 Hz), 144.1 (dm, $^1J_{F\text{-}C}$ = 250 Hz), 140.7, 128.7, 128.2, 125.5, 122.7 (t, $J_{F\text{-}C}$ = 15 Hz), 105.5 (t, $J_{F\text{-}C}$ = 23 Hz), 67.9 (m). ^{19}F NMR (470

MHz, $CDCl_3$): δ (ppm) = -138.5 – -138.6 (m, 2F), -143.6 – -143.7 (m, 2F). HRMS (ASAP): calcd. for $C_{13}H_8F_4O$ 256.0511, Found: 256.0500.

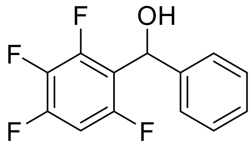

(**4b**): 86 mg, 84% yield, colorless oil. ^{1}H NMR (500 MHz, $CDCl_3$): δ (ppm) = 7.40-7.35 (m, 4H), 7.32-7.29 (m, 1H), 6.81-6.76 (m, 1H), 6.19 (s, 1H), 2.77 (s, 1H). ^{13}C{^{1}H} NMR (125 MHz, $CDCl_3$): δ (ppm) = 154.8 (dm, $^1J_{F\text{-}C}$ = 250 Hz), 150.2 (dm, $^1J_{F\text{-}C}$ = 250 Hz), 149.6 (dm, $^1J_{F\text{-}C}$ = 250 Hz), 141.1 (m), 137.3 (dm, $^1J_{F\text{-}C}$ = 250 Hz), 128.6, 128.0, 125.4 (t, $J_{F\text{-}C}$ = 1 Hz), 117.0 (tm, $J_{F\text{-}C}$ = 19 Hz), 101.3 (m), 67.4 (m). ^{19}F NMR (470 MHz, $CDCl_3$): δ (ppm) = -117.8 – -117.9 (m, 1F), -132.4 – -132.5 (m, 1F), -135.2 – -135.3 (m, 1F), -164.2 – -164.4 (m, 1F). HRMS (ASAP): calcd. for $C_{13}H_8F_4O$ 256.0511, Found: 256.0499.

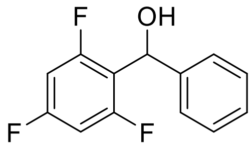

(**4c**): 76 mg, 80% yield, colorless oil. ^{1}H NMR (500 MHz, $CDCl_3$): δ (ppm) = 7.40-7.34 (m, 4H), 7.31-7.27 (m, 1H), 6.71-6.65 (m, 2H), 6.19 (s, 1H), 2.77 (s, 1H). ^{13}C{^{1}H} NMR (125 MHz, $CDCl_3$): δ (ppm) = 162.2 (dt, $^1J_{F\text{-}C}$ = 250 Hz, $J_{F\text{-}C}$ = 16 Hz), 161.1 (ddd, $^1J_{F\text{-}C}$ = 250 Hz, $J_{F\text{-}C}$ = 15 Hz, 11 Hz), 141.8, 128.5, 127.7, 125.5 (t, $J_{F\text{-}C}$ = 1 Hz), 115.9 (td, $J_{F\text{-}C}$ = 17 Hz, 5 Hz), 100.8 (dd, $J_{F\text{-}C}$ = 54 Hz, 2 Hz), 67.2 (t, $J_{F\text{-}C}$ = 3 Hz). ^{19}F NMR (470 MHz, $CDCl_3$): δ (ppm) = -108.1 – -108.2 (m, 1F), -111.1 – -111.2 (m, 2F). HRMS (ASAP): calcd. for $C_{13}H_9F_3O$ 238.0600, Found: 238.0595.

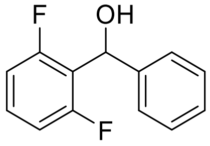

(**4d**): 70 mg, 80% yield, white solid. ^{1}H NMR (500 MHz, $CDCl_3$): δ (ppm) = 7.44-7.42 (m, 2H), 7.39-7.32 (m, 2H), 7.31-7.23 (m, 2H), 6.94-6.88 (m, 2H), 6.27 (s, 1H), 2.90 (s, 1H). ^{13}C{^{1}H} NMR (125 MHz, $CDCl_3$): δ (ppm) = 160.8 (dd, $^1J_{F\text{-}C}$ = 250 Hz, $J_{F\text{-}C}$ = 8 Hz), 142.1, 129.6 (t, $J_{F\text{-}C}$ = 11 Hz), 128.4, 127.6, 125.6 (t, $J_{F\text{-}C}$ = 1 Hz), 119.5 (t, $J_{F\text{-}C}$ = 16 Hz), 112.0 (m),

67.6 (t, $J_{F\text{-}C}$ = 3 Hz). ^{19}F NMR (470 MHz, $CDCl_3$): δ (ppm) = -114.4 – -114.5 (m, 2F). HRMS (ASAP): calcd. for $C_{13}H_{10}F_2O$ 220.0694, Found: 220.0691.

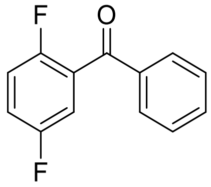

(**4e**):[3,4] 69 mg, 79% yield, colorless oil. ^{1}H NMR (500 MHz, $CDCl_3$): δ (ppm) = 7.85-7.83 (m, 2H), 7.64-7.61 (m, 1H), 7.51-7.47 (m, 2H), 7.28-7.20 (m, 2H), 7.17-7.12 (m, 1H). ^{13}C{^{1}H} NMR (125 MHz, $CDCl_3$): δ (ppm) = 191.9 (m), 158.4 (dd, $^{1}J_{F\text{-}C}$ = 250 Hz, $J_{F\text{-}C}$ = 2 Hz), 155.9 (dd, $^{1}J_{F\text{-}C}$ = 250 Hz, $J_{F\text{-}C}$ = 2 Hz), 136.7, 133.8, 129.8 (d, $J_{F\text{-}C}$ = 1 Hz), 128.6, 128.1 (dd, $J_{F\text{-}C}$ = 18 Hz, 7 Hz), 119.5 (dd, $J_{F\text{-}C}$ = 24 Hz, 9 Hz), 117.6 (dd, $J_{F\text{-}C}$ = 25 Hz, 8 Hz), 116.9 (dd, $J_{F\text{-}C}$ = 25 Hz, 4 Hz). ^{19}F NMR (470 MHz, $CDCl_3$): δ (ppm) = -117.0 – -117.1 (m, 1F), -117.6 – -117.7 (m, 1F). HRMS (ASAP): calcd. for $C_{13}H_8F_2O$ $[M+H]^+$ 219.0618, Found: 219.0614.

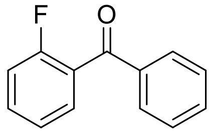

(**4f**):[5,6] 60 mg, 75% yield, colorless oil. ^{1}H NMR (500 MHz, $CDCl_3$): δ (ppm) = 7.85-7.83 (m, 2H), 7.60-7.44 (m, 5H), 7.22-7.24 (m, 1H), 7.17-7.12 (m, 1H). ^{13}C{^{1}H} NMR (125 MHz, $CDCl_3$): δ (ppm) = 193.5, 160.1 (d, $^{1}J_{F\text{-}C}$ = 250 Hz), 137.4 (d, $J_{F\text{-}C}$ = 1 Hz), 133.5, 133.1 (d, $J_{F\text{-}C}$ = 8 Hz), 130.7 (d, $J_{F\text{-}C}$ = 3 Hz), 129.8 (d, $J_{F\text{-}C}$ = 1 Hz), 128.5, 127.0 (d, $J_{F\text{-}C}$ = 15 Hz), 124.3 (d, $J_{F\text{-}C}$ = 4 Hz), 116.3 (d, $J_{F\text{-}C}$ = 21 Hz). ^{19}F NMR (470 MHz, $CDCl_3$): δ (ppm) = -111.0 – -111.1 (m, 1F). HRMS (ASAP): calcd. for $C_{13}H_9FO$ $[M+H]^+$ 201.0710, Found: 201.0706.

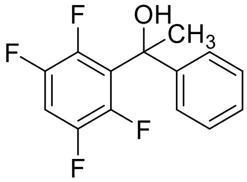

(**4g**): 73 mg, 68% yield, colorless oil. ^{1}H NMR (500 MHz, $CDCl_3$): δ (ppm) = 7.35-7.34 (m, 4H), 7.30-7.27 (m, 1H), 7.08-7.01 (m, 1H), 3.21 (s, 1H), 2.03 (t, J = 4 Hz, 3H). ^{13}C{^{1}H} NMR (125 MHz, $CDCl_3$): δ (ppm) = 147.1, 146.4 (dm, $^{1}J_{F\text{-}C}$ = 250 Hz), 144.8 (dm, $^{1}J_{F\text{-}C}$ = 250 Hz), 128.5, 127.6, 126.1 (t, $J_{F\text{-}C}$ = 12 Hz), 124.2, 105.2 (t, $J_{F\text{-}C}$ = 22 Hz), 77.1, 31.5 (t,

J_{F-C} = 7 Hz). ^{19}F NMR (470 MHz, $CDCl_3$): δ (ppm) = -138.5 – -138.6 (m, 2F), -139.6 – -139.7 (m, 2F). HRMS (ASAP): calcd. for $C_{14}H_{10}F_4O$ [M-OH]$^+$ 253.0635, Found: 253.0630.

V. ^{1}H NMR, ^{19}F NMR and ^{13}C NMR Spectra

Compound 3a： ^{1}H NMR spectrum (500 MHz, $CDCl_3$).

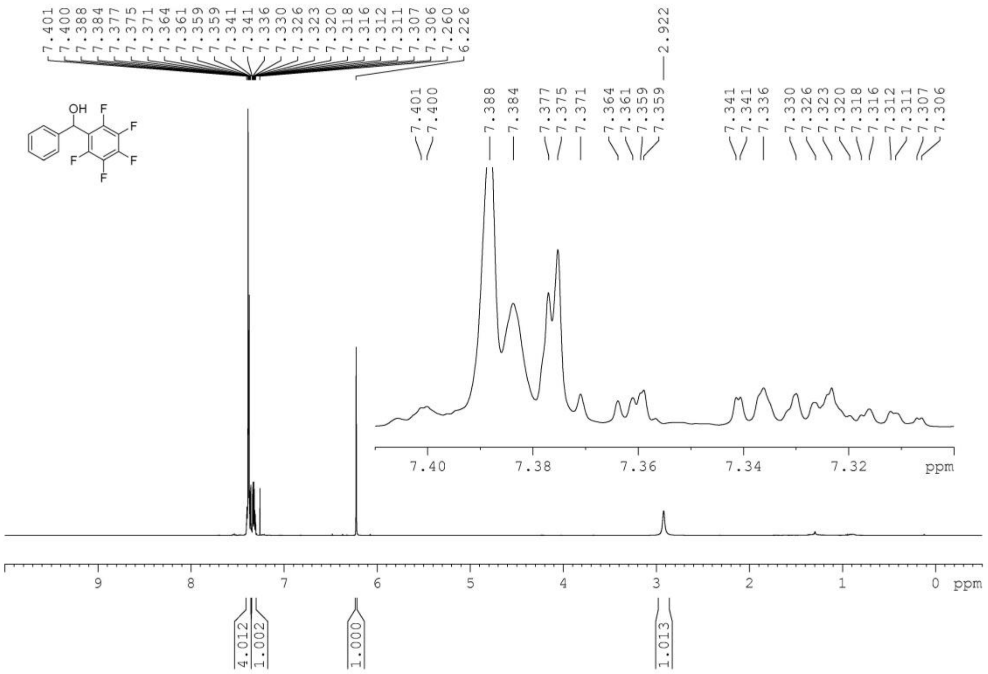

Compound 3a: ^{13} C{^{1}H} NMR spectrum (125 MHz, $CDCl_3$).

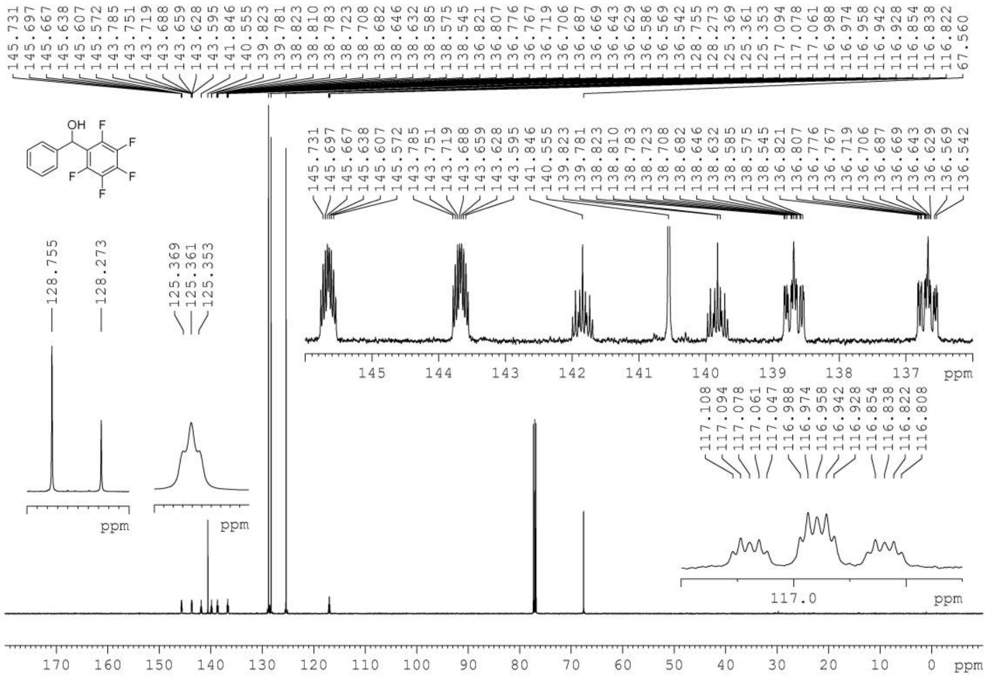

Compound 3a: ^{19} F NMR spectrum (470 MHz, $CDCl_3$).

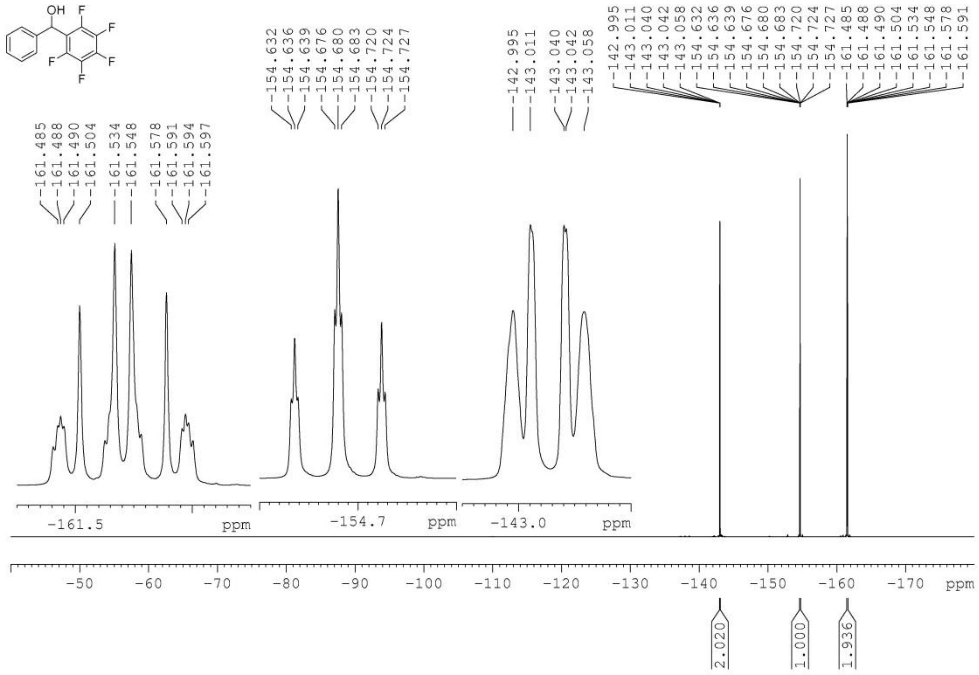

Compound 3b: ^{1}H NMR spectrum (500 MHz, CDCl$_3$).

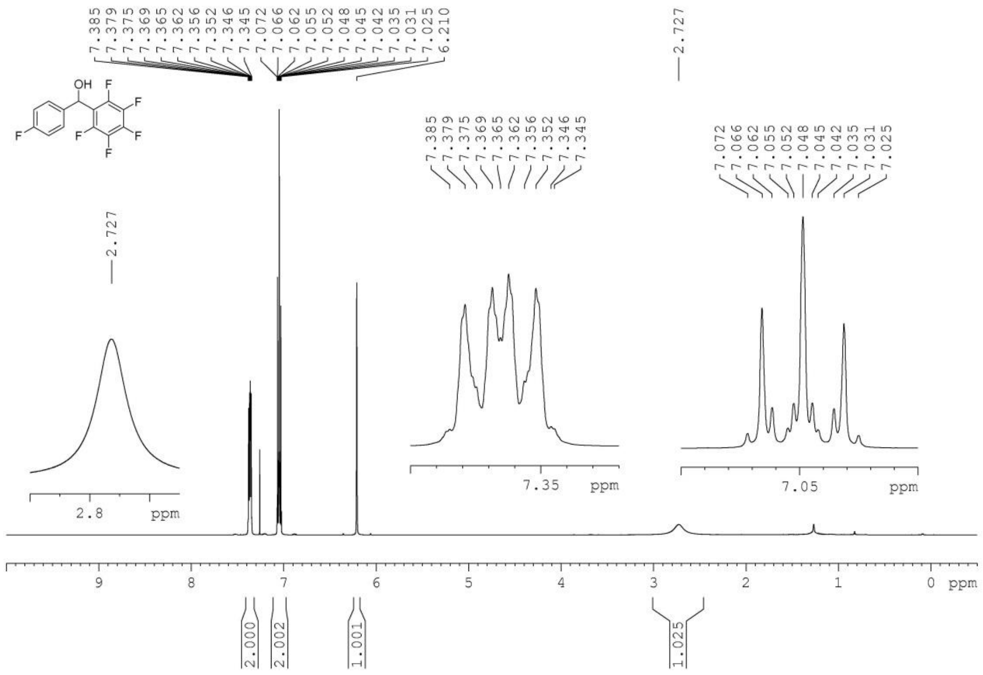

Compound 3b: ^{13}C{^{1}H} NMR spectrum (125 MHz, CDCl$_3$).

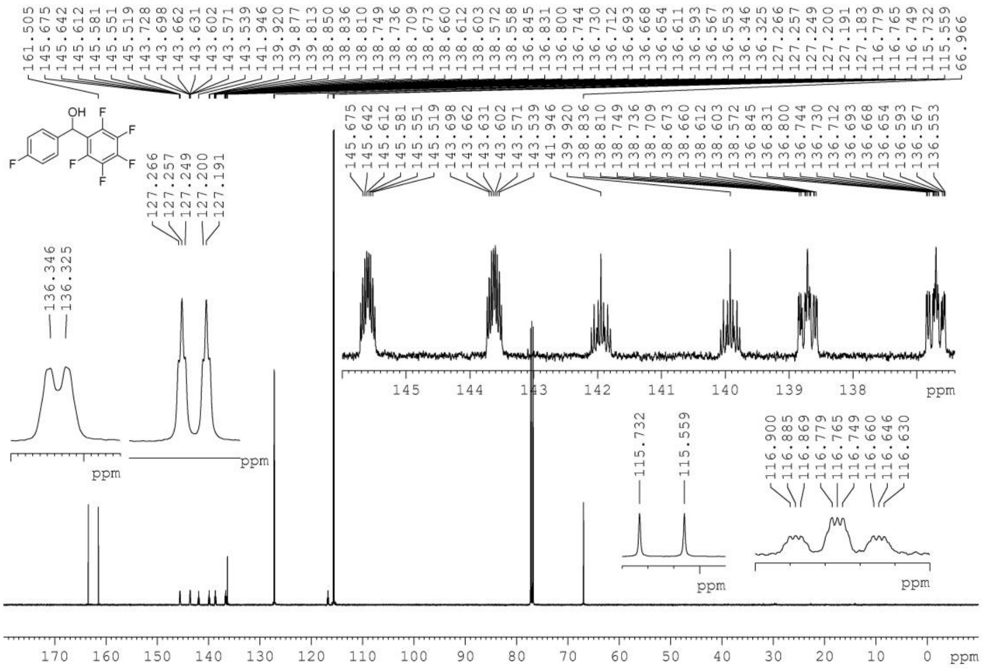

Compound 3b: [19] F NMR spectrum (470 MHz, $CDCl_3$).

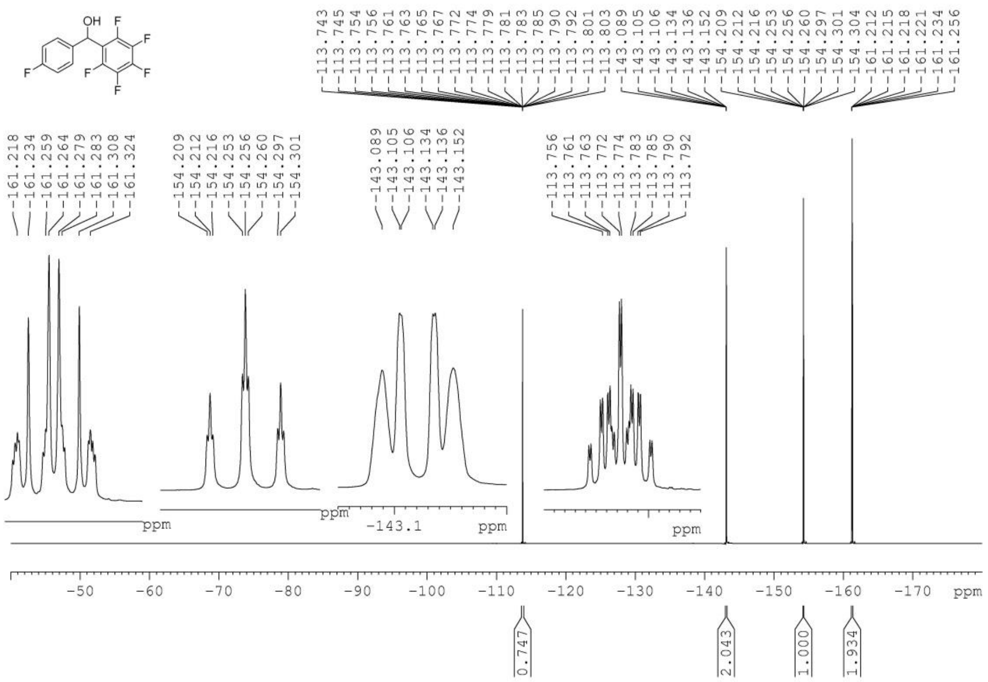

Compound 3c: [1] H NMR spectrum (500 MHz, $CDCl_3$).

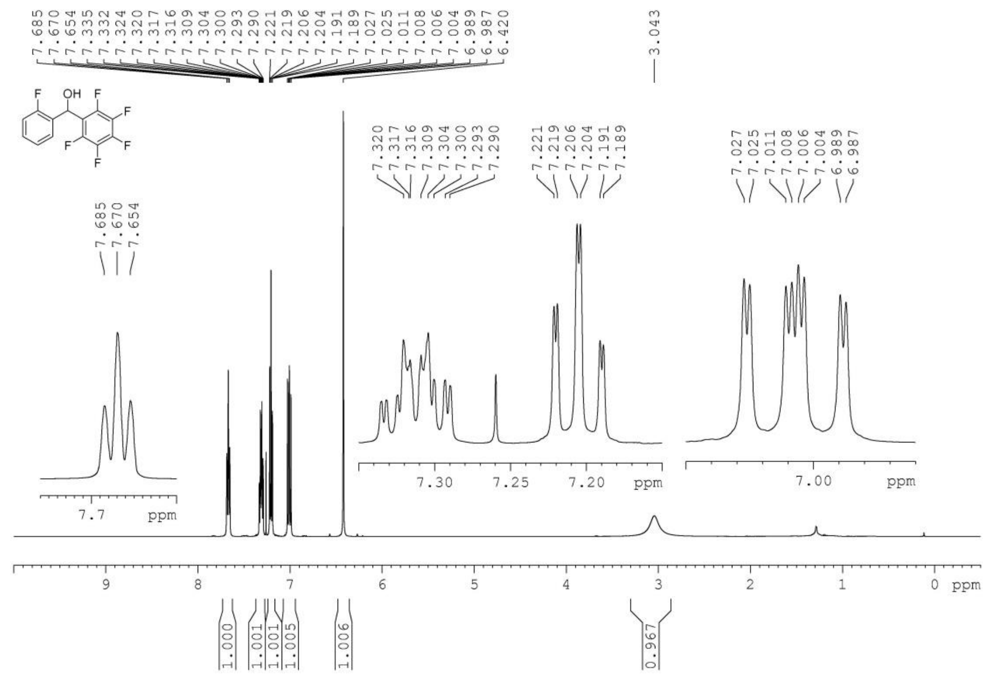

Compound 3c: ^{13}C{^{1}H} NMR spectrum (125 MHz, $CDCl_3$).

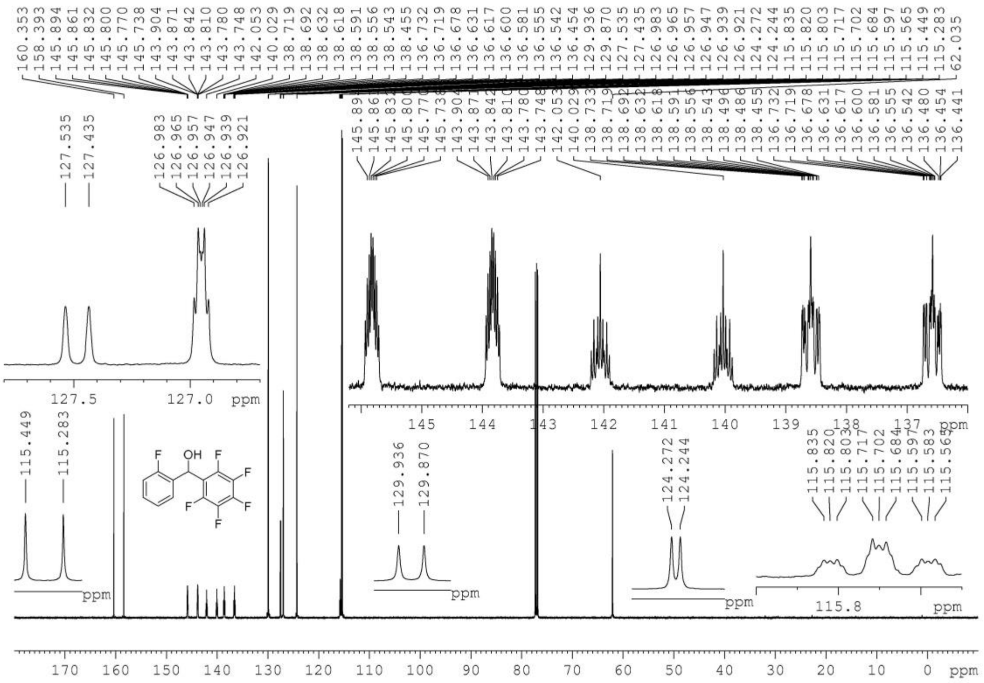

Compound 3c: ^{19}F NMR spectrum (470 MHz, $CDCl_3$).

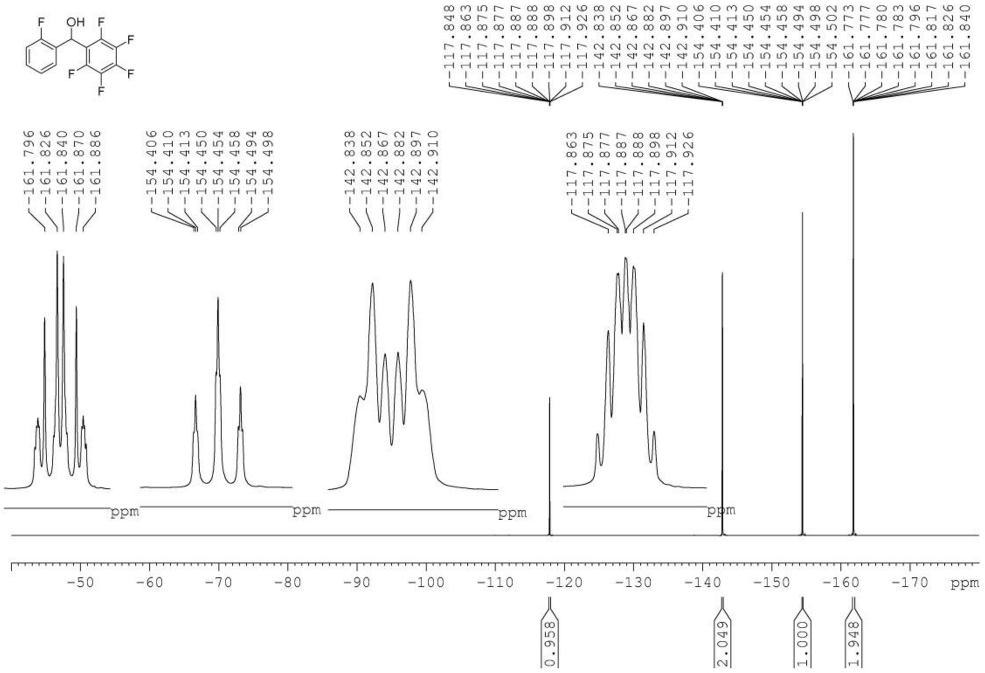

Compound 3d： [1] **H NMR spectrum (500 MHz, CDCl3).**

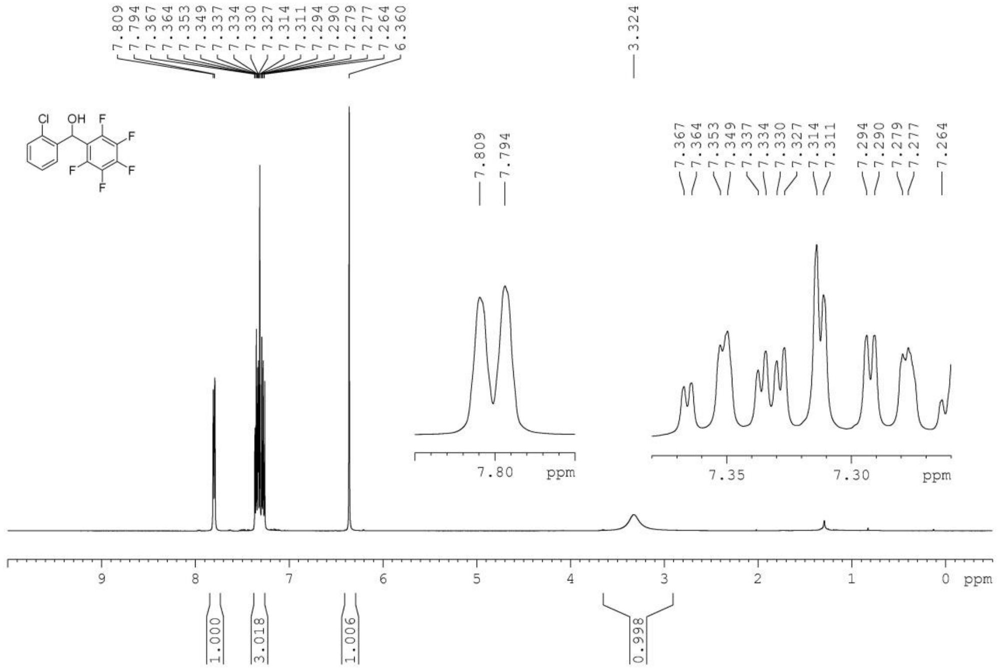

Compound 3d： [13] **C{^{1}H} NMR spectrum (125 MHz, CDCl3).**

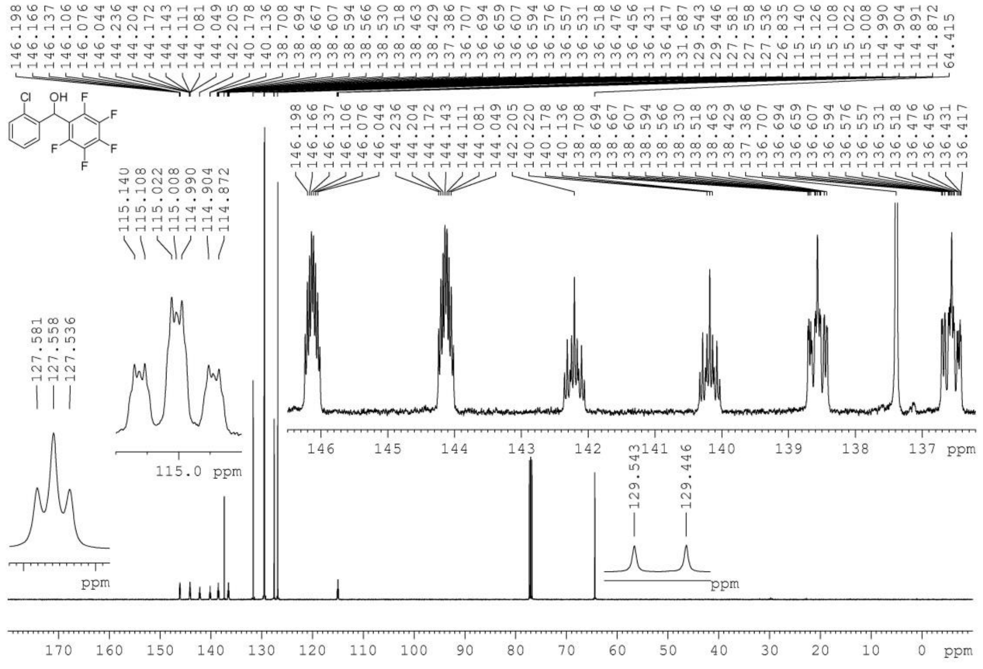

Compound 3d：[19] F NMR spectrum (470 MHz, CDCl3).

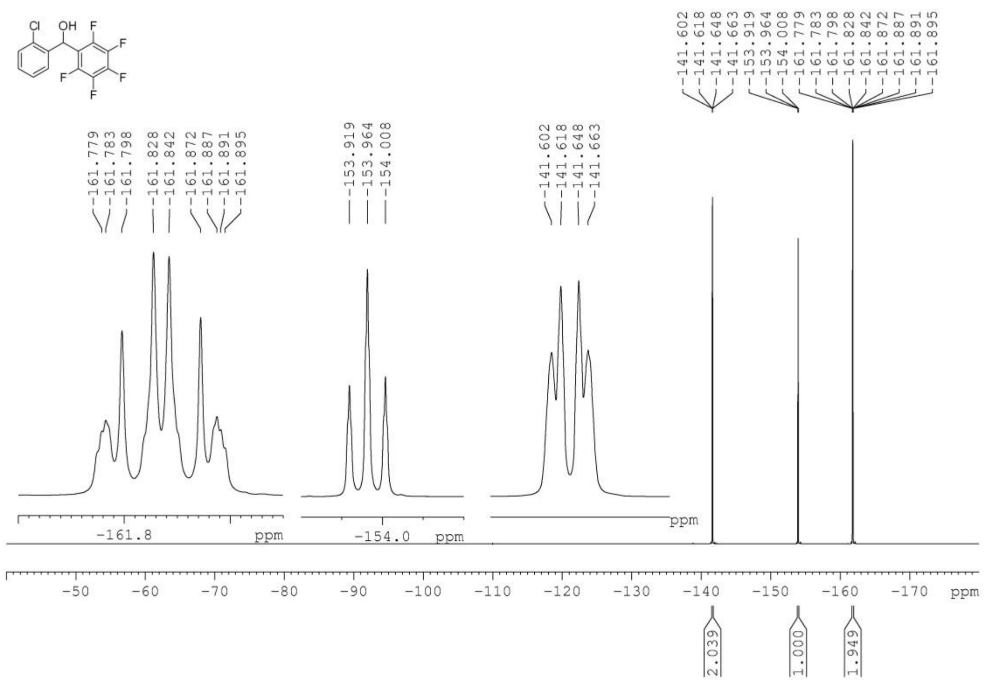

Compound 3e：[1] H NMR spectrum (500 MHz, CDCl3).

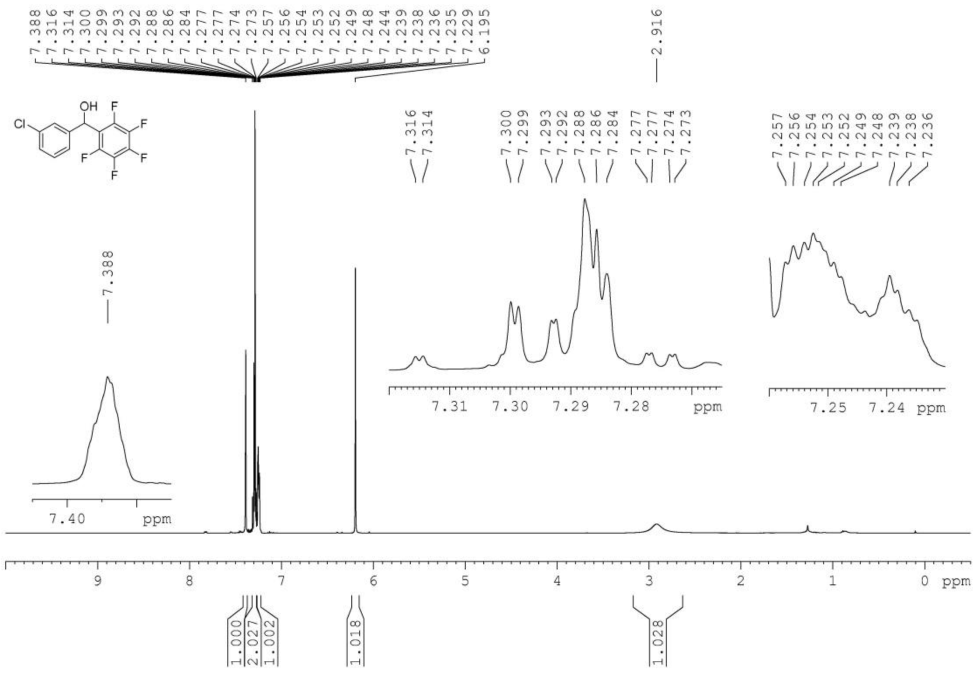

Compound 3e： $^{13}C\{^1H\}$ **NMR spectrum (125 MHz, $CDCl_3$).**

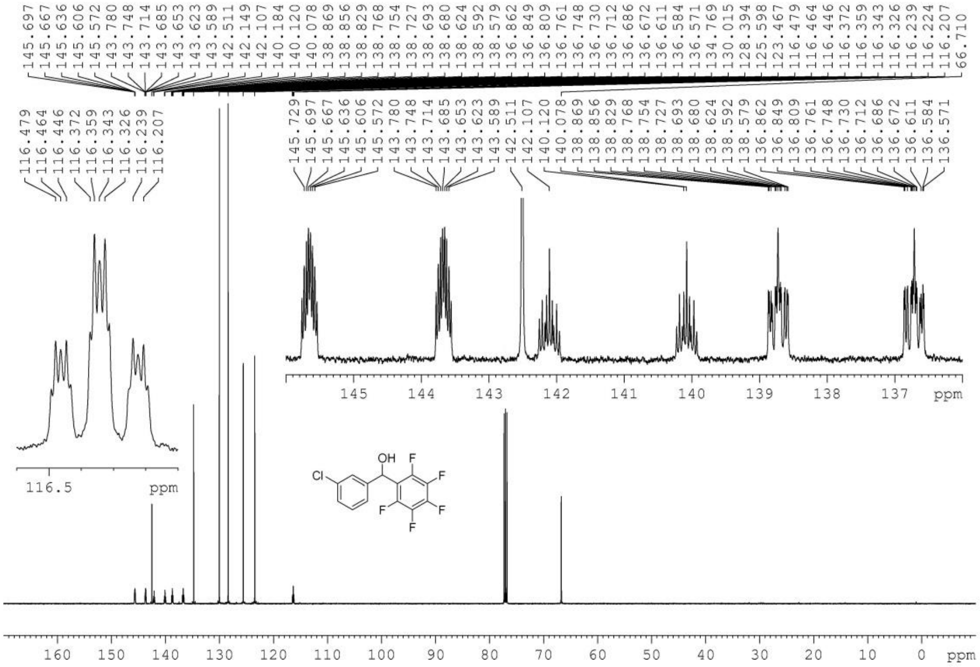

Compound 3e： ^{19}F **NMR spectrum (470 MHz, $CDCl_3$).**

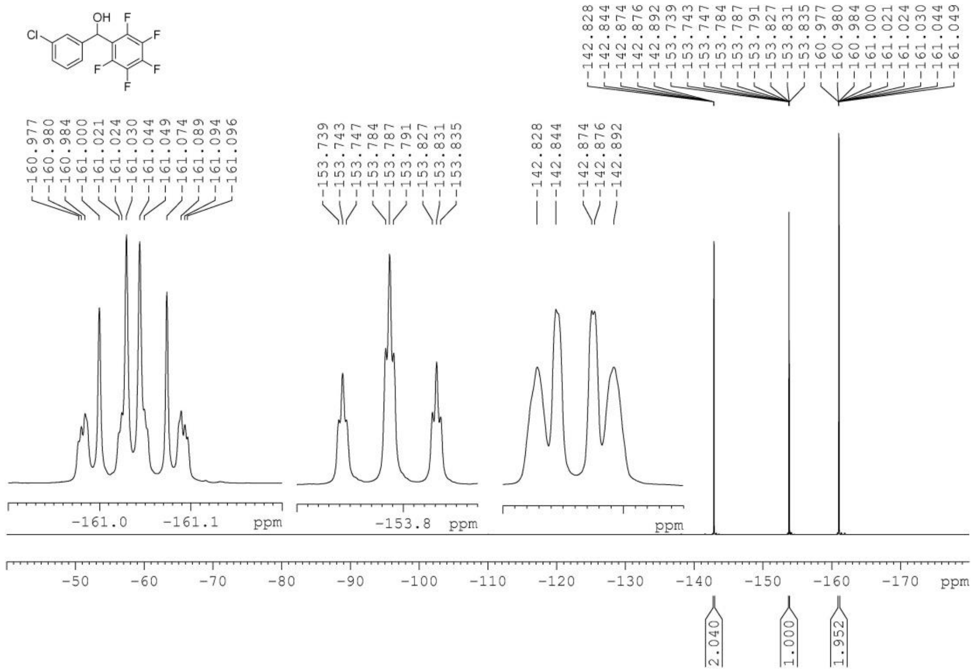

Compound 3f：^{1}H NMR spectrum (500 MHz, $CDCl_3$).

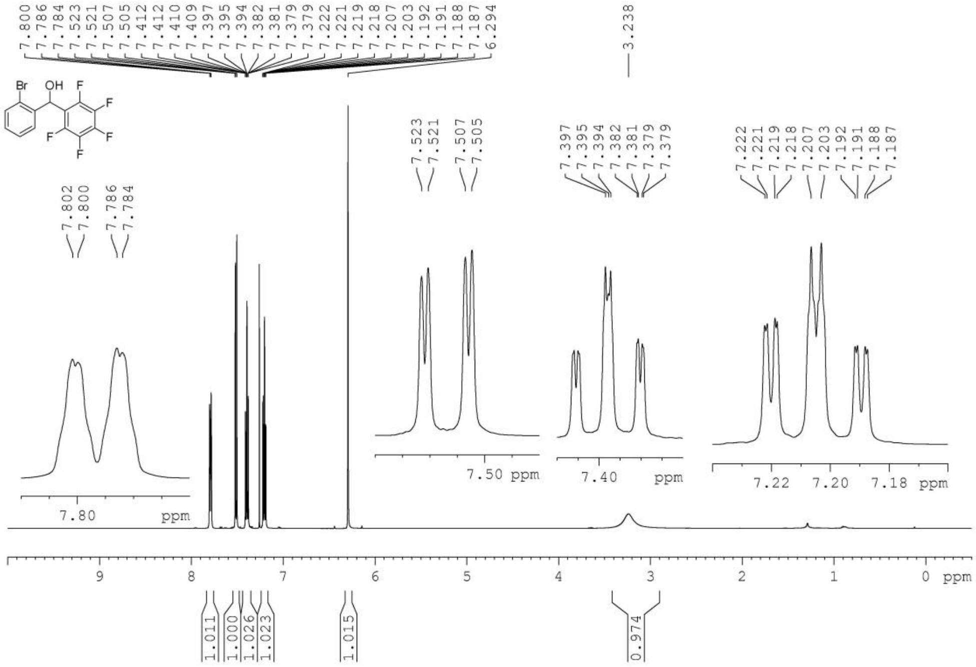

Compound 3f：^{13}C{^{1}H} NMR spectrum (125 MHz, $CDCl_3$).

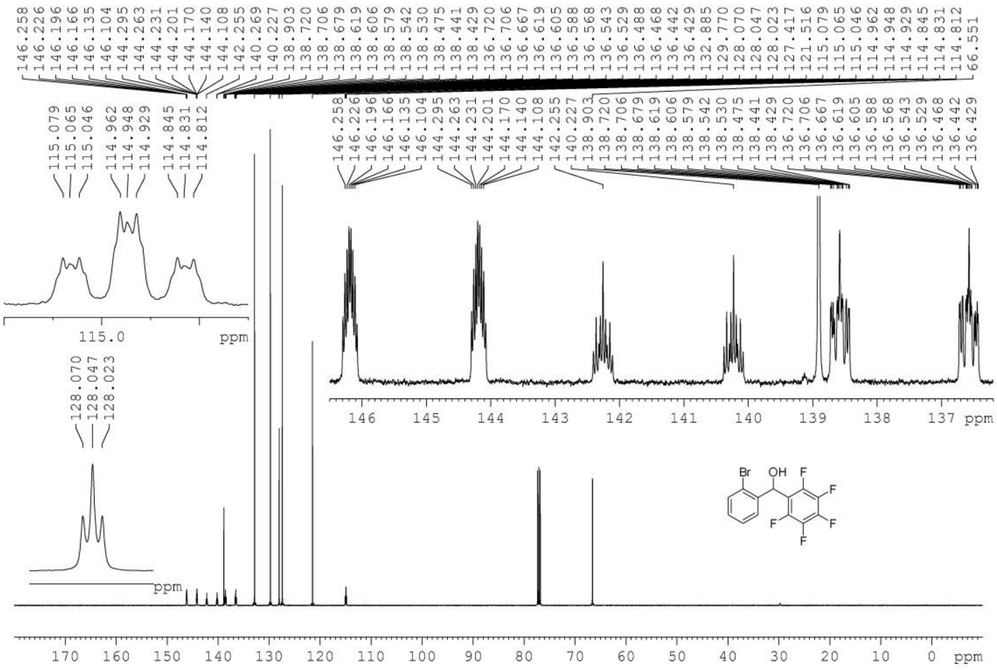

Compound 3f：19 F NMR spectrum (470 MHz, $CDCl_3$).

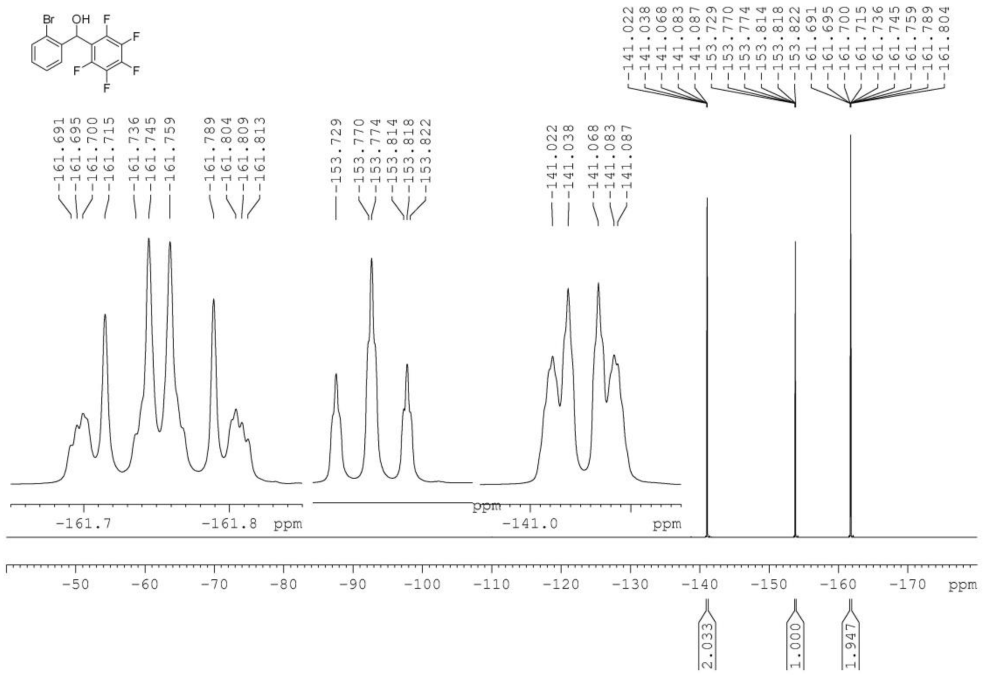

Compound 3g：1 H NMR spectrum (500 MHz, $CDCl_3$).

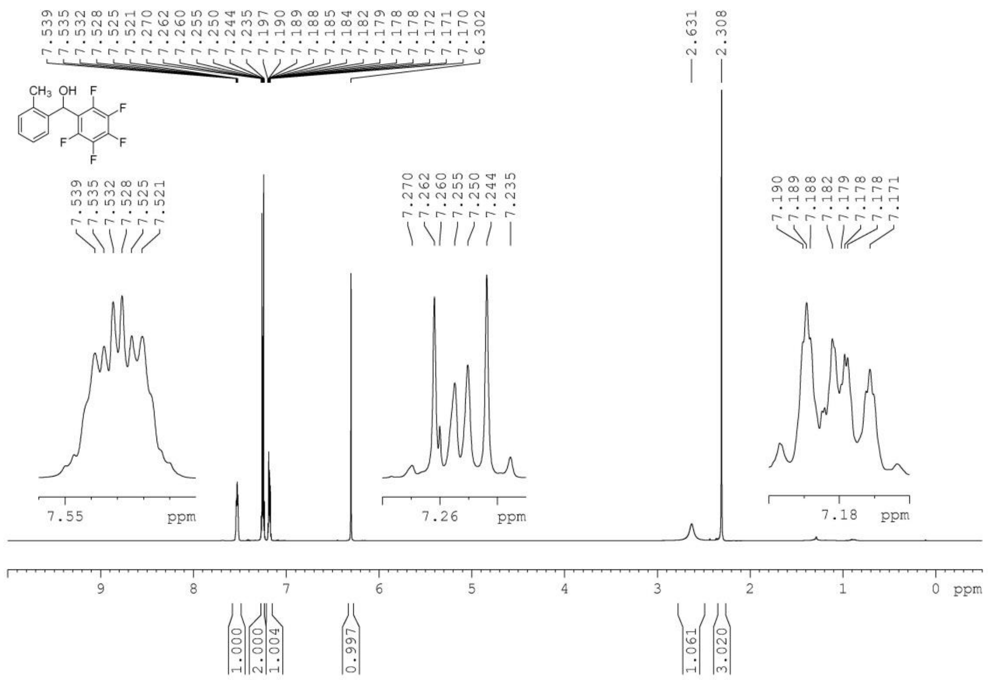

Compound 3g: [13] C{^{1}H} NMR spectrum (125 MHz, $CDCl_3$).

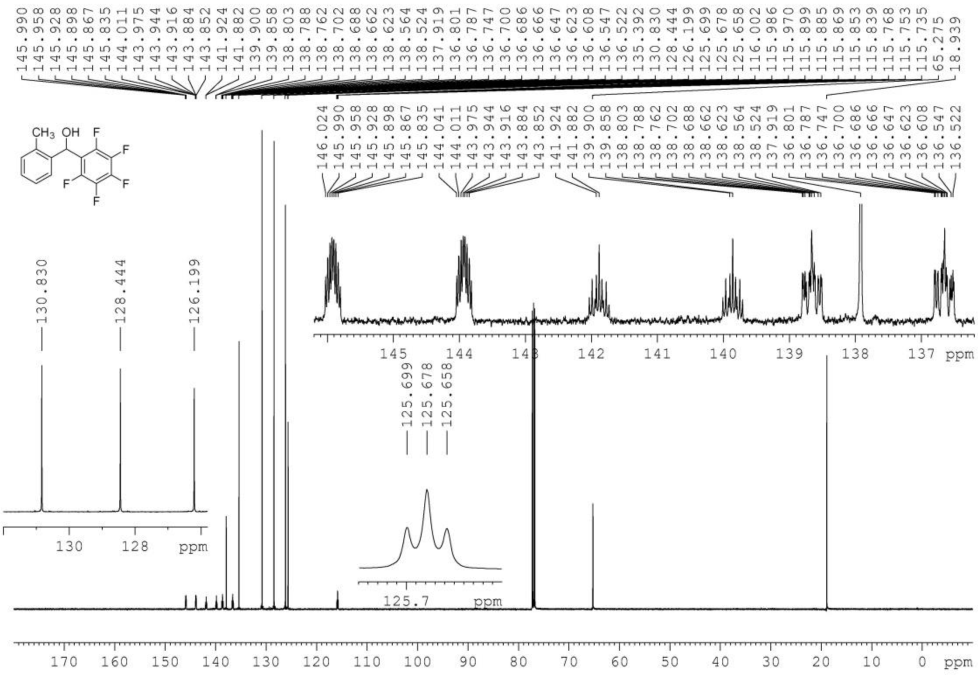

Compound 3g: [19] F NMR spectrum (470 MHz, $CDCl_3$).

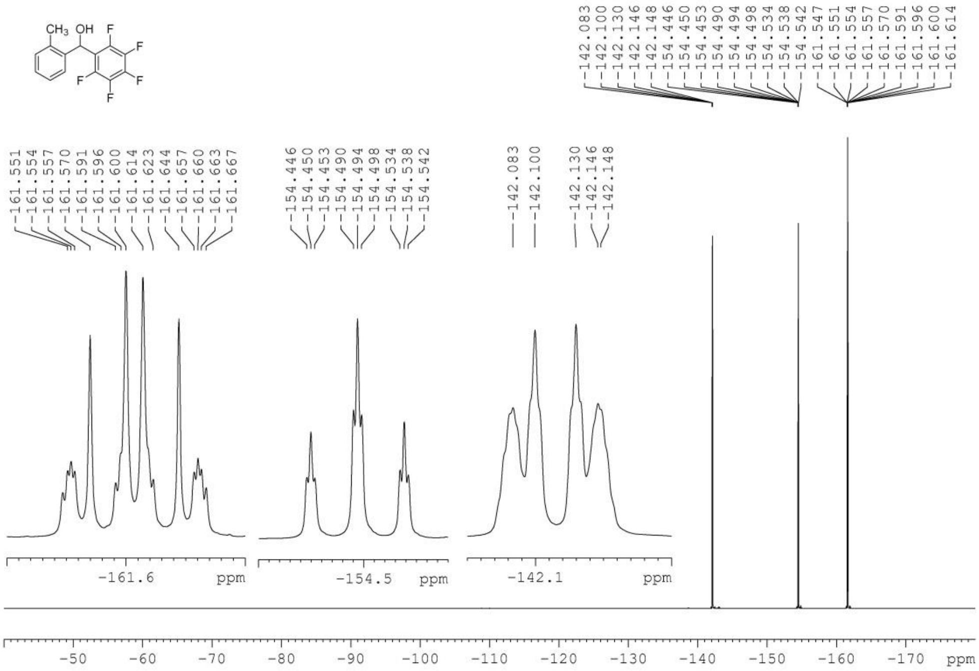

Compound 3h：^{1}H NMR spectrum (500 MHz, $CDCl_3$).

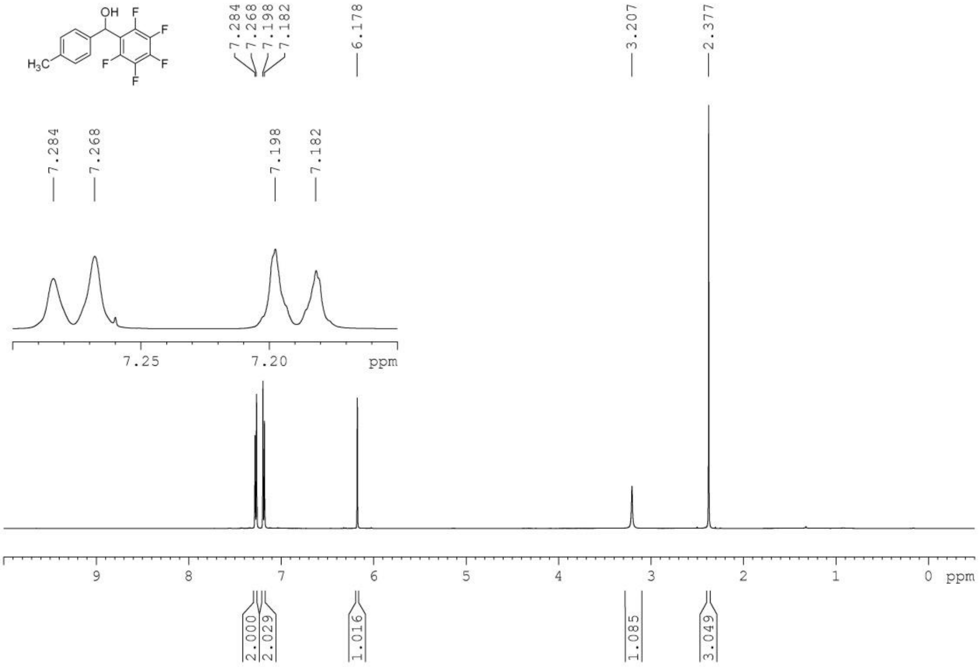

Compound 3h：^{13}C{^{1}H} NMR spectrum (125 MHz, $CDCl_3$).

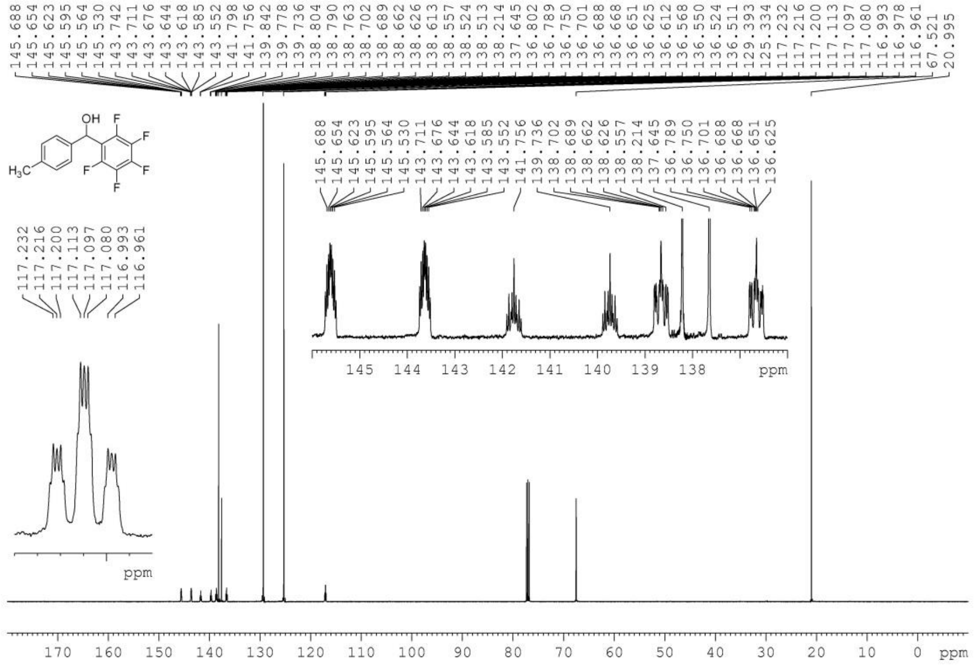

Compound 3h: [19] F NMR spectrum (470 MHz, CDCl3).

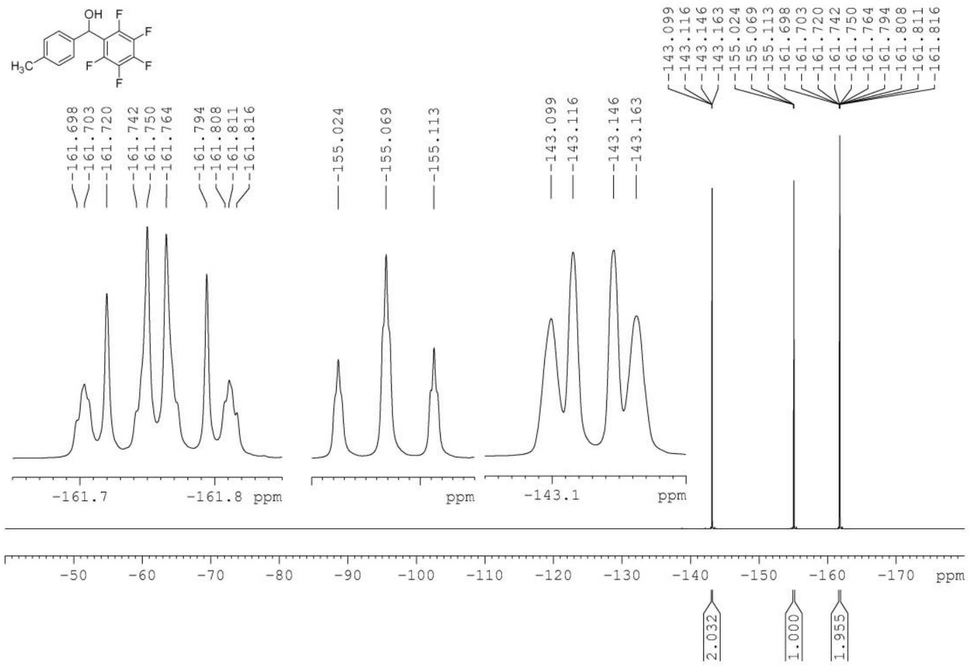

Compound 3i: [1] H NMR spectrum (500 MHz, CDCl3).

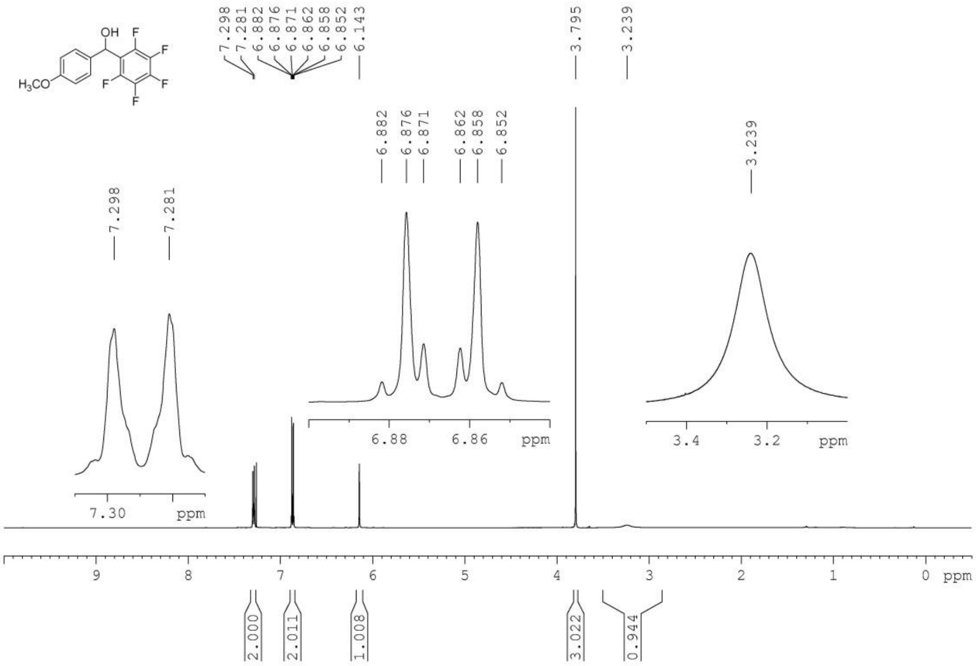

Compound 3i： [13] C{^{1}H} NMR spectrum (125 MHz, CDCl$_3$).

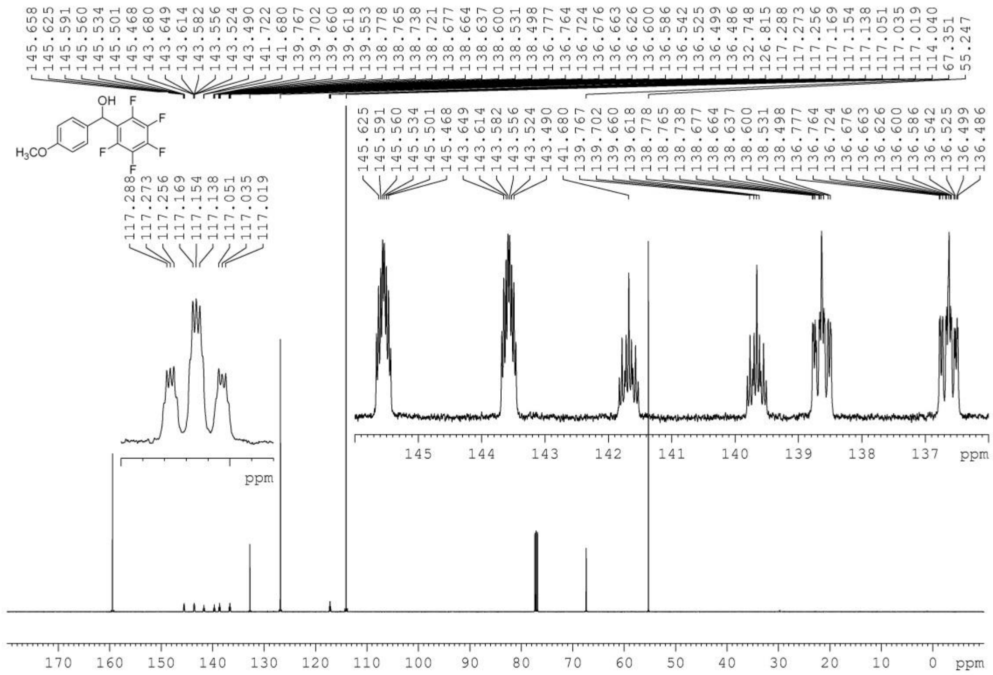

Compound 3i： [19] F NMR spectrum (470 MHz, CDCl$_3$).

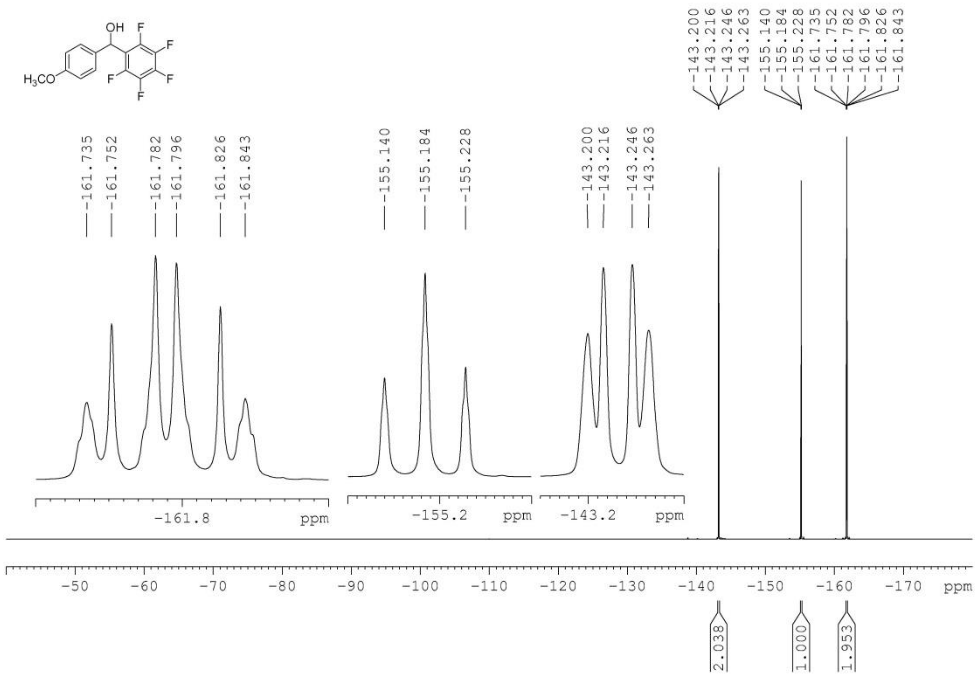

Compound 3j: ^{1}H NMR spectrum (500 MHz, $CDCl_3$).

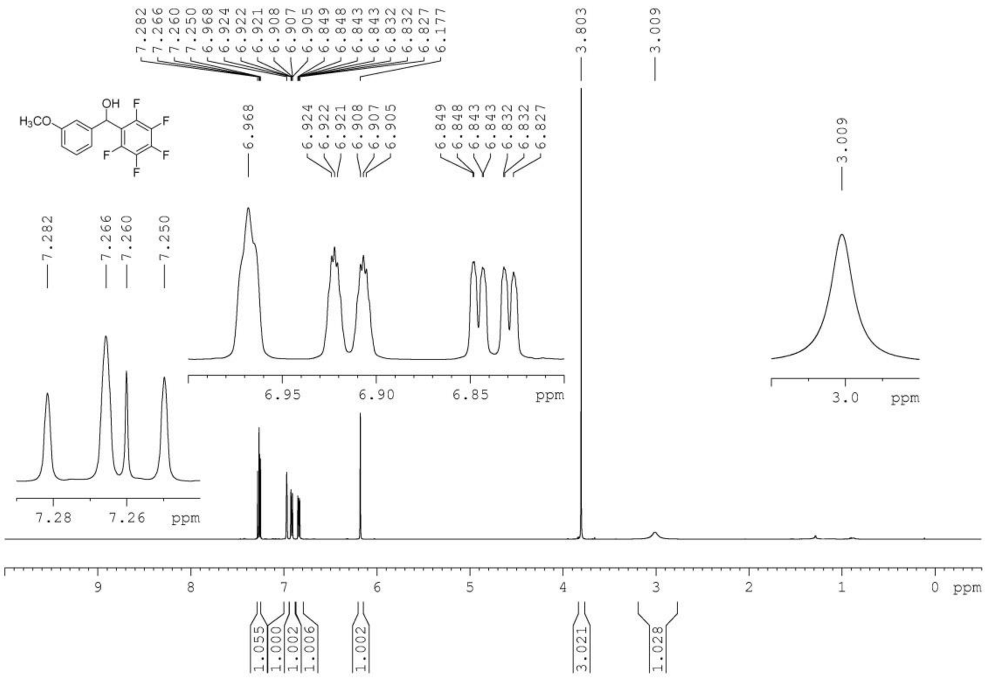

Compound 3j: ^{13}C{^{1}H} NMR spectrum (125 MHz, $CDCl_3$).

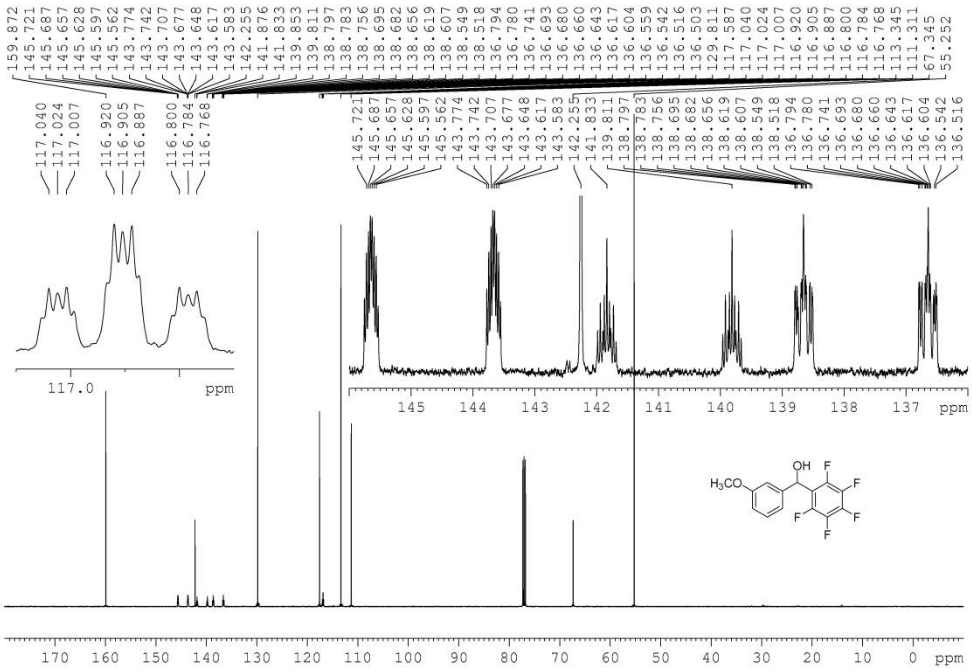

Compound 3j: [19] F NMR spectrum (470 MHz, CDCl3).

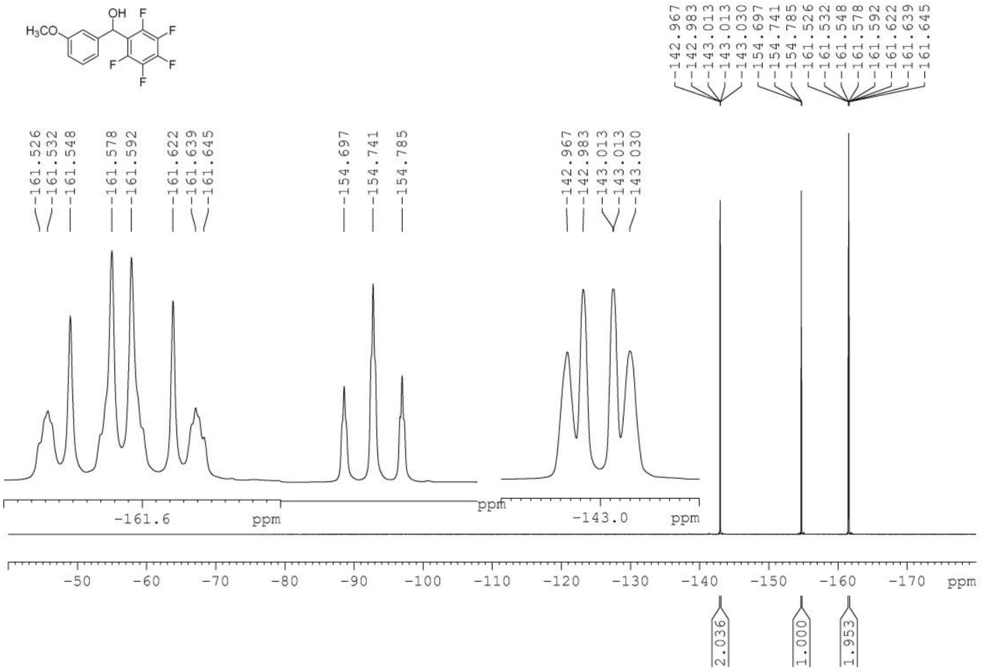

Compound 3k: [1] H NMR spectrum (500 MHz, CDCl3).

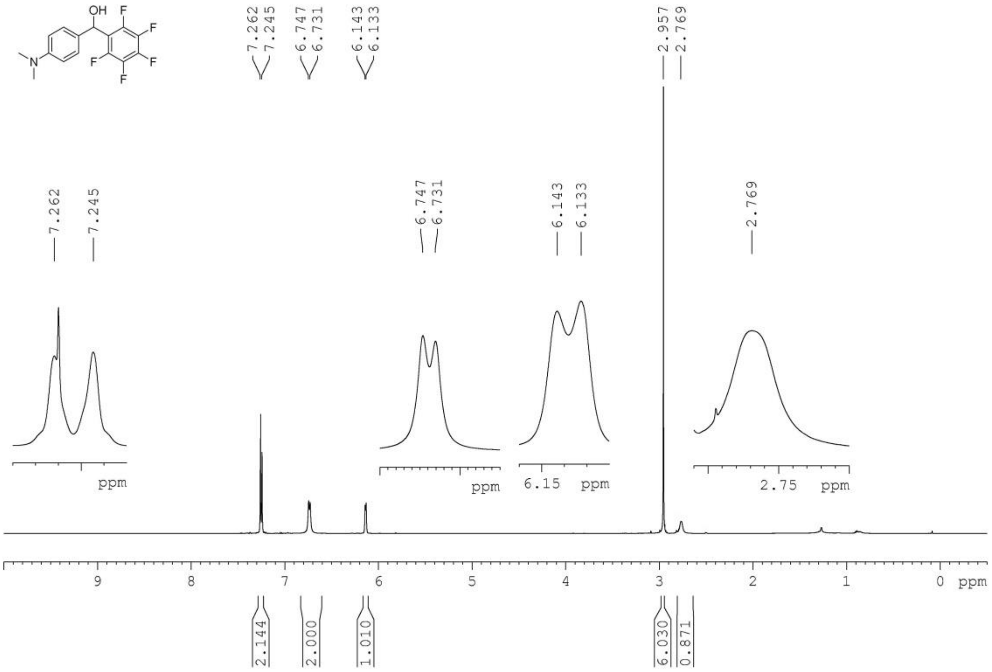

Compound 3k： [13] C{^{1}H} NMR spectrum (125 MHz, CDCl3).

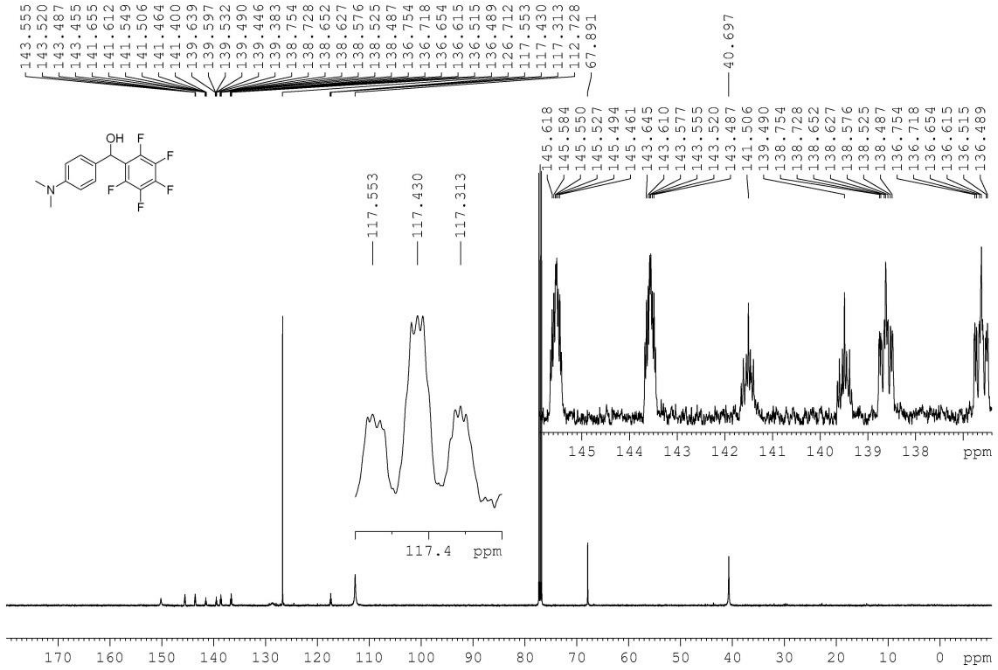

Compound 3k： [19] F NMR spectrum (470 MHz, CDCl3).

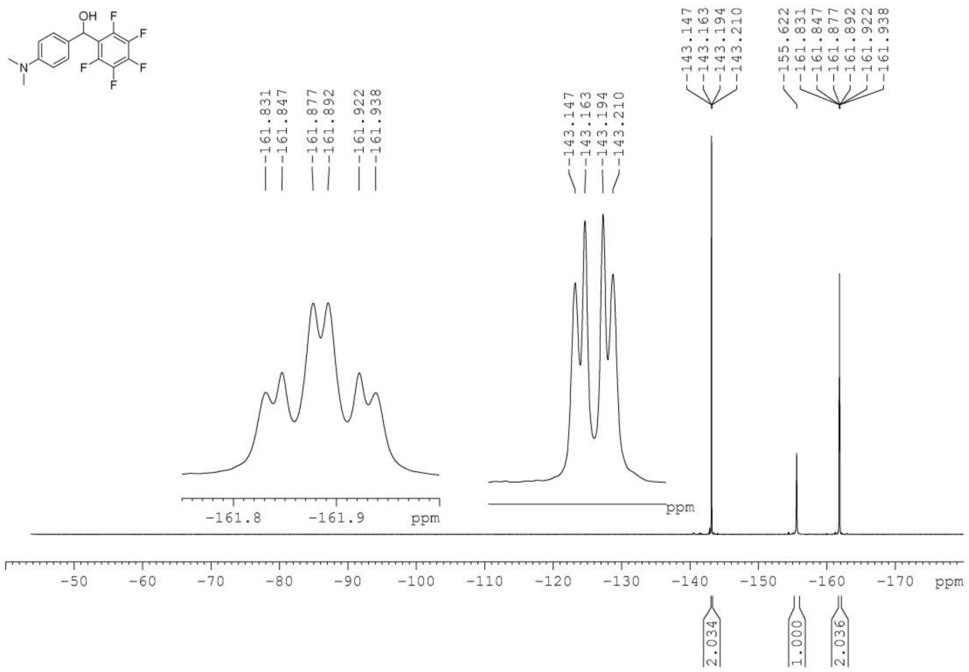

Compound 3l: [1] H NMR spectrum (500 MHz, $CDCl_3$).

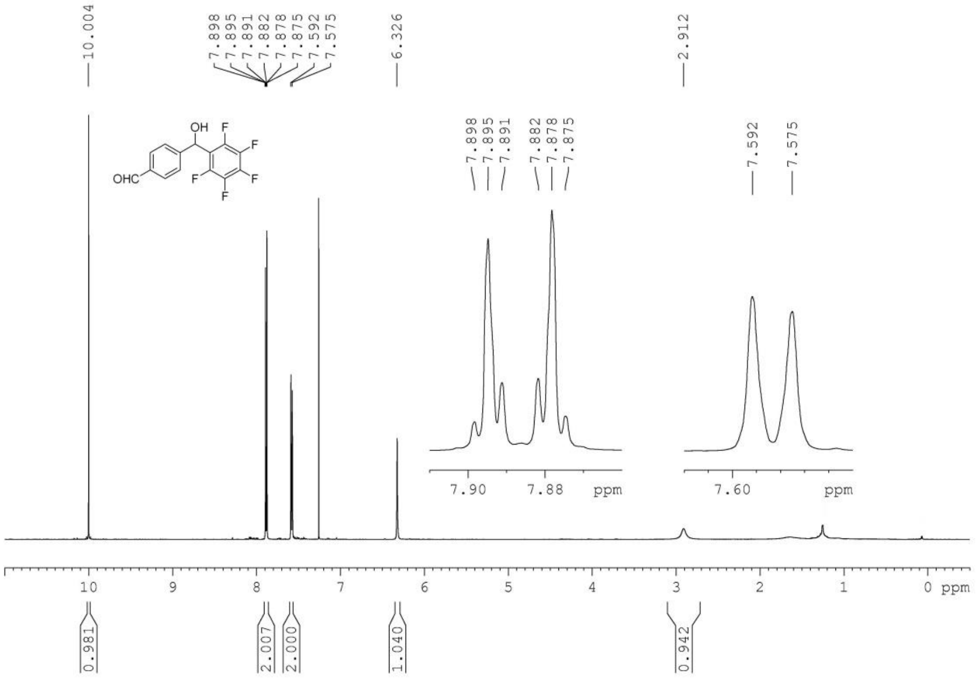

Compound 3l: [13] C{^{1}H} NMR spectrum (125 MHz, $CDCl_3$).

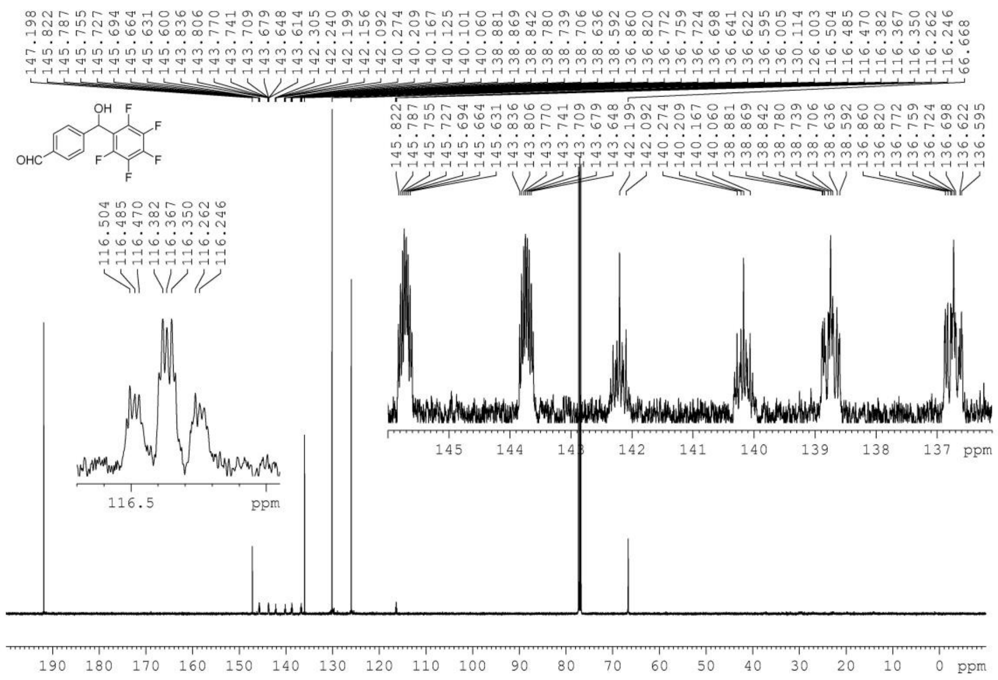

Compound 3l: [19] F NMR spectrum (470 MHz, $CDCl_3$).

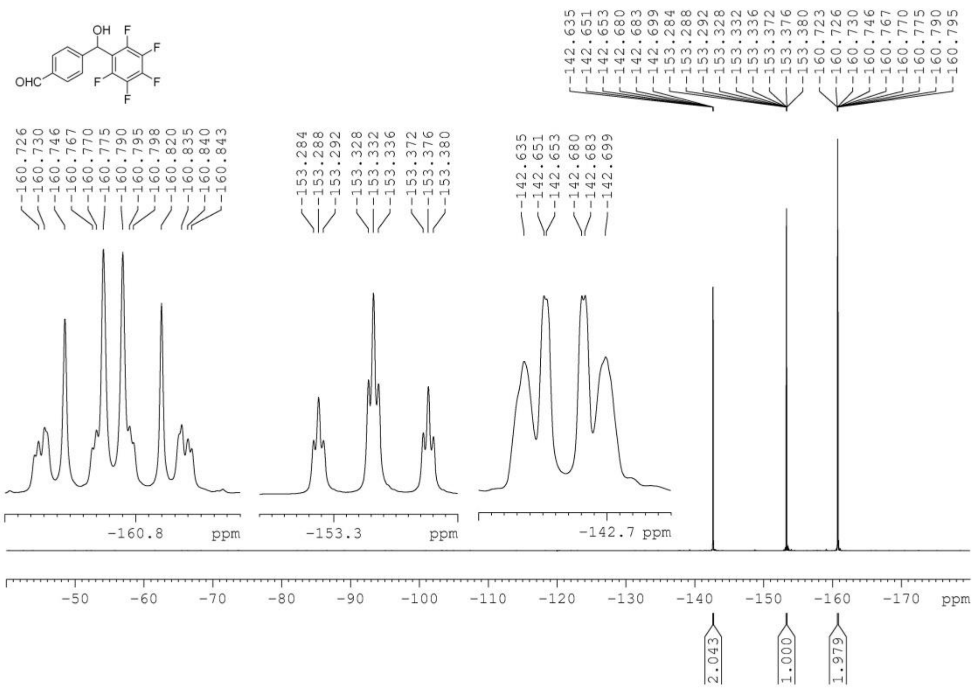

Compound 3m: [1] H NMR spectrum (500 MHz, $CDCl_3$).

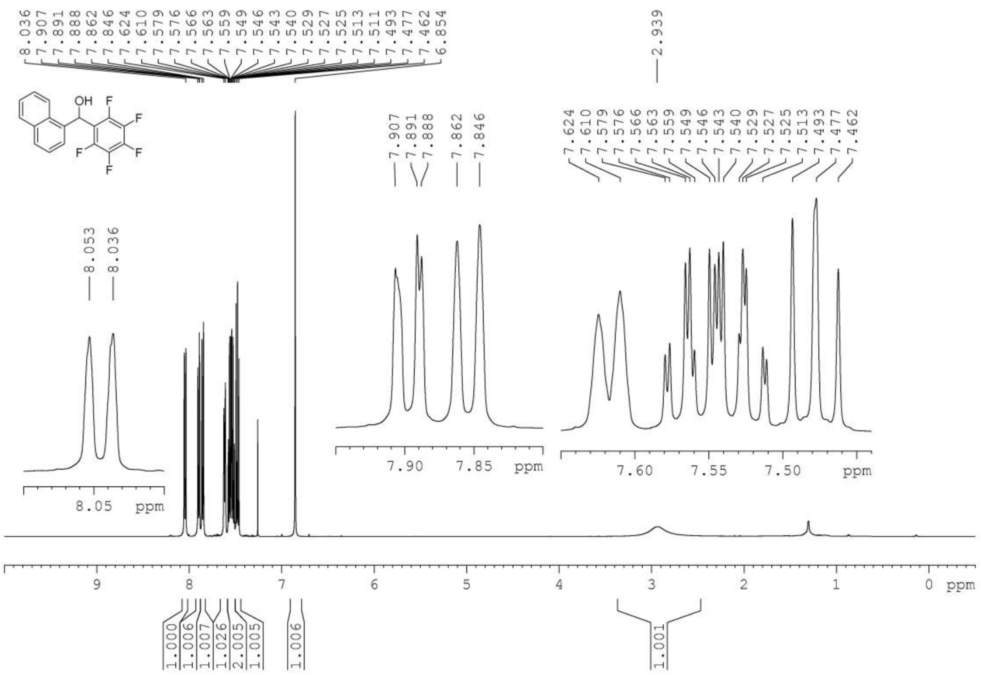

Compound 3m: ^{13} C{^{1}H} NMR spectrum (125 MHz, $CDCl_3$).

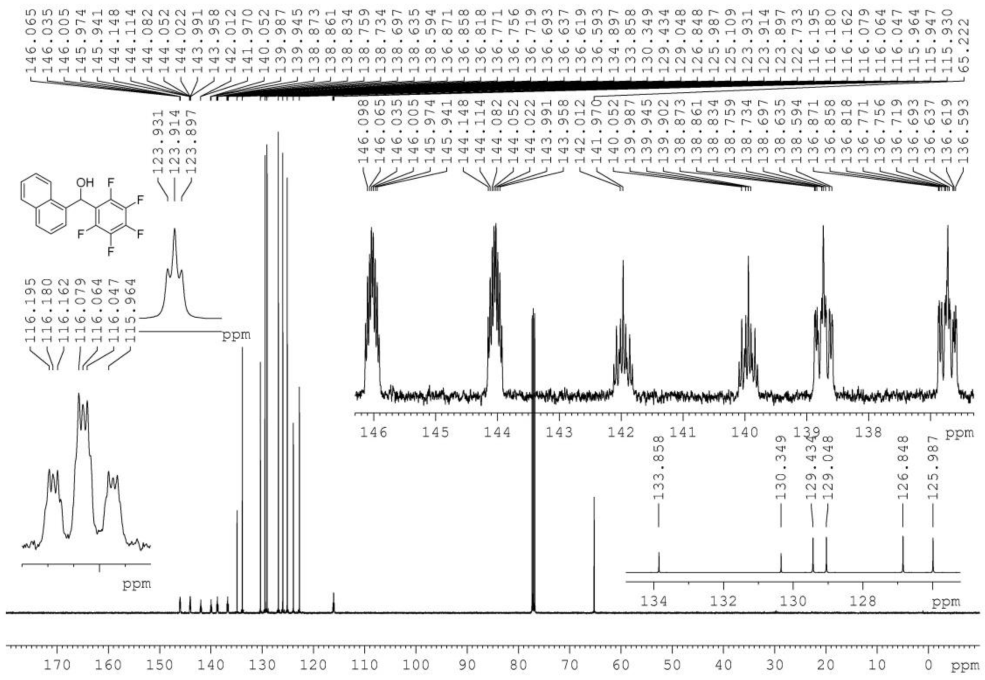

Compound 3m: ^{19} F NMR spectrum (470 MHz, $CDCl_3$).

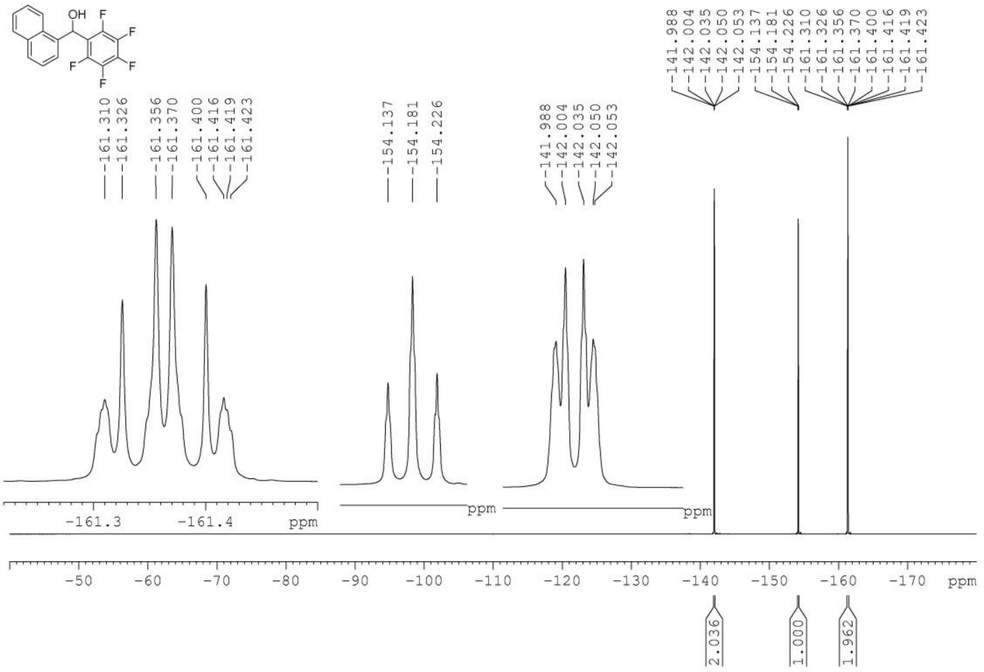

Compound 3n: ^{1}H NMR spectrum (500 MHz, $CDCl_3$).

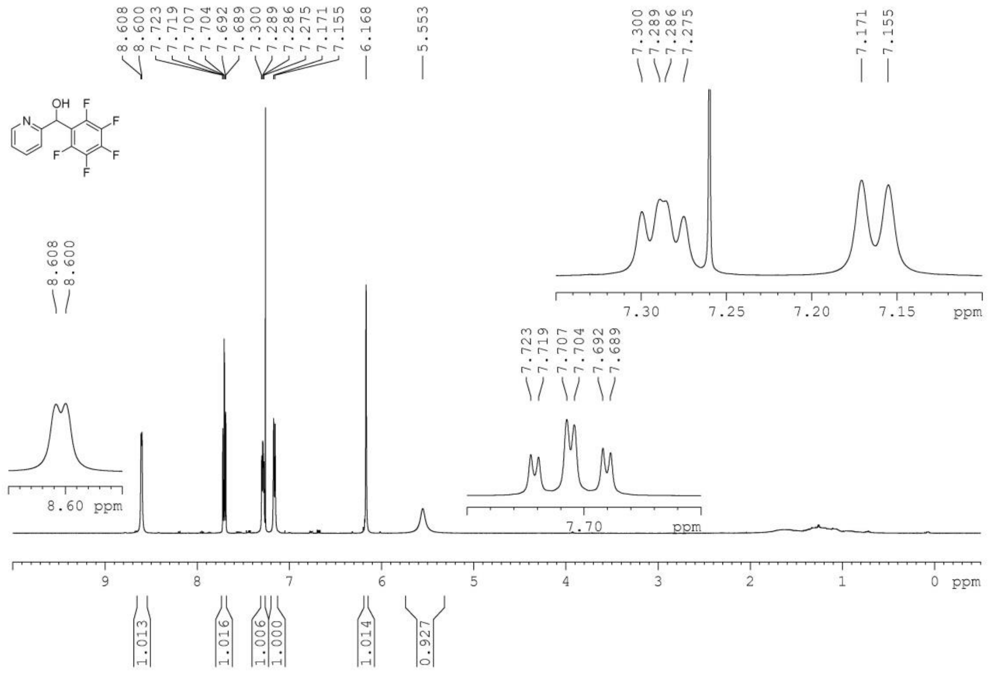

Compound 3n: $^{13}C\{^{1}H\}$ NMR spectrum (125 MHz, $CDCl_3$).

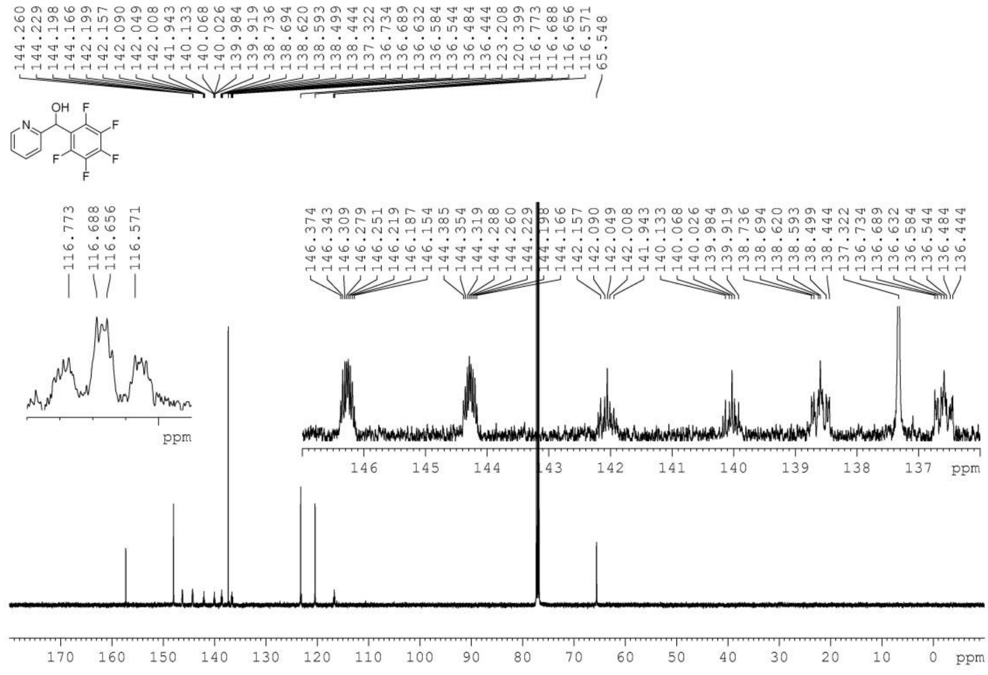

Compound 3n： [19] F NMR spectrum (470 MHz, $CDCl_3$).

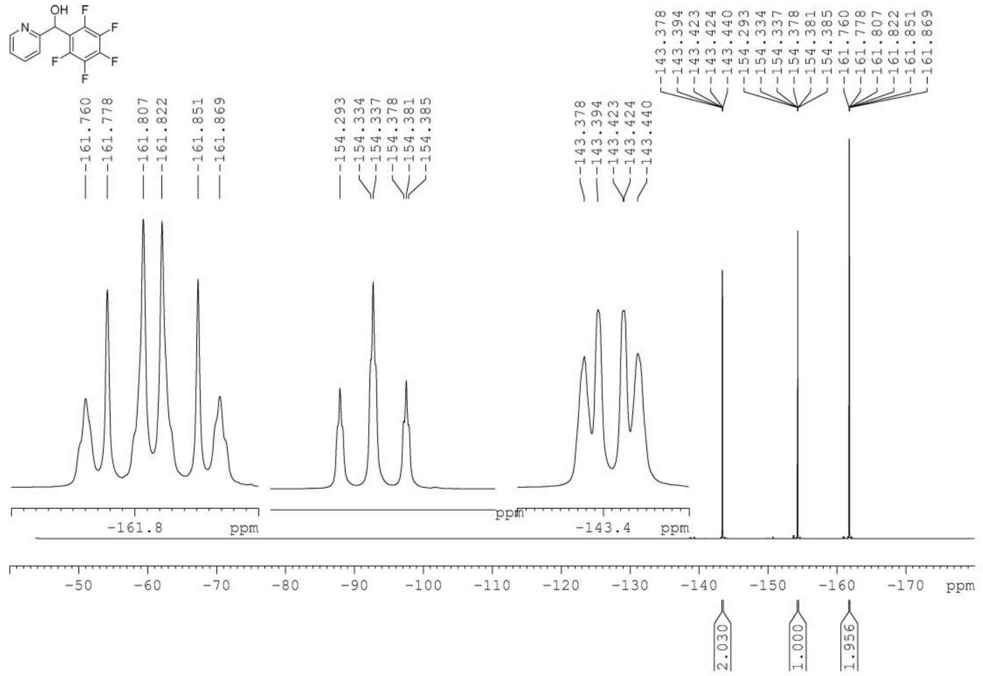

Compound 3o： [1] H NMR spectrum (500 MHz, $CDCl_3$).

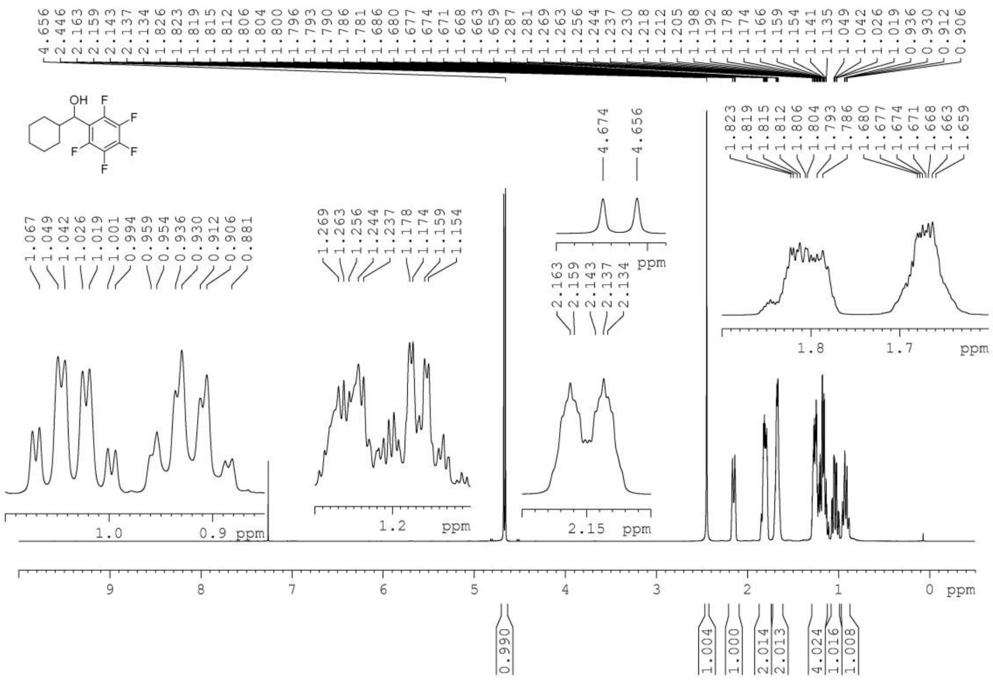

Compound 3o： 13 C{1H} NMR spectrum (125 MHz, CDCl$_3$).

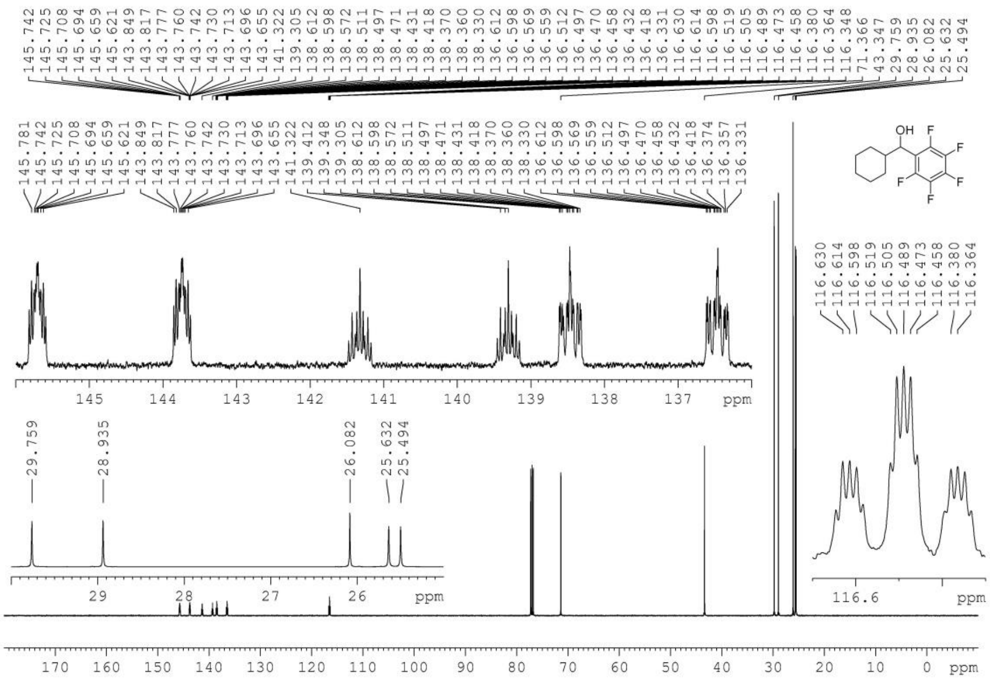

Compound 3o： 19 F NMR spectrum (470 MHz, CDCl$_3$).

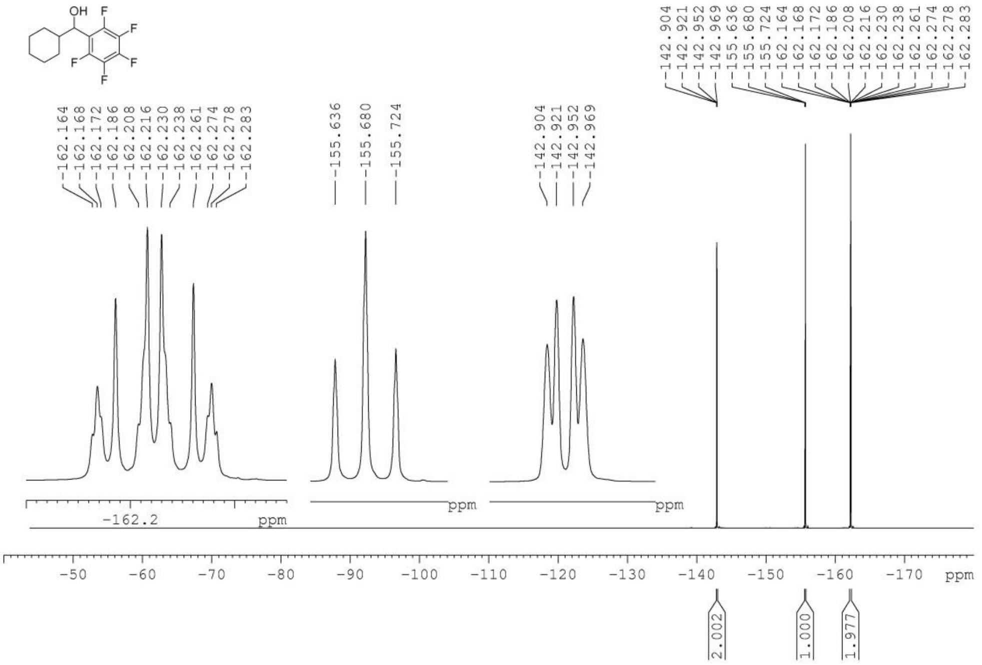

Compound 3p： [1] H NMR spectrum (500 MHz, $CDCl_3$).

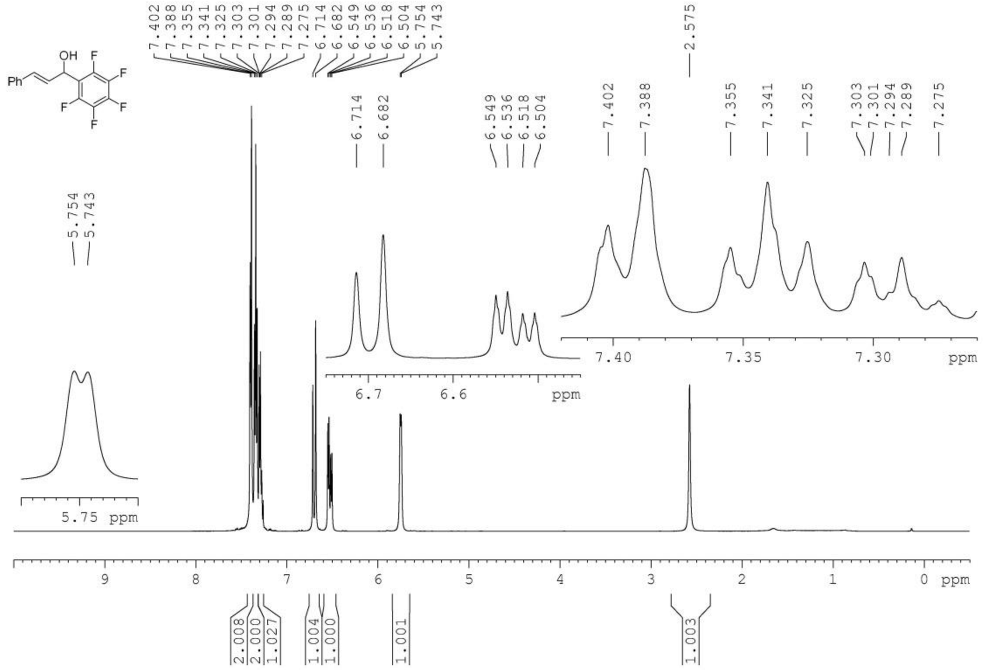

Compound 3p： [13] C{^{1}H} NMR spectrum (125 MHz, $CDCl_3$).

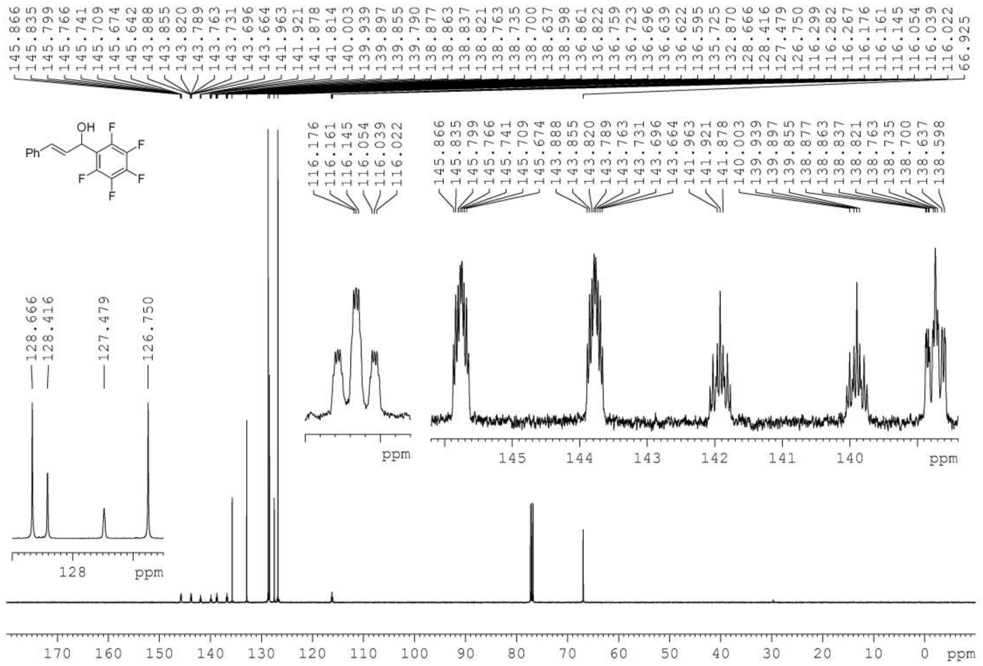

Compound 3p: [19] F NMR spectrum (470 MHz, $CDCl_3$).

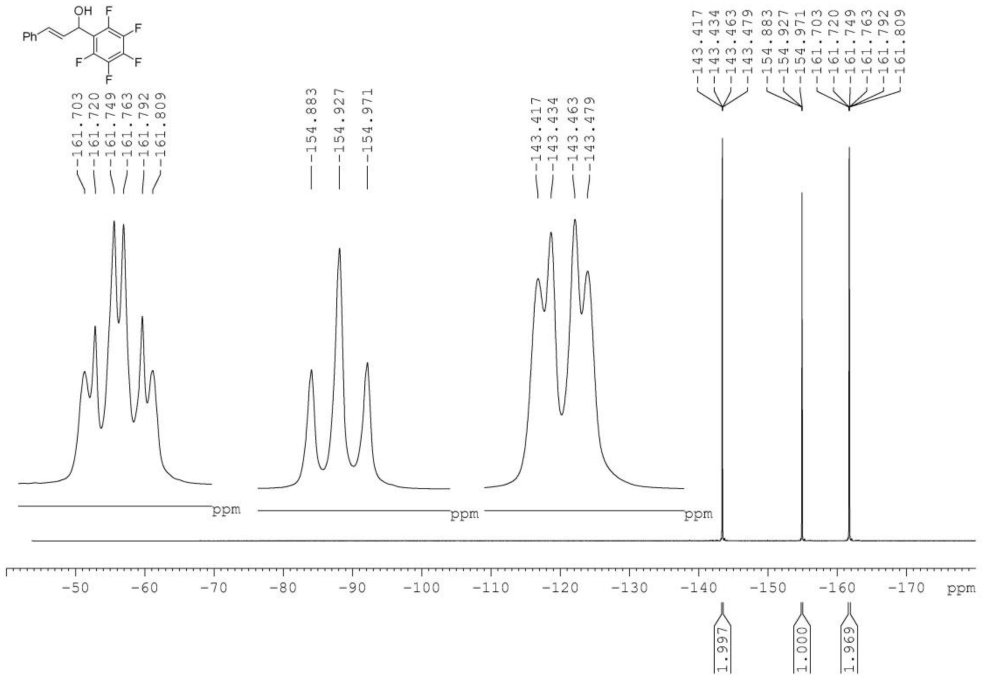

Compound 3q: [1] H NMR spectrum (500 MHz, $CDCl_3$).

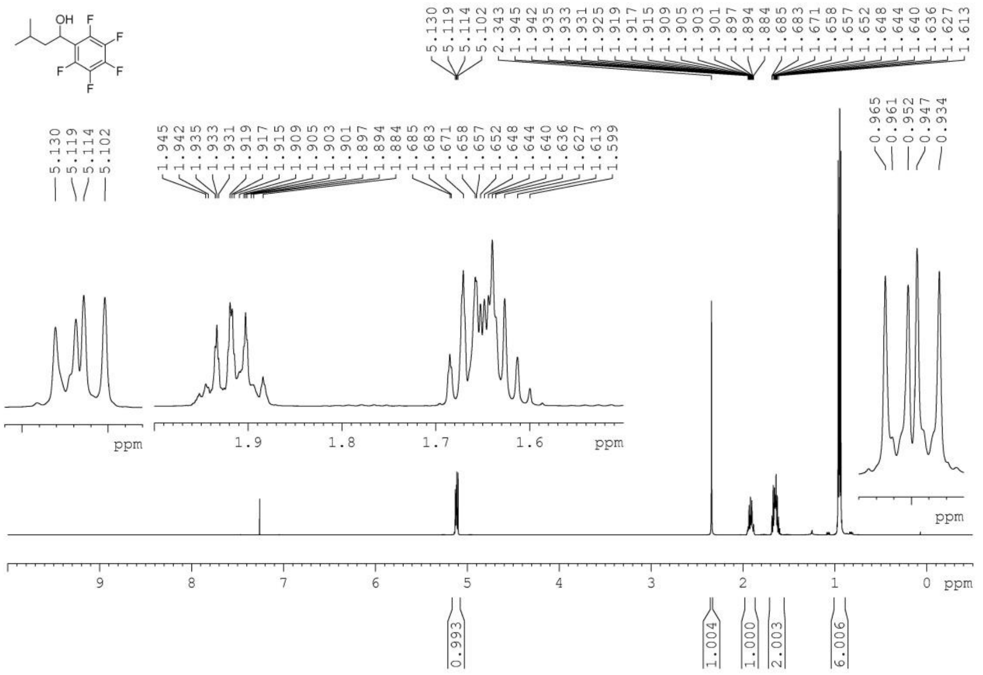

Compound 3q: [13] C{^{1}H} NMR spectrum (125 MHz, $CDCl_3$).

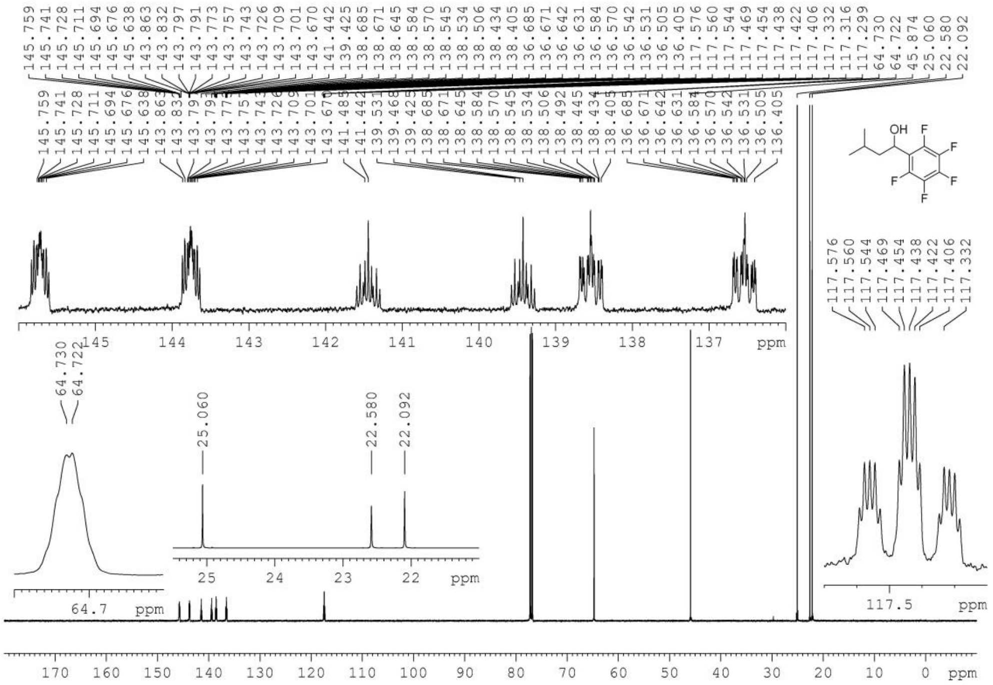

Compound 3q: [19] F NMR spectrum (470 MHz, $CDCl_3$).

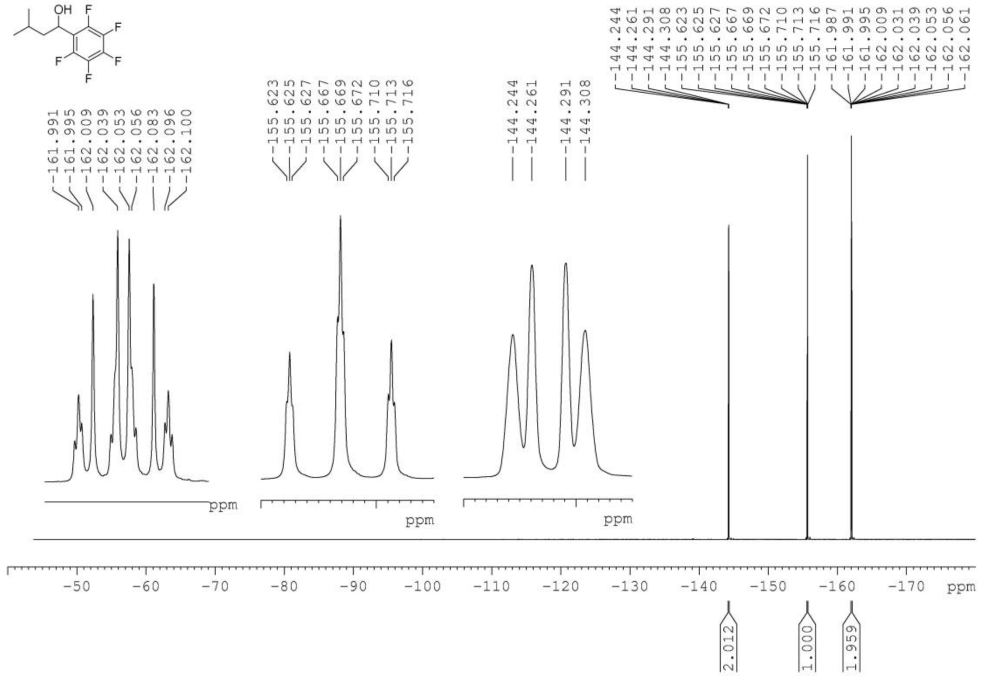

Compound 3r： 1 H NMR spectrum (500 MHz, $CDCl_3$).

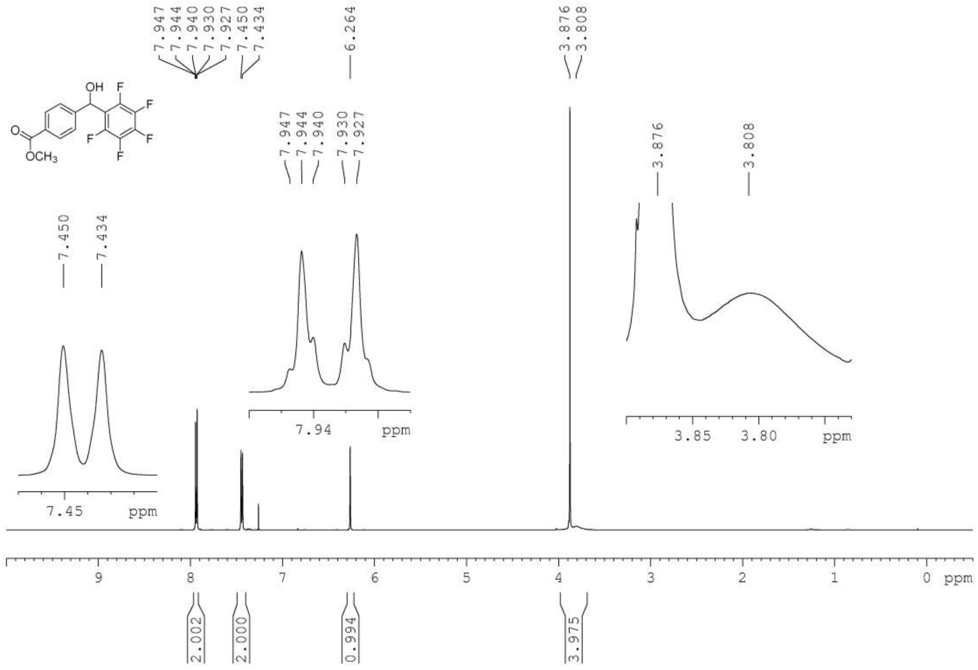

Compound 3r： 13 C{1H} NMR spectrum (125 MHz, $CDCl_3$).

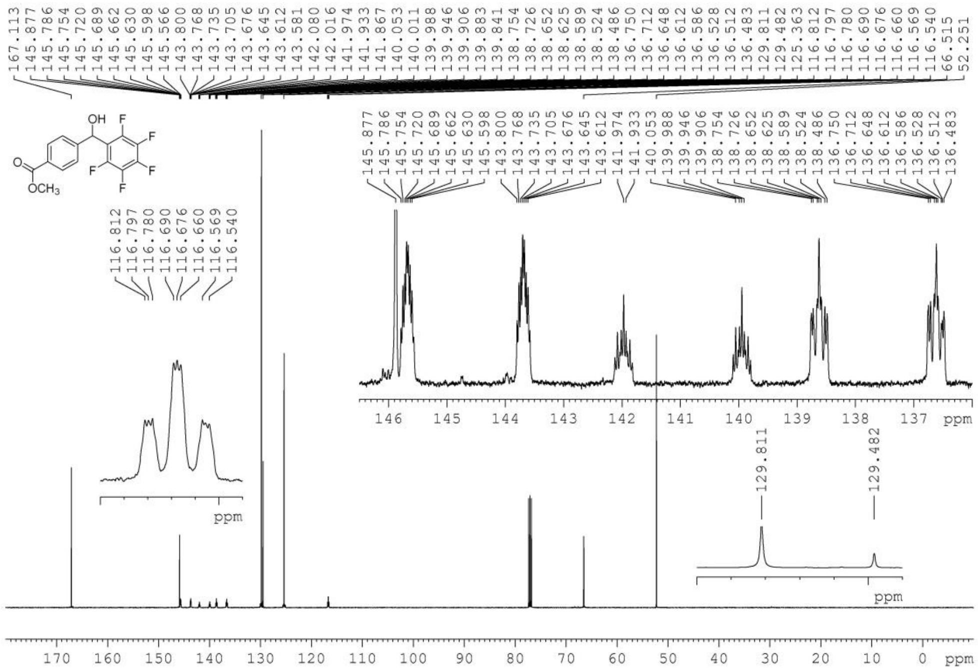

Compound 3r: [19] F NMR spectrum (470 MHz, $CDCl_3$).

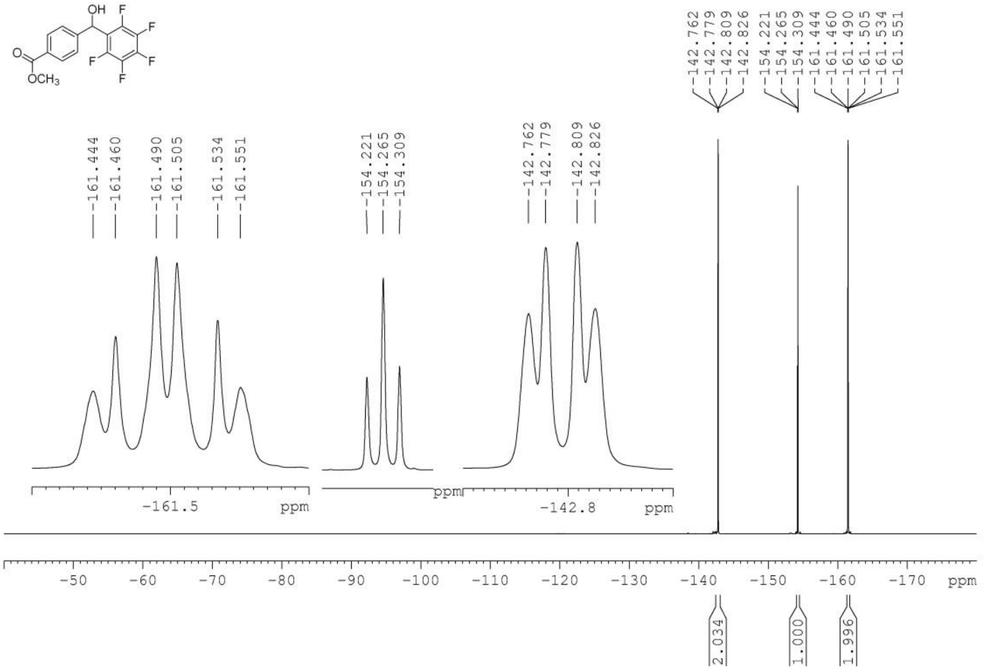

Compound 3s: [1] H NMR spectrum (500 MHz, $CDCl_3$).

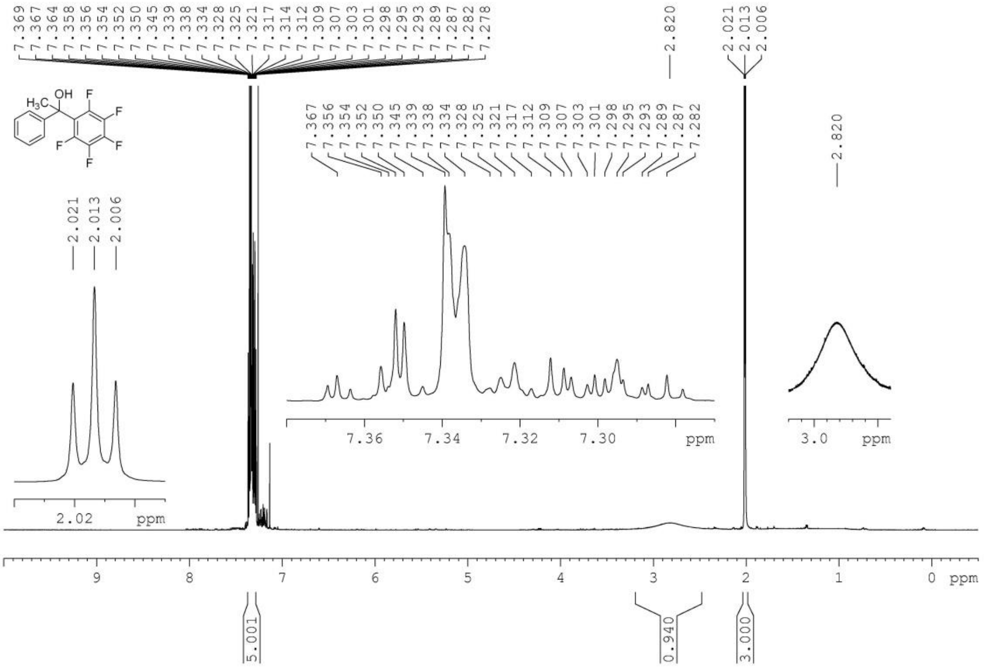

Compound 3s: $^{13}C\{^1H\}$ NMR spectrum (125 MHz, $CDCl_3$).

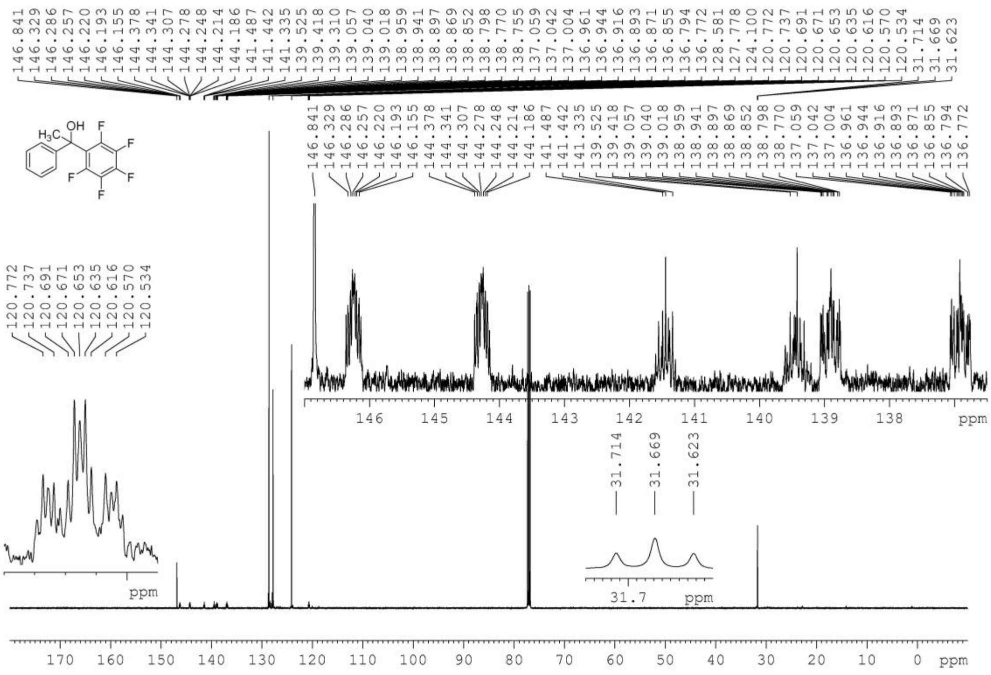

Compound 3s: ^{19}F NMR spectrum (470 MHz, $CDCl_3$).

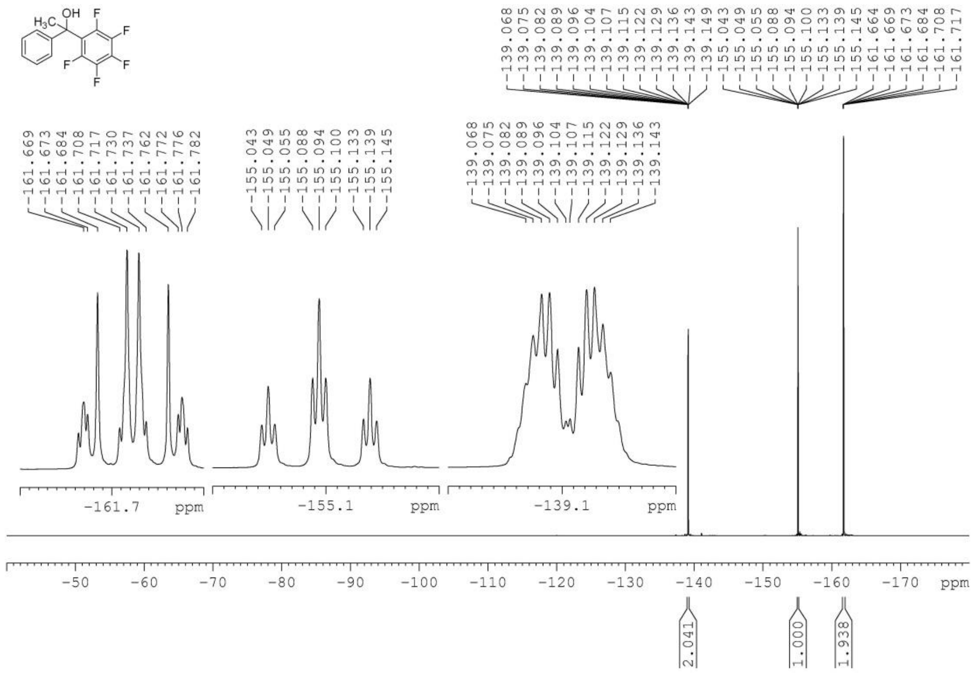

Compound 3t： [1] H NMR spectrum (500 MHz, CDCl3).

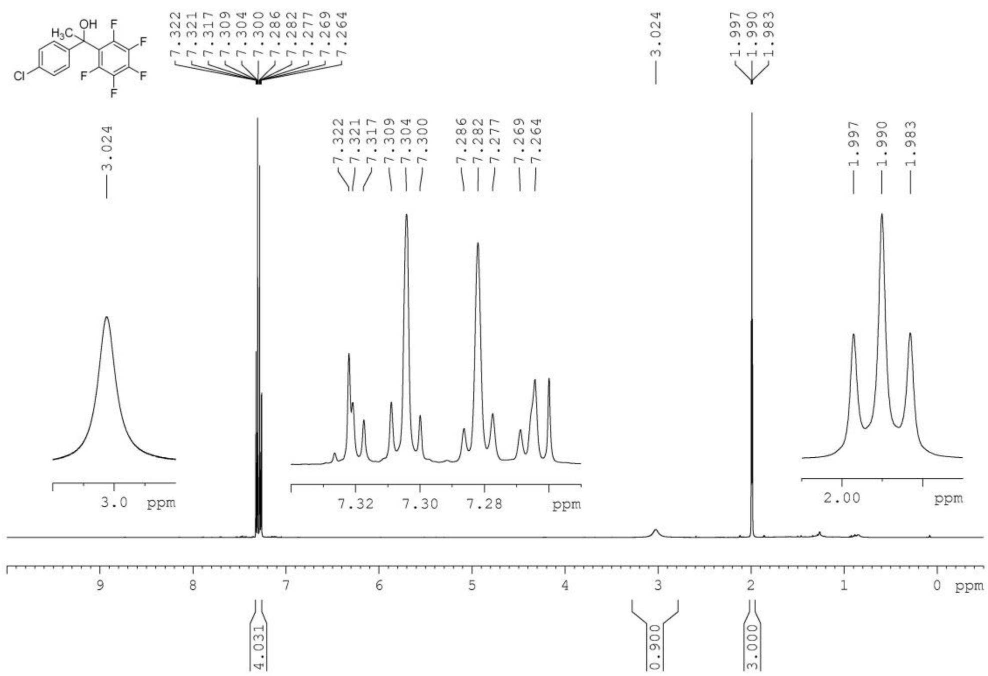

Compound 3t： [13] C{^{1}H} NMR spectrum (125 MHz, CDCl3).

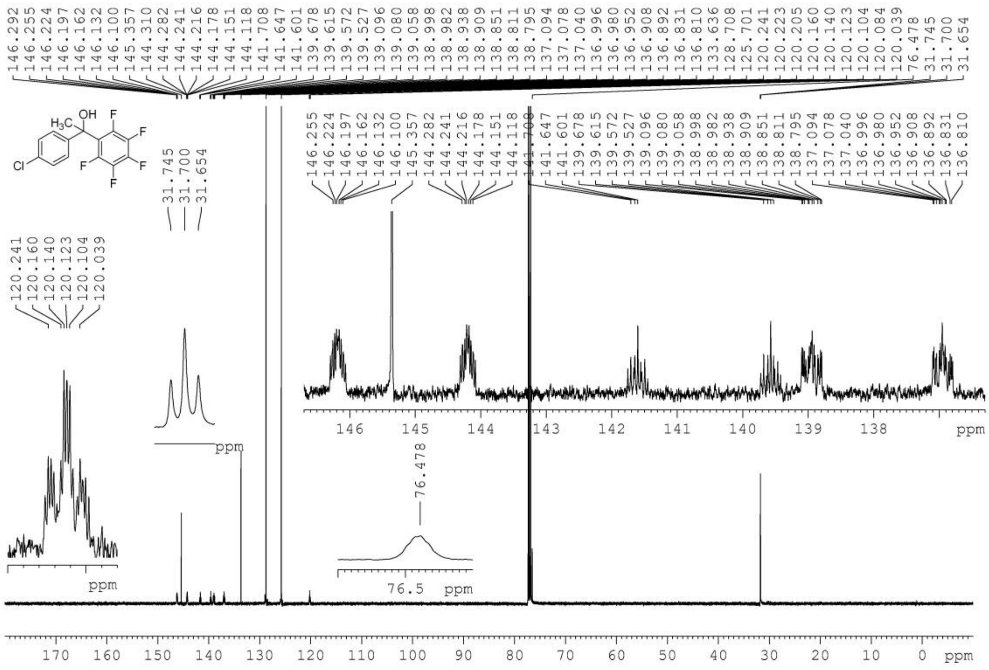

Compound 3t： 19 F NMR spectrum (470 MHz, $CDCl_3$).

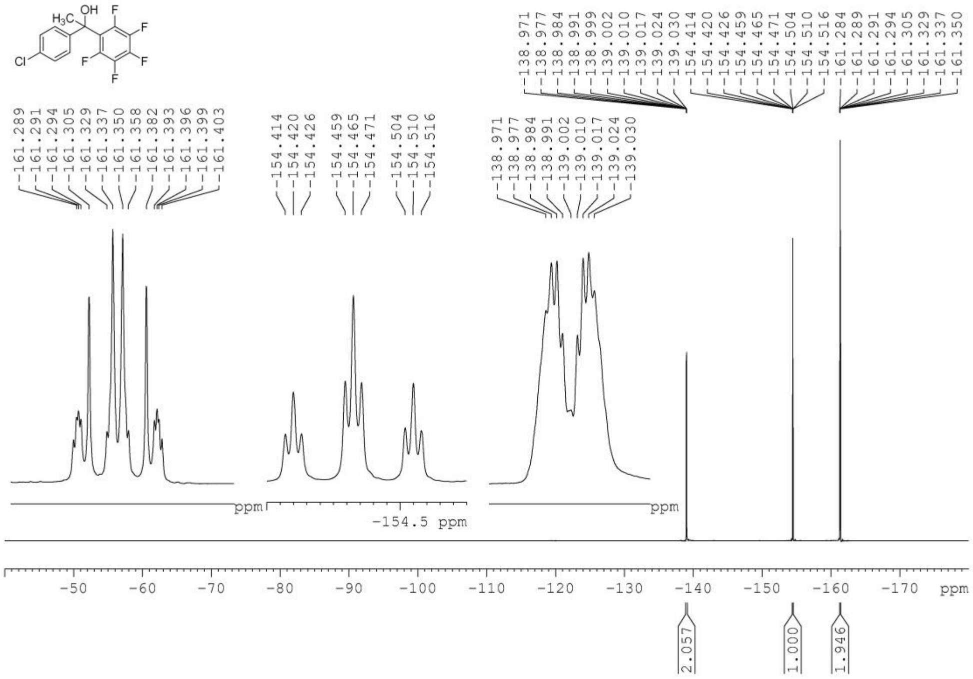

Compound 3u： 1 H NMR spectrum (500 MHz, $CDCl_3$).

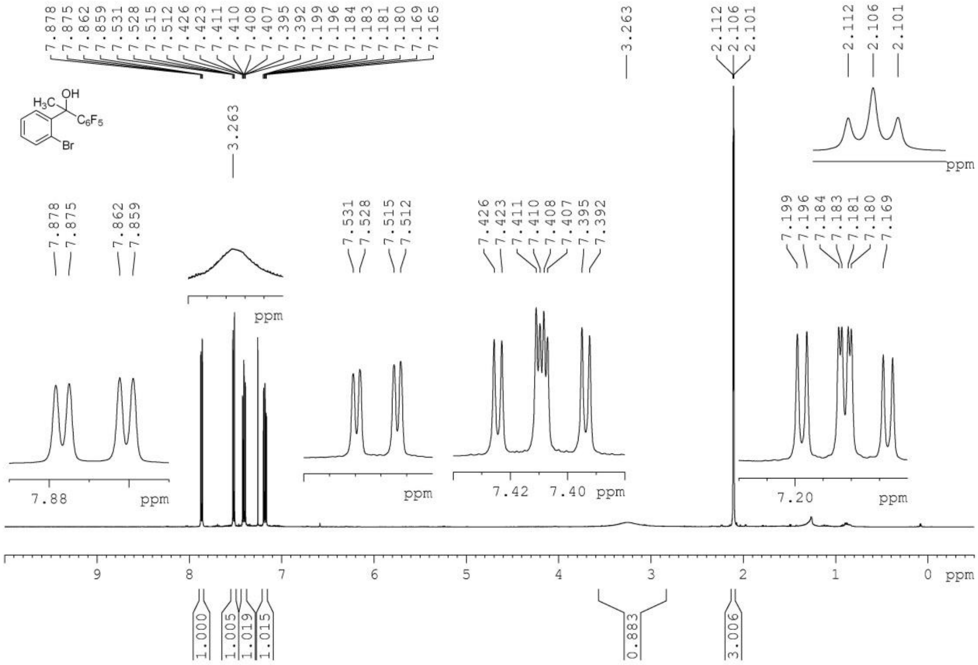

Compound 3u：[13] C{^{1}H} NMR spectrum (125 MHz, CDCl3).

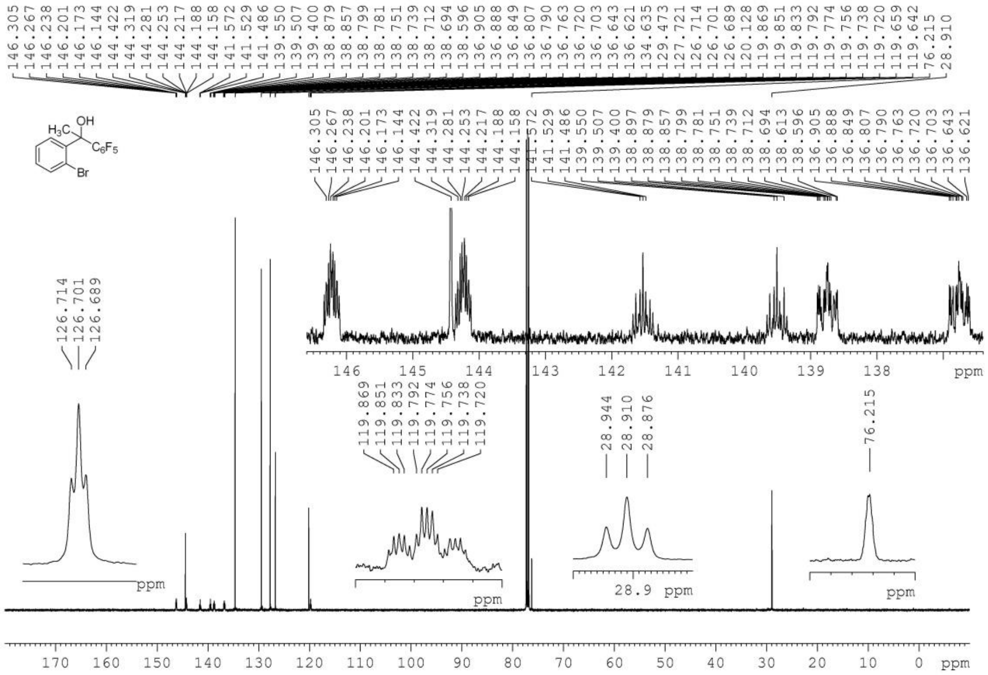

Compound 3u：[19] F NMR spectrum (470 MHz, CDCl3).

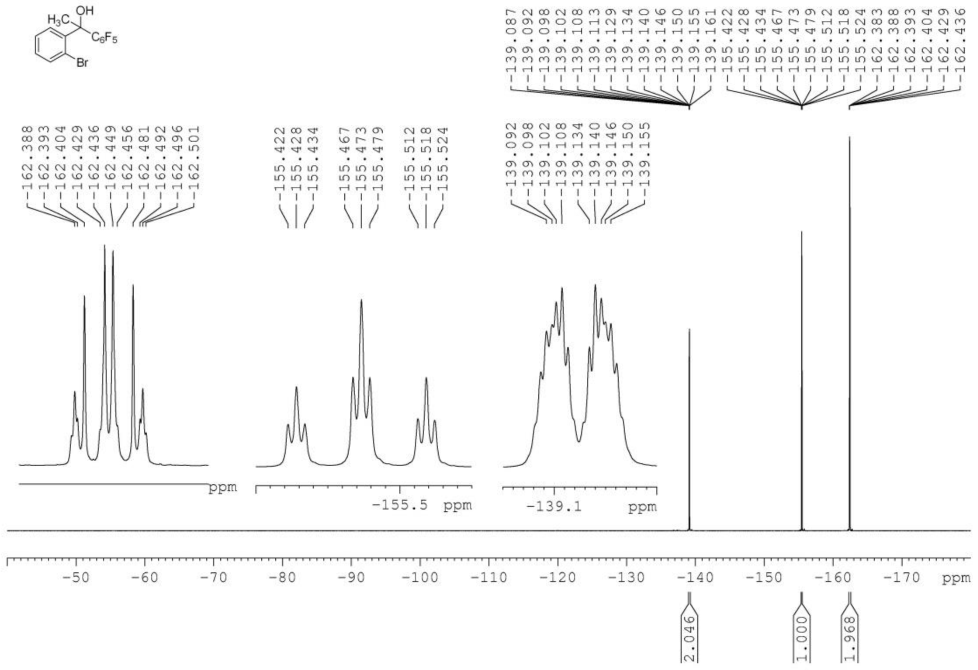

Compound 3v: [1] H NMR spectrum (500 MHz, $CDCl_3$).

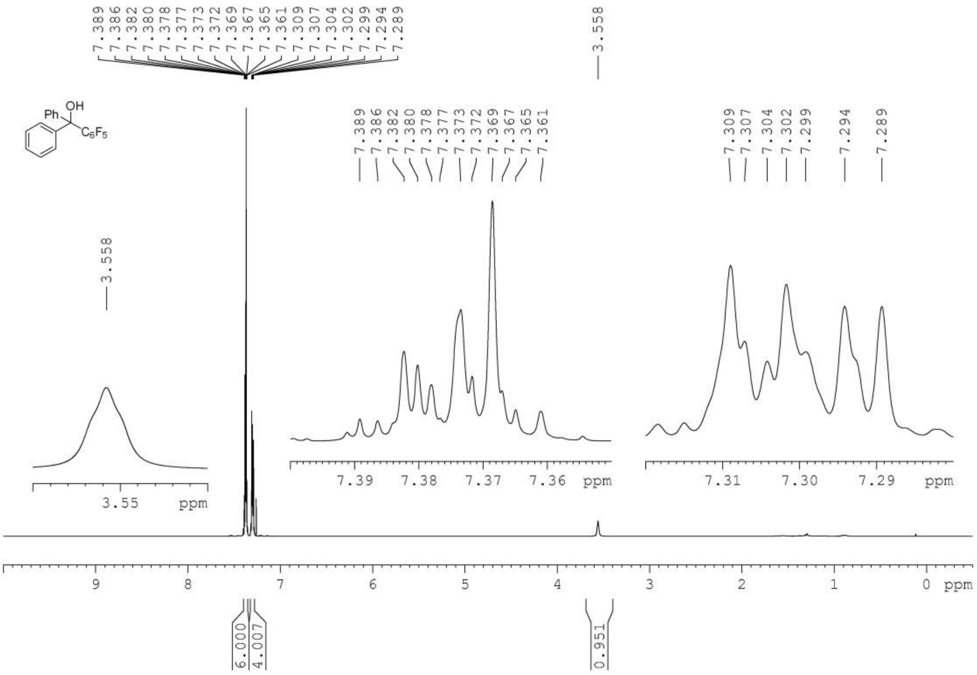

Compound 3v: [13] C{^{1}H} NMR spectrum (125 MHz, $CDCl_3$).

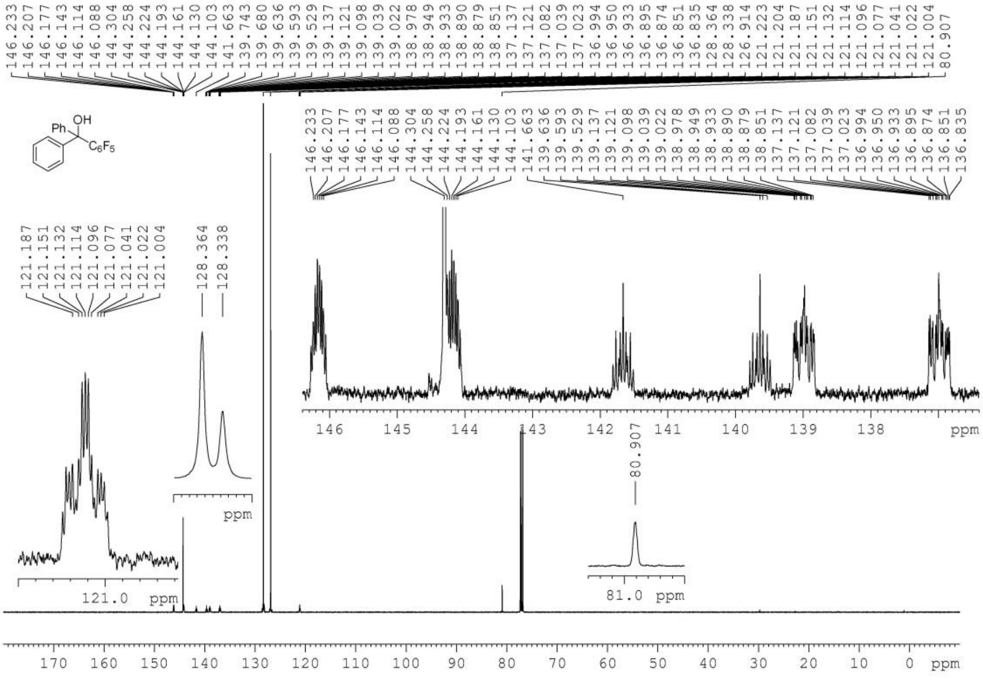

Compound 3v: [19] F NMR spectrum (470 MHz, CDCl3).

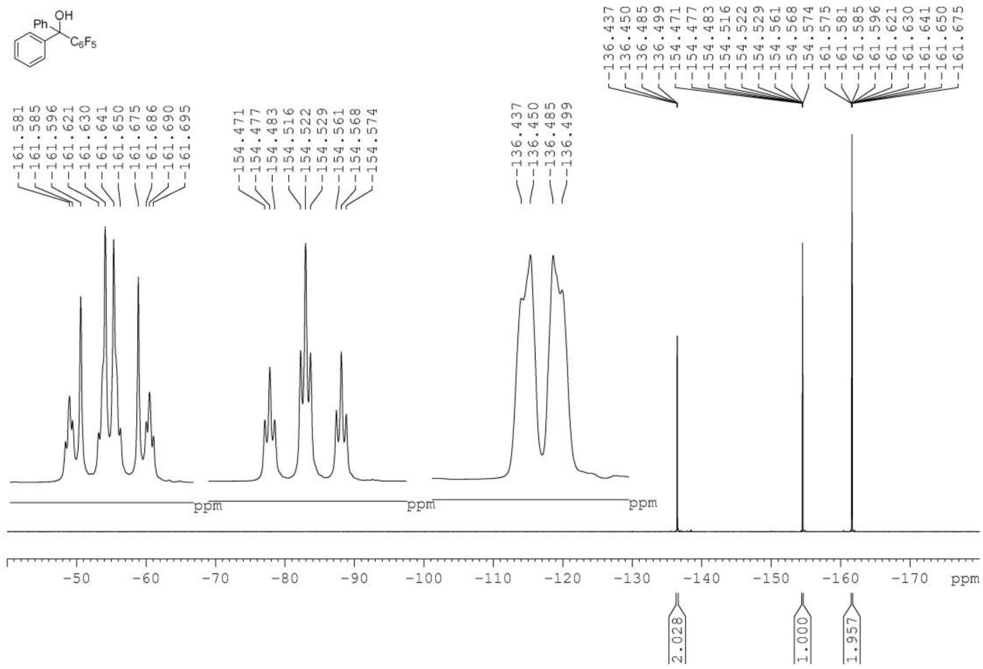

Compound 3w: [1] H NMR spectrum (500 MHz, CDCl3).

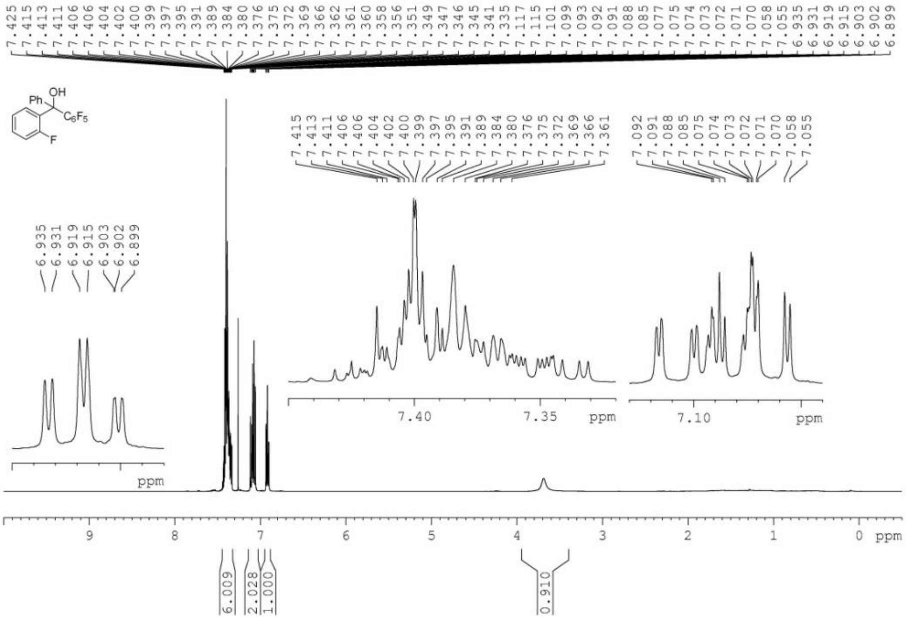

Compound 3w: ^{13}C{^{1}H} NMR spectrum (125 MHz, CDCl$_3$).

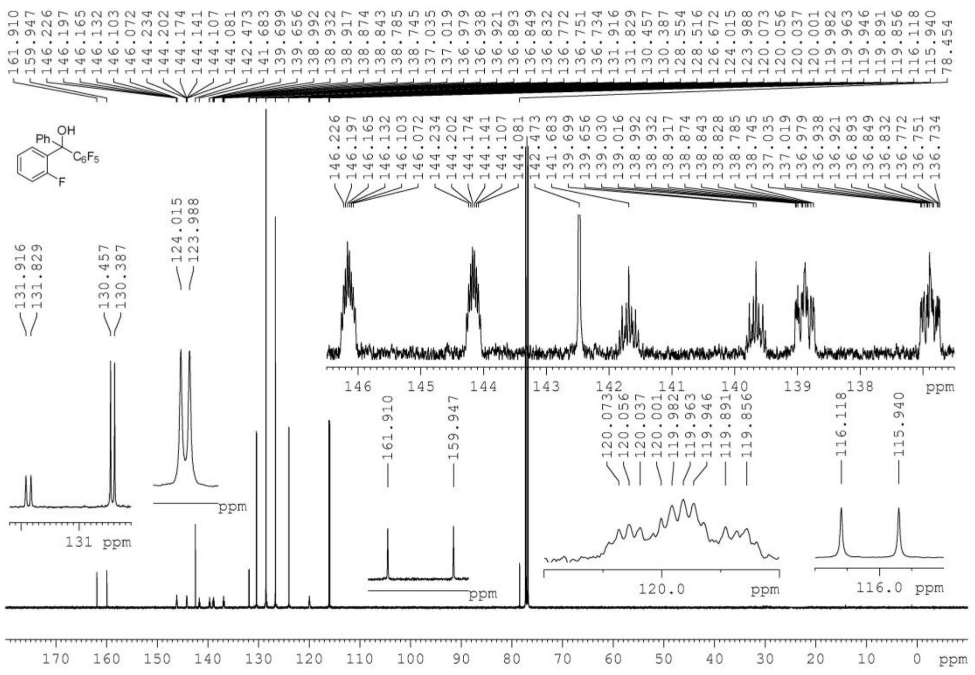

Compound 3w: ^{19}F NMR spectrum (470 MHz, CDCl$_3$).

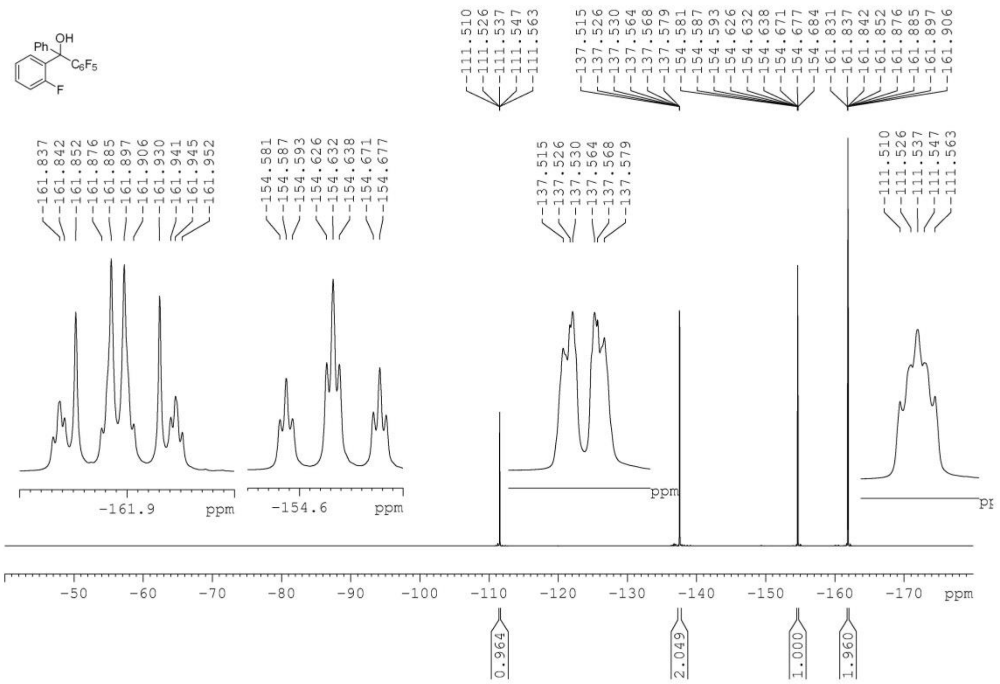

Compound 3x: [1] H NMR spectrum (500 MHz, $CDCl_3$).

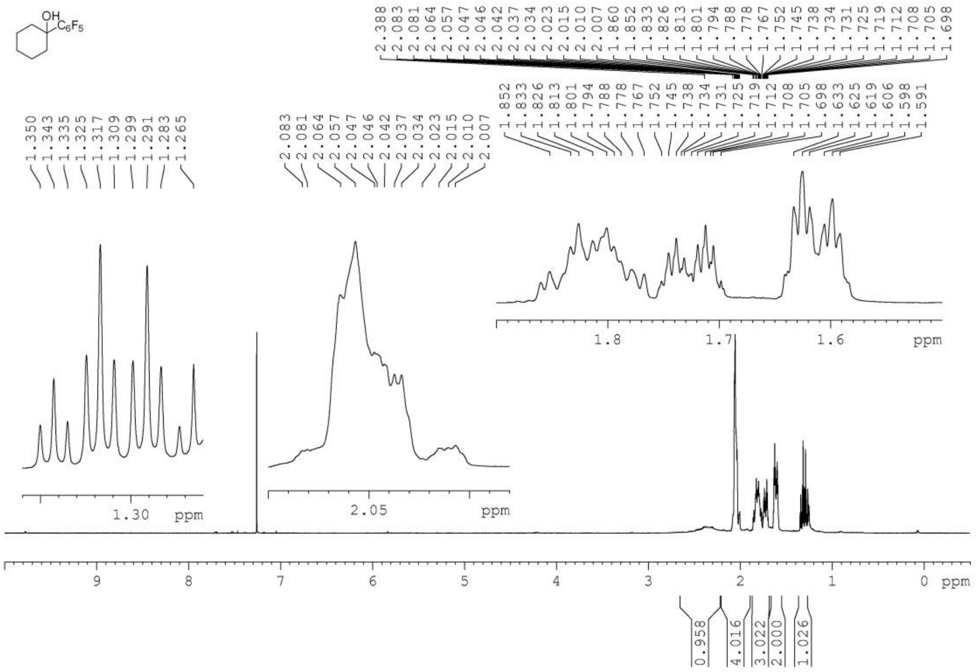

Compound 3x: [13] C{^{1}H} NMR spectrum (125 MHz, $CDCl_3$).

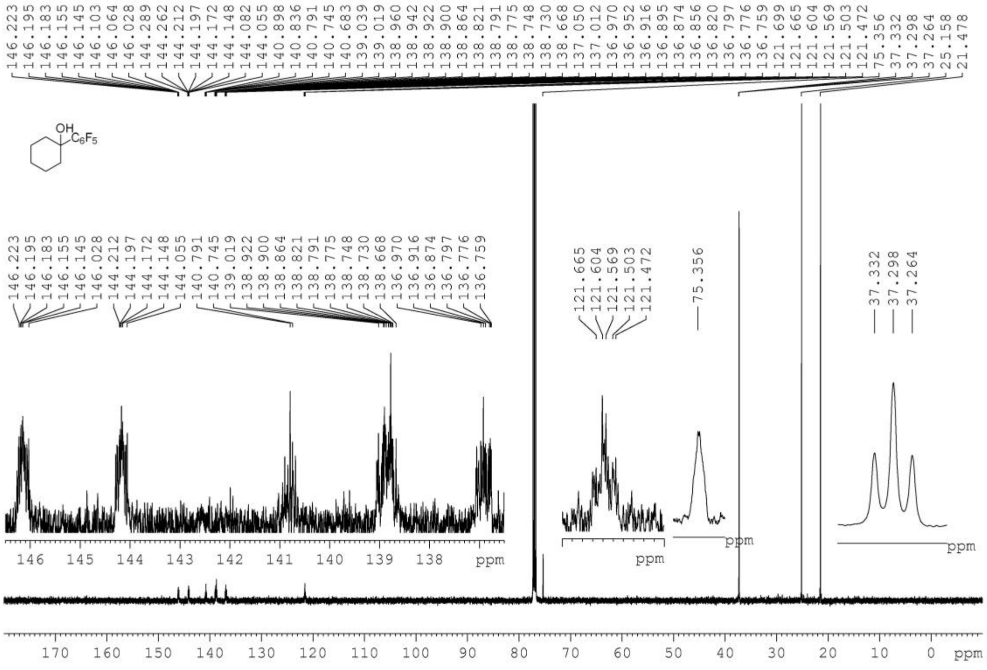

Compound 3x: [19] F NMR spectrum (470 MHz, CDCl3).

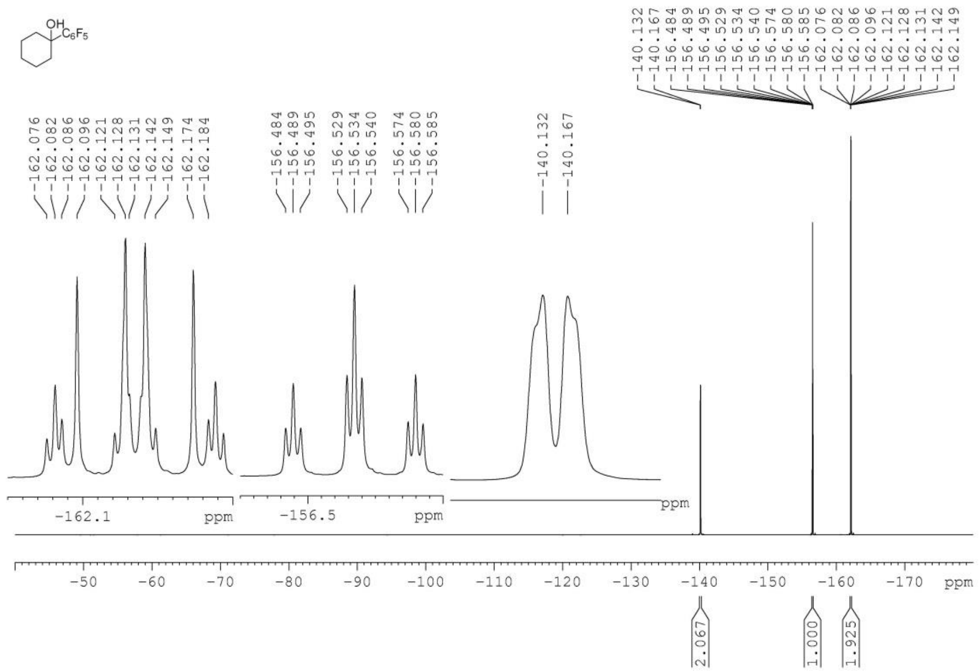

Compound 4a: [1] H NMR spectrum (500 MHz, CDCl3).

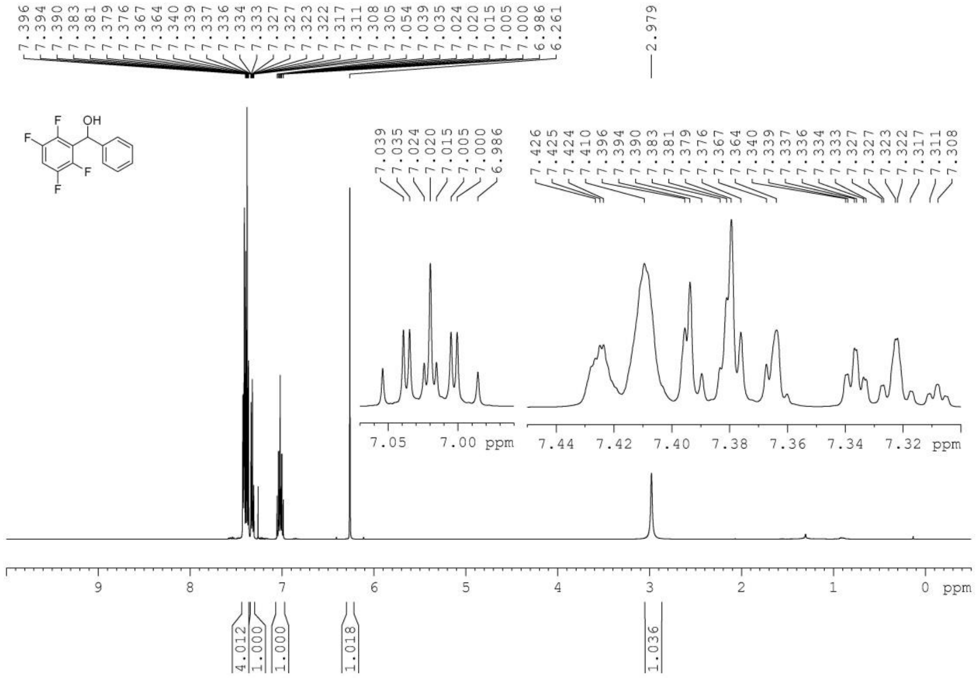

Compound 4a： 13 C{1H} NMR spectrum (125 MHz, CDCl3).

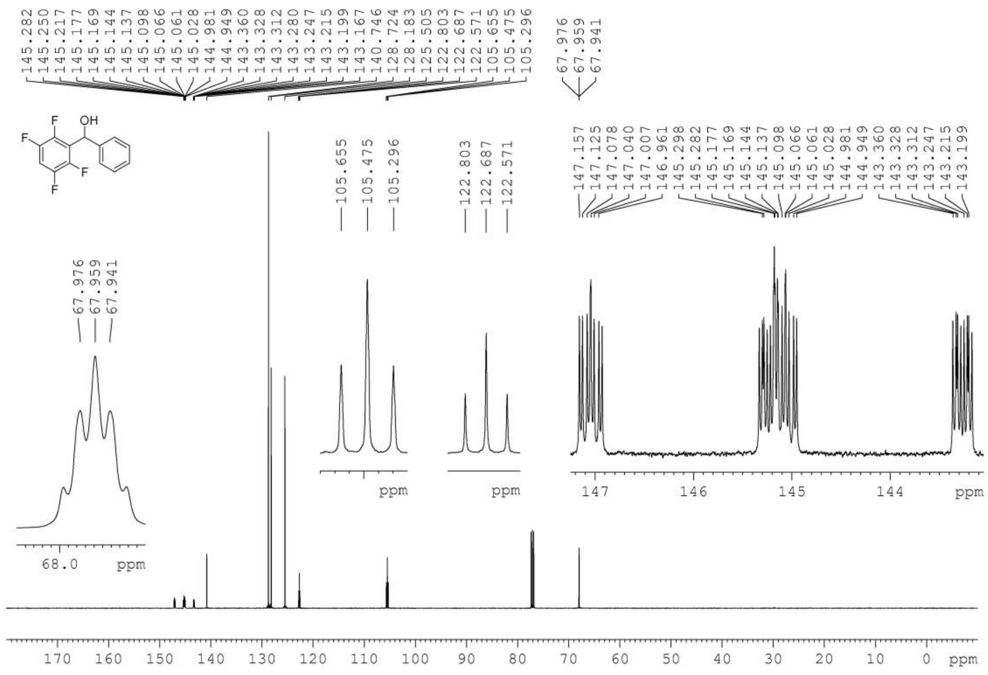

Compound 4a： 19 F NMR spectrum (470 MHz, CDCl3).

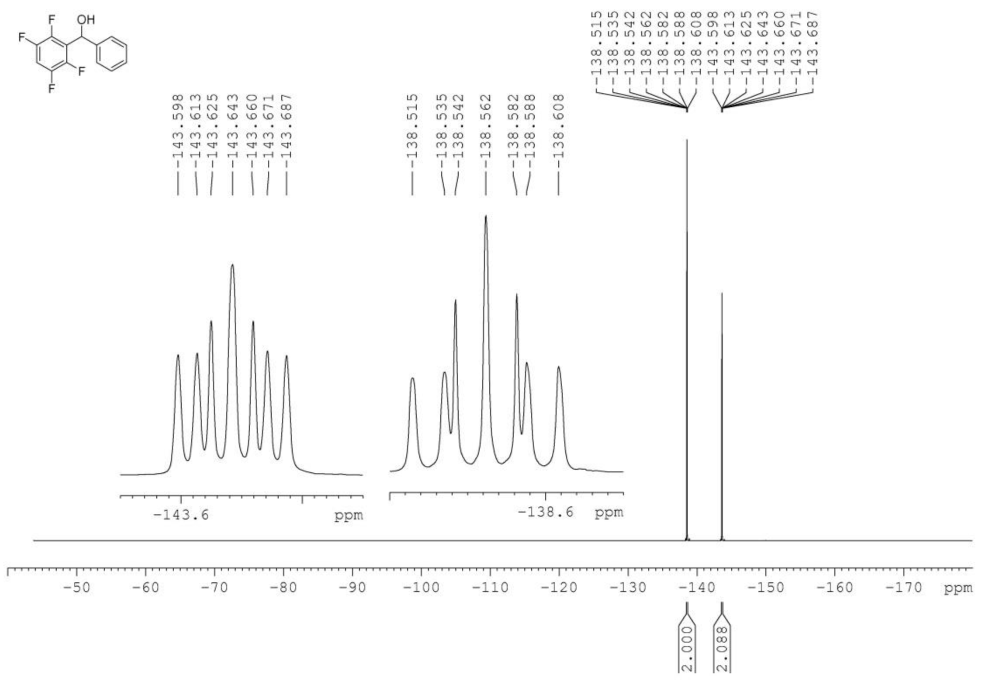

Compound 4b: [1] H NMR spectrum (500 MHz, CDCl3).

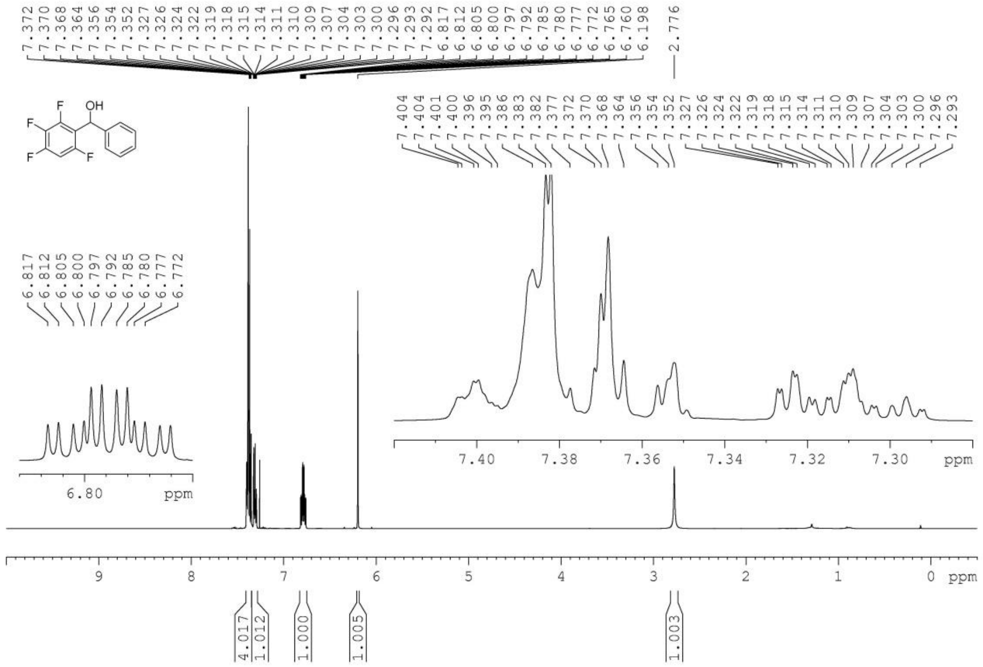

Compound 4b: [13] C{^{1}H} NMR spectrum (125 MHz, CDCl3).

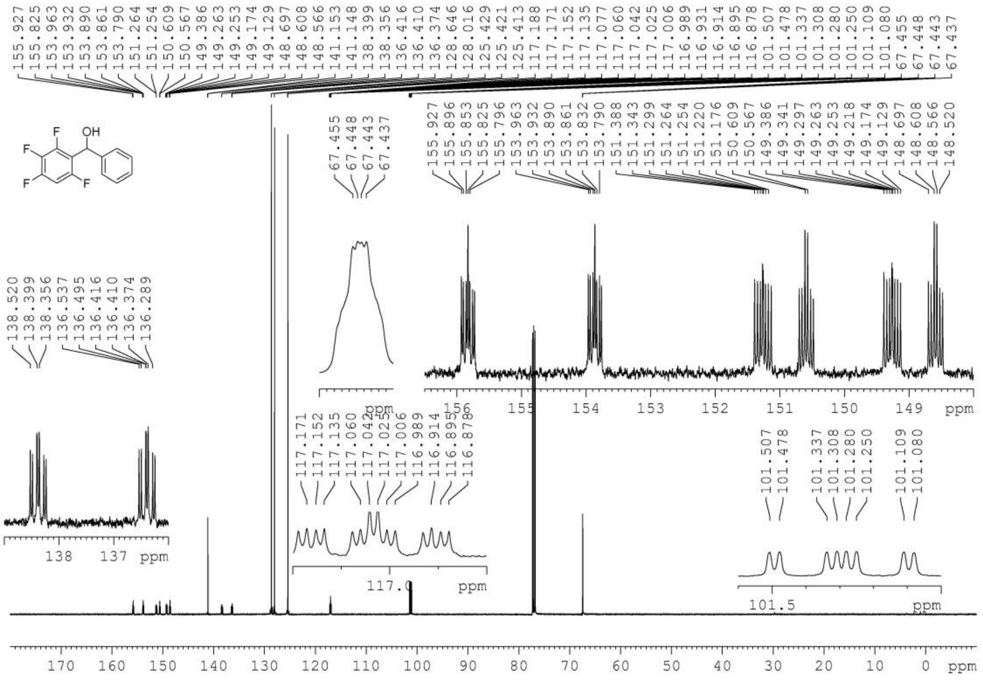

Compound 4b: [19] F NMR spectrum (470 MHz, $CDCl_3$).

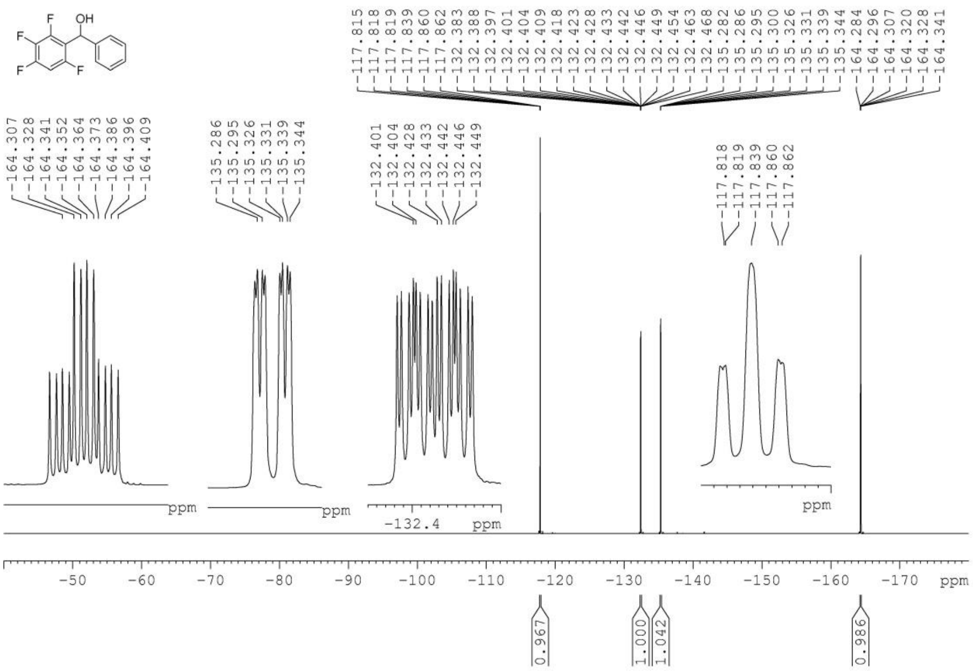

Compound 4c: [1] H NMR spectrum (500 MHz, $CDCl_3$).

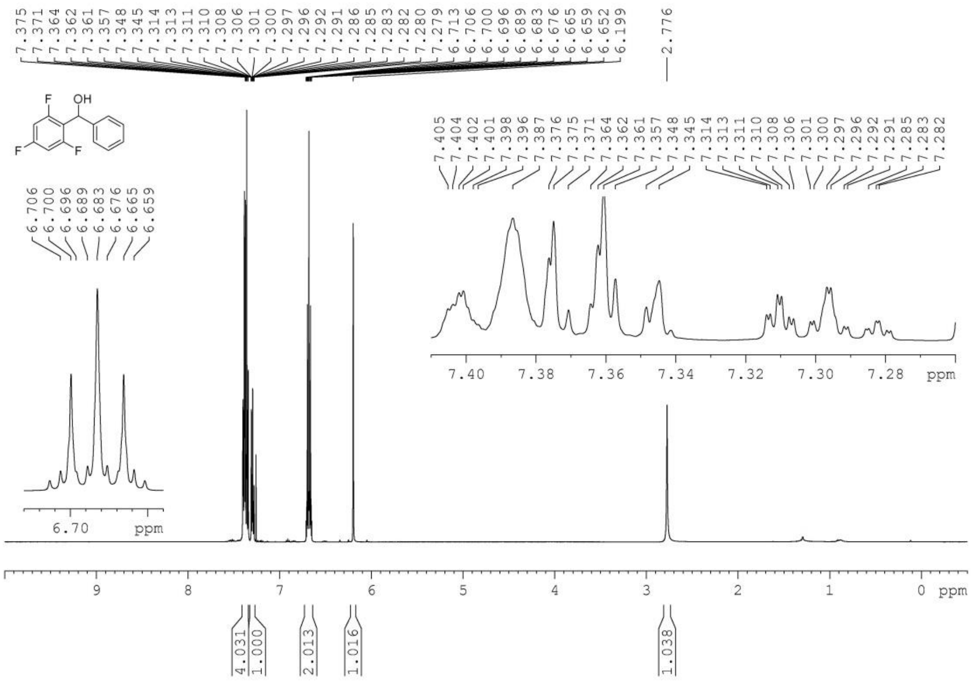

Compound 4c： $^{13}C\{^{1}H\}$ **NMR spectrum (125 MHz, $CDCl_3$).**

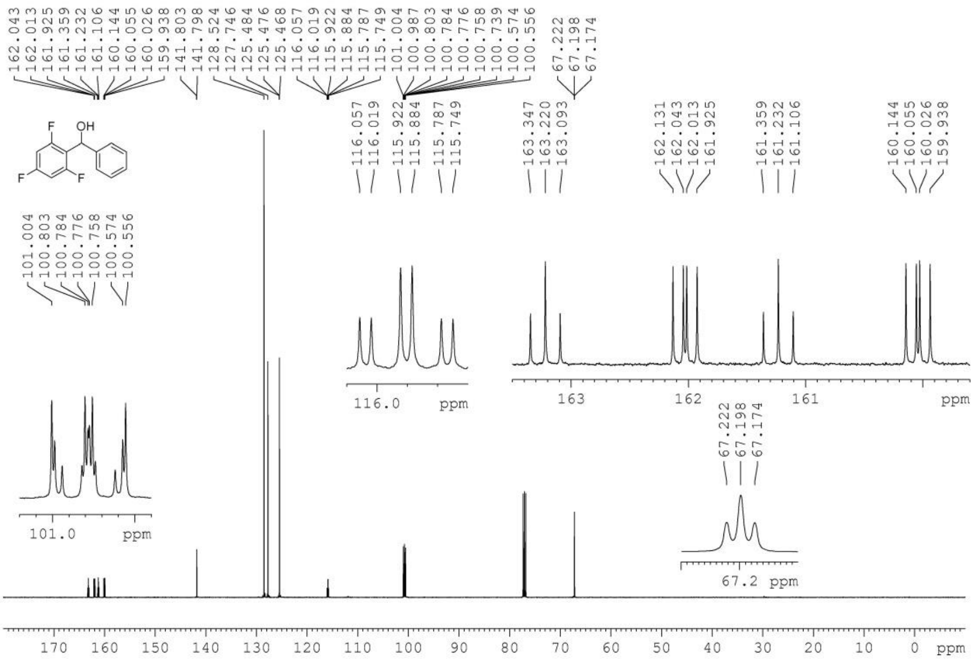

Compound 4c： ^{19}F **NMR spectrum (470 MHz, $CDCl_3$).**

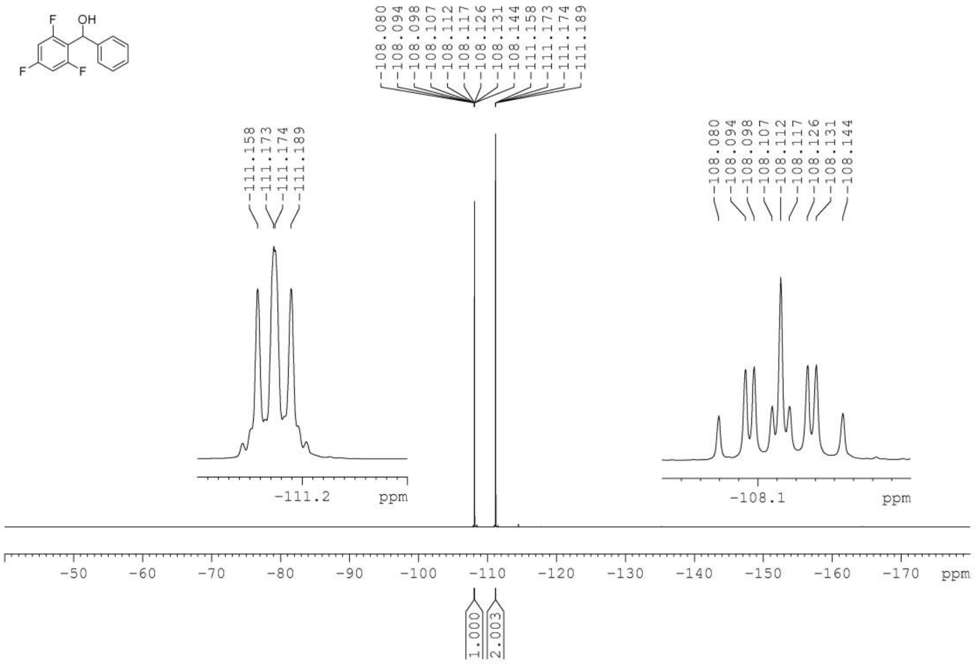

Compound 4d: [1] H NMR spectrum (500 MHz, $CDCl_3$).

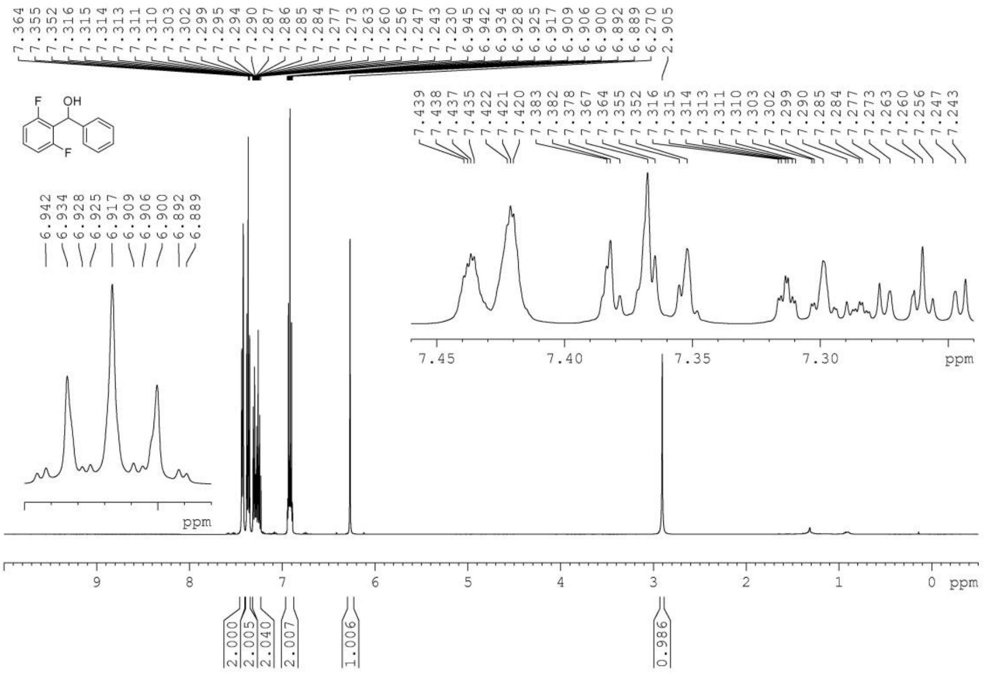

Compound 4d: [13] C{^{1}H} NMR spectrum (125 MHz, $CDCl_3$).

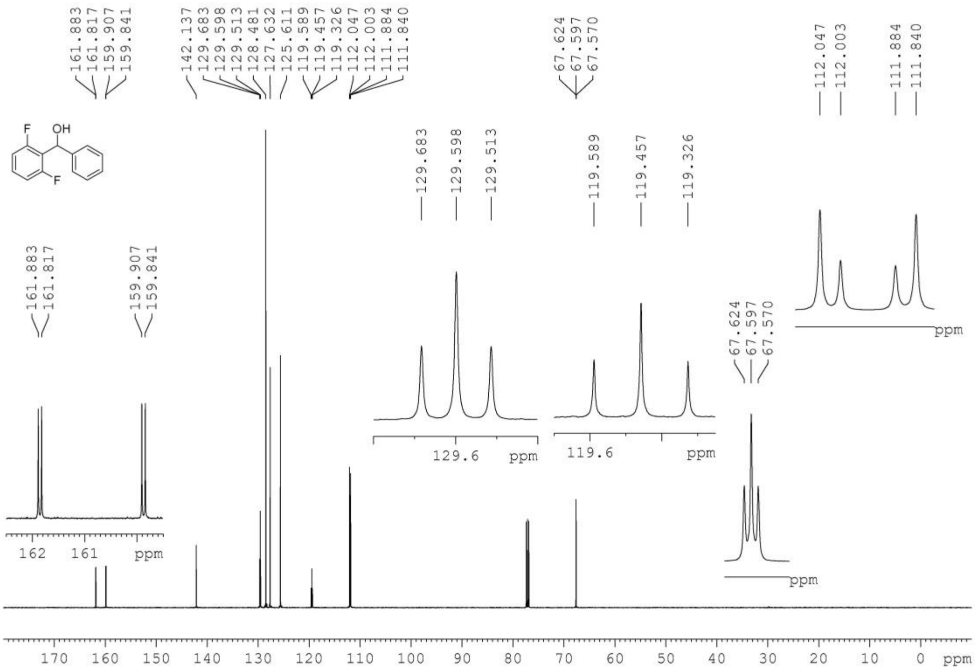

Compound 4d：[19] F NMR spectrum (470 MHz, $CDCl_3$).

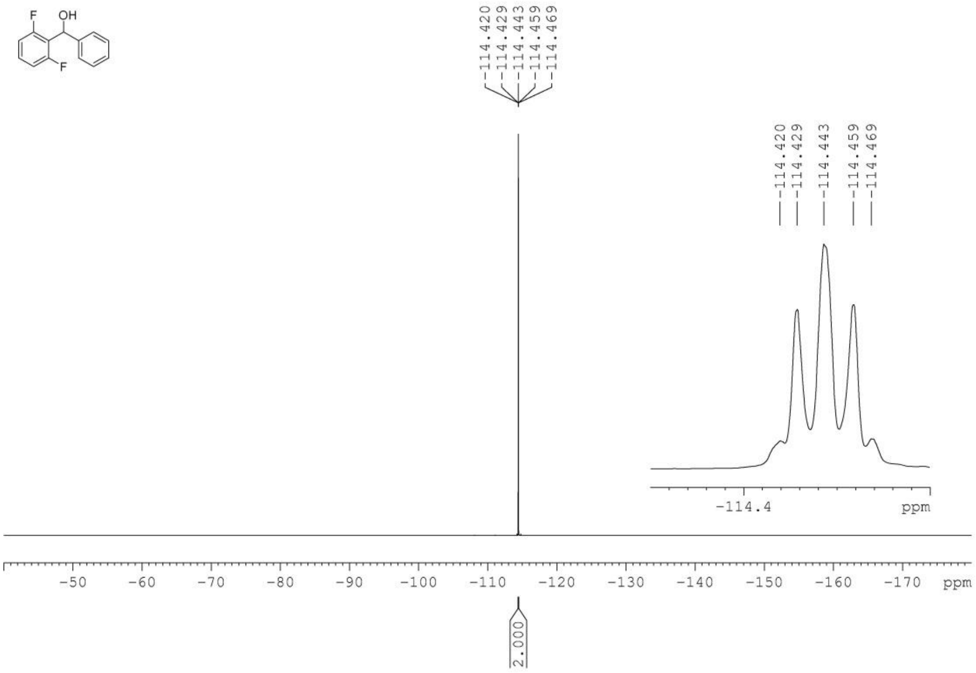

Compound 4e：[1] H NMR spectrum (500 MHz, $CDCl_3$).

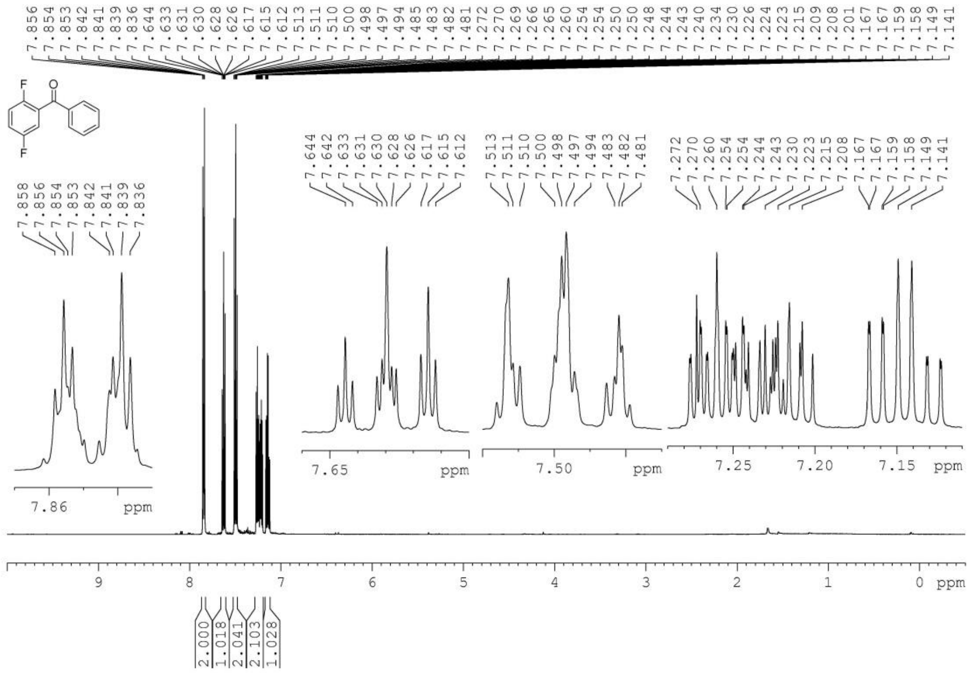

Compound 4e： ^{13} C{^{1}H} NMR spectrum (125 MHz, $CDCl_3$).

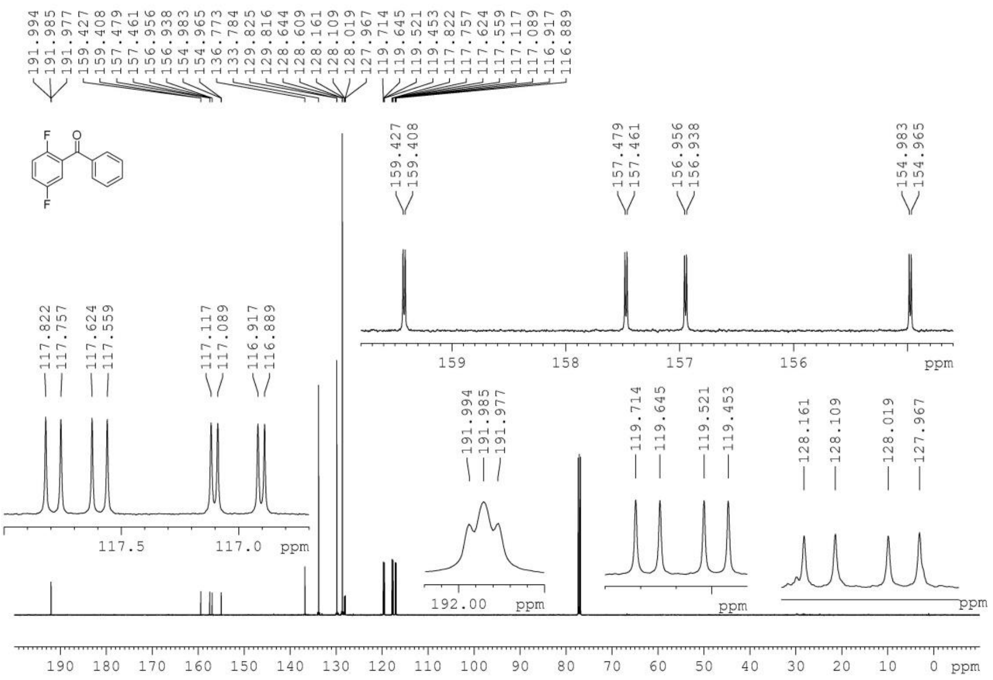

Compound 4e： ^{19} F NMR spectrum (470 MHz, $CDCl_3$).

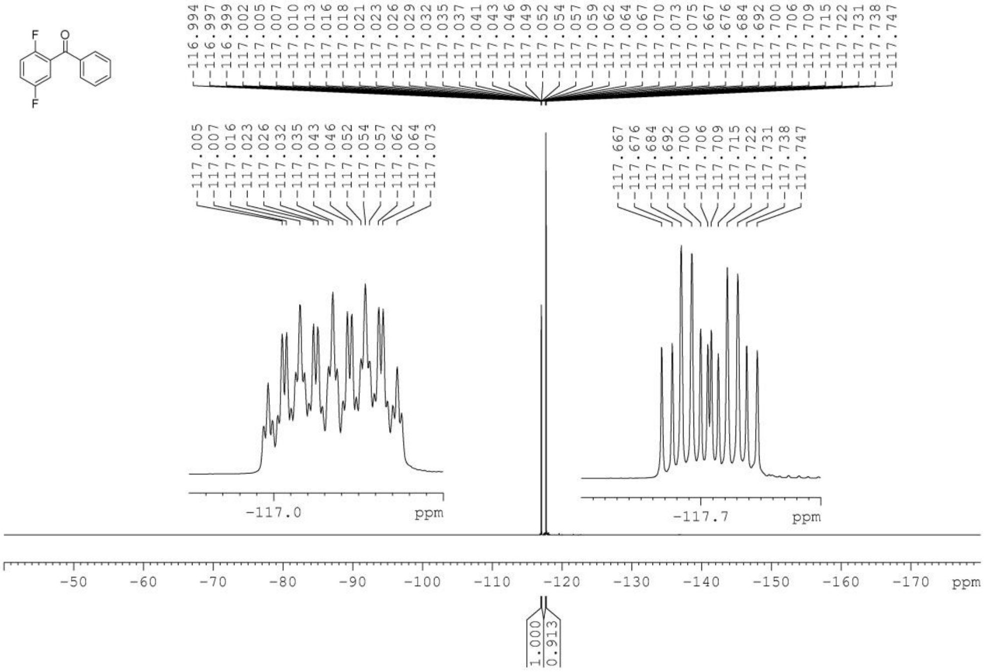

Compound 4f：[1] H NMR spectrum (500 MHz, $CDCl_3$).

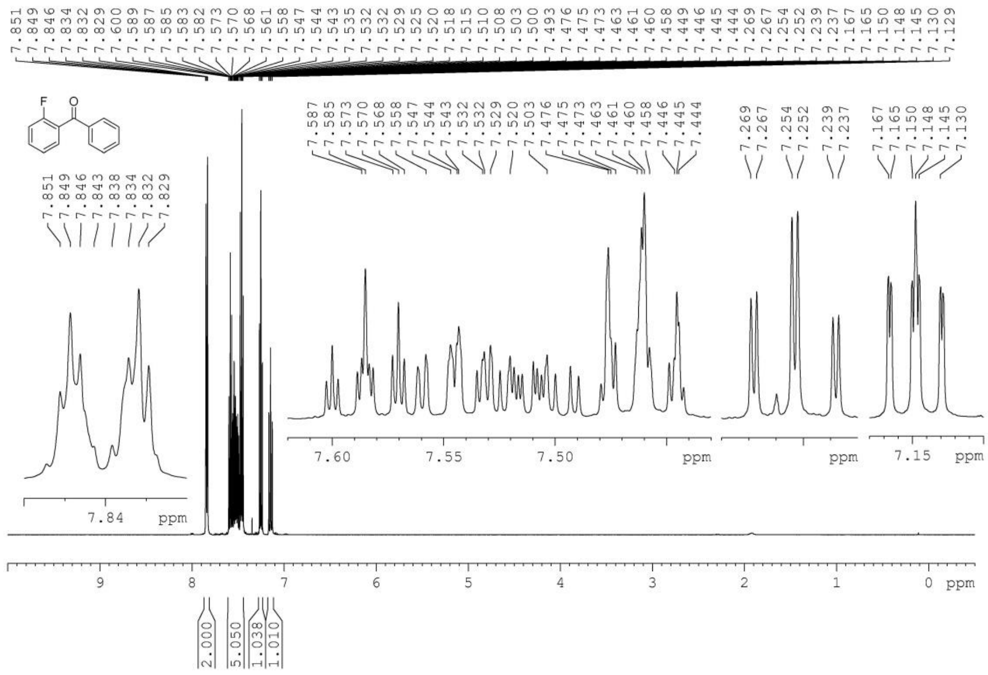

Compound 4f：[13] C{^{1}H} NMR spectrum (125 MHz, $CDCl_3$).

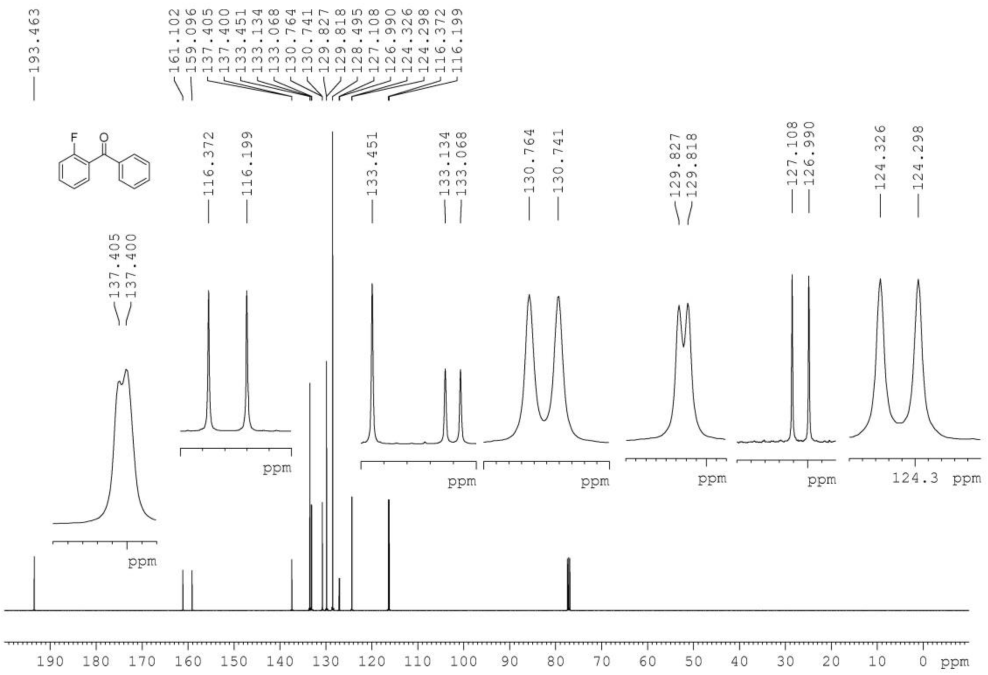

Compound 4f: [19] F NMR spectrum (470 MHz, $CDCl_3$).

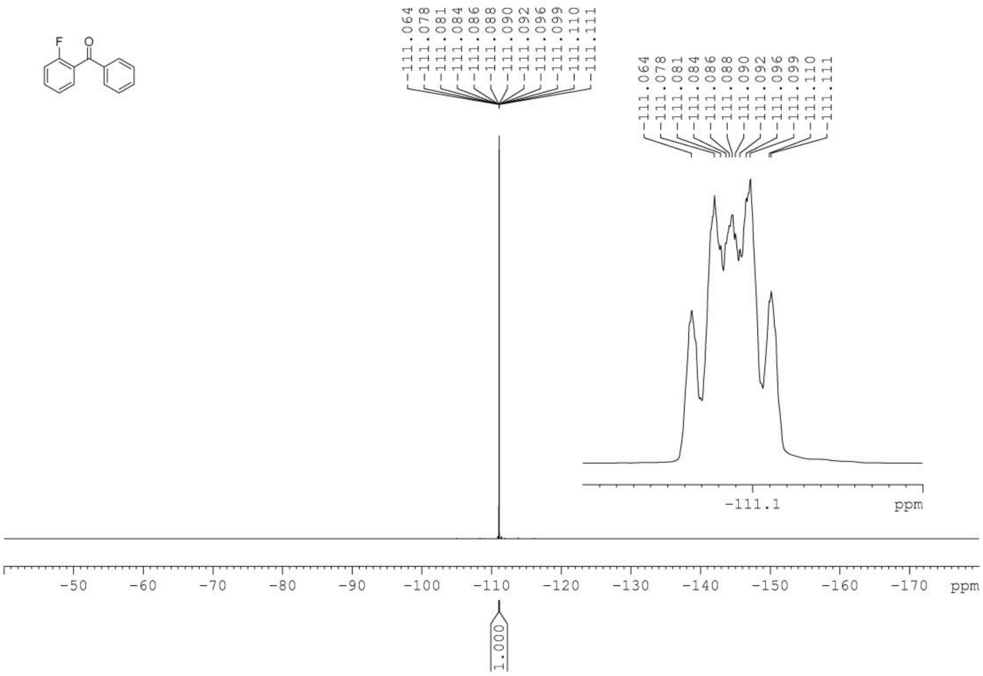

Compound 4g: [1] H NMR spectrum (500 MHz, $CDCl_3$).

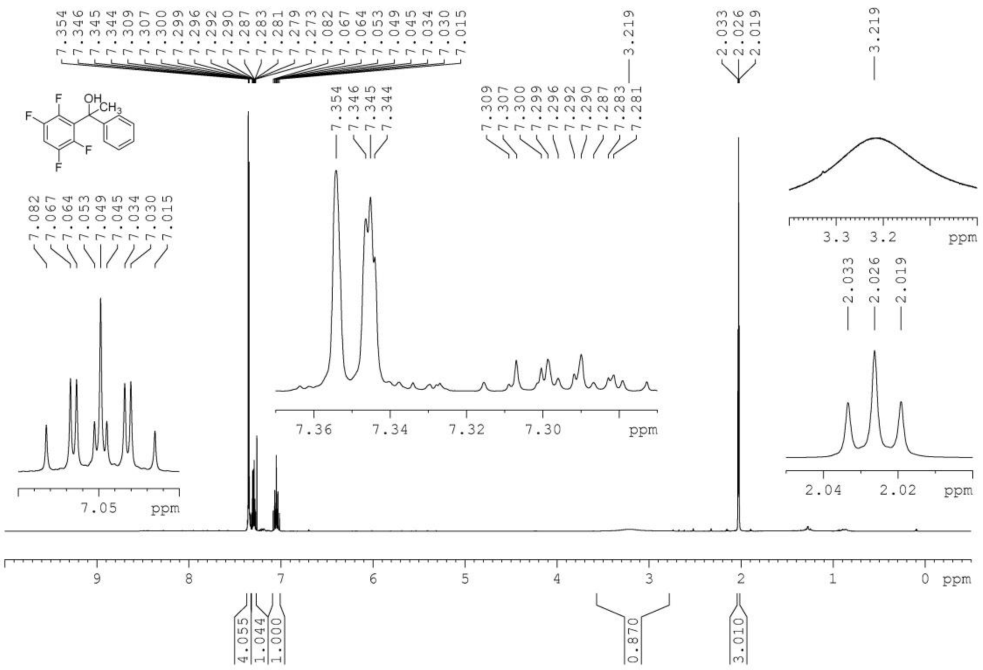

Compound 4g**:** ^{13} C{^{1}H} NMR spectrum (125 MHz, $CDCl_3$).

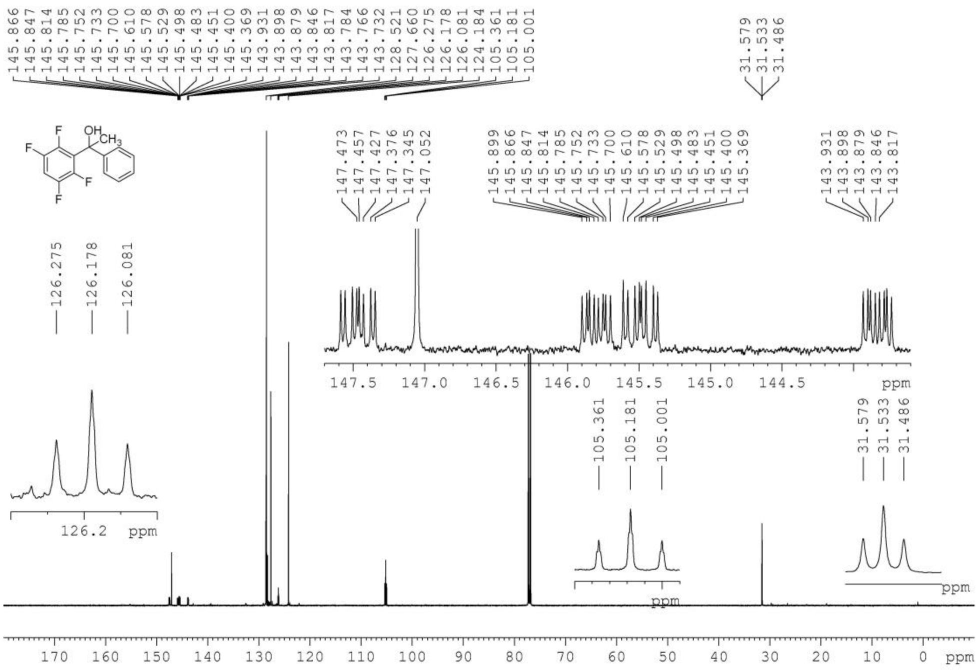

Compound 4g**:** ^{19} F NMR spectrum (470 MHz, $CDCl_3$).

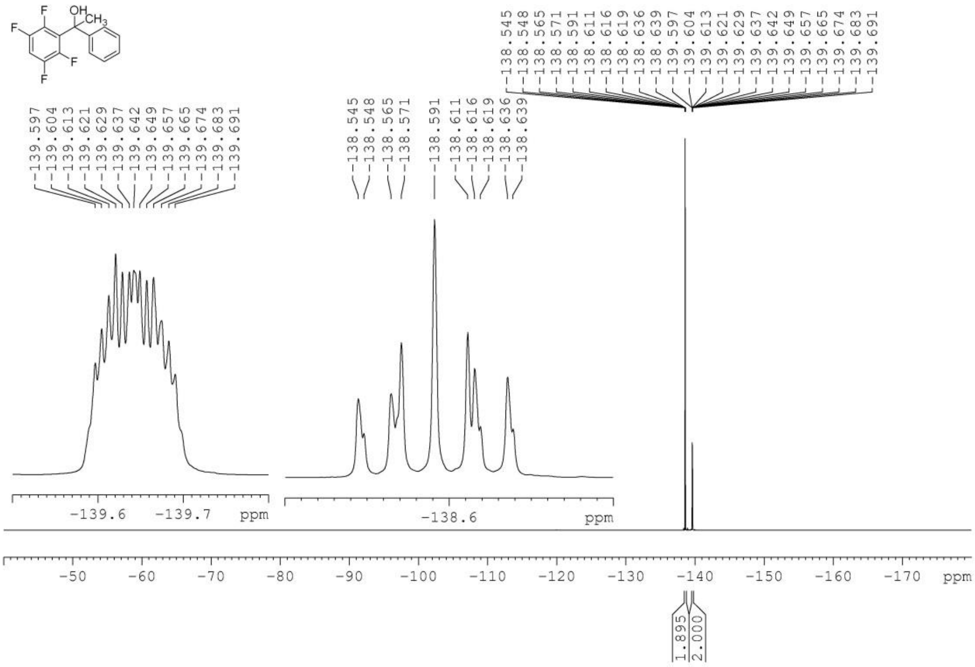

VI. Single Crystal X-Ray Diffraction

Single crystals suitable for X-ray diffraction were selected, coated in perfluoropolyether oil, and mounted on MiTeGen sample holders. Diffraction data were collected on Bruker X8 Apex II 4-circle diffractometers with CCD area detectors using Mo-Kα radiation monochromated by graphite or multi-layer focusing mirrors. The crystals were cooled using an Oxford Cryostream low-temperature device. Diffraction data were collected at 100 K. The images were processed and corrected for Lorentz-polarization effects and absorption effects by employing the Bruker software packages. The structure was solved using the intrinsic phasing method (SHELXT)[7d] and expanded using Fourier techniques. All the non-hydrogen atoms were refined in anisotropic approximation, with hydrogen atoms 'riding' in idealized positions, by full-matrix least squares against F^2 on all data, using SHELXL[7a,c] software and the SHELXLE[7b] graphical user interface. Crystal data and experimental details are listed in Table S1; full structural information has been deposited with the Cambridge Crystallographic Data Centre. CCDC-2045652 (**3f**), 2045653 (**3l**), 2045654 (**3m**), 2045655 (**3n**), and 2045656 (**4d**).

Table S1. Single-crystal X-ray diffraction data and structure refinements of 3f, 3l, 3m 3n, and 4d.

Compounds	**3f**	**3l**	**3m**	**3n**	**4d**
CCDC number	2045652	2045653	2045654	2045655	2045656
Empirical formula	$C_{13}H_6BrF_5O$	$C_{14}H_7F_5O_2$	$C_{17}H_9F_5O$	$C_{12}H_6F_5NO$	$C_{13}H_{10}F_2O$
Formula weight (g mol^{-1})	353.09	302.20	324.24	275.18	220.21
Temperature (K)	100(2)	100(2)	100(2)	100(2)	100(2)
Radiation, λ (Å)	Mo-Kα 0.71073	Mo-Kα 0.71073	Mo-Kα 0.71073	Mo-Kα 0.71073	Mo-Kα 0.71073
Crystal color, habit	Colorless, block	Colorless, block	Colorless, block	Colorless, block	Colorless, block
Crystal size (mm^3)	0.17×0.26×0.34	0.57×0.55×0.28	0.30×0.33×0.41	0.54×0.39×0.23	0.44×0.35×0.22
Crystal system	Triclinic	Triclinic	Monoclinic	Monoclinic	Monoclinic
Space group	$P1$	$P\bar{1}$	$P2_1/c$	$C2/c$	$C2/c$
Unit cell dimensions					
a (Å)	14.964(6)	6.9938(7)	8.422(5)	22.136(7)	20.058(3)
b (Å)	19.077(8)	7.7520(8)	23.486(13)	10.023(3)	10.796(3)
c (Å)	19.488(8)	11.9114(12)	14.108(9)	9.945(3)	9.2708(17)
α (°)	90.142(12)	71.116(2)	90	90	90
β (°)	109.530(7)	87.313(2)	99.583(17)	105.237(8)	98.046(8)
γ (°)	106.160(7)	82.190(2)	90	90	90
Volume (Å^3)	5008(4)	605.36(11)	2751(3)	2128.8(12)	1987.9(7)
Z	16	2	8	8	8
Calc. density (Mg·m^{-3})	1.873	1.658	1.565	1.717	1.472
μ (mm^{-1})	3.337	0.161	0.143	0.170	0.117
$F(000)$	2752	304	1312	1104	912
θ range (°)	1.493 - 26.370	2.800 - 27.103	1.701 - 26.369	1.907 - 26.371	2.051 - 26.372
Reflections collected	165215	25240	62274	51503	15767
Independent reflections	40933	2658	5627	2178	2035
Minimum/maximum transmission	0.486 / 0.533	0.679 / 0.717	0.621 / 0.702	0.711 / 0.746	0.691 / 0.746
Parameters / restraints	2963 / 208	191 / 0	618 / 615	173 / 0	148 / 0
Goof on F^2	1.003	1.070	1.089	1.072	1.037
R_1 [I>2$\sigma(I)$]	0.0351	0.0388	0.0945	0.0300	0.0397
wR^2 (all data)	0.0755	0.1099	0.2297	0.0842	0.1057
Maximum/minimum residual electron density (e·Å^{-3})	1.476 / –0.515	0.475 / –0.204	0.757 / -0.594	0.282 / –0.194	0.484 / –0.217

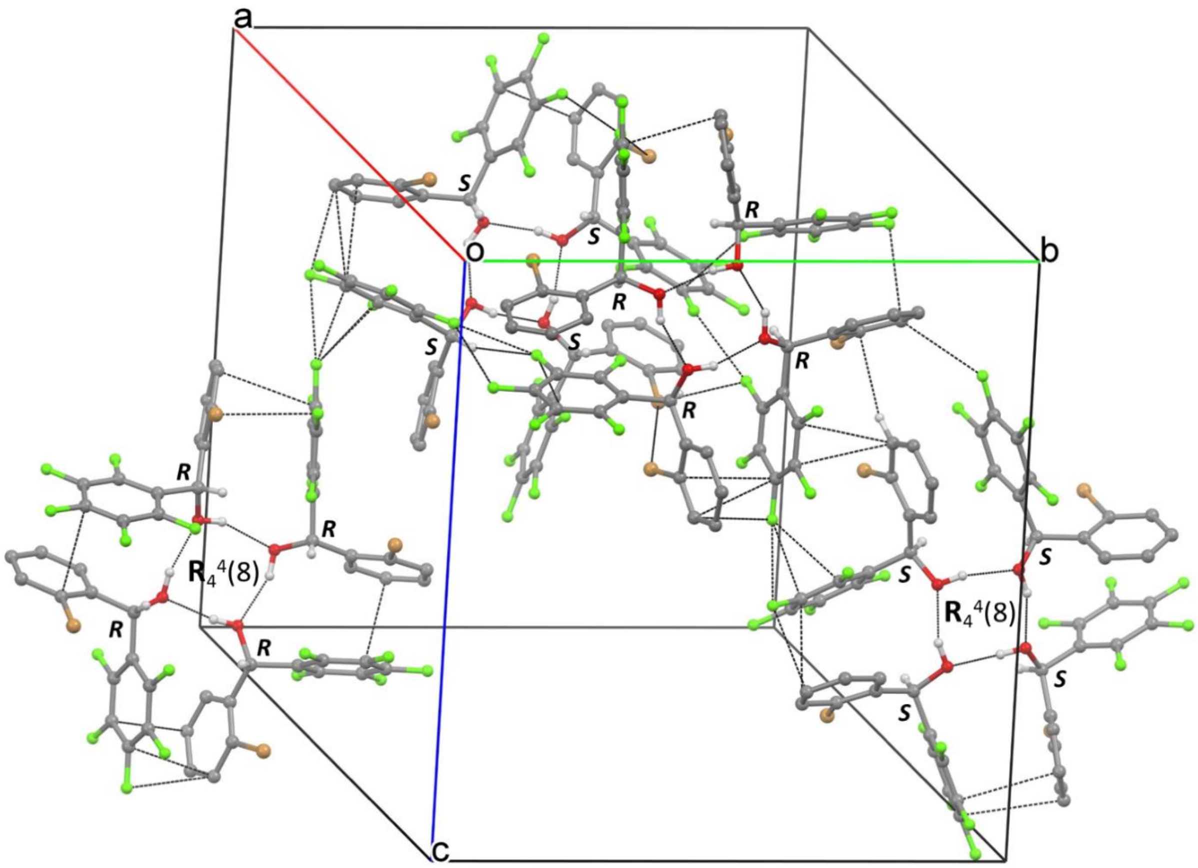

Figure S1. The asymmetric unit of **3f** and the unit cell metric are drawn. It consists of a racemic mixture of 16 symmetry-independent molecules which form four hydrogen-bonded tetramers of $\mathbf{R}_4^4(8)$ graphset. The chirality of all 16 molecules are shown. Each tetramer contains only one type of chirality. In addition to hydrogen bonding interactions, other interactions, such as π···π, C–F···π(C), C–H···π, F···F, F···Br, etc. are also observed. The interplanar separations and angles between the phenyl and pentafluorophenyl rings lie in the range of 3.281(7) – 3.687(14) Å and 4.96(19) – 16.8(3)°, respectively.

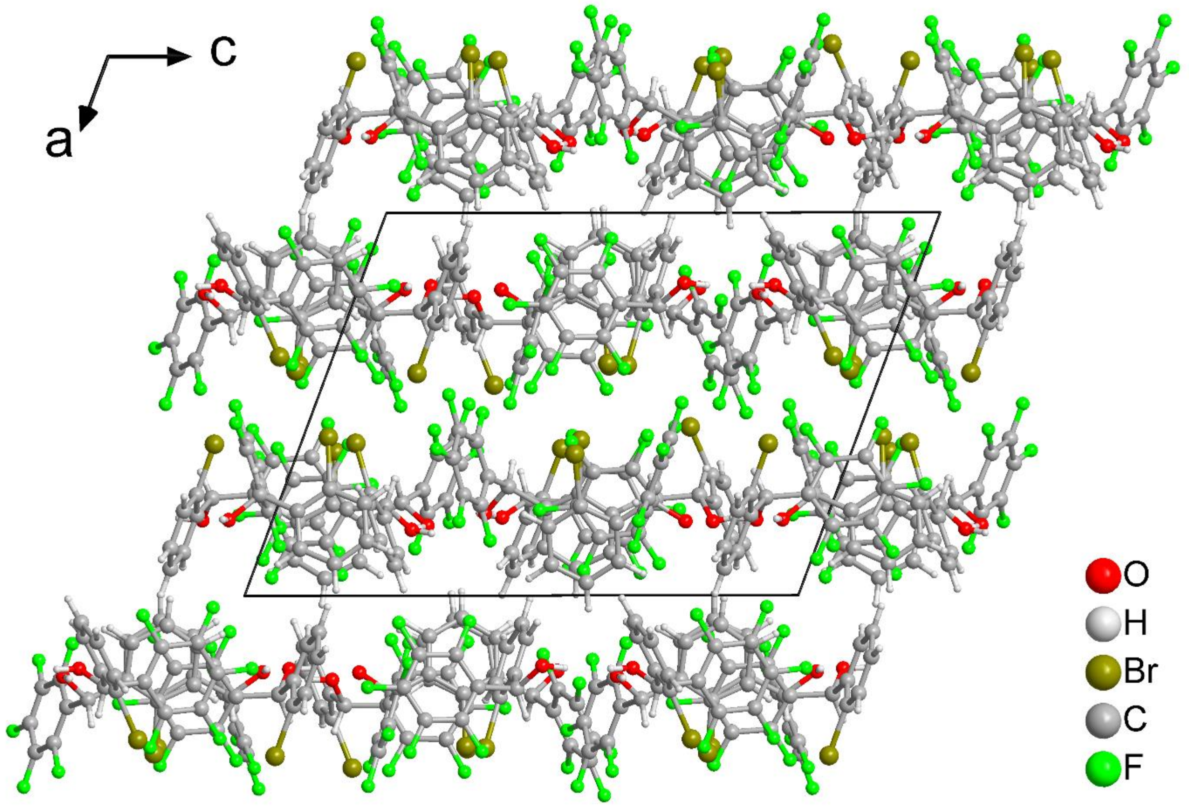

Figure S2. Tetramers of **3f** are arranged in sheets parallel to the $\vec{b},\vec{c}$-plane with bromine atoms all pointing up or down within a sheet. Parallel sheets face each other either with the bromine atoms or without.

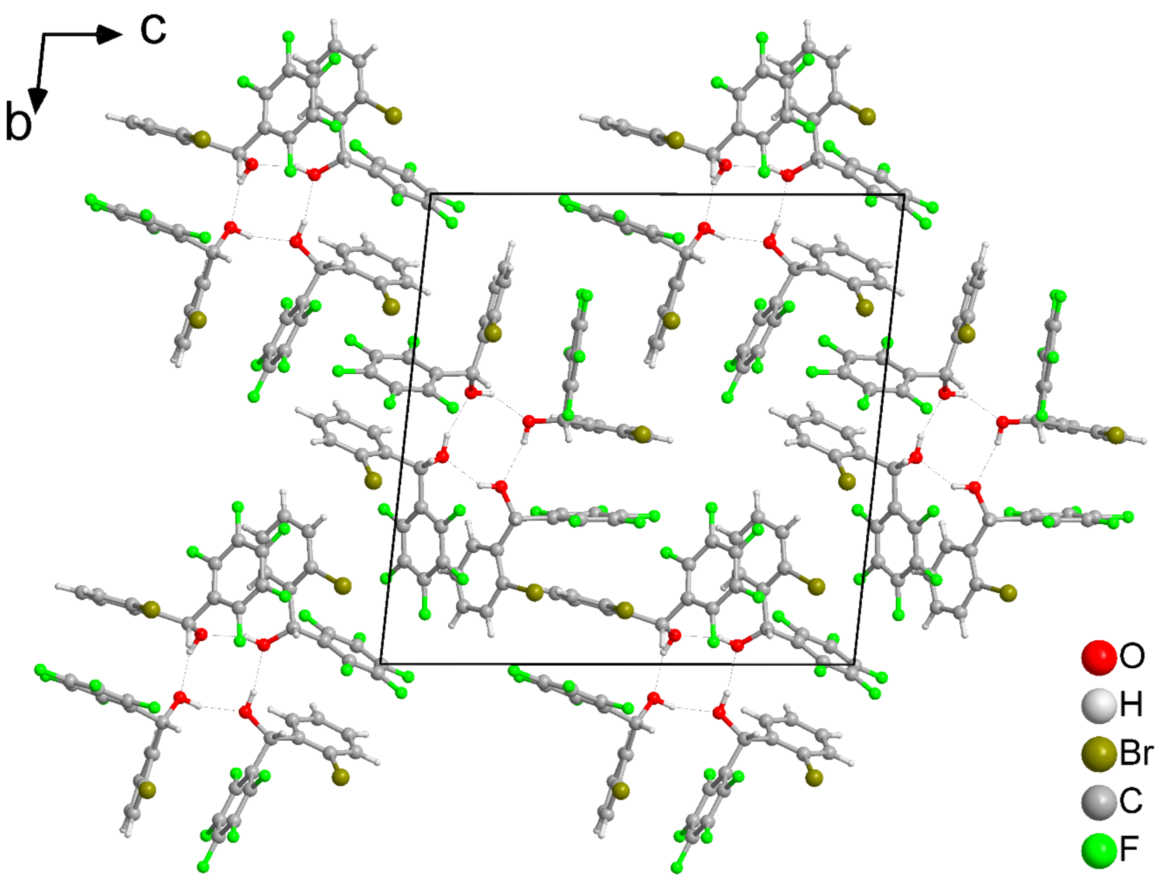

Figure S3. One of two symmetry-independent sheets of tetramers of **3f** containing two of four symmetry-independent tetramers. Tetramers within the sheet show alternating chirality.

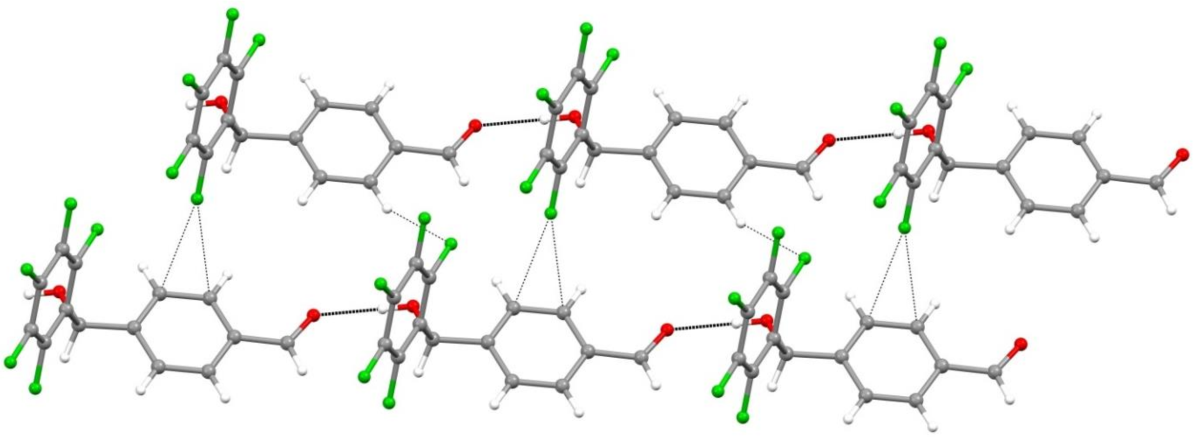

Figure S4. One-dimensional hydrogen bonded (O–H···O) chains also exhibit C–F···π(C) and C–H···F interactions between phenyl and pentafluorophenyl groups in **3l**.

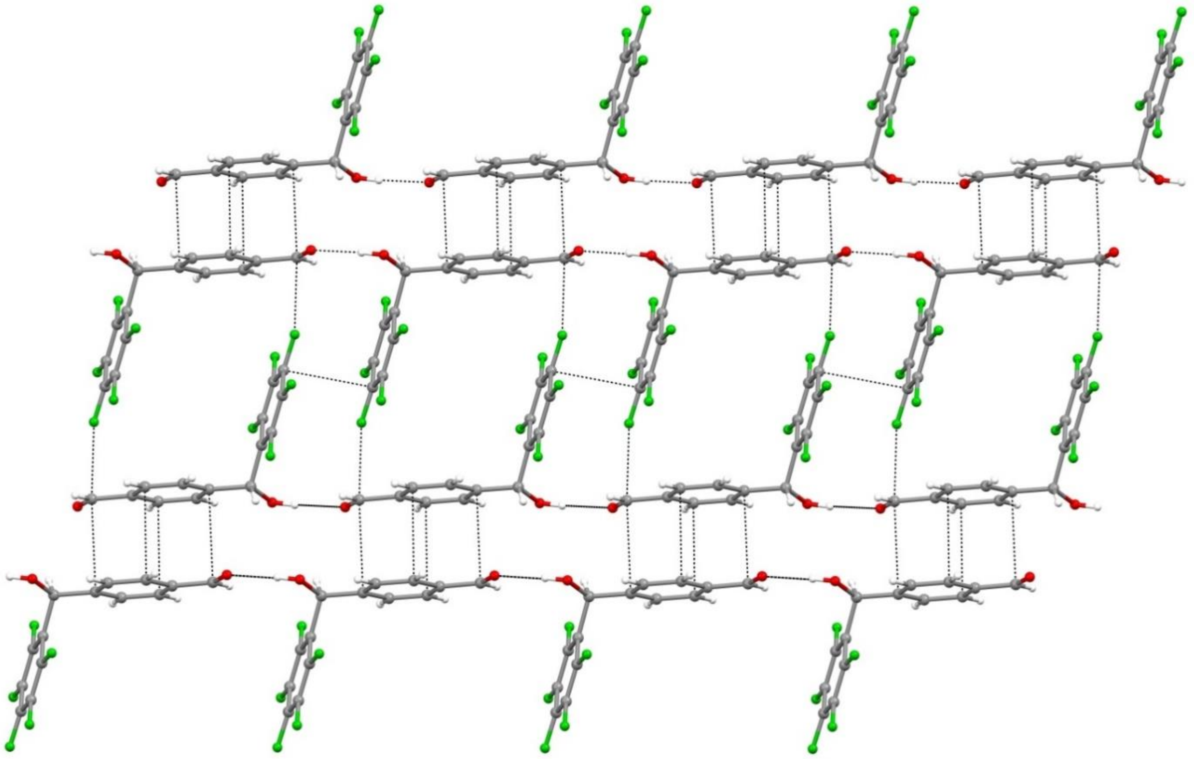

Figure S5. The π-stacked hydrogen-bonded chains further interdigitate[8] (viewed approximately along *a*-axis) and there exist partial π···π interactions between fluorinated moieties and C–F···π interactions between pentafluorophenyl and carbonyl groups in **3l**.

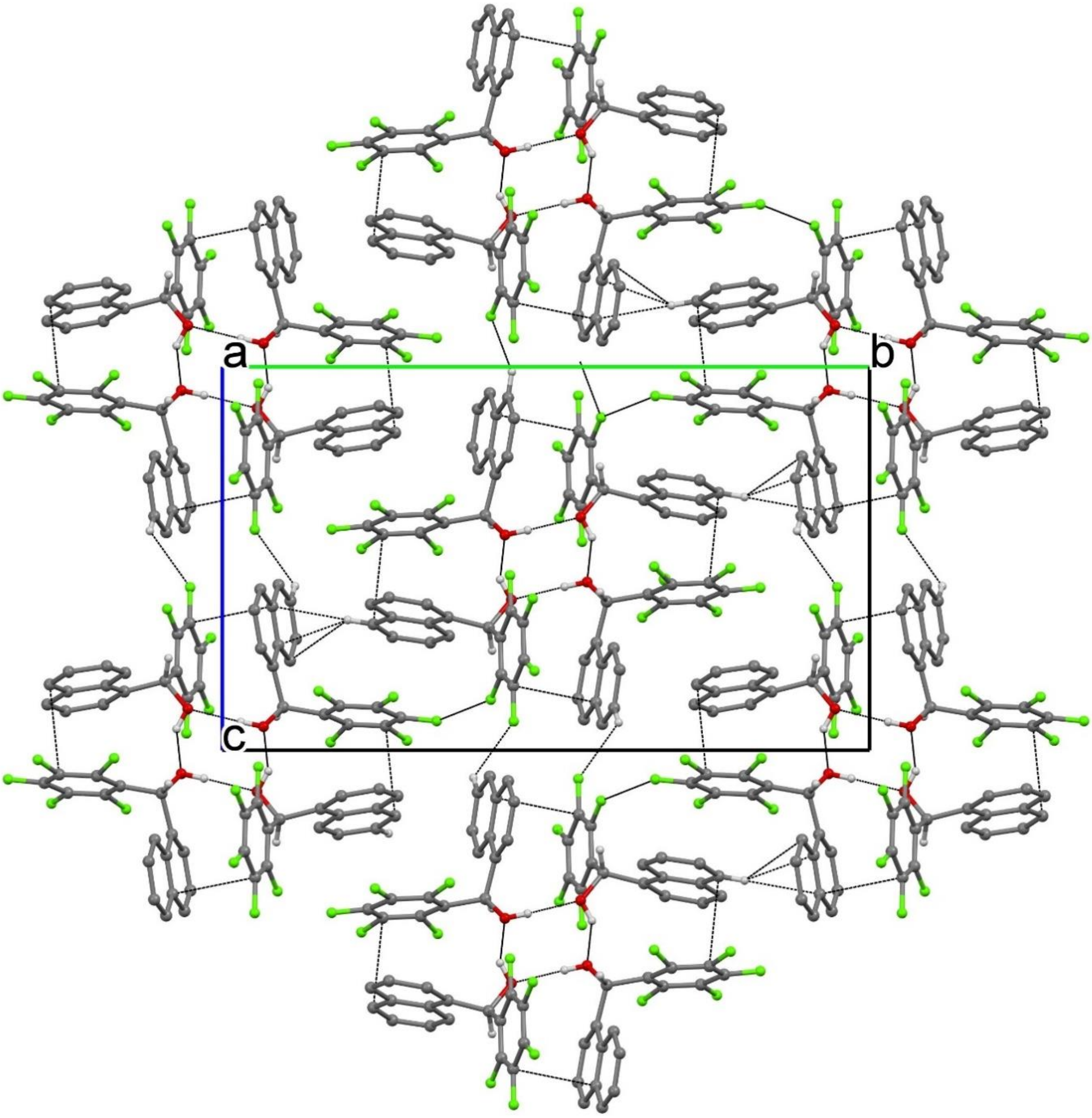

Figure S6. The crystal packing of compound **3m** is viewed along the *a*-axis. In addition to O–H···O hydrogen bonding, various intermolecular interactions, such as π···π, C–H···π, C–H···F, and F···F, are also observed. Interplanar separations and angles between the planes of naphthalene and pentafluorophenyl rings are 3.416(5) – 3.637(3) Å and 6.47(13) – 16.05(13)°.

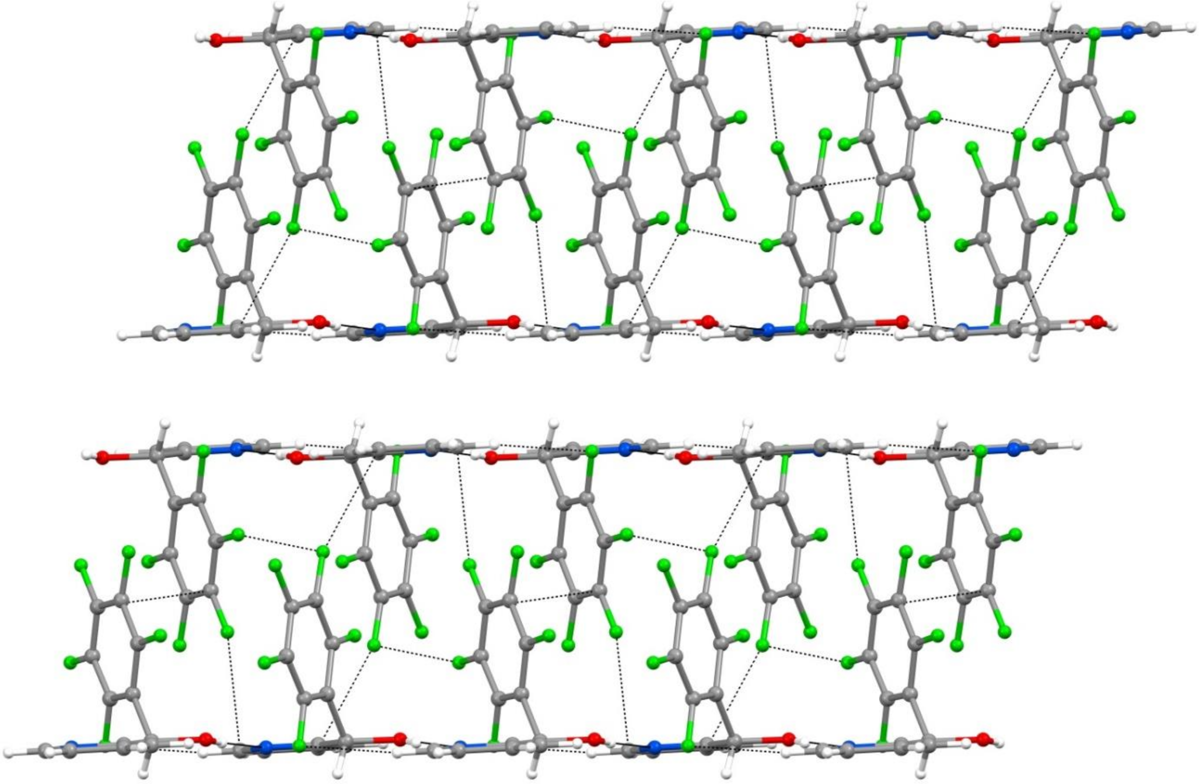

Figure S7. The crystal packing of **3n** is viewed along the *b*-axis. The pyridyl rings lie coplanar and the pentafluorophenyl groups interdigitate to form a parallel ribbon-like arrangement. Various types of weak interactions include, besides O–H···N hydrogen bonding, C–H···π, C–F···π, and F···F interactions ($d_{F\text{-}F}$ = 2.72 Å, $F_{vdW\text{-}radius}$ = 1.47 Å). Alternating hydrophobic and hydrophilic regions can be seen.

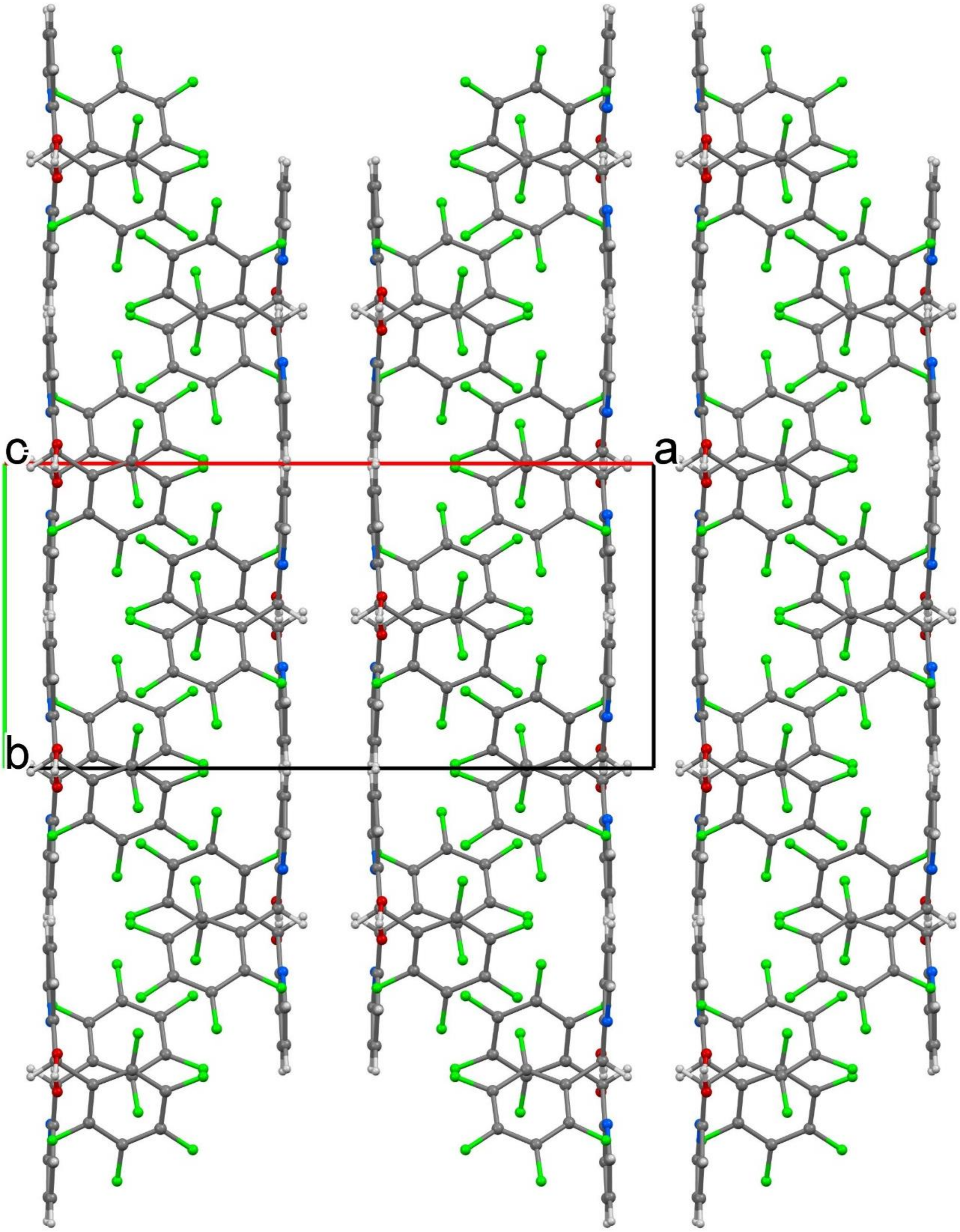

Figure S8. The crystal packing of **3n** is viewed along the *c*-axis. Alternating hydrophobic and hydrophilic regions can be seen.

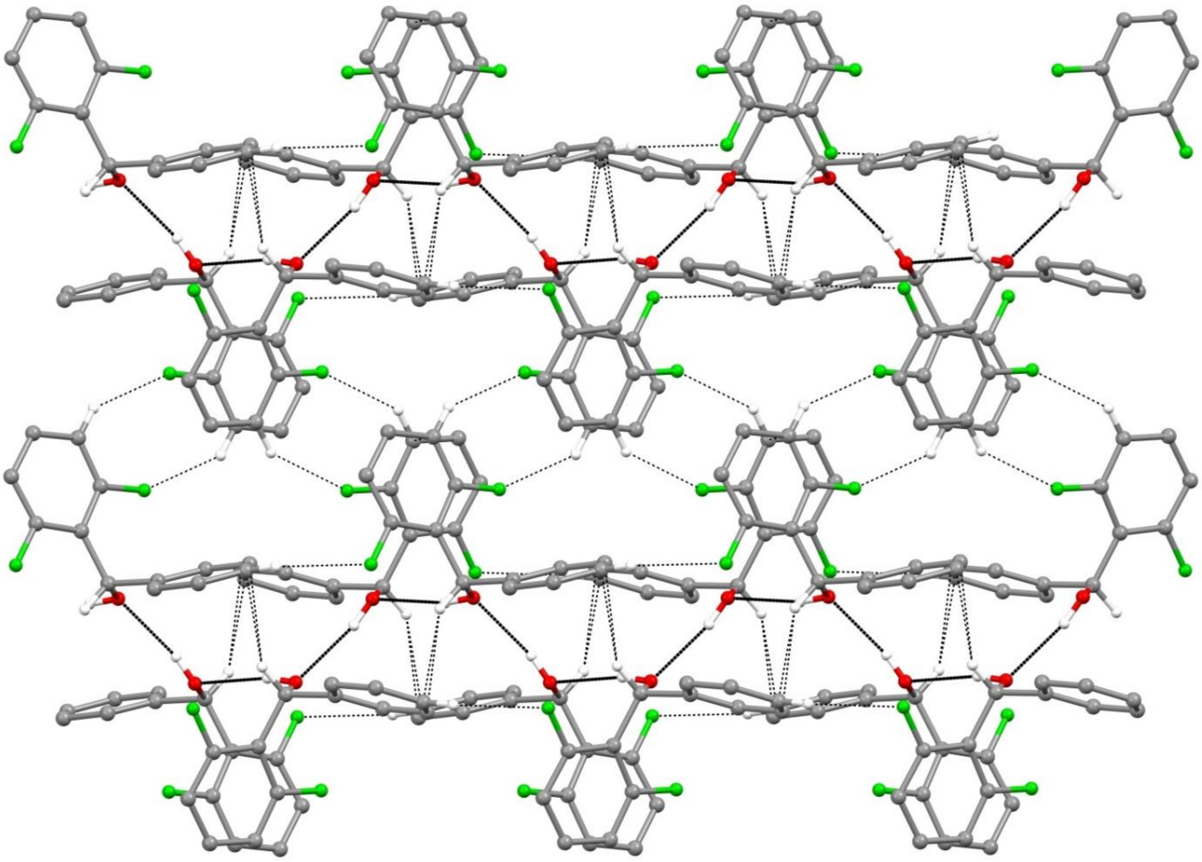

Figure S9. Crystal packing of **4d**, viewed along the *a*-axis, shows corrugated one-dimensional hydrogen bonded (O–H···O) chains propagating along the *c*-axis. In addition, several other intermolecular interactions, including C–H···F, C–H···π, and π···π, can be observed.

Table S2. Parameters for hydrogen bonding in 3f, 3l, 3m 3n and 4d.

**Donor(D)–H⋯Acceptor(A)** [a]	**H⋯A (Å)** [a]	**D⋯A (Å)**	**D–H⋯A (°)**
Compound **3f**			
O1–H1A⋯O2	1.90	2.693(5)	157.4
O2–H2A⋯O3	1.87	2.702(6)	171.0
O3–H3A⋯O4	1.90	2.704(5)	160.2
O4–H4A⋯O1	1.97	2.765(6)	156.5
O5–H5A⋯O8	1.91	2.697(6)	156.2
O8–H8A⋯O7	1.86	2.681(6)	166.6
O7–H7A⋯O6_1/11	2.02/2.07	2.703(14)/2.646(16)	138.4/125.4
O6_1/11–H6_1/11⋯O5	1.87/1.84	2.700(19)/2.666(11)	171.0/166.8
O9–H9A⋯O12	1.95	2.698(6)	148.1
O10–H10A⋯O9	1.97	2.756(5)	155.7
O11–H11A⋯O10	1.95	2.711(6)	150.3
O12–H12A⋯O11	1.92	2.740(5)	166.1
O13–H13A⋯O14	1.88	2.690(5)	162.0
O14–H14A⋯O15	1.90	2.716(5)	163.0
O15–H15A⋯O16	1.89	2.699(6)	161.0
O16–H16A⋯O13	1.88	2.677(6)	158.8
Compound **3l**			
O1–H1⋯O2	1.90	2.7412(16)	176.4
Compound **3m**			
O1_1–H1a_1⋯O1a_3	1.99	2.65(2)	135.3
O1_1–H1a_1⋯O1_3	2.11	2.72(2)	129.1
O1a_3–H1ab_3⋯O1_1	1.91	2.65(2)	145.6
O1a_3–H1ab_3⋯O1a_1	1.82	2.41(3)	125.4
Compound **3n**			
O1–H1⋯N1	1.95	2.7880(17)	173.5
Compound **4d**			
O1–H1A⋯O1	2.16	2.9815(17)	165.4
O1–H1B⋯O1	2.04	2.8661(16)	167.3

[a] The donor–H distance was constrained to be 0.84 Å for all H atoms. H atoms were refined riding in idealized positions.

Table S3. Aryl···aryl (π···π) distances (Å) and angles (°) in 3f, 3l, and 3m at 100 K.

Aryl···aryl	Centroid-centroid distance	Interplanar separation	Offset shift [a]	Plane normal angle
Compound **3f**				
Ar_{Br1}···Ar_F	3.648(4)	3.529(5)/3.330(7)	0.924(11)/1.490(12)	9.6(3)
Ar_{Br2}···Ar_F	3.536(4)	3.470(4)/3.284(5)	0.682(9) / 1.311(8)	11.04(19)
Ar_{Br3}···Ar_F	3.800(4)	3.570(5)/3.348(6)	1.301(11)/1.797(9)	9.9(2)
Ar_{Br4}···Ar_F	3.648(4)	3.525(5)/3.386(6)	0.940(11)/1.357(10)	9.5(2)
Ar_{Br5}···Ar_F	3.657(3)	3.538(4)/3.362(5)	0.927(9)/1.439(9)	8.75(19)
Ar_{Br6_1}···Ar_F	3.694(14)	3.640(14)/3.687(14)	0.631(14)/0.23(5)	10.7(8)
Ar_{Br6_11}···Ar_F	3.719(15)	3.717(14)/3.682(14)	0.12(5)/0.518(15)	9.9(8)
Ar_{Br7}···Ar_F	3.783(5)	3.285(8)/3.659(6)	1.875(12)/0.959(13)	16.8(3)
Ar_{Br8}···Ar_F	3.523(4)	3.416(4)/3.514(4)	0.729(10)/0.25(1)	8.0(2)
Ar_{Br9}···Ar_F	3.674(5)	3.494(6)/3.276(8)	1.138(12)/1.663(12)	8.9(3)
Ar_{Br10}···Ar_F	3.719(4)	3.528(5)/3.317(5)	1.179(10)/1.682(9)	11.43(19)
Ar_{Br11}···Ar_F	3.728(4)	3.554(5)/3.362(5)	1.128(10)/1.611(9)	8.8(2)
Ar_{Br12}···Ar_F	3.564(4)	3.484(4)/3.343(6)	0.754(11)/1.237(10)	9.7(2)
Ar_{Br13}···Ar_F	3.739(4)	3.357(6)/3.635(4)	1.646(10)/0.877(10)	13.3(2)
Ar_{Br14}···Ar_F	3.610(4)	3.593(4)/3.523(4)	0.35(1)/0.788(10)	12.5(2)
Ar_{Br15}···Ar_F	3.706(5)	3.559(5)/3.281(7)	1.034(12)/1.723(11)	12.9(3)
Ar_{Br16}···Ar_F	3.592(4)	3.577(4)/3.539(4)	0.330(9)/0.614(9)	4.96(19)
Compound **3l**				
Ar···Ar	3.6667(10)	3.3379(13)	1.5176(19)	0.00(7)
Ar_F ···Ar_F	4.6778(14)	3.375(2)	3.238(3)	0.00(1)
Compound **3m** [b]				
Ar_d···Ar_F	3.650(3)	3.637(3)/3.564(3)	0.305(7)/0.787(6)	16.05(13)
Ar···Ar_{F_d}	3.538(3)	3.448(3)/3.357(4)	0.794(5)/1.119(7)	6.47(13)
Ar_d···Ar_F (T···T)	3.886(3)	3.613(4)/3.416(5)	1.431(5)/1.854(7)	6.89(13)
Compound **3n**				
Ar_F ···Ar_F	4.2972(15)	3.193(2)	2.876(2)	0.0(5)
Compound **4d**				
Ar···Ar	4.8860(13)	3.177(3)	3.712(2)	0.00(1)
Ar_{HF} ···Ar_{HF}	4.5100(14)	3.358(2)	3.010(3)	17.65(7)

[a] The offset shift, also called inter-centroid shift, is the distance within a plane of an aryl ring between the centroid of the respective aryl ring and the intersection point with the normal to the plane through the centroid of the other aryl ring.

[b] Only interactions with the main parts (85%) of the disordered (d) pentafluorophenyl and naphthalene moieties are reported here.

VII. Computational Methods

All the calculations were performed with the Gaussian09 programs.[9] The geometries of the different structures were optimized at the DFT level using the B3-LYP[10-11] hybrid functional with 6-31+G(d) basis set. Frequency analysis was carried out at the same level to verify the stationary points as an intermediate or transition state and to obtain the thermodynamic energy corrections assuming a standard state of 1 atm and 298.15 K. Intrinsic reaction coordinates (IRC)[12] were calculated to confirm the connection between the transition state and the correct reactant/product. The solvent effects were taken into consideration by single point calculations of the gas-phase stationary points with the SMD[13-15] continuum salvation model. To obtain more accurate energy information, single-point calculations were carried out with the M06[16] functional and 6-311++G(d,p) basis set in toluene solvent (using the SMD solvent model). All of the three-dimensional molecular diagrams of the molecules were generated with CYLView.[17]

Table S4. Absolute calculated electronic energies, correction of enthalpies, and free energies.

Geometry	$E_{(elec\text{-}B3LYP)}$[1]	$G_{(corr\text{-}B3LYP)}$[2]	$H_{(corr\text{-}B3LYP)}$[3]	$E_{(M06\ toluene)}$[4]	IF[5]
**6**	-2602.91354	0.19189	0.276362	-2602.613003	
**7-ts**	-2602.862623	0.187452	0.27543	-2602.566437	-135.94
**8**	-2602.899113	0.183295	0.276536	-2602.586769	
**9**	-2948.493833	0.283666	0.395715	-2948.028436	
**10-ts**	-2948.48623	0.2872	0.395038	-2948.022579	-104.87
**11**	-2948.514631	0.289305	0.397303	-2948.048797	
**12-ts**	-2948.483194	0.291873	0.396411	-2948.029586	-71.99
**13**	-2948.526205	0.29542	0.397854	-2948.072049	
**14-ts**	-4087.664887	0.503267	0.650296	-4086.926819	-74.41
**15**	-4087.674391	0.503786	0.651069	-4086.934931	
**16-ts**	-4087.667933	0.502938	0.650055	-4086.930499	-106.81
**17**	-1484.742692	0.286987	0.373107	-1484.297391	
**18**	-2503.68739	0.201261	0.283462	-2503.387159	
**19-ts**	-2503.634465	0.196446	0.282443	-2503.338675	-135.57
**20**	-2404.458457	0.210645	0.290577	-2404.158042	
**21-ts**	-2404.40159	0.205886	0.289656	-2404.105012	-137.3
**22**	-2305.218418	0.220156	0.297974	-2304.918703	
**23-ts**	-2305.159478	0.215332	0.297033	-2304.863907	-141.31
**24**	-2205.970598	0.228529	0.305459	-2205.671805	
**25-ts**	-2205.908151	0.223326	0.304145	-2205.612351	-104.72
**26**	-2205.963365	0.228374	0.305711	-2205.664144	
**27-ts**	-2205.892339	0.222338	0.304343	-2205.595884	-114.48
**28**	-1402.96216	0.191335	0.268364	-1402.767999	
**29-ts**	-1402.951039	0.18894	0.266972	-1402.754229	-132.93
**30**	-675.200515	0.156661	0.211906	-675.04524	

**31**	-727.84762	0.011433	0.055189	-727.754167	
**32-ts**	-1073.433312	0.110004	0.173384	-1073.176777	-121.88
**33**	-1073.441942	0.113864	0.175008	-1073.188879	
**34-ts**	-1748.560884	0.292987	0.387263	-1748.200815	-108.2
**35**	-1748.578555	0.295372	0.389196	-1748.223167	

[1] The electronic energy calculated by B3-LYP/6-31+g(d) in gas phase. [2] The thermal correction to Gibbs free energy calculated by B3-LYP/6-31+g(d) in gas phase. [3] The thermal correction to enthalpy calculated by B3-LYP/6-31+g(d) in gas phase. [4] The electronic energy calculated by M06/6-311++G(d,p) in toluene. [5] The B3-LYP calculated imaginary frequencies for the transition states.

B3LYP geometries for all the optimized compounds and transition state.

6

C	-2.27197500	0.88043600	-1.53236400
C	-2.65585600	-0.62458300	-1.26977200
O	-1.50658800	-1.11901800	-0.57012800
O	-1.52181800	1.20667900	-0.35807100
B	-0.82930300	-0.00536400	0.14312500
C	0.81156600	-0.00311700	-0.20654500
C	1.57578400	1.16182700	-0.26982500
C	1.54782400	-1.16102800	-0.45624800
C	2.93231300	1.20292500	-0.58179700
C	2.90348000	-1.18470400	-0.77289900
C	3.60583200	0.01408700	-0.84209000
O	-1.03623900	-0.09693500	1.60633000
C	-0.02050700	-0.16995300	2.61978200
O	0.33486200	-1.32923400	2.92791100
O	0.33160600	0.93346200	3.09209100
C	-3.88967900	-0.76451400	-0.35566100
H	-4.81443900	-0.45330500	-0.85606700
H	-4.00876800	-1.81920100	-0.07493400
H	-3.76934400	-0.18045300	0.56199600
C	-2.86422200	-1.46237000	-2.53519300
H	-3.13209500	-2.49061300	-2.26257100
H	-3.67923300	-1.05863100	-3.14923800
H	-1.95640700	-1.50483400	-3.14221000
C	-3.46281000	1.83876300	-1.63913700
H	-3.10237700	2.85735100	-1.82798000
H	-4.12221100	1.56149300	-2.47087500
H	-4.05557800	1.85240900	-0.71940700
C	-1.37274000	1.05628800	-2.77092000
H	-1.92916100	0.90706000	-3.70377100

H	-0.96944700	2.07510200	-2.77334300
H	-0.52928100	0.35996600	-2.75615700
F	1.00950400	2.37975900	-0.00637500
F	3.59563500	2.37278600	-0.63053400
F	4.91280700	0.02264500	-1.14748600
F	3.53950500	-2.34771000	-1.00564700
F	0.95227900	-2.39118300	-0.38382100
K	-1.04721800	-2.87445200	1.41987500
K	-1.04337900	2.67075900	1.79668500

7-ts

C	0.46292300	2.80457500	-0.28914600
C	0.93411000	2.36303100	1.16428300
O	0.87683100	0.91600700	1.09949800
O	0.74813000	1.63109200	-1.08854900
B	0.85999100	0.54819300	-0.24076000
C	-1.31576800	-0.51892500	-0.33293200
C	-2.26133100	-0.08870200	-1.25197100
C	-1.86771100	-1.09607000	0.79104800
C	-3.64089400	-0.16870500	-1.05798600
C	-3.22770800	-1.21845600	1.06731000
C	-4.12723600	-0.73266400	0.12163900
O	1.51143300	-0.64889100	-0.66281000
C	2.83025300	-0.92611000	-0.45547200
O	3.66663800	-0.00183500	-0.32608100
O	3.09202800	-2.18298700	-0.43336200
C	2.39361100	2.73219200	1.47641400
H	2.52668100	3.81639300	1.57357700
H	2.67368000	2.26678300	2.42853100
H	3.07010100	2.35019600	0.70696100
C	0.03030000	2.83613500	2.30474800
H	0.41221200	2.44826600	3.25580400
H	0.01638800	3.93156600	2.36603700
H	-0.99545900	2.47979900	2.18548300
C	1.24801100	3.97521600	-0.88729600
H	0.87359700	4.18332900	-1.89564900
H	1.12174100	4.88282900	-0.28394900
H	2.31424600	3.74801700	-0.96396200
C	-1.04009200	3.10287600	-0.38850800
H	-1.29525600	4.03636500	0.12697000
H	-1.31041400	3.20420500	-1.44443900
H	-1.64393300	2.29651200	0.03300100
F	-1.86829200	0.45997600	-2.44177900
F	-4.52419900	0.27623100	-1.98186400
F	-5.45583100	-0.82835800	0.33350600
F	-3.69504800	-1.80380000	2.19687600

F	-1.01943300	-1.67657400	1.74561600
K	5.61649200	-1.62245000	-0.19876500
K	0.45353000	-2.92102000	-0.21376700

8

C	5.27663600	1.00011600	-0.46195400
C	5.40606700	-0.48068100	0.08284100
O	4.12362500	-0.67871900	0.74640200
O	3.83438000	1.14029100	-0.63301200
B	3.24118600	0.20198900	0.18236900
C	-3.10863500	1.35634300	-0.42346400
C	-4.37302000	1.75597400	-0.03626100
C	-3.02269700	0.02479900	-0.71918100
C	-5.47480000	0.90413400	0.06419200
C	-4.04871500	-0.90520200	-0.61153400
C	-5.30537500	-0.45243300	-0.22182000
O	1.89177800	0.27594500	0.43221300
C	1.06562400	-0.83599600	0.76323300
O	1.37994000	-1.95077400	0.31481000
O	0.03991600	-0.49648300	1.40842900
C	5.49761700	-1.53551300	-1.02858300
H	6.46620600	-1.49763500	-1.53953600
H	5.37924300	-2.52811800	-0.58281300
H	4.70507100	-1.40857800	-1.77337500
C	6.51783300	-0.69971100	1.10688100
H	6.50898800	-1.74351300	1.43793600
H	7.50013300	-0.49181600	0.66590700
H	6.38874600	-0.06699400	1.98856800
C	5.95120700	1.25762500	-1.80756200
H	5.77862700	2.29543300	-2.11263500
H	7.03429900	1.10170600	-1.73613400
H	5.55559600	0.60653200	-2.59064200
C	5.70937700	2.06356700	0.55700400
H	6.79481400	2.06027400	0.70533400
H	5.41738500	3.05163200	0.18562200
H	5.22789800	1.91042000	1.52872800
F	-4.60299700	3.06369700	0.31317600
F	-6.69539600	1.34037300	0.44678400
F	-6.33777700	-1.31272000	-0.11097600
F	-3.84415100	-2.24808800	-0.83563000
F	-1.78009700	-0.51937500	-1.13495500
K	-1.19472100	-2.64939000	0.40126200
K	-0.40122600	1.89915500	0.28674400

9

C	-4.83159700	0.03947600	0.36536300
C	-3.49213300	0.24315000	0.68414400

C	-2.43332400	-0.53403600	0.27153600
C	-2.82305300	-1.58400600	-0.52603000
C	-4.12916000	-1.88013400	-0.90604300
C	-5.15007300	-1.04698000	-0.44920900
C	-0.37872300	0.88334000	-1.84999700
H	-1.27348100	0.58003400	-2.42326600
C	-0.44116900	2.23293200	-1.25529700
C	0.62058800	2.74491200	-0.48514200
C	-1.59250300	3.00967300	-1.46242400
C	0.51966500	4.01975600	0.06988800
H	1.51168400	2.14438100	-0.32993100
C	-1.68897500	4.28654800	-0.90766900
H	-2.41496600	2.60399800	-2.04707200
C	-0.63356800	4.79065300	-0.14099000
H	1.34062500	4.41833400	0.66043400
H	-2.58369000	4.88211100	-1.06469900
H	-0.70650500	5.78474200	0.29266300
F	-1.85890200	-2.46842500	-1.03805700
F	-4.43643200	-2.93252400	-1.69759100
F	-6.43029900	-1.28964000	-0.79055800
F	-5.81969700	0.84875200	0.80924500
F	-3.24472100	1.35183000	1.51118600
O	0.57984300	0.12177200	-1.76178100
O	3.65752800	0.84274600	0.57060500
C	4.62415000	0.58634500	-0.48781100
C	4.65022600	-0.99420000	-0.54638300
O	3.32079500	-1.34269300	-0.05939500
B	2.87710000	-0.28772200	0.72954200
C	4.08528000	1.23626000	-1.76957700
H	3.94488600	2.30785800	-1.59133100
H	4.78897400	1.12321300	-2.60195000
H	3.12057900	0.81270200	-2.06457400
C	5.95184100	1.23396700	-0.09535100
H	5.83277500	2.32221900	-0.06045200
H	6.29170000	0.90220100	0.88859700
H	6.73099800	1.00304200	-0.83178500
C	5.65526100	-1.62696700	0.42654800
H	5.47767500	-2.70628700	0.46994000
H	6.68824300	-1.45871100	0.10277300
H	5.54061200	-1.22988100	1.44038800
C	4.83004000	-1.58777200	-1.94256100
H	5.79602600	-1.29241200	-2.36837800
H	4.81309300	-2.68189100	-1.88306500
H	4.04022100	-1.26755500	-2.62751400
C	1.30141900	-1.49565800	2.38753300

O	1.53720200	-2.59471600	1.88829000
O	0.70973000	-1.07937200	3.38076300
O	1.78250000	-0.29397100	1.53411200
K	0.76793500	-2.38861300	-0.62091400
K	-0.65111500	0.86371200	2.22826100

10-ts

C	-4.54637200	-0.41225900	0.76349200
C	-3.33810500	0.25411600	0.57317800
C	-2.37179400	-0.11990500	-0.33549000
C	-2.67609700	-1.25320400	-1.05445500
C	-3.85607600	-1.98087500	-0.93371900
C	-4.80613100	-1.54372100	-0.01036800
C	-0.58362500	1.06853900	-1.27705500
H	-1.03388200	0.68935200	-2.20450900
C	-0.97613300	2.47374700	-0.94180800
C	-0.12585800	3.27618800	-0.16589300
C	-2.15157600	3.03132400	-1.46687100
C	-0.46165700	4.60508900	0.10757200
H	0.81990900	2.86415100	0.17869300
C	-2.48809300	4.35632200	-1.19500200
H	-2.81087600	2.41333700	-2.07218700
C	-1.64721400	5.14533100	-0.39992900
H	0.20746000	5.22231000	0.70212400
H	-3.40627000	4.77581800	-1.59775900
H	-1.91059400	6.17819900	-0.18761800
F	-1.75561900	-1.74898500	-1.98136500
F	-4.10047400	-3.08522100	-1.66895500
F	-5.95916200	-2.21708500	0.14230800
F	-5.45672000	-0.00721700	1.67205100
F	-3.10710800	1.34515100	1.40033900
O	0.47453200	0.55807700	-0.83552700
O	4.02001300	0.37935600	1.33521400
C	4.84093600	0.64420500	0.16679500
C	4.67583600	-0.68770000	-0.66440800
O	3.34898100	-1.13929700	-0.25567200
B	3.09113500	-0.58237300	0.99253600
C	4.24355600	1.87427700	-0.53299100
H	4.22527000	2.70725400	0.17799800
H	4.84292700	2.17960700	-1.39819300
H	3.21687900	1.68841700	-0.86576800
C	6.26656500	0.94184900	0.62838100
H	6.27687000	1.87010400	1.20943800
H	6.66248900	0.14491400	1.26248800
H	6.93545600	1.07288600	-0.23090000
C	5.66487900	-1.78797200	-0.25705100

H	5.36450100	-2.73152700	-0.72454600
H	6.68418900	-1.55188500	-0.58188700
H	5.67182100	-1.94027800	0.82686000
C	4.68288500	-0.50636700	-2.18128300
H	5.63773800	-0.08548700	-2.51738800
H	4.56110900	-1.47872500	-2.67344500
H	3.87881300	0.15521800	-2.51497800
C	1.15081400	-2.02615600	1.78849700
O	1.51918500	-2.95555800	1.05640000
O	0.15020500	-1.84525800	2.49447100
O	2.03232600	-0.83270200	1.80841200
K	0.95562400	-2.07751000	-1.33046900
K	-0.38773600	0.64195700	1.94486700
**11**			
C	-4.57005300	-0.52949400	0.97168100
C	-3.34564900	0.06554300	0.68935500
C	-2.79054200	0.11619700	-0.59074600
C	-3.55133400	-0.48849100	-1.59300200
C	-4.78212100	-1.10006400	-1.35250300
C	-5.29673900	-1.12143900	-0.06020900
C	-1.36341900	0.66500100	-0.86330400
H	-1.27959800	0.67893000	-1.96956100
C	-1.24796900	2.15370800	-0.45533700
C	-0.01404700	2.63591400	0.00206400
C	-2.30409900	3.06609200	-0.59744200
C	0.15914700	3.98645400	0.32670200
H	0.81757500	1.93902800	0.06898300
C	-2.13892400	4.41514400	-0.27074800
H	-3.26997500	2.72357800	-0.96319000
C	-0.90635900	4.88176200	0.19792500
H	1.12803600	4.33987400	0.67338000
H	-2.97477900	5.10218600	-0.38060700
H	-0.77771800	5.93057600	0.45311300
F	-3.10373500	-0.50559700	-2.87133500
F	-5.47150300	-1.66845300	-2.35561200
F	-6.47669500	-1.70542800	0.19250400
F	-5.05539500	-0.54118400	2.22434400
F	-2.68220500	0.61538900	1.74379100
O	-0.42919200	-0.15664300	-0.28967200
O	4.51886000	0.22993500	1.08804600
C	5.12505300	0.67400700	-0.15479700
C	4.69150900	-0.46229300	-1.16321400
O	3.43319400	-0.91895200	-0.57925000
B	3.47349000	-0.61306600	0.77892900
C	4.51917800	2.04516900	-0.48788100

H	4.69708500	2.72309500	0.35333700
H	4.97485900	2.48307400	-1.38293400
H	3.43759300	1.98093000	-0.64836300
C	6.63216400	0.80570000	0.05854100
H	6.83003200	1.59927800	0.78682400
H	7.06894500	-0.11885000	0.44345700
H	7.13784300	1.07128700	-0.87775000
C	5.64386300	-1.66566500	-1.17006300
H	5.18743100	-2.48338700	-1.73767200
H	6.60130500	-1.41513000	-1.63968800
H	5.83738900	-2.03203100	-0.15707100
C	4.42473400	0.00799000	-2.59197700
H	5.33166200	0.43590500	-3.03496200
H	4.12547000	-0.84331500	-3.21490000
H	3.63221200	0.75975800	-2.63348300
C	1.84176600	-2.28350400	1.77295800
O	2.19402500	-3.11587800	0.92493500
O	0.97067400	-2.27405100	2.65026200
O	2.56076600	-0.97988100	1.71918700
K	0.99258900	-2.07938200	-1.18248800
K	0.00159100	0.12966500	2.31128400

12-ts

C	1.89045500	2.92142600	-0.25955200
C	2.13961800	2.72834400	1.29886300
O	2.23954700	1.61858200	-0.79508900
O	2.00559600	1.29308700	1.47715200
B	2.14545000	0.71290600	0.23655800
O	2.52961100	-0.62853100	0.06541200
C	3.63837400	-1.04055900	-0.62428700
O	4.68272500	-0.34817200	-0.59777600
O	3.49295300	-2.16249400	-1.22074000
C	3.56846900	3.08554500	1.73776300
H	3.75494900	4.16447600	1.68188900
H	3.70549900	2.76704900	2.77693800
H	4.31273800	2.56528100	1.12630100
C	1.12625000	3.43028200	2.20249300
H	1.35322500	3.20298200	3.25006100
H	1.17374400	4.51879400	2.07318000
H	0.10520500	3.09903500	1.99908700
C	2.78935300	3.96647100	-0.92377800
H	2.56979300	4.00469000	-1.99667800
H	2.60450300	4.96380200	-0.50576700
H	3.84893800	3.72641200	-0.80426400
C	0.42454900	3.18806200	-0.62443900
H	0.08546800	4.16240400	-0.25446700

H	0.32872600	3.18254800	-1.71613100
H	-0.22022400	2.39872600	-0.23396500
C	-3.89777800	-0.83607500	-1.79066000
C	-2.82175400	-0.93308100	-0.91654600
C	-2.47539500	0.06991900	-0.00799300
C	-3.30320200	1.19337000	-0.01905400
C	-4.39051400	1.33159000	-0.88340400
C	-4.69060000	0.31027100	-1.77783500
C	-1.17276300	-0.00880700	0.83901700
H	-1.15257800	0.93312300	1.42669800
C	-1.27863000	-1.13186400	1.90456200
C	-0.09042300	-1.70315000	2.38673100
C	-2.49301100	-1.56927700	2.45365200
C	-0.11709200	-2.69171100	3.37614200
H	0.85545600	-1.33823800	1.99749900
C	-2.52515200	-2.56273300	3.43844600
H	-3.43007100	-1.13537200	2.11119500
C	-1.33602200	-3.13279900	3.90204800
H	0.81773300	-3.10976400	3.74478500
H	-3.48067000	-2.89077400	3.84215700
H	-1.35862800	-3.90364800	4.66881200
F	-3.07300800	2.21828600	0.83327800
F	-5.15160000	2.44025900	-0.85716800
F	-5.73234900	0.42278200	-2.61893400
F	-4.18317800	-1.83734300	-2.64592600
F	-2.09523100	-2.08456200	-0.98485100
O	-0.10308700	-0.11906800	0.00354600
K	0.72419900	-2.32606400	-0.88250400
K	5.95106900	-2.00180100	-2.04595700
**13**			
C	-2.77645200	-1.03077300	-1.83590800
C	-2.02983700	-2.19903700	-1.08119300
O	-2.68870200	0.04941800	-0.89683100
O	-1.01744600	-1.50285700	-0.35248700
B	-1.49844500	-0.14750500	-0.04837100
O	-1.76316200	-0.10227800	1.40337600
C	-2.23819300	0.93610300	2.21767200
O	-2.21538600	0.63768700	3.44165100
O	-2.60966000	2.00371800	1.67569200
C	-2.95040000	-2.92977900	-0.08045500
H	-3.71029600	-3.54198100	-0.58090100
H	-2.33422300	-3.60262100	0.53090400
H	-3.45450000	-2.21857800	0.58143200
C	-1.36323300	-3.23042400	-1.99724400
H	-0.87672000	-4.00348100	-1.39032100

H	-2.09767100	-3.72590000	-2.64519800
H	-0.59764300	-2.76808700	-2.62553900
C	-4.25396400	-1.30244300	-2.13722800
H	-4.68806900	-0.44910400	-2.67307400
H	-4.37429700	-2.18898900	-2.77272800
H	-4.82945800	-1.44716700	-1.21913800
C	-2.05675700	-0.61884800	-3.13490100
H	-2.14281200	-1.38527600	-3.91441300
H	-2.51774300	0.29932500	-3.52199600
H	-0.99727700	-0.41752000	-2.95255100
C	3.08564400	-1.37432200	-1.37021600
C	2.21427100	-0.30935900	-1.15622300
C	1.66113500	-0.02602600	0.09672300
C	2.03336800	-0.89119800	1.12326600
C	2.89704700	-1.96778400	0.94724200
C	3.43191000	-2.21193300	-0.31234400
C	0.63845300	1.10578600	0.33083000
H	0.38521600	1.07042200	1.39600100
C	1.24214500	2.48616900	0.06825400
C	1.08385400	3.13974800	-1.16160200
C	1.95182200	3.13326200	1.08892100
C	1.62529100	4.41312800	-1.36442900
H	0.55136500	2.63679000	-1.96288200
C	2.49761600	4.40385000	0.88798200
H	2.06483100	2.64646100	2.05578200
C	2.33507400	5.04947200	-0.34126200
H	1.50174500	4.90433400	-2.32705900
H	3.03848600	4.89268400	1.69439600
H	2.75515400	6.03945900	-0.49971700
F	1.52251600	-0.73000200	2.38016300
F	3.20025100	-2.77628900	1.98073700
F	4.26691300	-3.24210400	-0.50670700
F	3.59176100	-1.60473100	-2.59186300
F	1.90565600	0.44573400	-2.22472900
O	-0.51162200	0.91133900	-0.44375500
K	-2.54950400	2.69329300	-0.72126700
K	-1.04484700	-1.65468800	3.26768700

14-ts

C	1.48107000	3.56279300	-0.48309300
C	1.63257800	2.68805100	-1.78999800
O	0.82984900	2.65782700	0.42348300
O	1.80481600	1.36737600	-1.26742400
B	1.17347400	1.28001000	0.04602700
O	-0.00237100	0.37060400	-0.09034100
C	-0.88228100	-0.05335400	0.87674000

O	-1.57730800	-1.05392300	0.49078700
O	-0.94527300	0.52819200	1.97924000
C	0.36445900	2.71277500	-2.66496500
H	0.21412100	3.68229300	-3.15406000
H	0.46929500	1.95582900	-3.45270900
H	-0.52940900	2.48144200	-2.07838400
C	2.84659100	3.04130100	-2.65551900
H	2.87793700	2.38319100	-3.53200900
H	2.79259100	4.07665800	-3.01534700
H	3.78284400	2.91072900	-2.10659100
C	0.60715300	4.81049600	-0.64488000
H	0.55678500	5.35689800	0.30520700
H	1.02381300	5.49175500	-1.39744100
H	-0.41386900	4.55137500	-0.93600400
C	2.84210600	3.96487500	0.11702300
H	3.36585200	4.69961000	-0.50564200
H	2.67645800	4.42520100	1.10016500
H	3.48871200	3.09293000	0.25122800
C	5.42859900	-0.65892300	-1.35313400
C	4.62318100	-0.38620000	-0.25071100
C	3.36170500	-0.96329800	-0.07538300
C	2.95200400	-1.82868800	-1.08844200
C	3.72752200	-2.12098800	-2.20635200
C	4.97981800	-1.53264900	-2.34076200
C	2.43241400	-0.61448700	1.10444200
H	1.53372800	-1.22619300	0.96929800
C	3.03685600	-1.01416500	2.45104900
C	3.72332600	-0.09479000	3.25600500
C	2.88384000	-2.32936200	2.91078800
C	4.24596600	-0.48434500	4.49320000
H	3.86320600	0.92040800	2.89778300
C	3.40847000	-2.72281600	4.14470700
H	2.33696800	-3.04938300	2.30479200
C	4.09218700	-1.79989800	4.94167200
H	4.78464600	0.23813000	5.10223600
H	3.27398800	-3.74588000	4.48682200
H	4.49930400	-2.10212500	5.90314400
F	1.72348100	-2.41763300	-1.03833000
F	3.26473900	-2.95281300	-3.15895200
F	5.74444300	-1.80104300	-3.40801600
F	6.63357800	-0.08036700	-1.47546100
F	5.10451900	0.48633000	0.65218000
O	2.09280500	0.74762000	1.10217700
C	-4.06972800	-3.46346200	0.54778100
C	-3.91110800	-3.35999200	-1.02222800

O	-3.39140100	-2.01037400	-1.20493300
O	-4.24832000	-2.07626100	0.92716800
B	-3.67629300	-1.28333900	-0.03837900
C	-3.92206600	0.28673500	-0.05176700
C	-3.59245800	1.09632800	-1.14052600
C	-4.46497900	0.96592200	1.04674300
C	-3.78061100	2.47384800	-1.16985000
C	-4.66557100	2.34606400	1.05911000
C	-4.32333200	3.10548600	-0.05493600
C	-2.81675800	-3.98492800	1.26803200
H	-2.96465100	-3.87018600	2.34691100
H	-2.64545700	-5.04716700	1.05598000
H	-1.93100000	-3.40535300	0.99754600
C	-5.29813100	-4.24521100	1.01700100
H	-5.24159800	-5.29288300	0.69650100
H	-5.34110600	-4.22747800	2.11095600
H	-6.22723800	-3.81192500	0.63851200
C	-5.24448700	-3.42364900	-1.78164800
H	-5.07030400	-3.16866500	-2.83279500
H	-5.68759900	-4.42498400	-1.73985900
H	-5.96695300	-2.70747400	-1.37779000
C	-2.91987200	-4.35154600	-1.63324700
H	-3.25703900	-5.38384000	-1.48287300
H	-2.84467600	-4.18444400	-2.71495800
H	-1.92372400	-4.25556700	-1.19127300
F	-4.82153700	0.30379200	2.15750600
F	-5.18383600	2.95330300	2.14085100
F	-4.50948100	4.43408300	-0.05425500
F	-3.43650700	3.19639200	-2.25146000
F	-3.02811900	0.55116000	-2.25660200
K	0.72278300	2.10386900	3.01686000
K	-0.80574100	-1.17676900	-2.03249700
**15**			
C	1.47875000	3.70467900	-0.03358300
C	1.72915200	3.00949800	-1.42837700
O	0.86344200	2.64946500	0.73117400
O	1.98688700	1.64925600	-1.05477600
B	1.29023300	1.36352100	0.18177200
O	0.12384900	0.44884600	-0.17639500
C	-0.82342500	-0.01879400	0.64069200
O	-1.47788000	-0.99276800	0.04567300
O	-1.03420000	0.39207700	1.78408600
C	0.48697500	3.04791200	-2.33956900
H	0.28251600	4.05545700	-2.71961200
H	0.67104900	2.39779800	-3.20427500

H	-0.40534600	2.68829800	-1.81855400
C	2.93846200	3.54393100	-2.20150200
H	3.03739100	3.00333800	-3.14997500
H	2.82341200	4.61015000	-2.43376500
H	3.86612700	3.40598200	-1.63997000
C	0.52487000	4.90149800	-0.07403100
H	0.41069000	5.32276200	0.93237100
H	0.91423000	5.69546200	-0.72324900
H	-0.46712700	4.61330200	-0.43070100
C	2.78864600	4.10975200	0.66672800
H	3.28054800	4.95084500	0.16459700
H	2.56231900	4.42630300	1.69330000
H	3.48914400	3.27053400	0.71263200
C	5.57570200	-0.34516200	-1.27290300
C	4.72416900	-0.23745500	-0.17667200
C	3.46235600	-0.83826000	-0.14043900
C	3.09868300	-1.54772300	-1.28461600
C	3.92212100	-1.67224600	-2.39939600
C	5.17450700	-1.06819300	-2.39403600
C	2.48217500	-0.67226400	1.03428300
H	1.60261600	-1.27031000	0.77291300
C	3.02709000	-1.24425300	2.34258200
C	3.65575200	-0.43576400	3.29950000
C	2.87216100	-2.61133200	2.61028300
C	4.12015900	-0.98558000	4.49856600
H	3.79768100	0.61994800	3.09024300
C	3.33936400	-3.16403100	3.80559500
H	2.36997200	-3.24774500	1.88406700
C	3.96516000	-2.35136800	4.75541400
H	4.61480400	-0.34803200	5.22777700
H	3.20513200	-4.22538400	3.99792200
H	4.32708200	-2.77780000	5.68736700
F	1.87463900	-2.13835300	-1.36936500
F	3.50534600	-2.35670400	-3.48092500
F	5.98401000	-1.17745200	-3.45561700
F	6.77872600	0.24902600	-1.26078300
F	5.15873200	0.49713100	0.86229500
O	2.11174000	0.67537000	1.20952200
C	-3.54583200	-3.56089300	0.25083300
C	-3.82668500	-3.16267500	-1.24866500
O	-3.08700800	-1.93631600	-1.37840800
O	-3.46080800	-2.28477400	0.89323800
B	-2.99713900	-1.30800300	-0.04078100
C	-3.78440900	0.14374800	0.05598900
C	-3.69283900	1.08346800	-0.96940900

C	-4.58446100	0.54357300	1.13102300
C	-4.34192700	2.31404300	-0.97800200
C	-5.25593700	1.76753800	1.17100800
C	-5.13692400	2.65949600	0.11001100
C	-2.20634200	-4.30212300	0.43269200
H	-1.98315600	-4.35798900	1.50371700
H	-2.24459000	-5.32399500	0.03523100
H	-1.38320000	-3.76653700	-0.05069900
C	-4.66222900	-4.36864300	0.91967200
H	-4.82832800	-5.32252300	0.40221200
H	-4.38398300	-4.58903600	1.95637800
H	-5.60183200	-3.81083000	0.93981900
C	-5.31268900	-2.86481500	-1.51895300
H	-5.40850900	-2.40079300	-2.50736100
H	-5.92190500	-3.77627800	-1.50778700
H	-5.71725000	-2.16883900	-0.77784900
C	-3.31390100	-4.16184800	-2.29032400
H	-3.80755400	-5.13531600	-2.18085800
H	-3.53126200	-3.79170900	-3.30015600
H	-2.23369600	-4.31824800	-2.20592600
F	-4.74467300	-0.24473900	2.21043200
F	-6.01360900	2.10261100	2.23315200
F	-5.77651900	3.84150600	0.13650800
F	-4.20273600	3.16966200	-2.01065300
F	-2.90171900	0.83402900	-2.06398200
K	0.59412200	1.74635000	3.20938700
K	-0.76476900	-0.89373800	-2.44852300

16-ts

C	1.74961600	-3.46780700	-1.39753700
C	2.01912300	-3.40549000	0.16252000
O	1.27412000	-2.12794100	-1.69321700
O	2.33988800	-2.01158200	0.36008300
B	1.76417300	-1.27282800	-0.67168700
O	-1.99581800	-0.07607300	0.93371500
C	-0.61411700	-0.01434200	0.87375800
O	-0.08957700	-0.76195200	-0.00844400
O	-0.03394200	0.70935700	1.71292900
C	0.78774700	-3.74545500	1.01768600
H	0.54018900	-4.81222400	0.96150400
H	1.01287100	-3.50212800	2.06158300
H	-0.08347700	-3.15515400	0.72332800
C	3.21080500	-4.24202300	0.63476100
H	3.33731000	-4.11704100	1.71561400
H	3.04738000	-5.30833500	0.43508800
H	4.14048500	-3.93390300	0.15018200

C	0.68021300	-4.47626100	-1.82222100
H	0.53136200	-4.42595300	-2.90810600
H	0.98755900	-5.50050600	-1.58025300
H	-0.27815100	-4.28981600	-1.32761000
C	3.02337300	-3.68740800	-2.22855200
H	3.42350500	-4.69995800	-2.10354200
H	2.78646500	-3.54054300	-3.28811000
H	3.80272200	-2.96819200	-1.95949500
C	6.42656500	-0.40381100	-0.00347200
C	5.18136000	0.02803700	-0.45435200
C	4.15316200	0.39351600	0.41952500
C	4.44907500	0.32003200	1.78206900
C	5.68604800	-0.10353700	2.26292700
C	6.68191700	-0.47098700	1.36375100
C	2.76983300	0.85703000	-0.05973000
H	2.10582300	0.87253200	0.81021700
C	2.85620900	2.27237000	-0.62499300
C	2.94461100	2.51352800	-2.00098500
C	2.87137600	3.35988000	0.25965400
C	3.05305800	3.82166200	-2.48264000
H	2.92483300	1.67354600	-2.68711300
C	2.98172800	4.66725300	-0.22057500
H	2.79123000	3.18102400	1.33007700
C	3.07427300	4.90260700	-1.59617300
H	3.12305400	3.99569800	-3.55396400
H	2.99186900	5.50059400	0.47810100
H	3.16042300	5.91880700	-1.97314700
F	3.52095500	0.66776800	2.69683800
F	5.92247800	-0.16172400	3.58484700
F	7.87949100	-0.88041500	1.80897200
F	7.38486000	-0.75281300	-0.87930100
F	4.99711800	0.07566600	-1.78703300
O	2.23723300	-0.02197000	-1.03905600
C	-2.40794700	2.27768200	-1.61406700
C	-3.25915800	2.97820900	-0.48841000
O	-3.10576400	2.07244900	0.61194200
O	-2.58362200	0.87747200	-1.31276000
B	-2.95711800	0.69310800	0.09424800
C	-4.35428200	-0.21054900	0.27337300
C	-5.13626000	-0.12803400	1.42419200
C	-4.80608100	-1.15436700	-0.64533500
C	-6.29596000	-0.86626500	1.64441400
C	-5.95827000	-1.92160600	-0.48285400
C	-6.71783500	-1.77065500	0.67330500
C	-0.91213400	2.61875600	-1.52756800

H	-0.34583300	1.96585300	-2.20086200
H	-0.71099900	3.65286000	-1.82961100
H	-0.51674300	2.46870600	-0.51973300
C	-2.90734500	2.53557700	-3.04012500
H	-2.88944500	3.60657400	-3.27683600
H	-2.25357400	2.03186500	-3.76339300
H	-3.92429100	2.16252800	-3.18752600
C	-4.75461200	3.08643300	-0.84298300
H	-5.30918900	3.39625700	0.04993200
H	-4.93338300	3.82881100	-1.62949100
H	-5.16256100	2.12760500	-1.17617800
C	-2.74555200	4.35822500	-0.06556500
H	-2.76760400	5.06458500	-0.90435700
H	-3.38839200	4.76406200	0.72532400
H	-1.72027900	4.31048600	0.31226500
F	-4.09312600	-1.41089300	-1.78686900
F	-6.33453800	-2.81322900	-1.41854000
F	-7.83189400	-2.49675000	0.85657300
F	-7.00332800	-0.72719000	2.78090400
F	-4.76604700	0.70132900	2.45009000
K	-2.10589500	1.67069500	3.00688600
K	-1.27650100	-1.24948800	-2.24053600

17

C	-4.00064800	-0.33220600	-0.79214500
C	-3.71036500	-0.34294100	0.76461900
O	-2.92200500	0.50349600	-1.30547900
O	-2.26370400	-0.14139000	0.80776100
B	-1.90990000	0.46139700	-0.37944500
C	-4.34511300	0.83778900	1.51186900
H	-5.43429300	0.73613800	1.57105600
H	-3.94744300	0.86803800	2.53165300
H	-4.11330500	1.79328400	1.02944100
C	-4.03728400	-1.65344200	1.47647400
H	-3.77480400	-1.57174900	2.53663100
H	-5.10913400	-1.87440400	1.40871300
H	-3.48062000	-2.49418300	1.05564800
C	-5.33273600	0.29644100	-1.19573400
H	-5.43252700	0.27563500	-2.28612200
H	-6.17307300	-0.26274800	-0.76725200
H	-5.40350300	1.33755300	-0.87198300
C	-3.84743200	-1.71066900	-1.45008900
H	-4.66239100	-2.38583000	-1.16672400
H	-3.86548800	-1.58659900	-2.53765100
H	-2.89700400	-2.18345300	-1.18061600
C	2.60390000	-1.72384700	-1.28367300

C	1.81963700	-0.58738800	-1.10061400
C	1.23235400	-0.27803700	0.13173000
C	1.48173100	-1.16563600	1.18086100
C	2.26668900	-2.30710600	1.02775300
C	2.82783200	-2.58912600	-0.21415000
C	0.35641800	0.95886900	0.33848800
H	-0.08988000	0.87227000	1.33266000
C	1.16695500	2.25028300	0.28342600
C	1.12947700	3.09801200	-0.82874000
C	1.97120900	2.59033400	1.38061400
C	1.89372100	4.26909800	-0.84315700
H	0.50115500	2.84036200	-1.67427800
C	2.73623700	3.75831200	1.36375600
H	1.99648300	1.94333600	2.25573400
C	2.69989000	4.60182000	0.24864500
H	1.85702700	4.92183400	-1.71173400
H	3.35323000	4.01202600	2.22201600
H	3.29229800	5.51306500	0.23379300
F	0.96585600	-0.92921200	2.40495000
F	2.47980000	-3.13274800	2.06353200
F	3.58235200	-3.68252700	-0.38007600
F	3.14727000	-1.99046000	-2.48045900
F	1.64297400	0.22024100	-2.15786800
O	-0.67758300	0.98132300	-0.64404000
**18**			
C	2.23586900	-0.89264300	-1.34664000
C	2.58542700	0.61772200	-1.06332100
O	1.36882900	1.11524500	-0.49251100
O	1.36497800	-1.20841700	-0.25634100
B	0.62298700	0.00728200	0.15995600
C	-0.97174600	-0.00315100	-0.35230900
C	-1.72148100	-1.17293100	-0.48610700
C	-1.68210000	1.15210900	-0.68207100
C	-3.03954100	-1.20463100	-0.93223400
C	-2.99966000	1.15355800	-1.13031100
C	-3.70415400	-0.03400200	-1.26912600
O	0.68595400	0.11536700	1.63626400
C	-0.41534000	0.19683700	2.55383800
O	-0.78394900	1.35928100	2.83422300
O	-0.82029800	-0.90321300	2.99105200
C	3.71820500	0.77429900	-0.02898800
H	4.69137400	0.46584400	-0.42934200
H	3.80148900	1.83235400	0.25223300
H	3.50838500	0.19714500	0.87670200
C	2.92084000	1.44027000	-2.31152900

H	3.15483800	2.47352500	-2.02689800
H	3.79646900	1.03223000	-2.83222100
H	2.08001000	1.46849300	-3.00894400
C	3.43466300	-1.84664900	-1.31164300
H	3.09930800	-2.86954800	-1.52211100
H	4.17839500	-1.57893200	-2.07234000
H	3.92523800	-1.84261500	-0.33345600
C	1.47616800	-1.08756900	-2.67312000
H	2.12914300	-0.94518200	-3.54243900
H	1.08028000	-2.10875500	-2.70743600
H	0.63245200	-0.39656700	-2.75787000
F	-1.17662200	-2.38804500	-0.15993300
F	-3.68079300	-2.39191300	-1.03449200
F	-3.60164100	2.32821300	-1.42930800
F	-1.09622900	2.38538700	-0.55728100
K	0.70624300	2.89049100	1.41887200
K	0.66167000	-2.65403100	1.84669300
H	-4.73211600	-0.04533900	-1.61240900

19-ts

C	0.12417100	2.78847800	-0.29409900
C	0.63787500	2.37005800	1.15225300
O	0.65748300	0.92253600	1.08506500
O	0.45936900	1.63218700	-1.09939700
B	0.63969800	0.55668700	-0.25483900
C	-1.50837300	-0.62278100	-0.30314000
C	-2.49882400	-0.23715600	-1.19568800
C	-2.00704700	-1.22972100	0.83238700
C	-3.86466700	-0.39289000	-0.95509300
C	-3.35565200	-1.41442600	1.12704600
C	-4.31844600	-0.98115100	0.22286300
O	1.33629500	-0.60868800	-0.68988300
C	2.66490300	-0.83501400	-0.48407400
O	3.46492900	0.12150600	-0.35622000
O	2.97599600	-2.08041400	-0.46218100
C	2.07973200	2.81656700	1.44525500
H	2.15567000	3.90623600	1.54276700
H	2.39807000	2.36544800	2.39222100
H	2.76508100	2.47270900	0.66563700
C	-0.27464400	2.79245600	2.30569300
H	0.14230000	2.42794100	3.25131200
H	-0.34983700	3.88544600	2.36660400
H	-1.27991300	2.37794400	2.20134500
C	0.83976800	3.99875000	-0.90046400
H	0.43938100	4.19026600	-1.90210100
H	0.67746300	4.89719200	-0.29195800

H	1.91477400	3.82593100	-0.99466000
C	-1.39325100	3.00928800	-0.37184800
H	-1.68724200	3.93014100	0.14592400
H	-1.68408600	3.09386400	-1.42373000
H	-1.94904400	2.17434400	0.05943100
F	-2.16286500	0.33525600	-2.39446600
F	-4.78063800	0.02186600	-1.87314300
F	-3.74712700	-2.02836900	2.27988300
F	-1.10621700	-1.77114500	1.76553200
K	5.47290900	-1.42247700	-0.21026600
K	0.36403200	-2.92031000	-0.23513700
H	-5.37723100	-1.10819700	0.41819300

20

C	2.14090500	-1.11386400	-1.19806800
C	2.48651400	0.42358600	-1.18342000
O	1.27151300	1.00991900	-0.70443600
O	1.27462500	-1.23776900	-0.06802300
B	0.52248800	0.02920800	0.13097900
C	-1.06495900	-0.07197100	-0.36804500
C	-1.83110100	-1.23877000	-0.30657100
C	-1.79666700	0.99001900	-0.90559600
C	-3.14112200	-1.39791200	-0.74686200
C	-3.10509900	0.94525800	-1.37568400
C	-3.75457500	-0.27716300	-1.28658700
O	0.59876100	0.38748700	1.57079400
C	-0.49425900	0.63866900	2.46483400
O	-0.87738200	1.82950800	2.51508300
O	-0.88018800	-0.35628100	3.11886500
C	3.62364900	0.76214100	-0.19813900
H	4.59512300	0.38441100	-0.53886100
H	3.70958700	1.85361300	-0.11318100
H	3.41611500	0.35948200	0.79804100
C	2.81388600	1.01776400	-2.55760200
H	3.04006800	2.08662200	-2.45772500
H	3.69165400	0.53270800	-3.00361500
H	1.97123400	0.91833700	-3.24633900
C	3.34251900	-2.04487200	-1.00164900
H	3.00823300	-3.08938000	-1.02743100
H	4.08335800	-1.91486300	-1.80047500
H	3.83703800	-1.86928600	-0.04138800
C	1.37835100	-1.53789000	-2.46836400
H	2.02738200	-1.54520800	-3.35219100
H	0.98696500	-2.55111100	-2.32355200
H	0.53131500	-0.87450300	-2.66497400
F	-1.27284000	-2.37323100	0.24453300

F	-1.20373200	2.23242300	-0.98584700
K	0.61074500	3.07726400	0.85519700
K	0.58093500	-2.28174200	2.25813500
F	-5.03304700	-0.37722900	-1.73247100
H	-3.59463700	1.82406100	-1.77846400
H	-3.65811800	-2.34640100	-0.66173700

21-ts

C	-0.08195000	2.71444500	-0.46642300
C	0.43722900	2.39517200	1.00472600
O	0.53324100	0.94986600	1.01157600
O	0.31133500	1.53454400	-1.20787800
B	0.54114700	0.51559000	-0.30726400
C	-1.58923500	-0.77025800	-0.30856700
C	-2.60253800	-0.44491800	-1.20462400
C	-2.12431800	-1.28718800	0.85599300
C	-3.98125200	-0.55007500	-0.99317600
C	-3.46570400	-1.44767500	1.21043900
C	-4.37890800	-1.04903000	0.24175500
O	1.29012200	-0.63620200	-0.68013100
C	2.61152100	-0.82072700	-0.39982700
O	3.38419700	0.16065100	-0.28885200
O	2.94861100	-2.05481900	-0.29626700
C	1.84970100	2.93346500	1.28473000
H	1.86634900	4.02953300	1.31526600
H	2.18040300	2.55795900	2.26008800
H	2.56158600	2.58034600	0.53357100
C	-0.50921100	2.82614200	2.12742400
H	-0.08485900	2.53056100	3.09365400
H	-0.64112700	3.91546800	2.13339400
H	-1.49058700	2.35542200	2.03418000
C	0.58589000	3.92166000	-1.13131200
H	0.18990900	4.03873600	-2.14614800
H	0.37524700	4.84424400	-0.57596400
H	1.66880000	3.79415400	-1.20512900
C	-1.60649100	2.86570400	-0.56539900
H	-1.94325600	3.79645200	-0.09331900
H	-1.89033100	2.88963100	-1.62243800
H	-2.13024400	2.02842300	-0.10051000
F	-2.25232500	0.03868700	-2.44603000
F	-1.21594400	-1.78386100	1.82199800
K	5.41185200	-1.32333500	0.04637900
K	0.33280300	-2.93652900	-0.13529200
F	-5.71320100	-1.17021600	0.50612600
H	-4.70703700	-0.26036800	-1.74575100
H	-3.78424600	-1.85520000	2.16388800

22

C	2.25218800	-0.89522500	-0.86253000
C	2.48956800	0.63543900	-0.57535400
O	1.16020000	1.11848100	-0.35874100
O	1.14472400	-1.19122600	-0.00995600
B	0.28684700	0.01648800	0.13792600
C	-1.11692400	-0.06926400	-0.75556900
C	-1.80386300	-1.25647900	-1.02440400
C	-1.75363700	1.02907900	-1.34029500
C	-2.94838200	-1.39023300	-1.80138300
C	-2.89573000	0.99668600	-2.13134800
C	-3.49737300	-0.23980400	-2.36867100
O	-0.01611700	0.19127700	1.58189900
C	-1.30266200	0.28871100	2.20720700
O	-1.75758900	1.45071400	2.30981700
O	-1.77188700	-0.79499500	2.62235800
C	3.32066900	0.87009200	0.70271000
H	4.36856100	0.57150500	0.57714700
H	3.31203800	1.94146900	0.94330800
H	2.89704200	0.32764000	1.55343900
C	3.11570200	1.41535500	-1.73646800
H	3.24422400	2.46735400	-1.45278300
H	4.10518300	1.01781000	-1.99616100
H	2.48101800	1.38491100	-2.62562100
C	3.42303600	-1.80949400	-0.48475300
H	3.16877400	-2.85069700	-0.71849900
H	4.32748400	-1.55440100	-1.05108100
H	3.65577600	-1.74708200	0.58275100
C	1.85159200	-1.16823700	-2.32543700
H	2.69677200	-1.03894000	-3.01217100
H	1.49906600	-2.20252500	-2.40768400
H	1.03909600	-0.50985000	-2.64584700
F	-1.33257300	-2.43567500	-0.47407400
F	-1.23165200	2.29164500	-1.12067800
K	-0.00630100	2.94623200	1.20178400
K	-0.04347300	-2.55004200	1.91336700
H	-3.29907100	1.91973300	-2.53486600
H	-3.39296100	-2.36995500	-1.94271700
H	-4.39327600	-0.30451300	-2.97918700

23-ts

C	-0.59147600	2.61627100	-0.46004900
C	0.00057900	2.36578000	0.99684600
O	0.25220100	0.93934100	1.00736800
O	-0.09819700	1.47827000	-1.20697700
B	0.26379200	0.49739200	-0.30836600

C	-1.74745200	-1.00132000	-0.23540400
C	-2.80497500	-0.78552200	-1.11370300
C	-2.20863300	-1.55742900	0.94379500
C	-4.15897000	-1.02440700	-0.86650600
C	-3.52106700	-1.84069300	1.31949000
C	-4.51672400	-1.55039500	0.38014900
O	1.10946600	-0.57930400	-0.69576300
C	2.44701300	-0.63432800	-0.43715400
O	3.12550400	0.41767400	-0.35800100
O	2.90136200	-1.82854000	-0.31893200
C	1.35426500	3.05398100	1.23548400
H	1.25461500	4.14568900	1.26699000
H	1.75127700	2.71807700	2.20047500
H	2.07856000	2.77789800	0.46420500
C	-0.95521000	2.69894100	2.14466900
H	-0.47672000	2.45045400	3.09882200
H	-1.19899000	3.76876500	2.15519500
H	-1.88440100	2.12857300	2.07692800
C	-0.07429400	3.88143700	-1.15170300
H	-0.50630200	3.94387600	-2.15667400
H	-0.36957600	4.78204500	-0.59876600
H	1.01389300	3.87103300	-1.25328300
C	-2.12573700	2.60479900	-0.51458400
H	-2.54519400	3.49523000	-0.03094200
H	-2.44061700	2.59727400	-1.56303000
H	-2.54333000	1.71629300	-0.03753500
F	-2.52126100	-0.28090000	-2.36845300
F	-1.22902800	-1.95230500	1.89674800
K	5.28756600	-0.86133200	-0.02872800
K	0.37623900	-2.94657200	-0.07568600
H	-4.90485900	-0.80447800	-1.62562500
H	-3.75278800	-2.26662400	2.29185100
H	-5.56085200	-1.74425300	0.61288100
**24**			
C	2.18119800	-0.24902100	-1.26275800
C	2.51853400	0.69289500	-0.04181300
O	1.22026900	1.06100500	0.43738300
O	0.99098700	-0.90424000	-0.82391200
B	0.22114700	0.00113200	0.07864200
C	-1.08655300	0.69968500	-0.63069400
C	-2.06563000	0.00913600	-1.34170900
C	-1.30101600	2.09219000	-0.57973400
C	-3.18122700	0.57430300	-1.94566500
C	-2.40720500	2.71892200	-1.16278700
C	-3.35811500	1.95630600	-1.84612500

O	-0.14081500	-0.79753800	1.28714600
C	-1.41267700	-1.00632100	1.91013000
O	-1.81757800	-0.07250500	2.64390400
O	-1.91982600	-2.12931800	1.69742800
C	3.26781800	-0.05061800	1.08245400
H	4.29149700	-0.31767700	0.79383200
H	3.33676900	0.60400600	1.96181600
H	2.73269100	-0.95872100	1.37591600
C	3.29051100	1.96460000	-0.41056600
H	3.47716800	2.56199000	0.49090800
H	4.26442700	1.72571100	-0.85653400
H	2.72769600	2.58420300	-1.11345200
C	3.24701100	-1.30946800	-1.56346400
H	2.93337800	-1.91319600	-2.42393700
H	4.20996900	-0.84813600	-1.81602500
H	3.39895300	-1.98184900	-0.71355700
C	1.87991000	0.53848700	-2.55295700
H	2.78526700	0.98677700	-2.97996600
H	1.45758600	-0.14961000	-3.29393300
H	1.14673100	1.33046300	-2.37711800
F	-1.93712100	-1.36487700	-1.49995700
K	0.11457500	1.53679600	2.78957400
K	-0.39628400	-3.07701600	-0.14963800
H	-0.54652100	2.70590900	-0.09102000
H	-2.52233400	3.79812700	-1.09185100
H	-4.22426900	2.42671800	-2.30419100
H	-3.88783800	-0.05809600	-2.47473700

25-ts

C	-0.62447200	2.55692500	-0.46082000
C	0.10154600	2.27489700	0.93015400
O	0.44578400	0.86481400	0.84070600
O	-0.13537100	1.48258300	-1.30755400
B	0.34387000	0.48770000	-0.49773100
C	-1.91798300	-1.03698700	-0.38376900
C	-2.49417700	-1.02403500	-1.67864900
C	-2.83241500	-1.43107500	0.57082300
C	-3.83231800	-1.35450000	-1.93950900
C	-4.17391000	-1.77120200	0.41441900
C	-4.68237600	-1.72465400	-0.88805300
O	1.07728200	-0.62041400	-0.93820800
C	2.40433000	-0.76867300	-0.59486400
O	3.22641900	0.10767400	-0.95236300
O	2.68041500	-1.84030300	0.04147000
C	1.42810900	3.03180700	1.09780000
H	1.26903500	4.11176700	1.19707800

H	1.92465900	2.67525000	2.00762400
H	2.10081000	2.84818800	0.25440900
C	-0.77712300	2.48280300	2.16413800
H	-0.20312300	2.24268800	3.06691600
H	-1.10309600	3.52723000	2.24146600
H	-1.66036700	1.84056000	2.14262900
C	-0.23161400	3.88048900	-1.12308900
H	-0.75069000	3.96857300	-2.08344600
H	-0.52646100	4.73345200	-0.49928600
H	0.84260900	3.93850000	-1.31605000
C	-2.15381300	2.45479300	-0.39431800
H	-2.57985800	3.28023500	0.18841500
H	-2.55418000	2.50996300	-1.41193800
H	-2.47645700	1.50369200	0.03263800
F	-2.36203000	-1.53926300	1.93042900
K	5.20223600	-1.37534500	-0.34319200
K	0.17786600	-2.04870600	1.22920100
H	-1.87302700	-0.72840100	-2.52680400
H	-4.21736000	-1.32165100	-2.95836500
H	-5.72364900	-1.97795600	-1.07553200
H	-4.79078300	-2.05861000	1.26288500

26

C	-2.10667900	-1.24804700	-0.95217600
C	-2.22453600	-1.33643100	0.62094100
O	-0.93698200	-0.88960200	1.05381100
O	-1.26170300	-0.10886200	-1.13652100
B	-0.35211100	0.04960000	0.04651600
C	1.21077400	-0.35049600	-0.23406000
C	2.07417700	0.44090900	-1.02053700
C	1.75792200	-1.54481700	0.27559400
C	3.39408300	0.07063500	-1.29416100
C	3.07890700	-1.94281000	0.02601300
C	3.87100700	-1.12093700	-0.76215700
O	-0.51954300	1.46349200	0.52555000
C	0.42506500	2.52042800	0.64193200
O	1.11007600	2.53173200	1.68977700
O	0.40650600	3.34808300	-0.30426100
C	-3.29864100	-0.38796000	1.19093700
H	-4.31574400	-0.71554200	0.94375400
H	-3.21153200	-0.36971000	2.28422200
H	-3.15429600	0.63495200	0.83108200
C	-2.46661700	-2.75043700	1.16122400
H	-2.53455700	-2.72327000	2.25583600
H	-3.40725700	-3.16898100	0.78094600
H	-1.65170500	-3.42750300	0.89143600

C	-3.43663700	-0.99916400	-1.67502200
H	-3.26422600	-0.94042600	-2.75691400
H	-4.14700900	-1.81596300	-1.49735300
H	-3.90692500	-0.06529600	-1.34951200
C	-1.42704300	-2.48323000	-1.57287200
H	-2.07034700	-3.37057300	-1.53224700
H	-1.20383300	-2.27020600	-2.62441100
H	-0.48200800	-2.71165100	-1.07398800
K	0.53050600	0.37485100	2.90416900
K	-1.43635500	2.42257800	-1.81179000
H	3.48418800	-2.87063400	0.42045900
H	4.04885700	0.69473300	-1.89540200
H	1.72690800	1.39270000	-1.41491200
H	1.12348700	-2.19930600	0.87048700
F	5.16001700	-1.49300800	-1.02022500

27-ts

C	-0.45211200	2.61635100	-0.76955600
C	0.17186100	2.50828700	0.69199400
O	0.54912300	1.11016900	0.78927700
O	0.13014300	1.47413200	-1.45086400
B	0.57829100	0.59693200	-0.48869700
C	-1.66393800	-1.02537100	-0.13635900
C	-2.67254500	-1.26542800	-1.10426100
C	-2.15766200	-0.99726200	1.19204500
C	-4.03722900	-1.44876200	-0.80865900
C	-3.50866200	-1.17343700	1.54368200
C	-4.42354400	-1.39755300	0.52319800
O	1.37806800	-0.50392800	-0.85788200
C	2.61560600	-0.77923000	-0.33904000
O	3.38959600	0.15571700	-0.03187800
O	2.87449100	-2.03087100	-0.25088300
C	1.46446300	3.32017600	0.87248500
H	1.27256800	4.39934000	0.84605600
H	1.90039400	3.07172000	1.84642000
H	2.20395600	3.06948600	0.10618600
C	-0.80266000	2.82700900	1.82673400
H	-0.29603000	2.69313900	2.78948700
H	-1.14687300	3.86705100	1.76776300
H	-1.67208900	2.16644700	1.80733900
C	-0.04577500	3.87432400	-1.54236800
H	-0.48843400	3.84068800	-2.54390900
H	-0.41255900	4.77843800	-1.04072100
H	1.03860600	3.95140200	-1.65651000
C	-1.97841700	2.46521700	-0.80806400
H	-2.47508700	3.32805200	-0.34856900

H	-2.29808400	2.40358300	-1.85415800
H	-2.30498300	1.55134100	-0.30891500
K	5.19068900	-1.44307800	0.76705700
K	0.30508400	-2.86782000	-0.75739100
H	-4.78404700	-1.61584600	-1.58272100
H	-3.85118500	-1.13486900	2.57627100
H	-1.46299700	-0.79878700	2.01325800
H	-2.40712700	-1.28090500	-2.16741600
F	-5.75053800	-1.57283900	0.84186900

28

C	-2.59640000	-1.20318400	-0.35307100
C	-2.79583400	-0.48706500	1.04172800
O	-1.52667900	0.07929200	1.27191000
O	-1.71079200	-0.34550600	-1.02649500
B	-0.94434400	0.49788200	-0.05378700
C	0.69080200	0.08292100	0.00501800
C	1.36716800	-0.72112500	-0.91550400
C	1.48846900	0.53253200	1.06024600
C	2.71985600	-1.04701500	-0.81232100
C	2.84315800	0.23361000	1.20071500
C	3.46921000	-0.56569500	0.25343200
O	-1.13974900	1.90929100	-0.26682700
C	-0.50416600	2.70172500	-1.24093100
O	-1.01218500	3.84392500	-1.38602600
O	0.49065400	2.20154300	-1.83548900
C	-3.85752900	0.63740800	0.97698200
H	-4.87823500	0.24481500	0.84550300
H	-3.82002600	1.20059300	1.91830700
H	-3.61901700	1.33524300	0.16967300
C	-3.14214500	-1.42864800	2.20799100
H	-3.25052900	-0.84125800	3.12987800
H	-4.08830300	-1.96286800	2.03030500
H	-2.34851400	-2.16433800	2.37395800
C	-3.87947800	-1.35459300	-1.18924900
H	-3.64078900	-1.85983400	-2.13502900
H	-4.64151300	-1.95135500	-0.66375400
H	-4.30039200	-0.37501900	-1.43311400
C	-1.94105000	-2.59970100	-0.20371500
H	-2.62491400	-3.34645300	0.22954500
H	-1.63191400	-2.94341200	-1.19822700
H	-1.04507200	-2.53732000	0.42246000
F	0.74291700	-1.24545800	-1.99780900
F	3.33030000	-1.83816600	-1.74267900
F	4.78971800	-0.88516000	0.37577000
F	3.56950700	0.70175500	2.25734200

F	0.97073400	1.32440800	2.03347800

29-ts

C	-2.27974700	-1.44925100	-0.64061000
C	-2.62142500	-1.08127600	0.86676200
O	-1.71118400	-0.02536800	1.15269300
O	-1.73768700	-0.23876500	-1.14817400
B	-1.28353300	0.56673800	-0.07050900
C	1.01945200	0.05342700	0.00505600
C	1.78095400	-0.12407800	-1.13435700
C	1.76034600	0.14496700	1.16826600
C	3.17376900	-0.20188000	-1.15482700
C	3.15281700	0.07625500	1.22821600
C	3.86715500	-0.09736700	0.04692100
O	-1.04793100	1.92028400	-0.21960500
C	-2.13246400	2.86548000	-0.32801800
O	-3.29300200	2.38397500	-0.28667000
O	-1.75275500	4.04719200	-0.44968200
C	-4.04774900	-0.52136200	1.04301900
H	-4.82118500	-1.27701600	0.83750400
H	-4.15944900	-0.18755600	2.08286000
H	-4.18315000	0.35781800	0.40405700
C	-2.39032700	-2.22097600	1.87102400
H	-2.61004900	-1.85638700	2.88253600
H	-3.05034700	-3.07710500	1.66506600
H	-1.35159400	-2.56331700	1.85913600
C	-3.49951700	-1.82370100	-1.49604100
H	-3.16935300	-2.03790000	-2.52095200
H	-4.00660500	-2.71805600	-1.10433400
H	-4.21302400	-0.99652800	-1.53730000
C	-1.21512100	-2.55828200	-0.77314900
H	-1.60046700	-3.53956600	-0.45937200
H	-0.90943800	-2.62164900	-1.82444200
H	-0.32536000	-2.31441600	-0.18708400
F	1.18458100	-0.25682600	-2.37187400
F	3.89216600	-0.38472500	-2.31071200
F	5.23204200	-0.17074100	0.06770100
F	3.85165600	0.16588400	2.40680200
F	1.14503200	0.30504400	2.39172600

30

C	-1.65964000	-0.64237800	-0.15132900
C	-1.19571600	0.81906300	0.23147700
O	-0.45551700	-1.21579200	-0.68159800
O	0.18906400	0.62403300	0.54803400
B	0.64278300	-0.51073900	-0.14208700
O	1.89331500	-0.93621500	-0.26181800

C	3.11636100	-0.01039400	-0.03371000
O	3.86114000	-0.45826300	0.83581300
O	3.11509400	0.95579000	-0.79936800
C	-1.26608000	1.80260700	-0.95038500
H	-2.30105700	2.05759200	-1.21276000
H	-0.73872700	2.72083600	-0.67068100
H	-0.76666300	1.39365300	-1.83460400
C	-1.89938100	1.42654300	1.44791700
H	-1.48845600	2.42338800	1.64504700
H	-2.97857100	1.52973300	1.27190500
H	-1.74542000	0.81938300	2.34403200
C	-2.75503700	-0.71627900	-1.21871700
H	-2.99040900	-1.76625100	-1.43021800
H	-3.67491800	-0.22207500	-0.87838400
H	-2.43233400	-0.25103000	-2.15395800
C	-2.07437400	-1.47941700	1.07260900
H	-3.02741500	-1.14146300	1.49926100
H	-2.18439600	-2.52471400	0.76212900
H	-1.30712500	-1.44024600	1.85298400

31

C	-1.20366400	0.37401300	0.00006500
C	-1.15470000	-1.01887200	0.00022700
C	0.00000000	-1.76188400	0.00001000
C	1.15470100	-1.01887300	-0.00019300
C	1.20366400	0.37401300	-0.00003600
C	0.00000000	1.07647500	0.00001200
F	2.37562900	1.08460400	0.00006600
F	0.00000000	2.43947900	-0.00000600
F	-2.37562900	1.08460400	-0.00006600
F	-2.40160600	-1.64596700	-0.00013800
F	2.40160500	-1.64596700	0.00008800

32-ts

C	2.15407200	-1.31467300	-0.23889700
C	0.85791200	-1.08848800	0.22299700
C	0.35956300	0.16798600	0.50722400
C	1.23236200	1.21344000	0.29821400
C	2.54056200	1.05949100	-0.15818000
C	3.00307500	-0.22577800	-0.42648600
C	-1.45152300	0.43034700	1.60914200
H	-1.17504000	1.49028400	1.76167300
C	-2.41693600	0.26099600	0.44882400
C	-3.15807800	-0.91997800	0.31828400
C	-2.64315700	1.30625900	-0.45743600
C	-4.09085200	-1.06243600	-0.71243200
H	-2.98075900	-1.71309800	1.03933800

C	-3.57940500	1.17155200	-1.48560400
H	-2.06506700	2.22420600	-0.35920000
C	-4.30710000	-0.01778300	-1.61965200
H	-4.65423700	-1.98940300	-0.80895800
H	-3.74145500	1.99110800	-2.18404900
H	-5.03515600	-0.12769200	-2.42145400
F	0.83007000	2.51485800	0.53023500
F	3.37716100	2.11717000	-0.35042600
F	4.27046100	-0.41695900	-0.87193700
F	2.61772800	-2.56342500	-0.51954700
F	0.07524900	-2.20178500	0.37371500
O	-1.48590900	-0.35122000	2.58478800

33

C	-1.84919400	1.43657800	0.03808500
C	-0.59573400	0.90880200	0.33214900
C	-0.33167200	-0.46615300	0.34194700
C	-1.41702000	-1.28084500	0.01492600
C	-2.68849900	-0.78784100	-0.27526000
C	-2.90985400	0.58353600	-0.25965700
C	1.00728900	-1.06771000	0.94136100
H	0.98035600	-2.13985700	0.61835500
C	2.22795600	-0.48587200	0.17343000
C	3.24404100	0.13810400	0.90332300
C	2.38652000	-0.63012400	-1.21190300
C	4.39141700	0.61938000	0.26325900
H	3.08985600	0.22604500	1.97615000
C	3.52900400	-0.14965400	-1.86015600
H	1.60624500	-1.12568900	-1.79081600
C	4.53976200	0.47987500	-1.12187400
H	5.17414100	1.10587300	0.84469800
H	3.63324700	-0.26556900	-2.93849500
H	5.43183200	0.85428900	-1.62174600
F	-1.27284100	-2.63213400	-0.04697000
F	-3.70870900	-1.62575000	-0.58356700
F	-4.13713500	1.08587900	-0.53171300
F	-2.05588000	2.77557000	0.02574400
F	0.38163200	1.80028300	0.60083800
O	1.00208000	-0.88477000	2.25688600

34-ts

C	-2.43523700	-2.35826100	-0.70621800
C	-2.70331900	-2.04640400	0.83127500
O	-2.64685300	-1.09213300	-1.32598500
O	-2.43337500	-0.64792800	0.93246900
B	-2.52030600	-0.07952200	-0.35814800
O	-2.89482800	1.22202000	-0.60021000

C	-4.29070300	1.60469500	-0.56335800
O	-4.47713700	2.81156300	-0.81115400
O	-5.10008700	0.68492200	-0.29272500
C	-4.17685500	-2.24566600	1.24003700
H	-4.48513800	-3.30018100	1.18750700
H	-4.29614700	-1.90223100	2.27560000
H	-4.82780200	-1.62494300	0.61568000
C	-1.78964200	-2.80318000	1.80321600
H	-2.02607600	-2.50030800	2.83126800
H	-1.93850500	-3.89021400	1.72504800
H	-0.73597300	-2.57688700	1.62226600
C	-3.41563000	-3.36483200	-1.32662000
H	-3.17937800	-3.48578200	-2.39164800
H	-3.33520600	-4.35038000	-0.84487200
H	-4.44783700	-3.01263900	-1.24869200
C	-0.99075400	-2.80225300	-1.00422600
H	-0.74907500	-3.76994900	-0.54176600
H	-0.87641600	-2.90057300	-2.09168400
H	-0.29354700	-2.02755600	-0.67256500
C	3.86698700	-0.32370100	-1.21896900
C	2.70959100	0.38579900	-0.91382900
C	1.91518000	0.09655300	0.20551600
C	2.38236200	-0.94149400	1.01339200
C	3.53182800	-1.67936200	0.72954900
C	4.27960300	-1.37223700	-0.39884800
C	0.49185000	0.74570400	0.39579100
H	0.15322400	0.40522300	1.40223400
C	0.62465500	2.28484500	0.52883200
C	-0.35396400	3.09308300	-0.06484100
C	1.63700000	2.89349400	1.28600400
C	-0.30942100	4.48296700	0.09271400
H	-1.16082700	2.61058200	-0.61152800
C	1.69114100	4.28419000	1.43492300
H	2.39756400	2.27583700	1.76537800
C	0.71390500	5.08721900	0.83374100
H	-1.09209800	5.09045000	-0.35803300
H	2.49266100	4.73854300	2.01850600
H	0.74582900	6.17048200	0.95057600
F	1.72614600	-1.27848800	2.15539200
F	3.93986100	-2.68398900	1.54758900
F	5.39826600	-2.07967800	-0.69947600
F	4.61702400	-0.00355300	-2.30395700
F	2.38968500	1.39438000	-1.74544200
O	-0.27947300	0.29444700	-0.60464200

35

C	3.17789600	0.05272200	-1.53301500
C	2.83728200	1.27642100	-0.57184700
O	2.74973800	-1.07358400	-0.80513600
O	1.74129200	0.79911600	0.18003800
B	1.70472300	-0.69858700	0.15506000
O	1.70023300	-1.36718100	1.42563100
C	2.69738400	-1.47131500	2.41721300
O	2.31754000	-2.09144200	3.44404800
O	3.82471800	-0.96157900	2.18115200
C	3.99231500	1.62117000	0.39199900
H	4.87645400	2.01254800	-0.13661900
H	3.63878300	2.39671600	1.08608700
H	4.25050100	0.73834000	0.98892600
C	2.41261600	2.55883700	-1.31425100
H	2.16712000	3.33423900	-0.57610900
H	3.22199900	2.94437900	-1.95341900
H	1.52545100	2.39354200	-1.93436400
C	4.67610100	-0.11024500	-1.84256200
H	4.81770100	-0.98075000	-2.49853000
H	5.08698500	0.77414500	-2.35377500
H	5.23746500	-0.28742900	-0.92133000
C	2.39743900	0.10596100	-2.86998800
H	2.72241600	0.93324400	-3.51959400
H	2.56610400	-0.83917300	-3.40339900
H	1.32303200	0.19302100	-2.68385200
C	-2.34071200	2.18533100	-0.97670400
C	-1.83368600	0.89091700	-0.91661700
C	-1.44417600	0.28834300	0.28400700
C	-1.61443700	1.05756700	1.43595700
C	-2.11914100	2.35828900	1.40621000
C	-2.47923000	2.92756900	0.19211700
C	-0.76484100	-1.10603600	0.31167900
H	-0.47889100	-1.30652200	1.35629400
C	-1.75524500	-2.19836200	-0.11164300
C	-1.34095800	-3.18899300	-1.01122700
C	-3.05028500	-2.27727300	0.42235500
C	-2.20402200	-4.22751100	-1.37427100
H	-0.33151500	-3.11425300	-1.40376600
C	-3.91628700	-3.31438400	0.06209500
H	-3.38479500	-1.52303800	1.13290900
C	-3.49700600	-4.29667900	-0.84269000
H	-1.86335400	-4.98976500	-2.07389300
H	-4.91697600	-3.35707600	0.49072900
H	-4.16814900	-5.10680800	-1.12501300
F	-1.31612900	0.55325900	2.64806000

F	-2.27607200	3.06988700	2.54694000
F	-2.97822200	4.18587400	0.14523000
F	-2.71629800	2.73175200	-2.15876700
F	-1.74802300	0.22031600	-2.08151600
O	0.35556800	-1.10110500	-0.48811000

VIII. Investigation of the Reaction Mechanism

(a) Reactivity test of acetophenone with pentafluorophenyl-Bpin and K_2CO_3

A mixture of acetophenone (0.04 mmol, 4.8 mg), pentafluorophenyl-Bpin (0.04 mmol, 6.7 mg) and K_2CO_3 (0.04 mmol, 5.5 mg, 1 equiv.) was dissolved in 0.7 mL C_6D_6 in a Young's tap NMR tube. The ^{1}H and ^{11}B NMR spectra of the mixture were recorded immediately. Then, the mixture was heated at 120 °C for 4 h. They subsequently were studied by ^{1}H and ^{11}B NMR spectra, which revealed that the formation of product **3s-2** was observed.

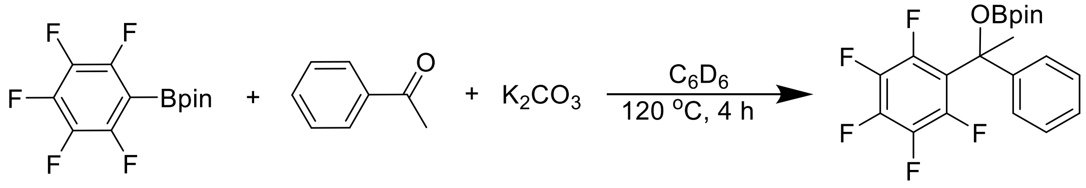

3s-2

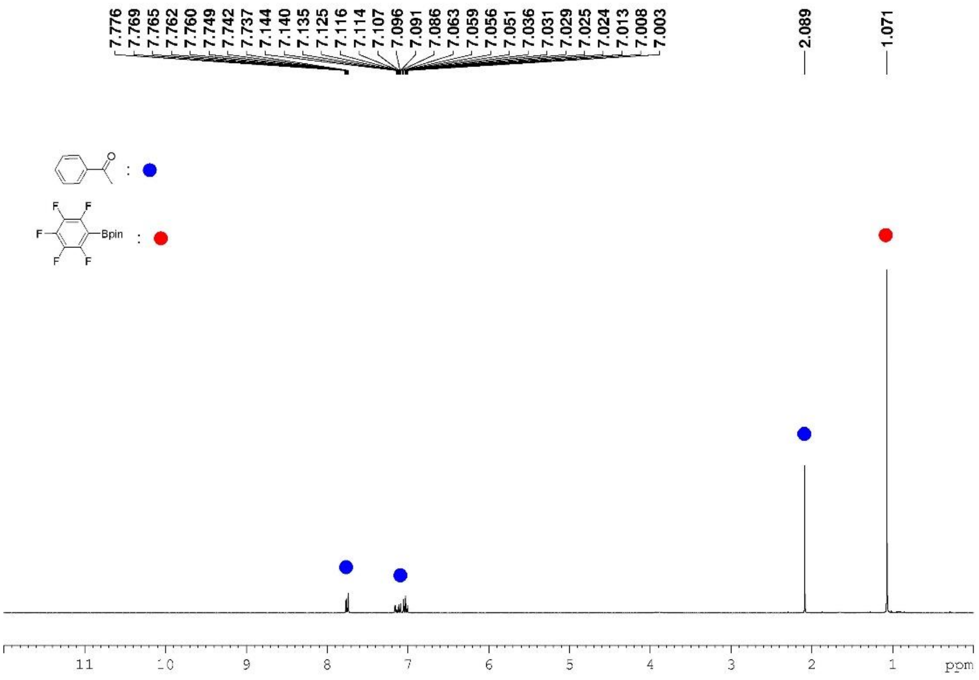

Figure S10. ^{1}H NMR spectrum at 0 min (300 MHz, C_6D_6).

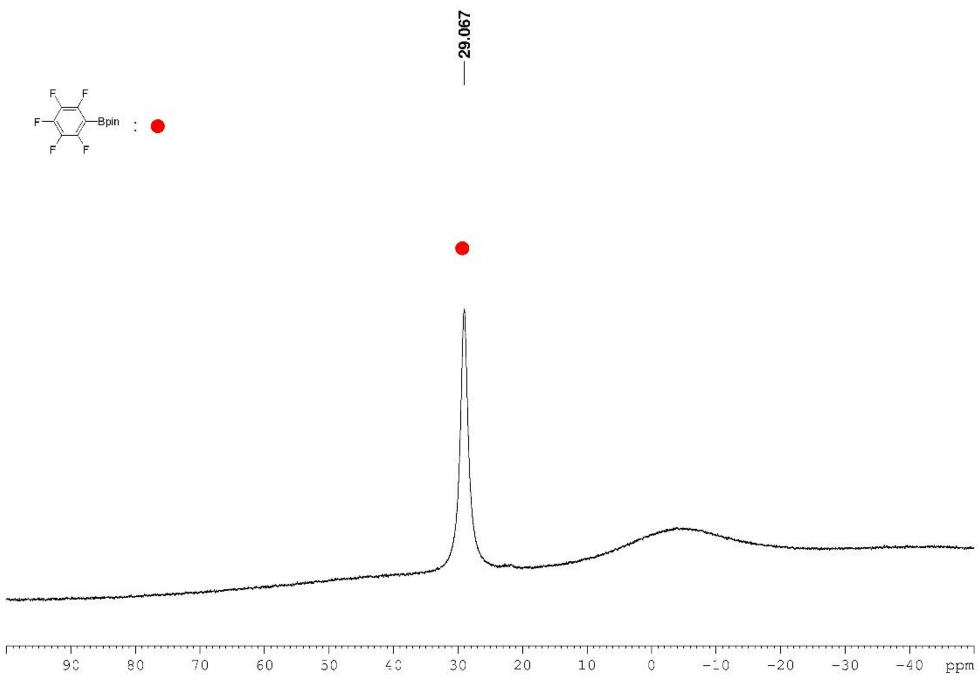

Figure S11. ^{11}B NMR spectrum at 0 min (96 MHz, C_6D_6).

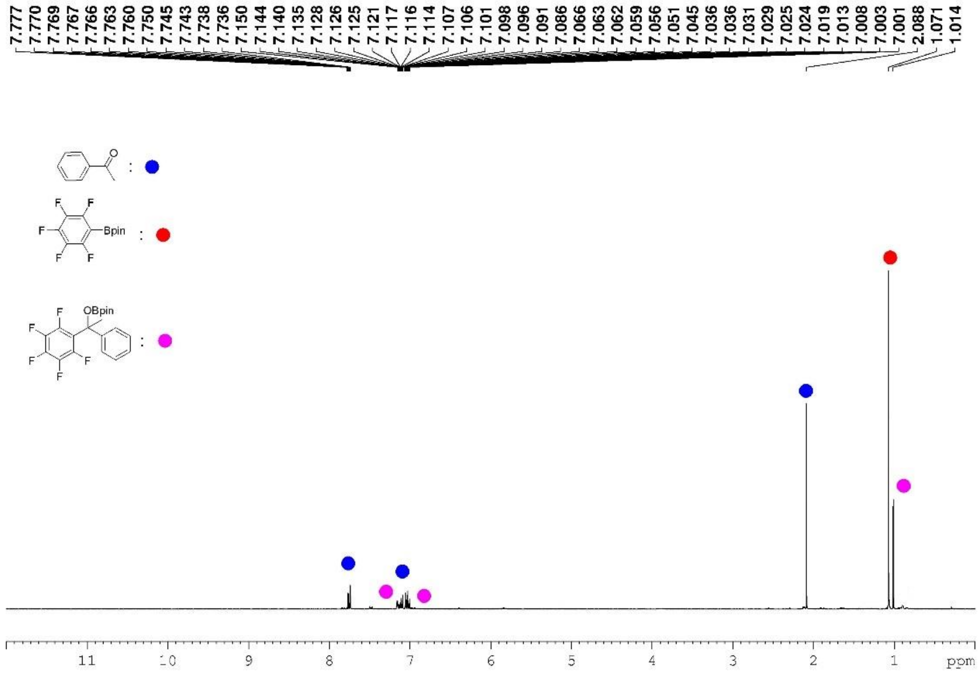

Figure S12. ^{1}H NMR spectrum after 4 h heating (300 MHz, C_6D_6).

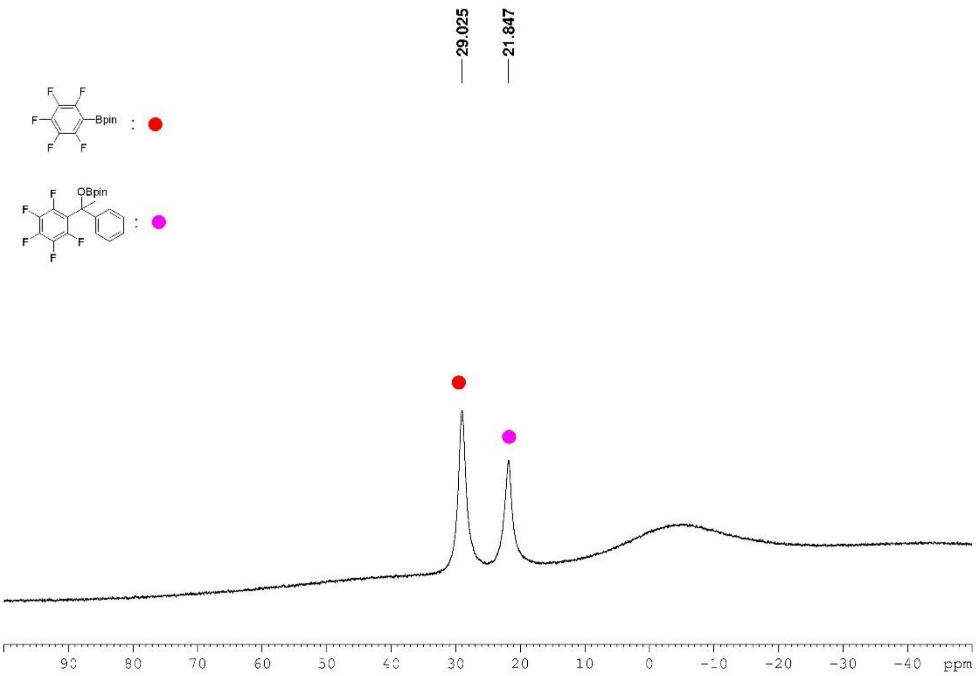

Figure S13. ^{11}B NMR spectrum after 4 h heating (96 MHz, C_6D_6).

HRMS data of intermediate D

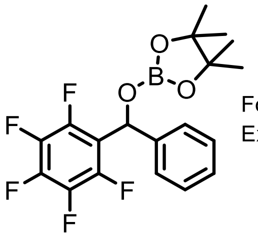

Formula: $C_{19}H_{18}BF_5O_3$
Exact Mass: 400.1269
Found: 400.1263

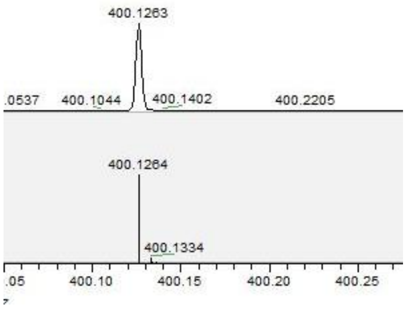

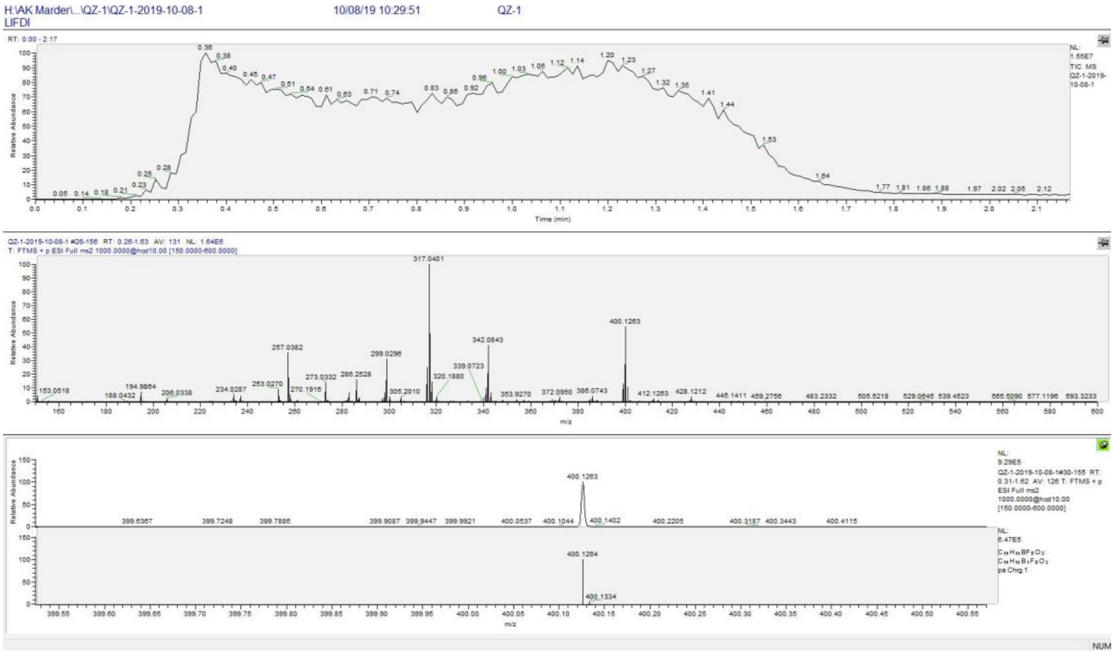

IX. References

[1] a) T. Ishiyama, J. Takagi, K. Ishida, N. Miyaura, N. R. Anastasi, J. F. Hartwig, *J. Am. Chem. Soc*. **2002**, *124*, 390-391; b) T. Ishiyama, J. Takagi, K. Ishida, N. Miyaura, J. F. A Hartwig, *Angew. Chem. Int. Ed.* **2002**, *41*, 3056-3058; c) Y. P. Budiman, A. Friedrich, U. Radius, T. B. Marder, *ChemCatChem* **2019**, *11*, 5387-5396.

[2] G.-F. Du, F. Xing, C.-Z. Gu, B. Dai, L. He, *RSC Adv*. **2015**, *5*, 35513-35517.

[3] M.-T. Meng, K. Cheng, *J. Org. Chem.* **2018**, *83*, 3275-3284.

[4] S. Chang, J. Wang, L. Dong, D. Wang, B. Feng, Y. Shi, *RSC Adv*. **2017**, *7*, 51928-51934.

[5] P. Lei, Y. Ling, M. Szostak, *J. Org. Chem.* **2017**, *82*, 6638-6646.

[6] M.-Z. Li, C. Wang, H. Ge, *Org. Lett*. **2011**, *13*, 2062-2064.

[7] a) G. M. Sheldrick, *Acta Crystallogr. Sect. A: Found. Crystallogr.* **2008**, *64*, 112-122; b) C. B. Hübschle, G. M. Sheldrick, B. Dittrich, *J. Appl. Crystallogr.* **2011**, *44*, 1281-1284; c) G. M. Sheldrick, *Acta Crystallogr. Sect. C: Struct. Chem.* **2015**, *71*, 3-8; d) G. M. Sheldrick, *Acta Crystallogr. Sect. A: Found. Adv.* **2015**, *71*, 3-8.

[8] B. Moulton, M. J. Zaworotko, *Chem. Rev.* **2001**, *101*, 1629-1658.

[9] M. J. Frisch, G. W. Trucks, H. B. Schlegel, G. E. Scuseria, M. A. Robb, J. R. Cheeseman, G. Scalmani, V. Barone, B. Mennucci, G. A. Petersson, H. Nakatsuji, M. Caricato, X. Li, H. P. Hratchian, A. F. Izmaylov, J. Bloino, G. Zheng, J. L. Sonnenberg, M. Hada, M. Ehara, K. Toyota, R. Fukuda, J. Hasegawa, M. Ishida, T. Nakajima, Y. Honda, O. Kitao, H. Nakai, T. Vreven, Jr. J. A. Montgomery, J. E. Peralta, F. Ogliaro, M. Bearpark, J. J. Heyd, E. Brothers, K. N. Kudin, V. N. Staroverov, T. Keith, R. Kobayashi, J. Normand, K. Raghavachari, A. Rendell, J. C. Burant, S. S. Iyengar, J. Tomasi, M. Cossi, N. Rega, J. M. Millam, M. Klene, J. E. Knox, J. B. Cross, V. Bakken, C. Adamo, J. Jaramillo, R. Gomperts, R. E. Stratmann, O. Yazyev, A. J. Austin, R. Cammi, C. Pomelli, J. W. Ochterski, R. L. Martin, K. Morokuma, V. G. Zakrzewski, G. A. Voth, P. Salvador, J. J. Dannenberg, S. Dapprich, A. D. Daniels, O. Farkas, J. B. Foresman, J. V. Ortiz, J. Cioslowski, and D. J. Fox, Gaussian 09, revision E.01; Gaussian, Inc.: Wallingford, CT, **2013**.

[10] A. D. Becke, *J. Chem. Phys*. **1993**, *98*, 5648-5652.

[11] C. Lee, W. Yang, R. G. Parr, *Phys. Rev. B: Condens. Matter* **1988**, *37*, 785-789.

[12] K. Fukui, *Acc. Chem. Res.* **2002**, *14*, 363-368.

[13] E. Cancès, B. Mennucci, J. Tomasi, *J. Chem. Phys.* **1997**, *107*, 3032-3041.

[14] M. Cossi, V. Barone, R. Cammi, J. Tomasi, *Chem. Phys. Lett.* **1996**, *255*, 327-335.

[15] A. V. Marenich, C. J. Cramer, D. G. Truhlar, *J. Phys. Chem. B* **2009**, *113*, 6378-6396.

[16] Y. Zhao, D. G. Truhlar, *Acc. Chem. Res.* **2008**, *41*, 157-167.

[17] C.Y. Legault, CYLView, Université de Sherbrooke, Canada, **2009**.
